# Stereoelectronic manipulation of ligands for perovskite solar cells

**DOI:** 10.1038/s41586-026-10626-0

**Published:** 2026-05-13

**Authors:** Tinghuan Yang, Erxin Zhao, Nan Wu, Xiaoming Chang, Chenqing Tian, Hai-Long Wang, Lu Zhang, Nannan Gu, Ting Nie, Ye Yang, Zheng Zhang, Tianfei Xu, Xin Chen, Shuang Wang, Tianqi Niu, Niansheng Xu, Chuang Ma, Haojin Li, Buyi Yan, Zicheng Ding, Shengzhong Frank Liu, Feng Gao, Kui Zhao

**Affiliations:** 1 Key Laboratory of Applied Surface and Colloid Chemistry, Ministry of Education; Shaanxi Key Laboratory for Advanced Energy Devices; Shaanxi Engineering Lab for Advanced Energy Technology; Institute for Advanced Energy Materials; School of Materials Science and Engineering, Shaanxi Normal University, Xi’an, China; 2 State Key Laboratory of Physical Chemistry of Solid Surfaces, College of Chemistry and Chemical Engineering, Innovation Laboratory for Sciences and Technologies of Energy Materials of Fujian Province (IKKEM), Xiamen University, Xiamen, China; 3 Hangzhou Microquanta Semiconductor Co., Ltd., Hangzhou, China; 4 Department of Physics, Chemistry and Biology (IFM), Linköping University, Linköping, Sweden; 5 Key Laboratory of Photoelectric Conversion and Utilization of Solar Energy, Dalian Institute of Chemical Physics, Center of Materials Science and Optoelectronics Engineering, University of Chinese Academy of Sciences, Dalian, China

**Keywords:** Solar cells, Solar cells

## Abstract

Interfacial losses at perovskite/charge transport layer (CTL) heterojunctions persist as a critical barrier to achieving high-performance perovskite solar cells (PSCs)^1–5^. Although molecular ligands can passivate interfacial vacancy defects, their vertical anchoring geometry compromises charge transport by increasing interfacial transport pathways. Here we demonstrate that stereoelectronic manipulation of ligand adsorption topology advances interfacial minimum energy loss for efficient and stable PSCs. By strategically replacing benzene carbons with nitrogen atoms to create pyridine or pyrimidine rings, we design ligands that concurrently anchor to the perovskite through Pb–N coordination bonds and Pb–I–π interactions, endowing a single molecule with dual, synergistic binding modes. This mutually reinforcing stereoelectronic interplay drives thermodynamically favourable planar alignment of ligands, enabling atomic-scale defect mitigation while maintaining sub-nanometre-scale charge transfer across the interface. The optimized interfacial architecture achieves a stabilized power output of 26.85%, with certificated reverse-scan and forward-scan efficiencies of 27.41% and 26.35%, respectively. Furthermore, the solar modules exhibit exceptional operational stability, retaining 85.8% of initial module efficiency after 258 days of outdoor real-time field testing.

## Abstract (web summary)

By tuning the stereoelectronic properties of ligand adsorption topology to optimize the interfacial architecture and improve stability in perovskite solar cells, high certified power outputs of nearly 27% in the laboratory are demonstrated.

## Main

PSCs have redefined photovoltaic technology frontiers through unprecedented efficiency gains, achieving certified power conversion efficiencies (PCEs) approaching 27% in laboratory settings^6–9^. However, there remain losses at the perovskite/CTL interfaces—a critical barrier to both efficiency ceilings and industrial scalability^1–5^. The losses at perovskite/CTL interfaces are fundamentally governed by two quantum-mechanically antagonistic processes^10–13^. One is defect-mediated non-radiative recombination, which is dominated by Shockley–Read–Hall kinetics with characteristic abundant interfacial trap states at untreated interfaces^14–16^. The other is interfacial transport resistance limited by quantum-mechanical transmission and wavefunction mismatch across the heterojunction^17,18^.

Existing molecular design strategies face an intrinsic trade-off. Ligands (for example, phenethylammonium) achieve defect passivation by means of intercalation of amine terminal into the Pb–I framework for ionic interaction bonding but impose prohibitive transport distance (*d* > 2 nm) and parasitic transport resistance owing to outside carbon-chain organic moieties^19–24^. This trade-off poses a fundamental challenge in molecular design: atomic-scale defect mitigation requires strong interfacial bonding (vertical alignment), whereas optimal charge transport demands minimized transport distance (planar alignment). Resolving this trade-off requires precise modulation of interfacial energy losses through synergistic defect passivation and charge transport geometry optimization, thereby enabling minimum interfacial losses. Yet, even state-of-the-art remedies including those using vertical ligands or geometric size-matching passivants^13,24–26^, leave this fundamental dilemma unresolved.

Here we report stereoelectronic manipulation of ligands that simultaneously suppresses atomic-scale defects and optimizes molecular-level charge transport at perovskite interfaces. By integrating N-heteroaromatic motifs into ligands, we reorient their adsorption topology from vertical to planar configurations by means of dual Pb–N coordination and Pb–I–π interactions with the perovskite lattice. This interfacial reconfiguration enhances interfacial binding energy, reduces charge transport distance and suppresses energy disorder, while accelerating charge extraction. Through synergistic energy loss management, our devices achieve a certified stabilized power output of 26.85%. Our ligand design strategy is also applicable to wide-gap perovskites and large-area PSCs/modules. Moreover, large-area modules retain 85.8% of initial efficiency (23.10%) after 258 days of outdoor real-time field testing, demonstrating unprecedented efficiency–stability synergy.

## Stereoelectronic manipulation of ligands

**Fig. 1:**
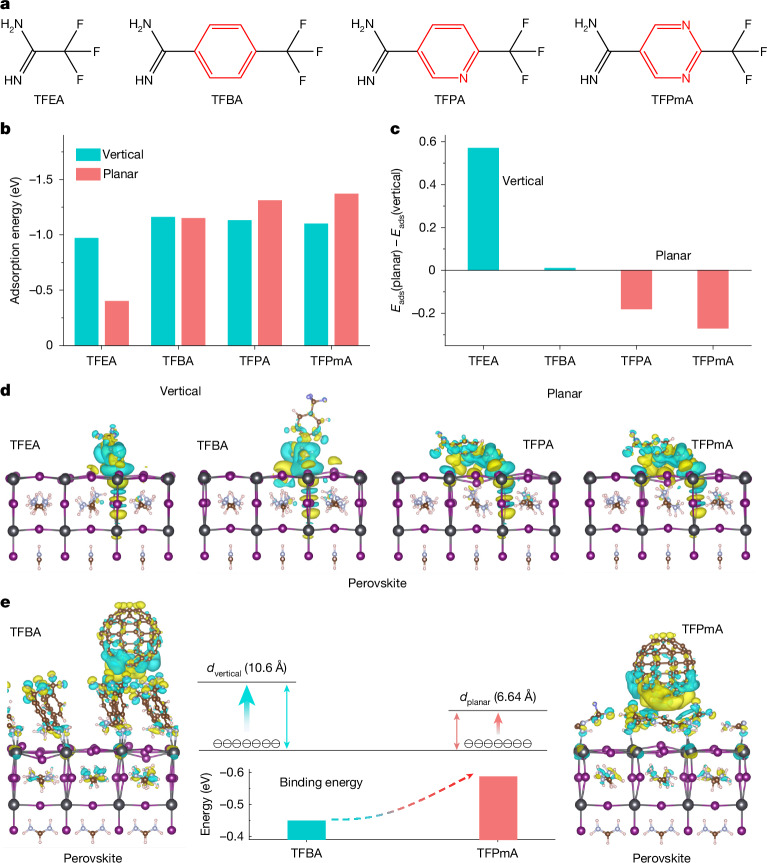
Ligand structures and adsorption topologies on the perovskite surface. **a**, Chemical structures of the four ligands. **b**, Adsorption energies of each ligand on the perovskite surface in vertical and planar topology. **c**, Formation-energy differences between vertical and planar adsorption topology for each ligand on the perovskite surface. **d**, Differential charge–density maps for ligands adsorbed in vertical versus planar topology, illustrating regions of charge accumulation and depletion. **e**, Differential charge–density maps, binding energies and charge transport distance of perovskite/ligand/C_60_ interfaces with TFBA or TFPmA. Source data

The stereoelectronic design principle was designed to precisely control ligand adsorption topology through nitrogen-mediated orbital hybridization. Four multifunctional ligands were engineered with amidino (FA^+^, for the Pb–N ionic bonding) as defect passivation and -CF_3_ (hydrophobic moiety) as enhancement of the environmental stability for the perovskite surface^22,27–29^. At the centre of the ligands, we introduced conjugated benzene rings and progressive nitrogen incorporation in aromatic cores gradually replaced the C on the benzene rings with N to form N-heteroaromatic motifs, thereby modulating the interaction ability between the central part of the ligands and the Pb–I framework. Figure 1a shows the four amidino-based derivatives: trifluoroethylamidine (TFEA), trifluorobenzamidine (TFBA), trifluoropyridylamidine (TFPA) and trifluoropyrimidylamidine (TFPmA), distinguished by their central conjugated cores (highlighted in red) benzene, pyridine (one N atom) and pyrimidine rings (two N atoms), respectively.

First-principles density functional theory (DFT) calculations were used to explain the stereoelectronic factors governing ligand adsorption topology on the Pb–I framework of perovskites (see details in Supplementary Note 1). Electrostatic potential maps revealed increasingly negative surface regions associated with N-heteroaromatic motifs (Supplementary Fig. 1), concomitant with an increase in molecular dipole moment from 4.18 D for TFEA to 4.38 D, 6.39 D and 6.48 D for TFBA, TFPA and TFPmA, respectively (Supplementary Fig. 2). We evaluated adsorption energies (*E*_ads_) for each ligand in both vertical and planar orientations on the Pb–I framework. Vertically adsorbed *E*_ads_ values were −0.97 eV (TFEA), −1.16 eV (TFBA), −1.13 eV (TFPA) and −1.10 eV (TFPmA), whereas planar adsorption energies were −0.40 eV, −1.15 eV, −1.31 eV and −1.37 eV, respectively (Fig. 1b and Supplementary Fig. 3). The difference Δ*E* = *E*_ads_(planar) − *E*_ads_(vertical) decreased from 0.57 eV (TFEA) to almost zero (0.01 eV) for TFBA and became negative for TFPA (−0.18 eV) and TFPmA (−0.27 eV), indicating a thermodynamic preference shift from the vertical (TFEA) to random (TFBA) and then planar (TFPA, TFPmA) topology as the number of N-heteroaromatic units increases (Fig. 1c). This thermodynamic preference shift was also observed on FA–I-terminated surfaces (Supplementary Figs. 4 and 5 and Supplementary Note 1). Charge–density difference analysis (Fig. 1d) indicated that the planar topology enabled multicentre interactions—namely, Pb–N coordination bonds and Pb–I–π interactions between Pb–I frameworks and ligands.

We next examined how ligand adsorption topology modulates charge transport across the perovskite/C_60_ interface. Using TFBA in a vertical topology and TFPmA in a planar topology as representative cases, we found that the vertical transport distance (*d*_vertical_) from the perovskite surface to the C_60_ acceptor was 10.60 Å, whereas the corresponding distance for the planar topology (*d*_planar_) contracted by about 37% to 6.64 Å (Fig. 1e). On reorientation from vertical to planar, the N-heteroaromatic rings on the ligand approached the C_60_ molecule more closely, resulting in enhanced perovskite/ligands/C_60_ interactions. Consequently, the overall binding energy of the perovskite/ligands/C_60_ interface decreased from −0.44 eV for TFBA to −0.59 eV for TFPmA. Theoretical simulation reveals that the ligand adsorption topology opens both sub-nanometre charge-transfer channels and enhances the perovskite/ligand/C_60_ electrostatic coupling, yielding an interfacial electronic landscape that favours efficient charge extraction.

## Multicentre interactions resolved via spectroscopies

**Fig. 2:**
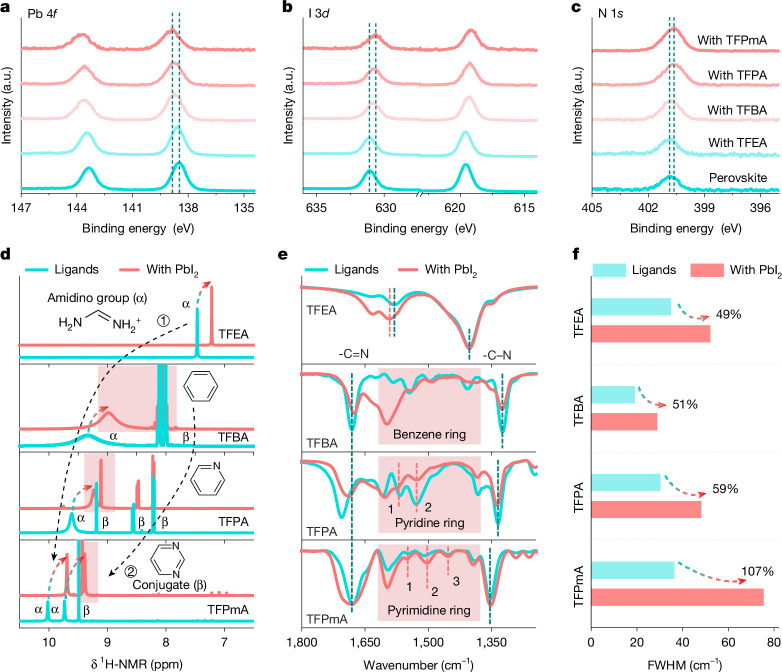
The interaction of ligands with the perovskite framework. **a**–**c**, XPS spectra of Pb 4*f* (**a**), I 3*d* (**b**) and N 1*s* (**c**) from perovskite films treated with TFEA, TFBA, TFPA or TFPmA. **d**, ^1^H-NMR spectra of different ligands interacting with PbI_2_. **e**, FTIR spectra of different ligands interacting with PbI_2_. **f**, FWHM of the -C=N vibrational peak extracted from the FTIR spectrum. a.u., arbitrary units. Source data

To explain the chemical mechanisms of stereoelectronic factors governing multicentre interactions between ligands and the Pb–I framework, we conducted X-ray photoelectron spectroscopy (XPS), ^1^H nuclear magnetic resonance (^1^H-NMR), and Fourier-transform infrared spectroscopy (FTIR) for series of samples (see details in Supplementary Note 2). The XPS spectra of calibrated C 1*s* and F 1*s* references for FAPbI_3_-based perovskite with ligands are provided in Supplementary Figs. 6 and 7. As shown in Fig. 2a–c, monotonic binding energy shifts were observed in Pb 4*f*_7/2_ (138.49 → 138.84 eV), I 3*d*_3/2_ (631.09 → 630.63 eV) and N 1*s* (400.81 → 400.62 eV) of the perovskite/ligand systems with increasing N-heteroaromatic content. These results indicate that N-heteroaromatic rings enhanced charge redistribution of perovskite with Pb–N coordination bonds and Pb–I–π interactions between Pb–I frameworks and ligands.

^1^H-NMR spectra indicated a paramagnetic shifting (7.47 → 10.0 ppm) of the amidino proton δH_α_ with progressive N-heteroaromatics of four ligands (Fig. 2d and Supplementary Figs. 8–11), accompanied by peak splitting (Δ*δ* = 0.27 ppm for TFPmA). Likewise, the conjugated ring δH_β_ exhibited deshielding (8.02 → 9.49 ppm, Δ*δ* = 1.47 ppm) with progressive N-heteroaromatics. On PbI_2_ interaction, δH_α_ moved to high field for all ligands (blue-red dashed line), suggesting the strong NH⋯I hydrogen-bonding interactions between the Pb–I framework and the ligand amidino moiety^30,31^. The distance between δH_α_ and δH_β_ gradually decreased and eventually overlapped (Δ*δ*: 0.94 → 0.12 → –0.04 ppm; red-shaded region) with progressive N-heteroaromatics, reflecting chelate-assisted Pb–I–π delocalization and a transition from monodentate to multicentre interactions.

FTIR spectroscopy of ligands/PbI_2_ complexes (Fig. 2e and Supplementary Figs. 12 and 13) further confirmed multicentre interactions underpinning geometric transitions. On PbI_2_ complexation, the amidino C=N stretch in TFEA upshifted by +12 cm^−^^1^ (1,580 → 1,592 cm^−1^), confirming dominant Pb–N coordination interaction. Introducing N-heteroaromatic motifs reversed this trend: TFBA and TFPA exhibited C=N downshifts (Δ*ν* = −4/−17 cm^−^^1^) with full width at half-maximum (FWHM) broadening (19 → 29/30 → 48 cm^−^^1^) (Fig. 2f). TFPmA further amplified this effect, showing strong broadening by 107% (FWHM: 36 → 75 cm^−^^1^) of C=N stretch and a −3 cm^−1^ redshift in the C–N vibration (1,354 → 1,351 cm^−^^1^). Concurrently, N-heteroaromatics participation emerged by means of split vibrational modes (1,590–1,400 cm^−^^1^): pyridine N–Pb interaction softened ring breathing (1,571/1,526 cm^−^^1^), whereas pyrimidine distortion splits the symmetric stretch into a 1,550/1,502/1,452 cm^−^^1^ triple (Fig. 2e, shaded area lines 1–3), which demonstrate direct engagement of N-heteroaromatic motifs in Pb–I–π interaction. The spectroscopic observations align with DFT-predicted multicentre interactions between ligands and the Pb–I framework, validating stereoelectronic engineering as a tool for atomic-scale control of ligand orientation and interaction cooperativity.

## Characterizations of perovskite/ligands interface

**Fig. 3:**
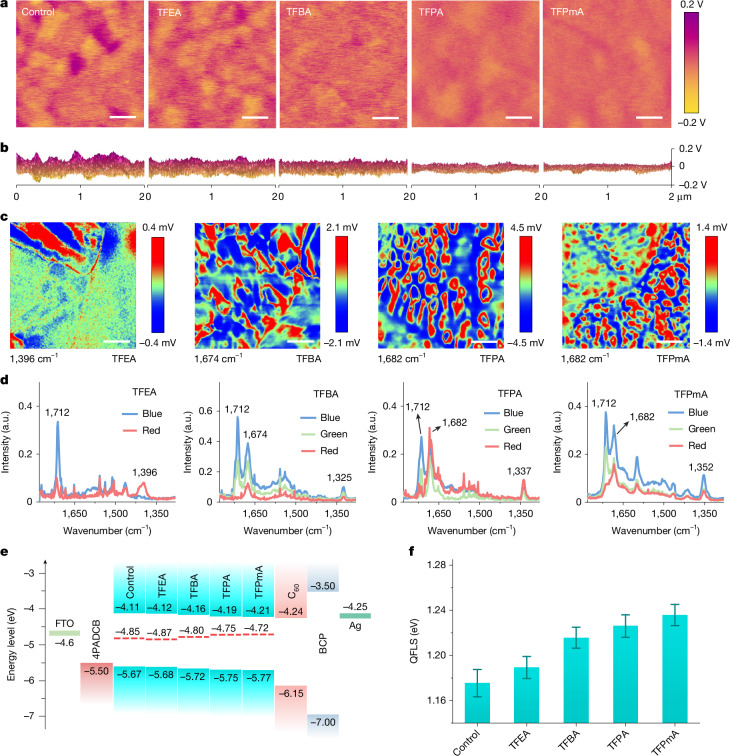
Surface characterization of ligand-modified perovskite films. **a**, KPFM topography showing surface potential variations for control and perovskite films treated with TFEA, TFBA, TFPA and TFPmA ligands. Scale bars, 400 nm. **b**, Corresponding cross-sectional potential profiles extracted from the KPFM images in **a**. **c**, Nano-infrared chemical mapping of perovskite films modified with TFEA, TFBA, TFPA and TFPmA, showing the spatial distribution of key infrared absorptions. Scale bars, 60 nm. **d**, Nano-infrared line-plot spectra acquired from the perovskite surface mappings (Supplementary Fig. 21; the blue, green and red lines corresponding to the blue, green and red mappings, respectively), highlighting the vibrational signatures of each ligand. **e**, Energy-level diagrams comparing the valence-band and conduction-band edges of untreated (control) versus ligands-treated perovskite films, derived from UPS measurements and optical absorption data. **f**, QFLS values determined for FTO/4PADCB/perovskite/ligands samples, indicating changes in photovoltage potential. a.u., arbitrary units. Source data

Complementary grazing-incidence wide-angle X-ray scattering (GIWAXS), X-ray diffraction and ultrafast transient absorption spectroscopy analyses of FAPbI_3_-based perovskite films verified that interfacial treatment preserved the intrinsic crystallographic integrity of perovskite phases, with no detectable low-dimensional phase formation or lattice distortion (Supplementary Figs. 14–16). This confirms molecular-level ligand anchoring on perovskite surface rather than bulk intercalation. Scanning electron microscopy revealed dissipation of surface-aggregated PbI_2_ domains after interfacial treatment (Supplementary Fig. 17), yielding atomically smooth interfaces with approximately 1.4–1.7 nm reduced root mean square roughness (Supplementary Fig. 18). Kelvin probe force microscopy (KPFM) quantified a roughly 61.8% decrease in surface potential differential (30.1 → 11.5 mV; Fig. 3a) with substantially narrowed spatial heterogeneity (±150 → ±50 mV; Fig. 3b and Supplementary Fig. 19) with progressive N-heteroaromatic motifs. The improved surface smoothness and potential homogenization, driven by the N-heteroaromatic motifs, enhance the contact-related interfacial quality and is beneficial for interfacial charge extraction.

Hyperspectral nano-infrared imaging (<10 nm resolution; Fig. 3c and Supplementary Note 3) mapped the nanoscale spatial heterogeneity of ligands on perovskite surfaces^32–35^. Untreated films showed uniform FA cations (-C=N, 1,712 cm^−1^) with PbI_2_-rich clusters (Supplementary Fig. 20). Ligand-treated surfaces retained this signature but exhibited distinct vibrational bands (Fig. 3d and Supplementary Fig. 21): C–N bending (TFEA, 1,396 cm^−1^), C=N/C–N stretches (TFBA: 1,674/1,325 cm^−1^; TFPA: 1,682/1,337 cm^−1^; TFPmA: 1,682/1,352 cm^−1^), corroborated by FTIR (Fig. 2d). Multivariate analysis (Fig. 3c, Supplementary Fig. 22 and Supplementary Note 3) resolved four relatively signal-active adsorption modes: sparse fractal aggregates (TFEA, about 7.7% relative signal-active fractions), discontinuous superstructures (TFBA, about 25.7%), aligned nanowires (TFPA, about 33.6%) and near-complete epitaxial nanodomains (TFPmA, about 46.3%). TFPmA achieved intra-grain uniformity, reducing the average aggregation size by 39% versus TFEA (about 39 versus about 100 nm; Supplementary Table 1), establishing a conformal passivation network that suppresses interfacial traps.

Ultraviolet photoelectron spectroscopy (UPS) revealed a systematic modulation of interfacial energy landscapes governed by ligand stereoelectronic effects. Work functions decreased from 4.85 eV (control) to 4.72 eV (TFPmA) (Supplementary Fig. 23 and Supplementary Table 2), correlating with charge redistribution by the strong surface interaction/dipole of N-heteroaromatic motifs^16,36^. This trend reflects flattened surface energy undulation, consistent with the reduced spatial potential fluctuations observed by KPFM, which mitigates interfacial charge recombination through reduced electrostatic disorder. Concomitantly, the perovskite conduction band minimum, which was derived from the UPS-determined valence band maximum and the optical bandgap^22,37^ (Supplementary Figs. 23–25 and Supplementary Table 2), shifted downward from −4.11 eV (control) to −4.21 eV (TFPmA) (Fig. 3e), thereby narrowing the energy offset with C_60_ (−4.24 eV) from 0.13 to 0.03 eV. Molecular orbital calculations and differential charge–density maps indicated that ligand–perovskite multicentre interactions induced interfacial charge redistribution, which in turn promoted interfacial energy-level realignment (Supplementary Figs. 26 and 27 and Supplementary Table 3). The sub-0.1 eV energy offset at the perovskite/TFPmA/C_60_ interface drives the energy barrier to the theoretical minimum, enabling quasi-ideal charge extraction across the heterojunction.

Quasi-Fermi level splitting (QFLS) analysis for FTO/4PADCB/perovskite/ligands samples quantified a hierarchy of perovskite/ligands interfacial defect suppression, showing a monotonic increase from 1.175 ± 0.012 eV (control, without ligands) to 1.236 ± 0.009 eV (TFPmA) (Fig. 3f). This 61-meV enhancement, enabled by stereoelectronic manipulation, arises from the above observed synergistic interfacial mechanisms: topographic defect healing through multicentre interactions by means of Pb–N coordination, delocalized electronic screening mediated by Pb–I–π interactions, a reduction in the interfacial energetic barrier^13,24^ and an increase in molecular dipole moment that reshapes the local electrostatic landscape^38,39^.

## Ligands anchoring and charge dynamics

**Fig. 4:**
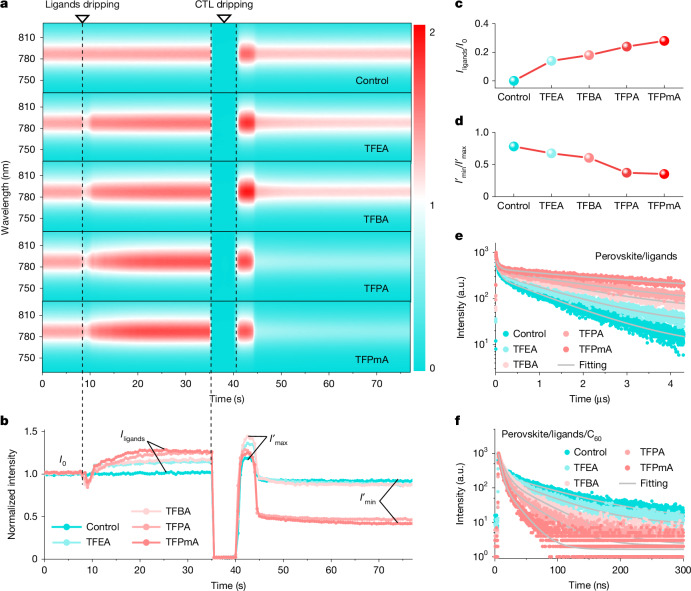
In situ passivation and carrier behaviour. **a**, In situ PL spectroscopy evolution of the FAPbI_3_-based perovskite surface during ligands and the consequent CTL spin-coating processes. **b**, Normalized PL intensity extracted from in situ PL evolution maps. **c**, The ratio of the PL intensity before (*I*_0_) and after ligands modification (*I*_ligands_). **d**, The ratio of the PL intensity before (*I*′_max_) and after CTL spin coating (*I*′_min_). **e**, TRPL curves of control and ligands-treated films with glass/perovskite/ligands structure. **f**, TRPL curves of control and ligands-treated films with glass/perovskite/ligands/C_60_ structure. a.u., arbitrary units. Source data

The ligands anchoring and charges dynamics at the perovskite/CTL interface during solution processing were investigated using in situ photoluminescence (PL) spectroscopy with a 405-nm laser (see details in Methods). We spin-coated the ligand solution onto the FAPbI_3_-based perovskite film and then drop-cast a solution of [6,6]-phenyl-C_61_-butyric acid methyl ester (PCBM) instead of insoluble C_60_ to form the CTL (Supplementary Fig. 28). Figure 4a shows two-dimensional PL intensity evolution maps recorded with and without surface ligands and Fig. 4b traces the evolution of the 788-nm emission peak over time (extracted PL spectra at 5, 25, 42, and 60 s are given in Supplementary Figs. 29–32).

On contact with the ligand solution, the PL intensity showed a two-stage anchoring process. In the initial rapid stage (less than about 3 s), PL increased by approximately 18 % relative to the neat perovskite (control), reflecting immediate ligand adsorbing at the perovskite surface in solution. A subsequent, slower stage (about 3–20 s) driven by solvent evaporation then ensued. The PL intensity further increased (up to 28% for TFPmA; Fig. 4c), consistent with continuing defect passivation that may involve Pb–N coordination and Pb–I–π interactions^12,40^.

On PCBM addition, the PL intensity exhibited a biphasic decay, with an initial rapid quenching phase (at about 42.5–45 s) from maximum value (*I*′_max_), followed by a slower decrease until stabilization (>45 s). The sub-2.5 s quenching arises from ultrafast electron transfer from perovskite to CTL during film deposition, even in the presence of residual solvent. This kinetic profile ended on complete solvent evaporation and PCBM film solidification, marking the conclusion of interfacial charge-extraction dynamics. Notably, TFPmA-modified films retained only 35% of their maximum PL intensity—versus 78% retention in the unmodified control (Fig. 4d)—indicating enhanced charge-extraction efficiency at the perovskite/ligand/CTL interface with a planar adsorption topology. This experimental outcome corroborated DFT predictions (Fig. 1e) that planar adsorption topology increases electrostatic coupling and shortens charge transport distance across the perovskite/ligands/CTL interface, thereby facilitating more efficient charge extraction.

Time-resolved photoluminescence (TRPL) revealed the dual role of ligand adsorption topology in simultaneously passivating defects and enabling ultrafast charge extraction. In perovskite/ligands architectures, TFPmA-modified films exhibited a 195% prolonged average carrier lifetime (3.19 μs versus control 1.08 μs; Fig. 4e and Supplementary Table 4). This defect suppression hierarchy—scaling with N-heteroaromatic motifs—arises from multicentre interactions locking undercoordinated Pb^2+^ sites and I^−^ vacancies. Notably, C_60_ integration inverted this trend: TFPmA-treated devices showed an 83% shortened carrier lifetime (11.0 ns versus control 65.9 ns; Fig. 4f and Supplementary Table 5), indicative of improved charge-extraction efficiency enabled by adsorption-topology-induced interfacial dipole alignment. The lifetime reduction efficacy followed the order TFPmA > TFPA > TFBA > TFEA (16.7% → 69.0%), mirroring DFT-predicted charge transport distance compression by roughly 37%. Spatially resolved TRPL mapping provided complementary insights. The ligands-treated films showed more homogeneous distributions than the control film for the TFEA, TFBA, TFPA and TFPmA (Supplementary Figs. 33 and 34), verifying increased spatial consistence, which aligns with nano-infrared observations (Fig. 3d). Collectively, this duality—lifetime extension in passivated systems versus acceleration in extraction-optimized interfaces—demonstrates efficient interfacial carrier management through stereoelectronic ligand regulation.

Mott–Schottky analysis quantified enhanced built-in potential (*V*_bi_) from 1.098 V (control) to 1.199 V (TFPmA) (Supplementary Fig. 35), in line with the improvement in charge-separation efficiency suggested by the QFLS data. Electrochemical impedance spectroscopy revealed ligand-dependent interface optimization: series resistance (*R*_s_) decreased by 78% (65.8 → 14.4 Ω), whereas recombination impedance (*R*_rec_) increased 8.9-fold (3,314 → 29,690 Ω) (Supplementary Fig. 36 and Supplementary Table 6), demonstrating suppression of interfacial recombination, which originates from N-heteroaromatic motif contribution including contact-related morphological optimization and multicentre coordination-induced electronic microenvironment enhancement^40,41^.

## Device performance and stability

**Fig. 5:**
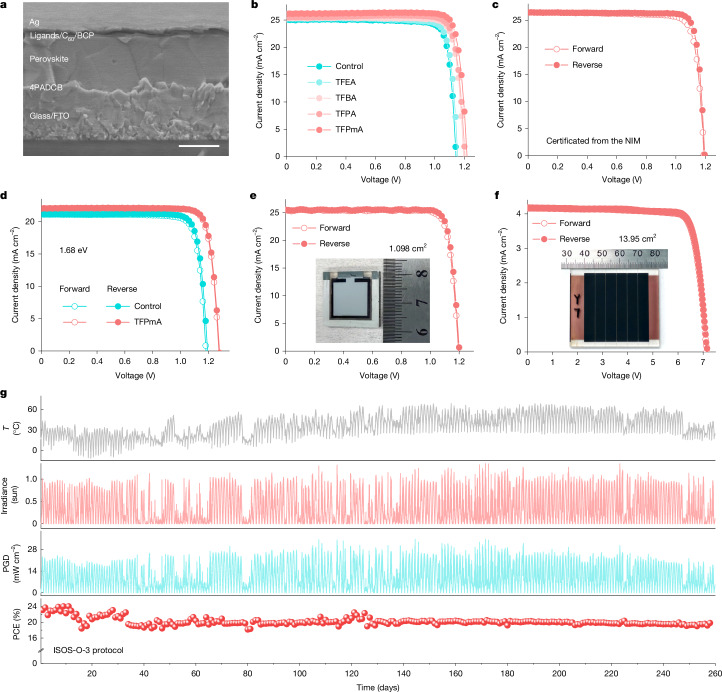
Device performance and stability. **a**, Cross-sectional structural image of the PSC. Scale bar, 500 nm. **b**, *J*–*V* curves of the control and ligands-treated devices. **c**, Certified photovoltaic performance of devices obtained at the NIM. **d**, *J*–*V* curves of wide-bandgap (1.68 eV) devices for control and TFPmA-treated devices. **e**, *J*–*V* curves of 1.098-cm^2^ devices for TFPmA-treated solar cells. **f**, *J*–*V* curves of the TFPmA-treated module devices. **g**, The real-time field testing of a TFPmA-treated module under actual outdoor operating conditions for 258 days. PGD, plane of array global irradiance. Source data

By using stereoelectronic ligand manipulation, we fabricated p-i-n PSCs (FTO/4PADCB/perovskite (FAPbI_3_-based)/ligands/C_60_/BCP/Ag, 0.074 cm^2^ cell; Fig. 5a), achieving PCEs of 24.26%, 24.65%, 25.98%, 26.37% and 27.58% for devices without (control) and with TFEA, TFBA, TFPA and TFPmA modifications (Fig. 5b), respectively. Owing to the presence of hysteresis, all efficiency parameters were extracted from reverse-scan *J*–*V* curves unless otherwise stated. Ligand-modified devices exhibited progressively reduced hysteresis indices (5.9% → 2.1%) (Supplementary Fig. 37 and Supplementary Table 7). TFPmA-based cells delivered *V*_OC_ of 1.218 V, *J*_SC_ of 26.13 mA cm^−2^ and fill factor (FF) of 86.69%, with a hysteresis index of 2.1%. We sent the best-performing device to an accredited photovoltaics laboratory (the PV Metrology Lab of the National Institute of Metrology (NIM), China) for third-party certification and obtained a reverse-scan efficiency of 27.41% with stabilized power output efficiency of 26.85% (Fig. 5c and Supplementary Figs. 38–41). The external quantum efficiency integrated currents agree with *J*–*V* measurements (variation < 3.2%) and the calculated bandgap of 1.556 eV confirmed a voltage loss of only 0.338 V (Supplementary Fig. 42). Statistical analysis of 20 independent devices confirmed excellent reproducibility of the ligand modification strategy (Supplementary Fig. 43 and Supplementary Table 8). The strategy is readily applicable to wide-bandgap perovskite systems (1.68 eV), yielding 24.04% PCE (certified at 23.04%; Fig. 5d, Supplementary Figs. 44 and 45 and Supplementary Table 9). Unencapsulated devices retained 90% of the initial PCE after 1,056 h at 85 °C (ISOS-D-2) and 2,190 h maximum power point tracking (ISOS-L-1) (Supplementary Figs. 46 and 47), delivering excellent operational stability.

Building on stereoelectronic manipulation, we demonstrated scalable device fabrication using TFPmA interfacial modification. In 1.098-cm^2^ single-junction cells, we achieved a champion PCE of 26.40% (certified at 25.62%; Fig. 5e and Supplementary Figs. 48 and 49) with *V*_OC_ of 1.202 V, *J*_SC_ of 25.54 mA cm^−2^ and FF of 86.04%. Extending the active area to 13.95 cm^2^, modules achieved a champion PCE of 24.74% with *V*_OC_ of 7.160 V, *J*_SC_ of 4.18 mA cm^−2^ and FF of 82.73% (Fig. 5f and Supplementary Table 10). These results underscore the compatibility of planar-adsorbed ligands with scalable fabrication and their ability to preserve uniform, high-performance interfaces over large areas.

We performed real-time field testing of our large-area perovskite modules under actual outdoor operating conditions to investigate interfacial stability (Fig. 5g and Supplementary Figs. 50 and 51). Subjected to 258 days of continuous operation with temperature cycling (−11.5 to 68.9 °C) and irradiance fluctuations (0–1.39 sun; plane of array global irradiance of 0–34.7 mW cm^−^^2^), the TFPmA-treated module exhibited only a 14.2% loss (from 23.10% to 19.82% PCEs). By sharp contrast, the control module degraded by 24.6% (from 21.03% to 15.85% PCEs) after only 27 days of outdoor testing (Supplementary Fig. 52). The results from ISOS-D-2 testing, ISOS-L-1 testing and real-time outdoor field testing collectively demonstrate that stereoelectronic manipulation of ligands not only minimizes energy-loss pathways at the perovskite/CTL interface but also endows devices with notable operation robustness at practical operating conditions, which would provide a compelling technical foundation for reliable large-scale perovskite photovoltaics deployment.

## Conclusions

This work demonstrates that the ligand adsorption topology on perovskite surfaces, governed by ligand–perovskite interactions, enables minimization of interfacial energy losses. Introducing N-heteroaromatic motifs into ligands triggers a stereoelectronic reconfiguration from vertical to planar alignment through synergistic Pb–N coordination and Pb–I–π interactions. This topology change concurrently annihilates defects while sustaining sub-nanometre charge transport channels, achieving a stabilized power output of 26.85%. Notably, the strategy is both bandgap-universal and readily scalable, delivering high efficiencies for wide-bandgap (1.68 eV) devices and large-area cells and modules. Extended outdoor validation reveals an interfacial robustness during 258 days of real-time field testing. This atomic-scale interface control model provides new design principles for minimizing interfacial energy losses to advance perovskite photovoltaics towards their theoretical efficiency limit while ensuring scalability and durability.

## Methods

### Materials

FAPbI_3_ single-crystal powder (99.99%) was purchased from Perov-Opto Co., Ltd. Pb (II) iodide (PbI_2_, 99.999%) was purchased from TCI. Methylammonium iodide (MAI, 99.5%) and methylammonium chloride (MACl, 99.5%) were purchased from GreatCell Solar. Caesium iodide (CsI, 99.99%), PCBM and Pb (II) bromide (PbBr_2_, 99.99%) were purchased from Advanced Election Technology Co., Ltd. C_60_, (4-(7H-dibenzo[c,g]carbazol-7-yl)butyl)phosphonic acid (4PADCB) and Pb (II) chloride (PbCl_2_, 99.99%) were purchased from Xi’an Yuri Solar Co., Ltd. 2,2,2-Trifluoroacetimidamide hydrochloride (TFEA, 95%) and 4-(trifluoromethyl)benzimidamide hydrochloride (TFBA, 97%) were purchased from Bide Pharmatech Co., Ltd. 6-(Trifluoromethyl)nicotinimidamide hydrochloride (TFPA, 99%) and 2-(trifluoromethyl)pyrimidine-5-carboximidamide hydrochloride (TFPmA, 95%) were purchased from Leyan. Chlorobenzene (CB) (anhydrous, 99.8%) and isopropanol (IPA) (anhydrous, 99.5%) were purchased from Sinopharm Chemical Reagent Co., Ltd. N,N-dimethylformamide (DMF) (anhydrous, 99.8%), dimethyl sulfoxide (DMSO) (anhydrous, ≥99.9%) and 2,9-dimethyl-4,7-diphenyl-1,10-phenanthroline (BCP) were purchased from Alfa Aesar. Nickel acetylacetonate dihydrate (Ni(acac)_2_·2H_2_O, 99%) was purchased from Shanghai Aladdin. All of the chemical materials and reagents were used as received without further purification.

### Perovskite precursor solution

Normal-bandgap FAPbI_3_-based perovskite (1.56 eV) solution (1.5 M, FA_0.85_MA_0.1_Cs_0.05_Pb(I_0.98_Br_0.02_)_3_) was prepared by mixing 807 mg FAPbI_3_ single-crystal powder, 138 mg PbI_2_, 23.9 mg MAI, 19.5 mg CsI, 16.5 mg PbBr_2_ and 12.3 mg MACl in the 1 ml mixed solvent of DMF and DMSO (volume ratio: 4:1) and stirred for 2 h. Wide-bandgap perovskite (1.68 eV) solution (1.3 M, Cs_0.22_FA_0.78_Pb(I_0.85_Br_0.15_)_3_) was prepared by mixing 641.8 mg FAPbI_3_ single-crystal powder, 20.3 mg PbI_2_, 74.3 mg CsI and 107.3 mg PbBr_2_ in mixed solvent of DMF and DMSO (volume ratio: 4:1) and stirred for 5 h. 5 mol% MACl (4.4 mg) and PbCl_2_ (18.1 mg) with molar ratio were added to increase the crystallinity. Mini-modules perovskite: 1.2 M FA_0.9_Cs_0.1_PbI_3_ was prepared by mixing 31.2 mg CsI, 55.3 mg PbI_2_ and 683.6 mg FAPbI_3_ single-crystal powder in mixed solvent of DMF and DMSO (volume ratio: 4:1) and stirred for 2 h. 15 mol% MACl (12.2 mg) and 3 mol% PbCl_2_ (10.0 mg) with molar ratio were added to increase the crystallinity. All of the perovskite precursor solutions were filtered by 0.22-μm polytetrafluoroethylene filters before use.

### Fabrication of PSCs

For the normal-bandgap PSCs, the pre-patterned FTO glass substrates (2.5 × 2.5 cm, 10 Ω per square) were cleaned by ultrasonication in detergent and ultrapure water (four times) for 30 min, respectively. The cleaned substrates were dried under N_2_ flow and treated with ultraviolet ozone for 15 min and then immediately transferred into an N_2_ glovebox (oxygen content less than 0.5 ppm and water content less than 0.1 ppm) in preparation for the deposition process. The hole transport layer was prepared with 4PADCB (0.5 mg ml^−1^) dissolved in anhydrous ethanol and spin-coated onto the above-prepared FTO substrates and then annealed at 100 °C for 10 min. The deposition of the perovskite layer was carried out after the substrates were cooled to room temperature. The perovskite layer was deposited by one-step spin-coating-filtered precursor solution at 1,000 rpm for 10 s and 4,000 rpm for 40 s, in which 300 μl ethyl acetate was quickly dropped onto the centre of the spinning substrate 10 s before the end. The film was immediately annealed at 100 °C for 30 min. After cooling down to room temperature, the ligand engineering strategy was executed. Specifically, 1 mg ml^−^^1^ TFEA/TFBA/TFPA/TFPmA solution (in IPA solvent) was drop-cast on the spinning perovskite film at 3,000 rpm for 30 s without further annealing. Next, C_60_ (20 nm) and BCP (5 nm) were thermally evaporated on the perovskite films under a high vacuum of <1 × 10^−4^ Pa. Finally, Ag electrodes (120 nm) were thermally evaporated under a vacuum of <5 × 10^−4^ Pa.

For the wide-bandgap PSCs, the substrate preparation process is the same as above. After that, Cs_0.22_FA_0.78_Pb(I_0.85_Br_0.15_)_3_ perovskite precursor solution was spin-coated on the FTO/4PADCB substrates at 4,000 rpm for 40 s. During the last 10 s, 200 μl ethyl acetate was dropped as the antisolvent. The film was immediately annealed at 100 °C for 50 min. After cooling down to room temperature, 1 mg ml^−1^ TFPmA solution was drop-cast on the spinning perovskite film surface at 3,000 rpm for 30 s without further annealing. Finally, C_60_ (20 nm)/BCP (5 nm)/Ag (120 nm) were sequentially deposited on top of the perovskite by thermal evaporation.

### Fabrication of perovskite solar modules

The fabrication of perovskite mini-modules began with the P1 laser (nanosecond green laser, scan speed = 1.0 m s^−1^, laser power = 1.5 W and repetition rate = 350 kHz) scribing process to isolate the patterned FTO film. The 6 × 6-cm^2^ FTO substrates were sequentially cleaned with detergent and ultrapure water (four times) for 30 min, respectively. Then the cleaned substrates were dried under N_2_ flow and treated with ultraviolet ozone for 15 min before use. A NiO_*x*_ hole transport layer was then deposited by spin-coating a freshly prepared precursor solution of nickel acetylacetonate dihydrate (0.1 mmol ml^−1^) dissolved in ethanol and ethanolamine (1:0.005, v/v), followed by thermal annealing in air. After cooling, a 1.5 mg ml^−1^ PTAA solution in chlorobenzene was spin-coated and dried. The perovskite photoactive layer was subsequently deposited by spin-coating precursor solution, followed by thermal annealing to form a dense and uniform crystalline film. After that, the interface TFPmA layers were drop-cast at spinning perovskite layers at 3,000 rpm for 30 s without further annealing. All wet processing steps are conducted in a Class 1000 cleanroom at about 23–25 °C with relative humidity maintained below 45%.

The perovskite-coated substrates were then transferred to a vacuum deposition system for sequential thermal evaporation of 20 nm of C_60_ and 5 nm of BCP. This was followed by the deposition of a 7-nm SnO_2_ layer using spatial atomic layer deposition at 90 °C, with tetrakis(dimethylamino)tin and water as precursors. The P2 laser (picosecond green laser, scan speed = 0.5 m s^−1^, laser power = 0.2 W and repetition rate = 200 kHz) scribing process was subsequently performed in ambient atmosphere to selectively remove all functional layers above the FTO. A 150-nm Ag back electrode was then deposited by thermal evaporation, followed by P3 laser (picosecond green laser, scan speed = 0.5 m s^−1^, laser power = 0.15 W and repetition rate = 200 kHz) scribing to define the sub-cell interconnections. Conductive copper tapes with Sn95/Pb5 coating (140 mm in length) are applied to connect the electrode terminals. For encapsulation, a 10 × 10-cm^2^ tempered glass sheet is used as the bottom substrate. A butyl rubber sealant was applied along the edges and a PO-based encapsulant film is placed at the centre. The 6 × 6-cm^2^ perovskite mini-module was then aligned and placed on top, with the conductive tape fixed to the surrounding butyl sealant. A second layer of butyl rubber was applied to cover the conductive tape, followed by placement of a PO film over the back side of the device. A top 10 × 10-cm^2^ tempered glass sheet is then added to complete the stack. The entire assembly was laminated under vacuum at 90 kPa and 120 °C for 15 min. After lamination, the module was ready for *J*–*V* testing.

### Film characterization

Scanning electron microscopy (SU-8020, Hitachi FE-SEM) was used to characterize the surface morphology and cross-sectional view of the films. Atomic force microscopy and KPFM images were obtained from the Bruker Dimension Icon instrument. The contact potential difference (CPD) measured by KPFM is related to the work function difference between the tip and the sample according to: CPD = (*Φ*_tip_ − *Φ*_sample_)/*e*, in which *Φ*_tip_ and *Φ*_sample_ are the work functions of the tip and sample, respectively, and *e* is the elementary charge. Owing to the uncertainty in the absolute tip work function, KPFM is used here to evaluate the relative surface potential distribution and spatial heterogeneity. Statistical parameters, including the mean absolute deviation (Ra) and standard deviation (*σ*), are extracted from pixel-by-pixel CPD data. X-ray diffraction (2700BH, Dandong Haoyuan) patterns were measured with a diffractometer using Cu Kα (*λ* = 1.54 Å) radiation. Ultraviolet–visible spectrophotometry (Hitachi-UH4150) was performed to obtain the ultraviolet–visible spectra of perovskite films and the wavelength detection range was set to 400–900 nm. PL and TRPL spectra were measured with a PicoQuant FluoQuant 300 and a laser with filter wavelength 510 nm. In situ PL spectra were obtained using a short-wavelength laser diode (405 nm), which aims to detect the surface region of films to minimize interference from the bulk material, coupled with a visible-range spectrometer under a dark environment at 25 °C and 15–20% humidity. Moreover, the initial state of all perovskite films measured was identical. UPS and XPS spectra were obtained using a photoelectron spectrometer (ESCALAB 250Xi, Thermo Fisher Scientific). Unless otherwise specified, all characterizations were performed on FAPbI_3_-based perovskite (FA_0.85_MA_0.1_Cs_0.05_Pb(I_0.98_Br_0.02_)_3_) films fabricated following our standard one-step spin-coating protocol. ^1^H-NMR spectra were measured by the JNM-ECZ400R/S1 (JEOL) apparatus. FTIR spectra were collected using a Bruker Tensor ΙΙ spectrometer equipped with a KBr beam splitter.

### Solar cell characterization

*J*–*V* and *I*–*T* curves were measured with a source meter (Keithley 2450) and solar simulator (SAN-EI ELECTRIC DM-50S1) under AM1.5G, which was adjusted using a standard silicon cell. The voltage range for forward and reverse scans were 1.4 to −0.1 V, with a step of 0.02 V and a delay time of 20 ms. The contact area of the cell is 0.12 cm^2^ and the illumination area is 0.074 cm^2^. All devices were tested at room temperature (about 25 °C) in an N_2_ glovebox. The external quantum efficiency curves were performed on a QE-R system and the light source is a 300-W Xe lamp (QE-R, EnliTech). The electrochemical impedance spectroscopy curves were measured with an electrochemical workstation (ModuLab XM). The M-S measurements were performed on an electrochemical workstation (ModuLab XM) and the frequency was set to 10 kHz and the scan voltage range was 0–1.5 V. The device structure was FTO/4PADCB/Perovskite/ligands/C_60_/BCP/Ag. The built-in potential (*V*_bi_) was obtained from the linear fitting derived from the equation ${C}_{{\rm{dl}}}=\sqrt{\frac{q\varepsilon {\varepsilon }_{0}N}{2({V}_{{\rm{bi}}}-V)}}$, in which *q* is the elementary charge, *ε* is the relative dielectric constant of the perovskite material, *ε*_0_ is the vacuum permittivity and *N* is the carrier density.

### Outdoor real-time field testing of perovskite solar modules

The outdoor stability measurements were conducted in Hangzhou, China, from February 2025 to October 2025 for 258 days for the TFPmA-treated module and from October to November 2025 for 27 days for the control module. All modules were subjected to continuous operation under daylight illumination and heat cycles from day to night. The temperature sensor (type: PT100) is placed on the back of the modules, which can reach a peak temperature of 68.9 °C during the daytime and a minimum of about −11.5 °C at night. On sunny days, the light intensity reaches a maximum of 1.39 sun. All data are monitored in real time and recorded on a computer.

### Reporting summary

Further information on research design is available in the Nature Portfolio Reporting Summary linked to this article.

## Online content

Any methods, additional references, Nature Portfolio reporting summaries, source data, extended data, supplementary information, acknowledgements, peer review information; details of author contributions and competing interests; and statements of data and code availability are available at 10.1038/s41586-026-10626-0.

## Supplementary information

Supplementary Information This file contains Supplementary Notes 1–3, Supplementary Figs. 1–52, Supplementary Tables 1–10 and Supplementary references.

Reporting Summary

Peer Review File

## Source data

Source Data Figs. 1, 2, 3, 4, 5

## Data Availability

The data that support the findings of this study are available in the article and its Supplementary Information/Source Data file. Source data are provided with this paper.
