# Supplementary material for: Stereoelectronic manipulation of ligands for perovskite solar cells
Source: Nature. 2026 May 13;654(8119):660–7. doi: 10.1038/s41586-026-10626-0 (PMC13275290; doi:10.1038/s41586-026-10626-0)
Supplement: Supplementary file 1 — This file contains Supplementary Notes 1–3, Supplementary Figs. 1–52, Supplementary Tables 1–10 and Supplementary references. [file 41586_2026_10626_MOESM1_ESM.pdf]

nature portfolio

https://doi.org/10.1038/s41586-026-10626-0

Supplementary information

Stereoelectronic manipulation of ligands for perovskite solar cells

In the format provided by the authors and unedited

Supplementary Information for

Stereoelectronic manipulation of ligands for perovskite solar cells

Yang et al.

Table of Contents:

I. Supplementary Notes

Supplementary Note 1 Theoretical calculations.

Supplementary Note 2 XPS, NMR and FTIR measurement and analysis.

Supplementary Note 3 Nano IR measurement and analysis.

II. Supplementary Figures and Tables

Supplementary Figures 1-52

Supplementary Tables 1-10

III. Supplementary references

Supplementary Notes

Supplementary Note 1

Theoretical calculations

The theoretical calculations for the dipole moment and electrostatic potential (ESP) of ligands (TFEA, TFBA, TFPA, TFPmA) were performed via the Gaussian 16 suite of programs and Gauss View package at the B3LYP-D3BJ/def2-TZVP level, respectively. The structures were characterized as a local energy minimum on the potential energy surface by verifying that all the vibrational frequencies were real. The direction of the dipole moment in the figure is from the negative charge center to the positive charge center.

All the density functional theory (DFT) calculations were performed by Vienna Ab initio Simulation Package(VASP) [1,2] within the generalized gradient approximation (GGA) using the Perdew-Burke-Ernzerhof (PBE)[3] formulation. The projected augmented wave (PAW) potentials [4,5] were used for describing the ionic cores and the plane wave cutoff energy was 500 eV. Partial occupancies of the Kohn−Sham orbitals were allowed using the Gaussian smearing method and a width of 0.05 eV. For the optimization of both geometry and lattice size, the Brillouin zone integration was performed with a 2 × 2 × 1 Gamma centered sampling.[6] The weak interaction was corrected by the DFT+D3 method.[7,8] The self-consistent calculations applied a convergence energy threshold of 10^{-5} eV. The equilibrium geometries and lattice constants were optimized with maximum stress on each atom within 0.02 eV $Å^{-1}$. The adsorption energy was calculated as: $E_{ads} = E_{(perovskite@ligands)} - E_{(perovskite)} - E_{(ligands)}$. The binding energy was calculated as $E_{(perovskite/ligands/C60)} - E_{(perovskite/ligands)} - E_{(C60)}$.

On Pb–I–terminated surfaces, the formation-energy differences ($\Delta E = E_{ads(planar)} - E_{ads(vertical)}$) for TFEA, TFBA, TFPA, and TFPmA decrease sequentially from 0.57 to 0.01, −0.18, and −0.27 eV, respectively. For TFBA, ΔE is close to zero (0.01 eV), suggesting no strong configurational preference between vertical and planar adsorption. This trend indicates a gradual thermodynamic shift in preferred adsorption topology from predominantly vertical (TFEA), to nearly non-preferential or mixed

configurations (TFBA), and further toward planar geometries (TFPA and TFPmA) as the number of N-heteroaromatic units increases.

In comparison, the calculated adsorption energies on FA–I–terminated surfaces are relatively smaller, with E_{ads}(vertical) values of −0.45, −0.65, −0.52, and −0.51 eV and E_{ads}(planar) values of −0.21, −0.46, −0.36, and −0.44 eV for TFEA, TFBA, TFPA, and TFPmA, respectively. Further analysis of the corresponding formation-energy differences (ΔE) reveals a similar decreasing trend with increasing N content, indicating that higher N incorporation progressively lowers the energetic penalty for planar adsorption. Collectively, these results suggest that increasing N heteroatom content promotes a tendency toward planar adsorption geometries across different surface terminations, thereby corroborating the central adsorption trend established in the main text.

Supplementary Note 2

XPS, NMR and FTIR measurement and analysis

XPS measurements and sample preparation. Thin films for XPS characterization were prepared using the same deposition procedures as for functional devices. Following surface ligand modification, all samples were handled entirely within a nitrogen-filled glovebox and transferred to the XPS analysis chamber via an airtight transfer vessel under continuous N_2 atmosphere. This protocol minimized exposure to ambient air, light, and heat, thereby ensuring surface integrity prior to measurement. Spectra were acquired using a monochromatic Al Kα X-ray source (1486.68 eV) with the analysis chamber base pressure maintained between 1.0×10^{-9} and 5.0×10^{-8} mbar. High-resolution scans were recorded at a pass energy of 20 eV.

NMR measurements and sample preparation. To investigate the coordination interaction between the passivating ligands (TFEA, TFBA, TFPA, and TFPmA) and PbI_2, liquid-state ^1H NMR experiments were carried out in deuterated DMSO (d_6-DMSO). Each ligand and its equimolar mixture with PbI_2 (1:1 molar ratio) were dissolved in d_6-DMSO and analyzed by ^1H NMR. Upon addition of PbI_2, distinct

upfield shifts were observed for the amidino and aromatic proton signals, indicating strong ligand–Pb^{2+} coordination. These ligand-dependent spectral changes reveal a clear hierarchy of coordination strength that correlates with the trends in defect passivation and device performance.

FTIR measurements and sample preparation. FTIR measurements were performed on KBr pellets (sample:KBr = 1:100) pressed under vacuum, with spectra acquired from 4000–400 cm^{-1} at 0.4 cm^{-1} resolution. Interaction with PbI_2 broadened the –C=N stretching vibration, with FWHM increases of 49% (TFEA), 51% (TFBA), 59% (TFPA), and 107% (TFPmA). This trend reflects stronger ligands-perovskite interactions at the presence of multicenter interactions (TFBA, TFPA, TFPmA).

Finally, a consistent coordination hierarchy among the ligands is established through multiple complementary techniques. Solution-phase ^{1}H NMR (chemical shifts of δHα and δHβ) and solid-state characterizations—including core-level shifts of Pb, I, and N in XPS and vibrational changes of key functional groups in FTIR—provide convergent evidence. Together, these results substantiate strong and multicenter ligand and Pb-I coordination at the perovskite interface.

Supplementary Note 3

Nano IR measurement and analysis

Nano IR spectra and images were collected with a home-build AFM-IR system combing AFM (Dimension Icon) from Bruker with mid IR laser (Mircat) from Daylight Solutions. The home-build AFM-IR was operated in tapping mode in air, 1Hz, 256 pts. A gold-coated probe (HQ:NSC 14, MikroMasch) was used to enhance the IR-induced thermal expansion signal in AFM-IR measurement. The AFM-IR signal was demodulated from the fundamental resonance mode of the gold-coated probe which was driven at second-order resonance frequency. The repetition frequency of the mid-infrared excitation light was the difference between the first and second resonant frequencies of the probe.

The relative signal-active fractions of the ligands on the perovskite surface were performed using the image processing software Photoshop. We used the ratio of the near red pixel area representing the distribution of ligands to the total pixels of the image to estimate the relative signal-active fractions of ligands. In this work, the total pixels of the image were 41208 and the near red area pixels representing TFEA, TFBA, TFPA and TFPmA were 3179, 10584, 13849 and 19074, respectively. And hence the corresponding relative signal-active fractions values were determined to be 7.7%, 25.7%, 33.6% and 46.3%, respectively.

Supplementary Figures

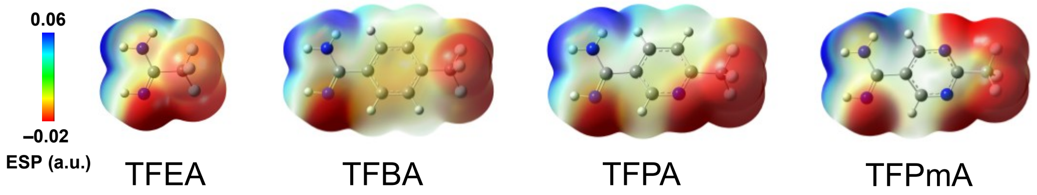

Supplementary Fig. 1| The electrostatic potential redistribution for TFEA, TFBA, TFPA and TFPmA ligands.

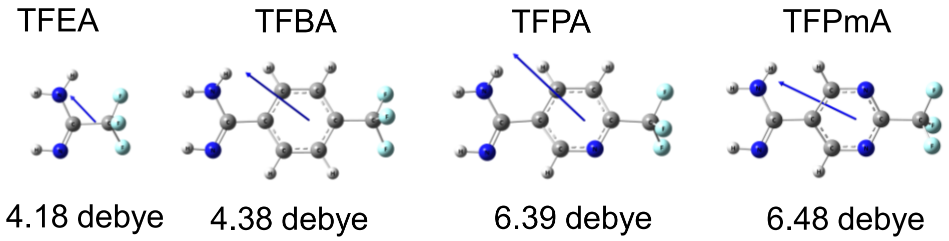

Supplementary Fig. 2| The molecular dipole moments for TFEA, TFBA, TFPA and TFPmA ligands.

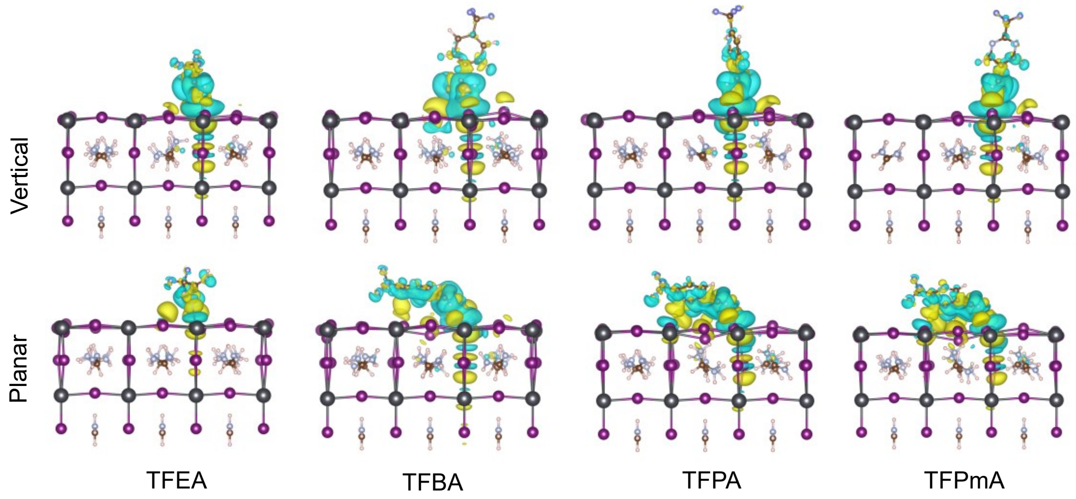

Supplementary Fig. 3| The adsorption configurations of different ligands on the Pb-I terminal of perovskites with the k-point grid to (2×2×1) and the Plane-wave cutoff to 500 eV.

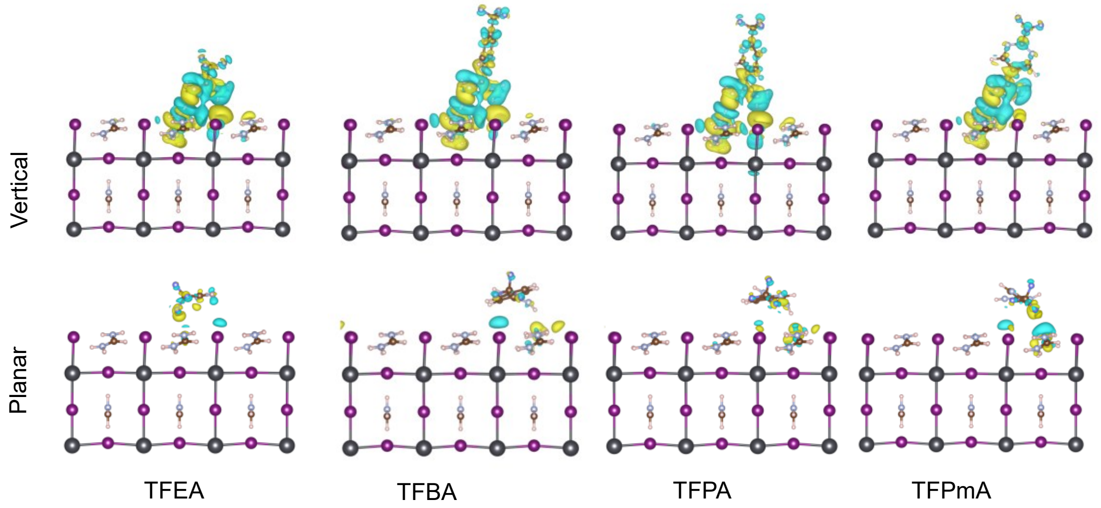

Supplementary Fig. 4| The adsorption configurations of different ligands on the FA-I terminal of perovskites with the k-point grid to (2×2×1) and the Plane-wave cutoff to 500 eV.

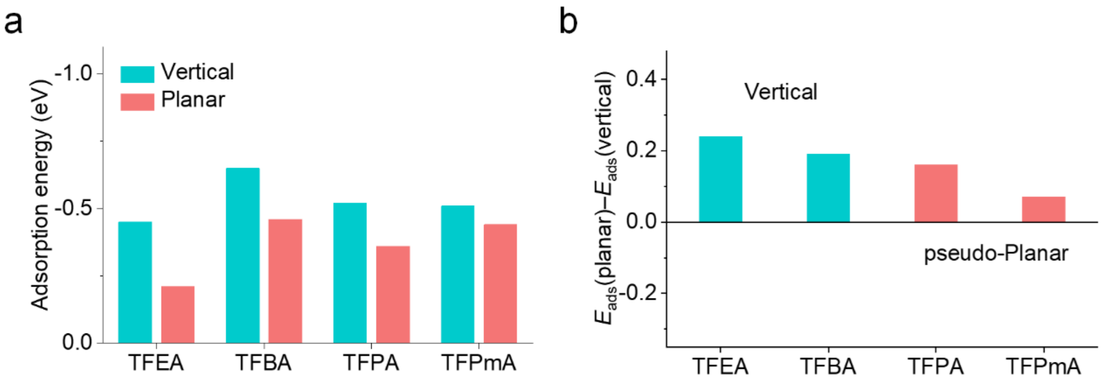

Supplementary Fig. 5| (a) The adsorption energy of different ligands on the FA-I terminal of perovskites with the k-point grid to (2×2×1) and the Plane-wave cutoff to 500 eV. (b) Formation-energy differences ($\Delta E = E_{ads}(planar) - E_{ads}(vertical)$) between vertical and planar adsorption topology for each ligand on the FA-I terminal perovskite surface.

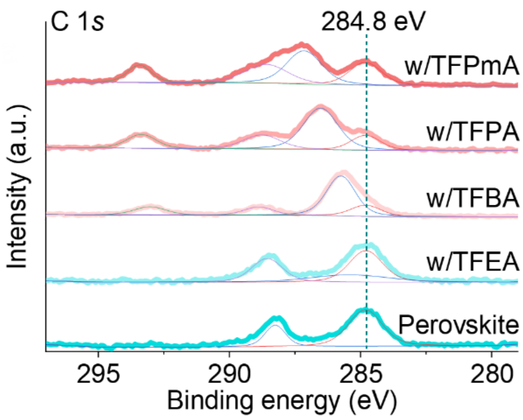

Supplementary Fig. 6| XPS spectra of C 1*s* for perovskite films treated without/with TFEA, TFBA, TFPA and TFPmA.

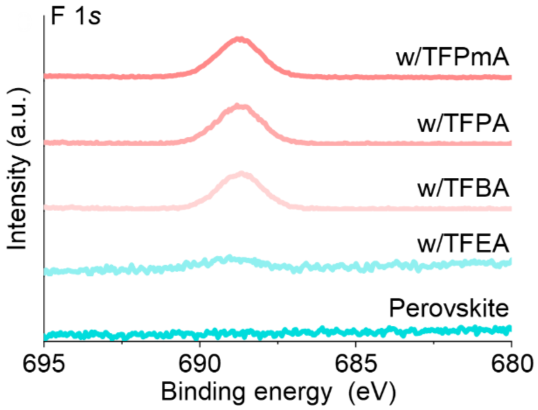

Supplementary Fig. 7| XPS spectra of F 1*s* from perovskite films treated without/with TFEA, TFBA, TFPA and TFPmA.

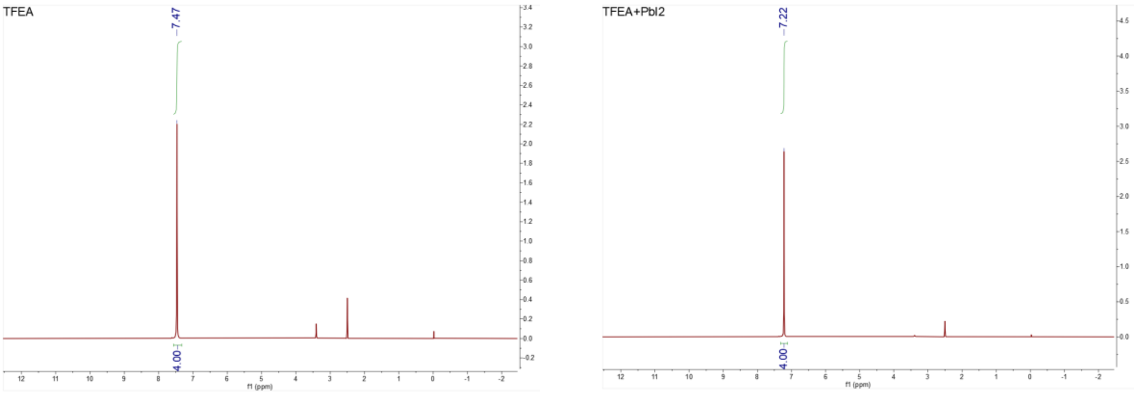

Supplementary Fig. 8| The ^{1}H-NMR spectra of TFEA and TFEA with PbI_2 (d_6-DMSO, 400 MHz, ppm).

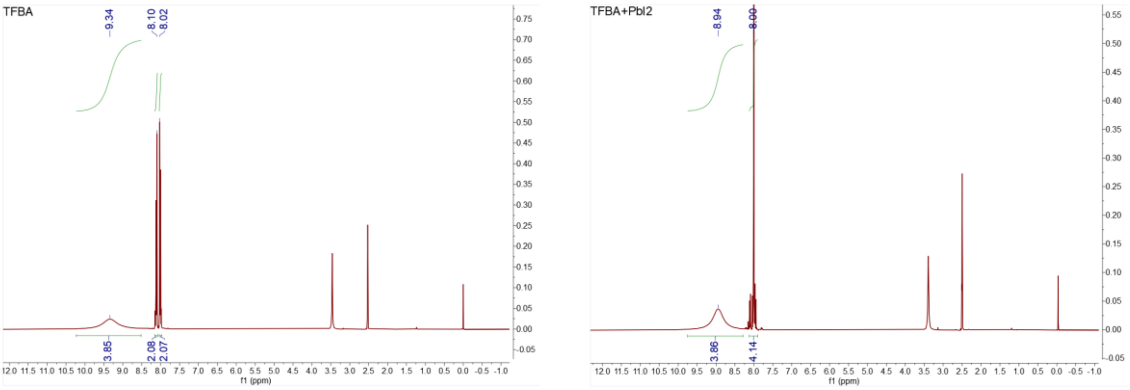

Supplementary Fig. 9| The ^{1}H-NMR spectra of TFBA and TFBA with PbI_2 (d_6-DMSO, 400 MHz, ppm).

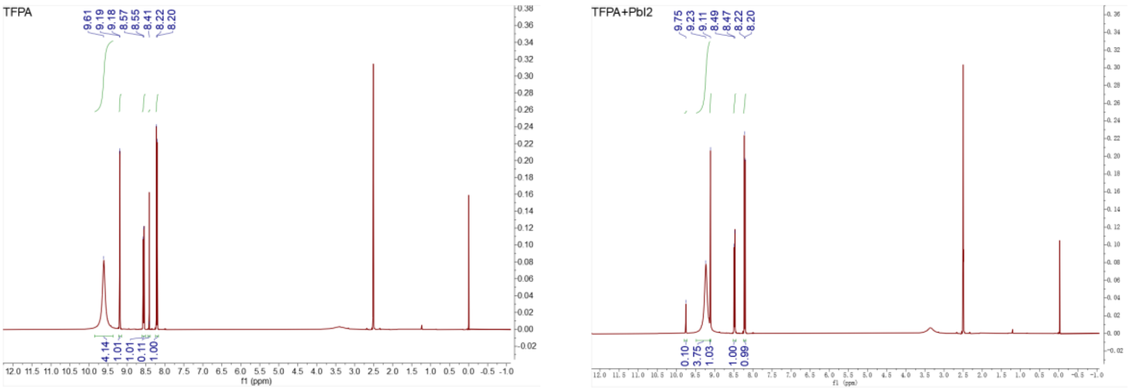

Supplementary Fig. 10| The ^{1}H-NMR spectra of TFPA and TFPA with PbI_2 (d_6-DMSO, 400 MHz, ppm).

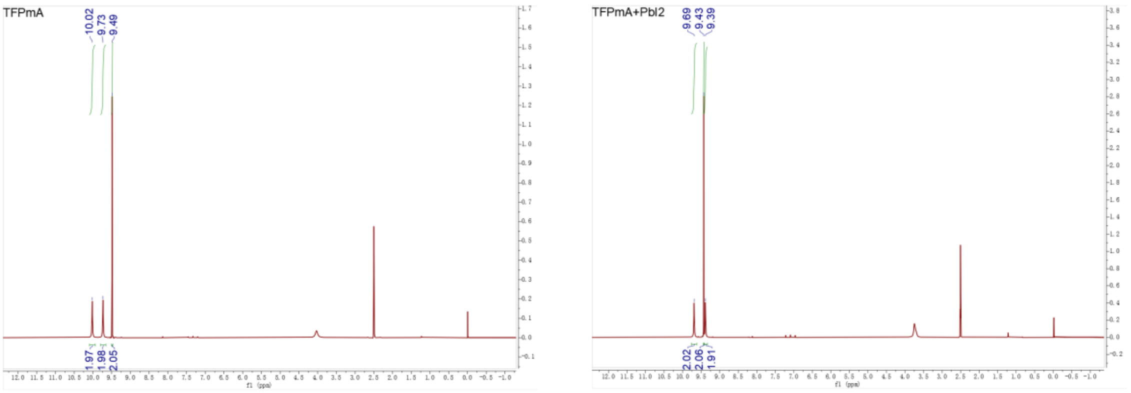

Supplementary Fig. 11| The ^{1}H-NMR spectra of TFPmA and TFPmA with PbI_2 (d_6-DMSO, 400 MHz, ppm).

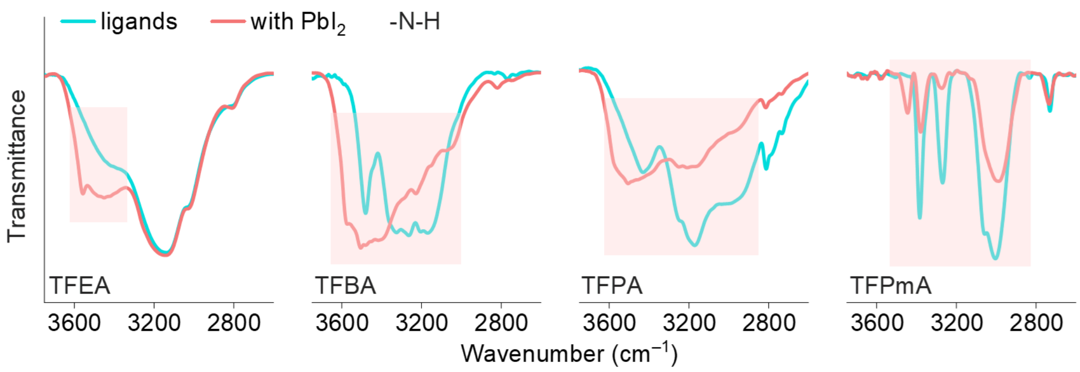

Supplementary Fig. 12| The FTIR spectra (3750~2600 cm^{-1}) of different ligands interacting with PbI_2.

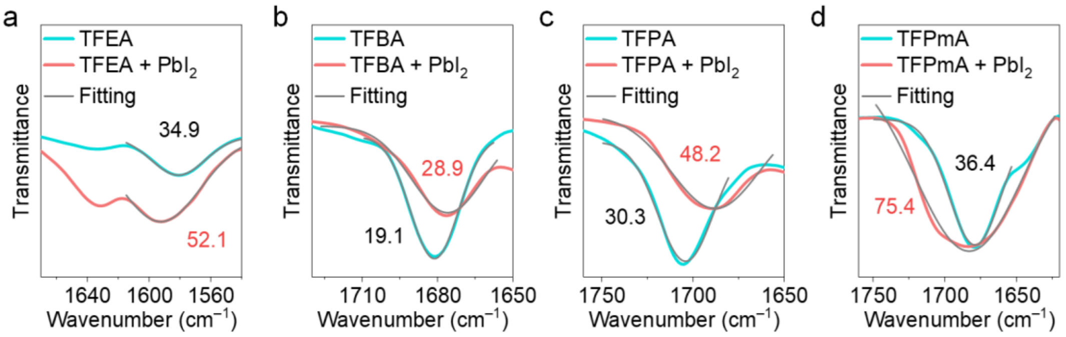

Supplementary Fig. 13| Fitting of -C=N vibrational peak extracted from the FTIR spectra of different ligands interacting with PbI_2 and corresponding full width at half-maximum (FWHM).

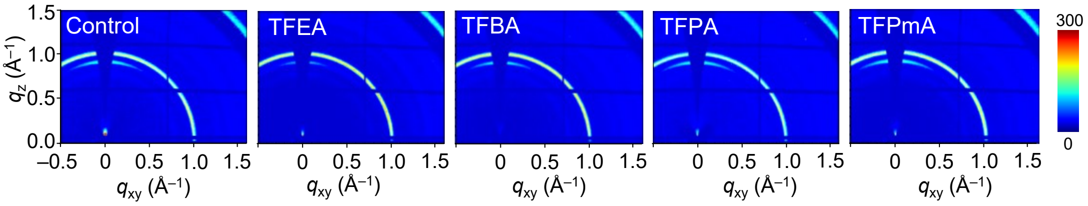

Supplementary Fig. 14| The GIWAXS patterns of the control and ligands treated perovskite films with TFEA, TFBA, TFPA and TFPmA.

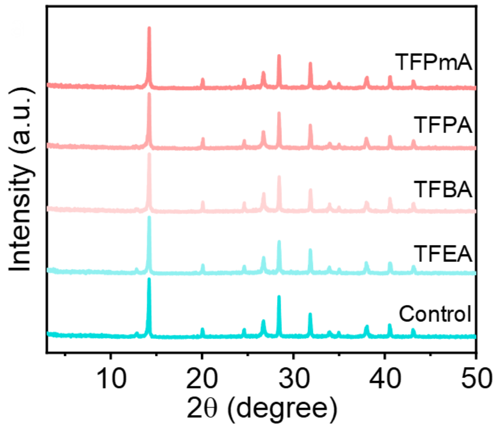

Supplementary Fig. 15| The XRD patterns for the control and ligands-treated perovskite films.

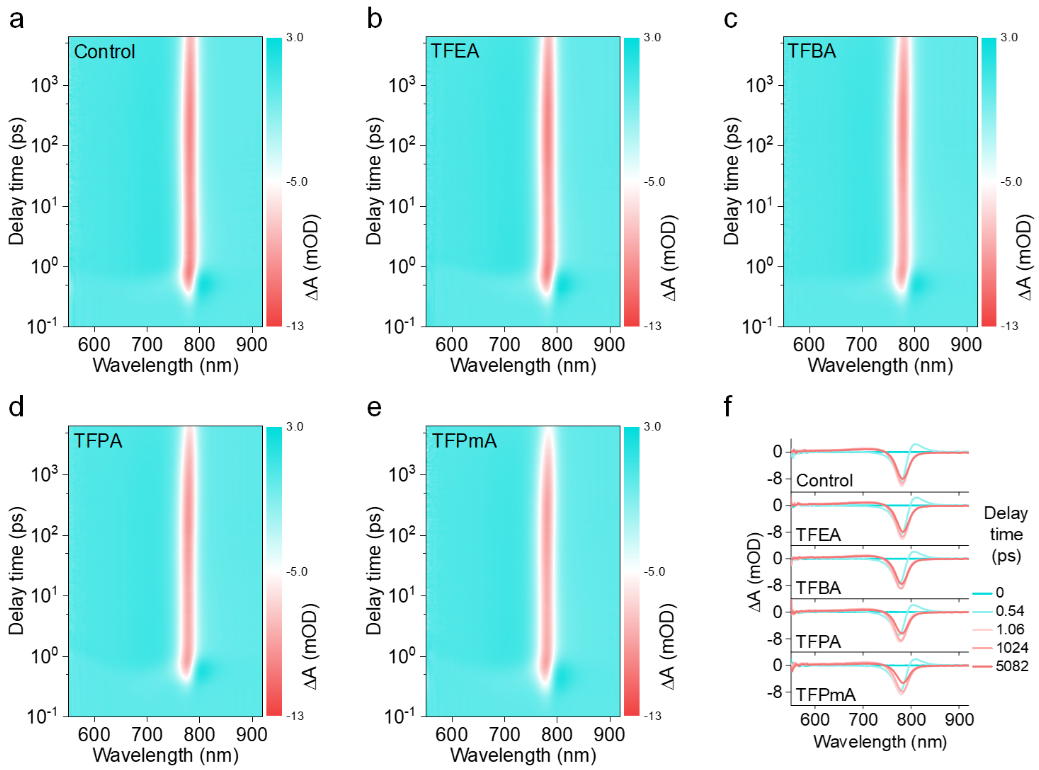

Supplementary Fig. 16| The TAS spectra of the control and ligands-treated films.

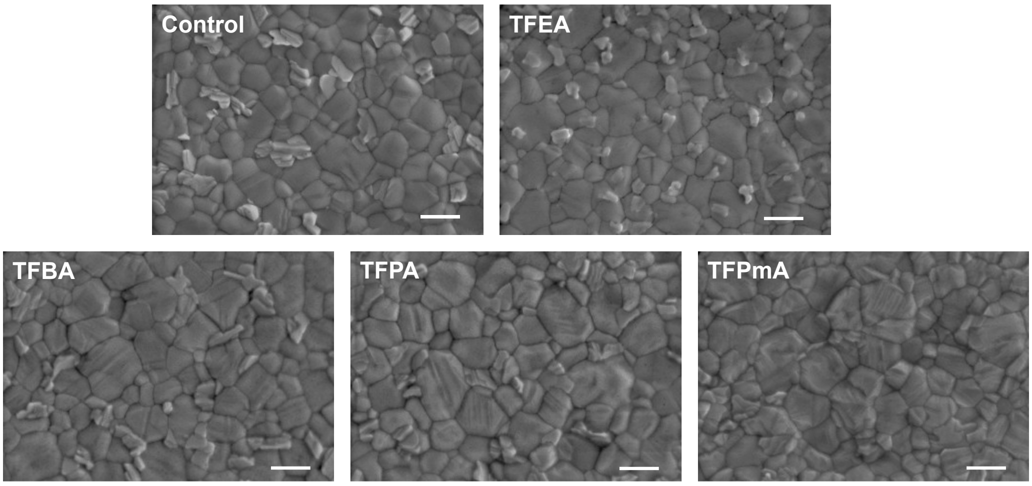

Supplementary Fig. 17| The SEM images of the control and ligands-treated perovskite films. The scale bar is 500 nm. In contrast to the control sample, there is a distinct presence of PbI_2 (Bright, irregular domains) at the grain boundaries. After ligand treatment, these domains are significantly reduced, indicating redistribution or dissipation of excess PbI_2 and a more homogeneous surface morphology.

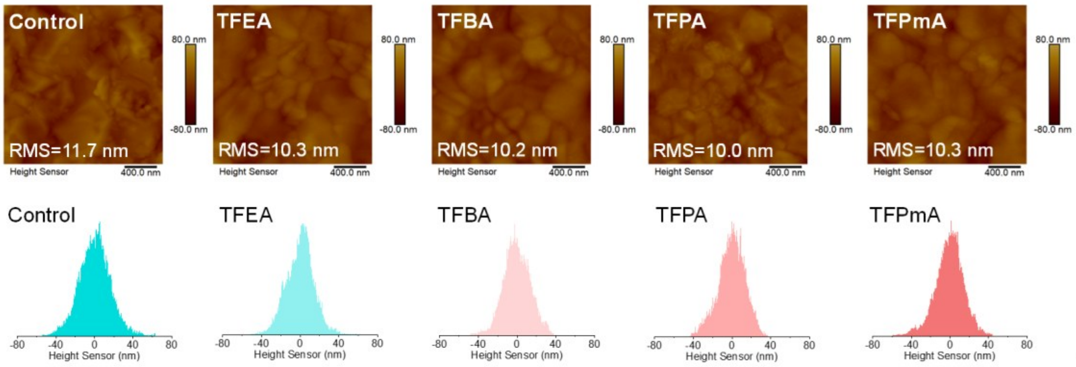

Supplementary Fig. 18| The AFM images and corresponding height sensor distribution for the control and ligands-treated perovskite films. Compared to the control sample, which had a root-mean-square (RMS) roughness of 11.7 nm, the roughness of the ligands-modified films decreased by 1.4, 1.5, 1.7, and 1.4 nm, respectively.

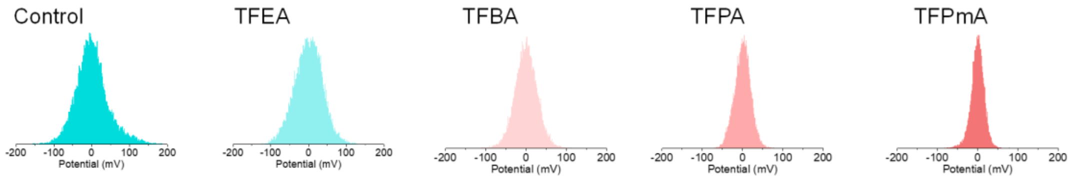

Supplementary Fig. 19| The potential distribution extracted from the KPFM test for the control and ligands-treated perovskite films. The histograms are constructed from pixel-by-pixel KPFM data and the mean absolute deviation (Ra) and standard deviation (σ) for each sample are summarized as follows: Control (Ra = 30.1 mV, σ = 39.5 mV), TFEA (Ra = 27.1 mV, σ = 33.7 mV), TFBA (Ra = 20.1 mV, σ = 25.4 mV), TFPA (Ra = 14.6 mV, σ = 18.4 mV), and TFPmA (Ra = 11.5 mV, σ = 14.8 mV). The reduced σ values indicate suppressed spatial potential fluctuations upon surface modification.

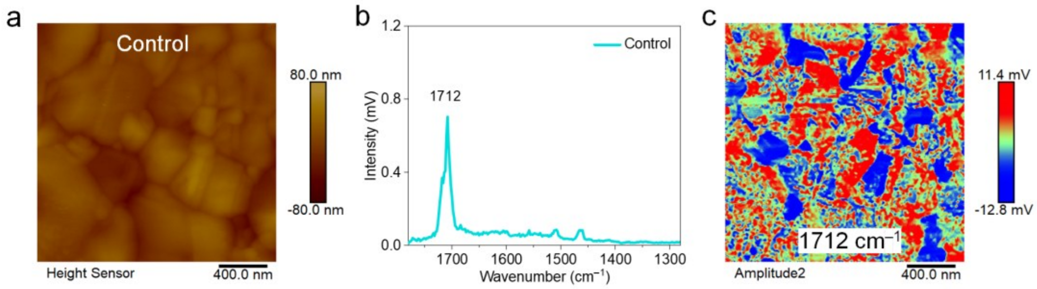

Supplementary Fig. 20| The nano-IR measurement of the control perovskite film. (a) AFM images. (b) the IR spectral. (c) nano-IR maps of film at 1712 cm^{-1}.

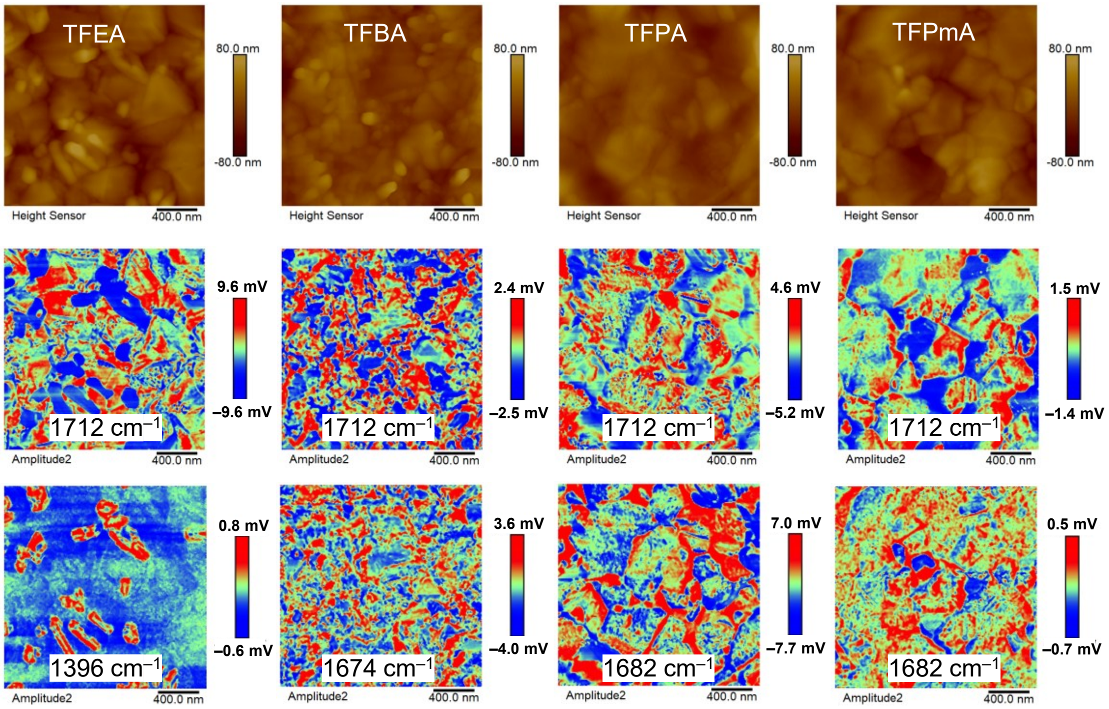

Supplementary Fig. 21| The nano-IR measurement of ligand-treated perovskite films at 1712, 1396, 1674, 1682 and 1682 cm^{-1}, respectively.

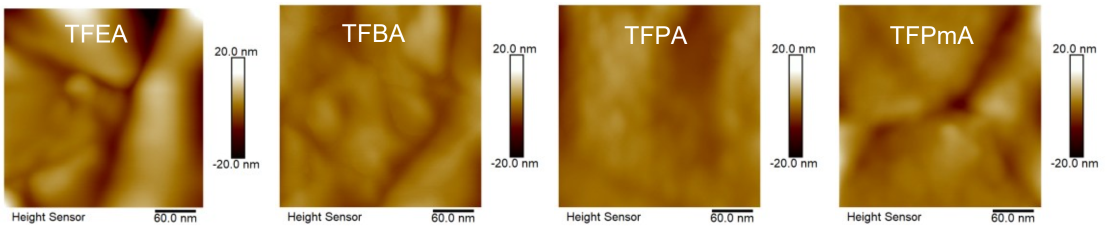

Supplementary Fig. 22| The microregion morphology of ligand-treated perovskite films for Nano-IR measurement at 1396, 1674, 1682 and 1682 cm^{-1}, respectively.

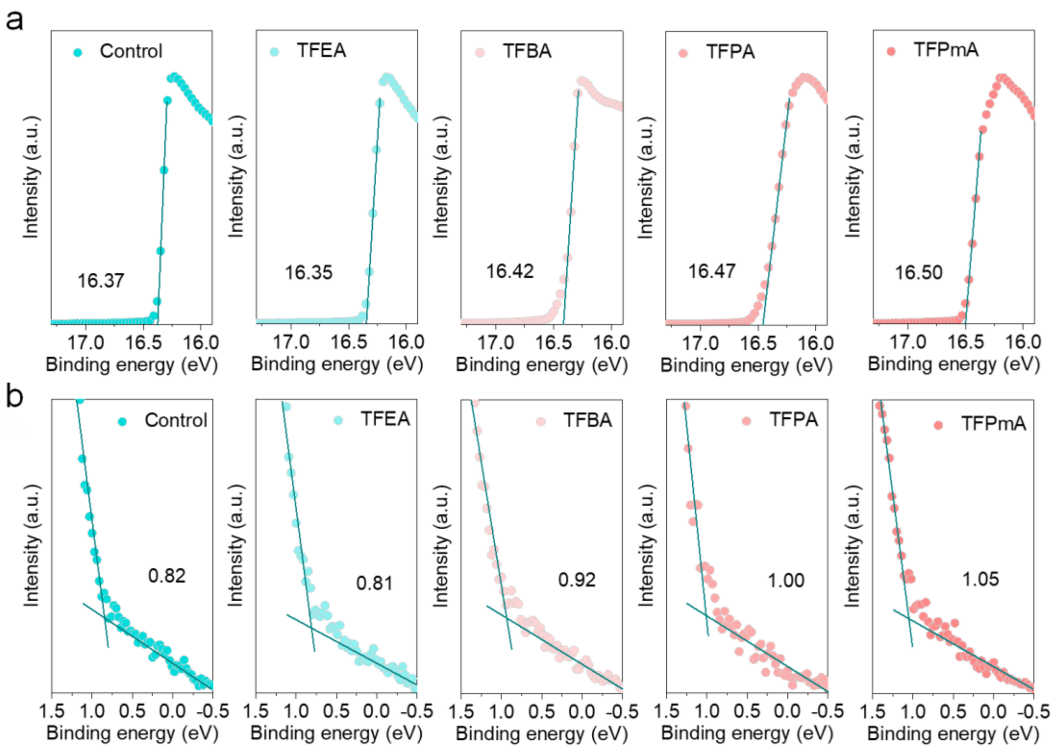

Supplementary Fig. 23| The UPS measurements for the control and ligands-treated perovskite films.

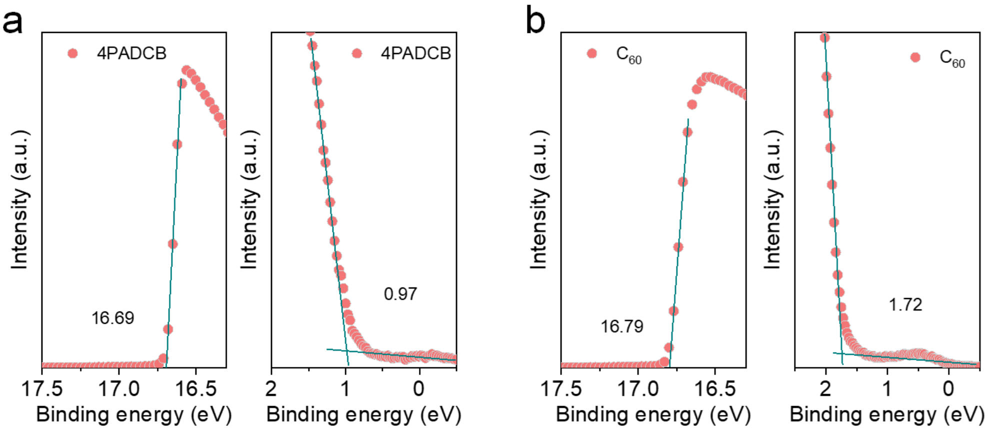

Supplementary Fig. 24| The UPS measurements of (a) 4PADCB hole-transporting layer and (b) C_{60} electron-transporting layer.

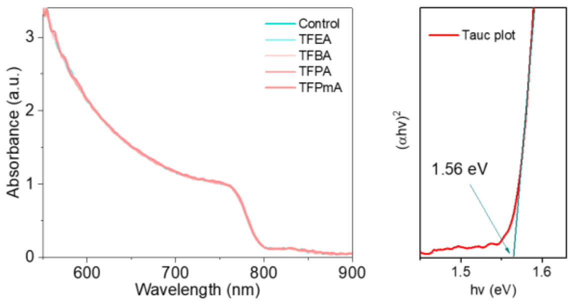

Supplementary Fig. 25| The ultraviolet-visible (UV-vis) absorption spectra and optical band gaps of perovskite films.

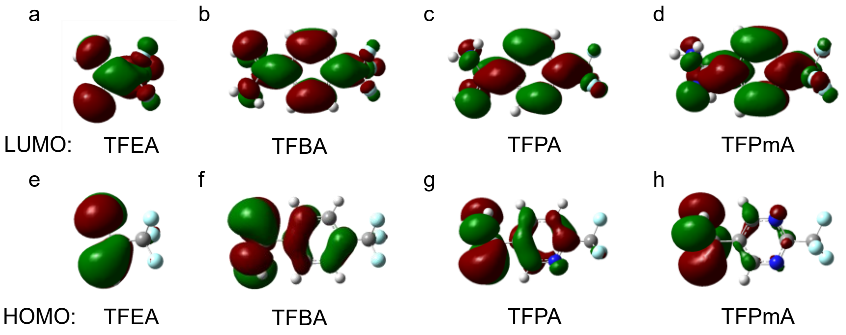

Supplementary Fig. 26| The Molecular orbital calculations. (a–d) the LUMO and (e-h) HOMO energy levels for TFEA, TFBA, TFPA, and TFPmA, respectively.

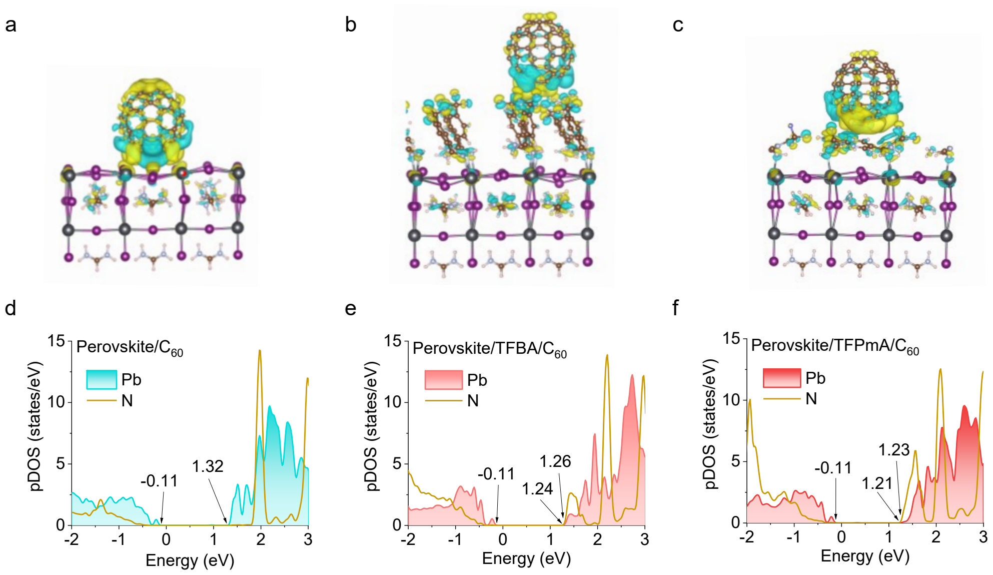

Supplementary Fig. 27| The Differential charge-density maps and computed pDOS plots of the Pb and N in perovskite/C_{60}, perovskite/TFBA/C_{60} and perovskite/TFPmA/C_{60}, respectively.

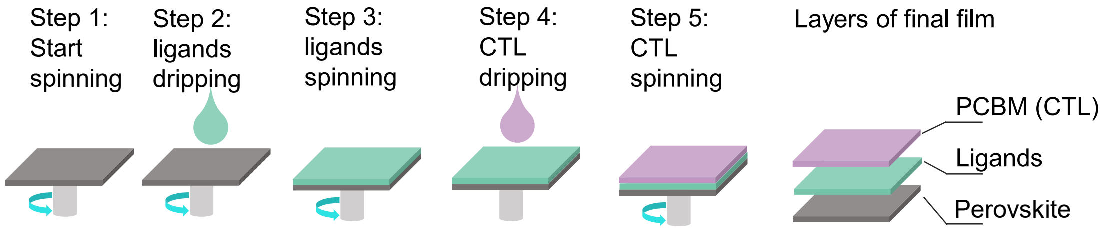

Supplementary Fig. 28| Schematic diagram of the ligands treatment and CTL deposition process during in situ PL measurements.

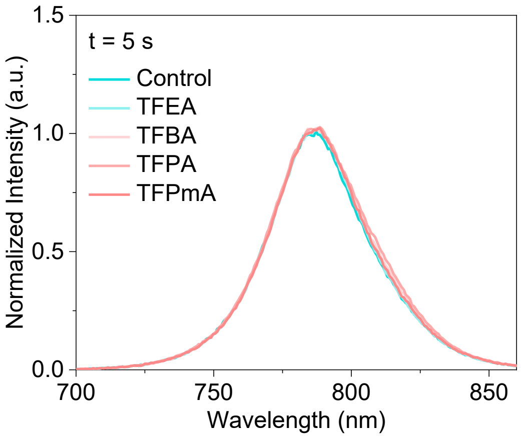

Supplementary Fig. 29| The PL spectra of the control and ligands-treated perovskite films extracted from in situ PL at 5 s.

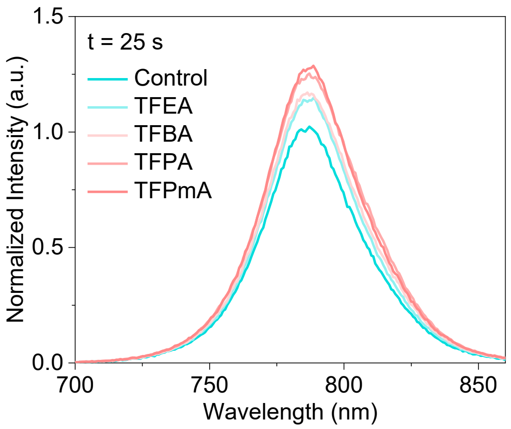

Supplementary Fig. 30| The PL spectra of the control and ligands-treated perovskite films extracted from in situ PL at 25 s.

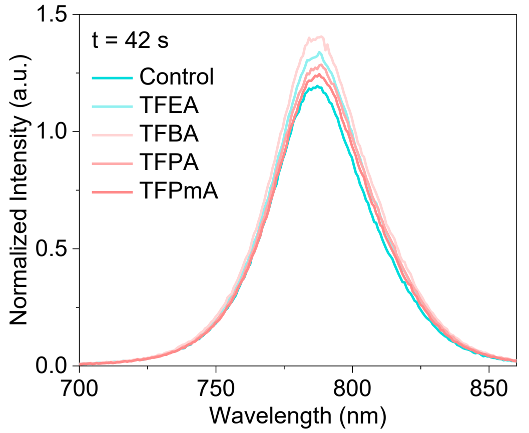

Supplementary Fig. 31| The PL spectra of the control and ligands-treated perovskite films extracted from in situ PL at 42 s.

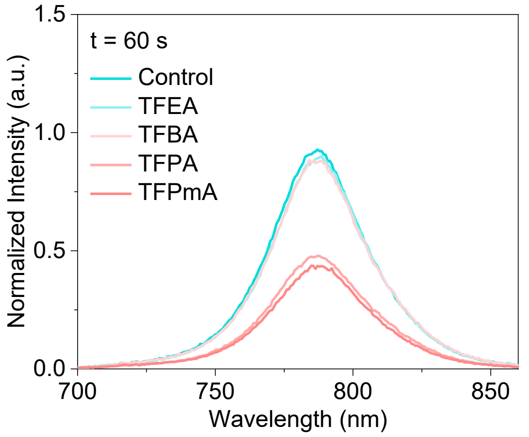

Supplementary Fig. 32| The PL spectra of the control and ligands-treated perovskite films extracted from in situ PL at 60 s.

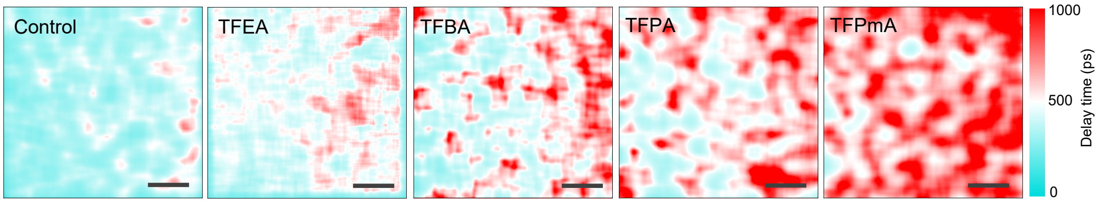

Supplementary Fig. 33| The TRPL mapping of the control and ligands-treated perovskite films, respectively. The scale bar is 2 μm.

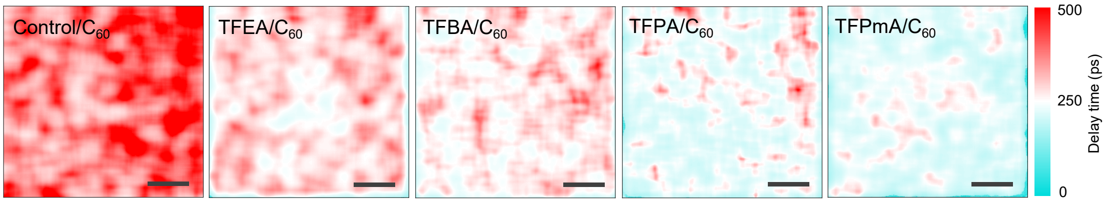

Supplementary Fig. 34| The TRPL mapping of the control and ligands-treated perovskite films with C_{60}, respectively. The scale bar is 2 μm.

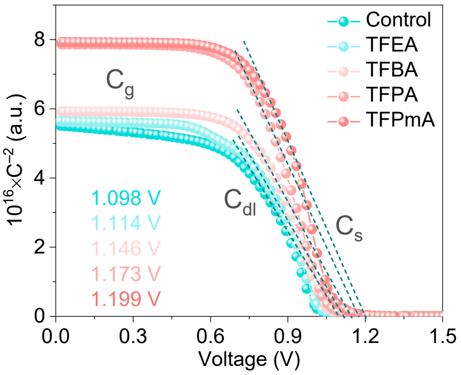

Supplementary Fig. 35| The Mott-Schottky analysis for the control and ligands-treated devices.

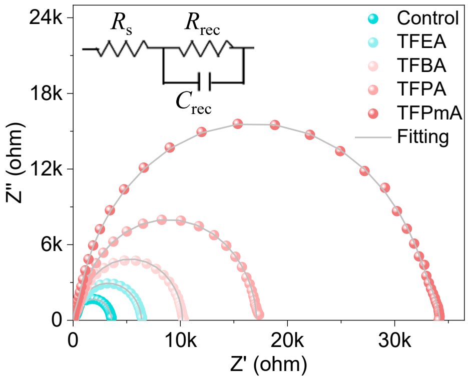

Supplementary Fig. 36| The Nyquist plots of the control and ligands-treated devices measured under dark conditions. The inset shows the equivalent circuit model (R(CR)) used to fit the impedance spectra, where R_s represents the series resistance, R_{rec} corresponds to the recombination resistance and C_{rec} denotes the recombination capacitance. The extracted fitting uncertainties for the control, TFEA-, TFBA-, TFPA-, and TFPmA-treated devices are 6.8%, 2.9%, 2.6%, 4.8%, and 1.4%, respectively.

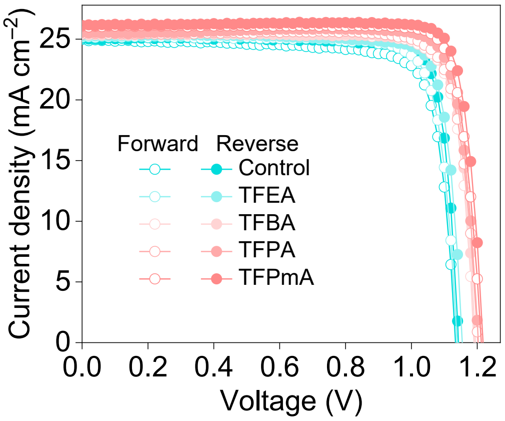

Supplementary Fig. 37| Forward and Reverse scan parameters of the control and ligands-treated solar cells.

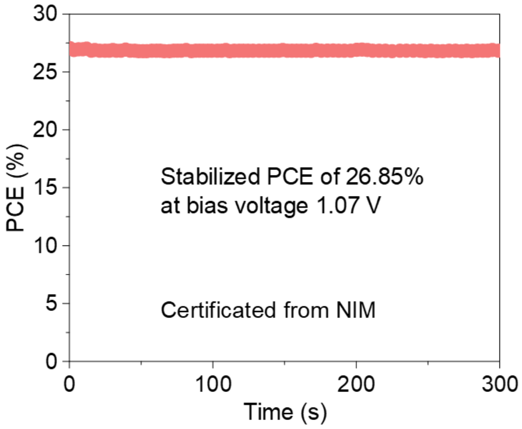

Supplementary Fig. 38| The Certificated results from the National Institute of Metrology, China (NIM, China). The stabilized power output (SPO) of the TFPmA treated device.

中国计量科学研究院
National Institute of Metrology,China

Appendix: Summary of the Report
Report No.: GXgf2025-03922
Client: Shaanxi Normal University
Sample: Perovskite Solar Cell
Type/Model: Perovskite Solar Cell
DUT S/N: Y5-M0821-2
Manufacturer: Shaanxi Normal University
Sample temperature: (25±1)°C
Date of Test: 11/28/2025
Environmental conditions: (22 ± 1)°C RH (17± 2)%
Mask: An aperture area of 7.420 mm^2 (M0821-2, Certificate No.: CDjc2025-05685)

The test has been conducted by the PV Metrology Lab of NIM (National Institute of Metrology, China). Measurement of irradiance intensity and all other measurements are traceable to the International System of Units (SI). The data in this report is only applicable to the samples tested at that time. For more details, please refer to the text of the report.

	Area (mm^2)	I_{sc}(mA)	V_{oc}(V)	P_{max}(mW)
Reverse Scan	7.420	1.961	1.194	2.034
Forward Scan	7.420	1.961	1.188	1.955
	I_{max}(mA)	V_{max}(V)	FF (%)	η (%)
Reverse Scan	1.884	1.080	86.90	27.41
Forward Scan	1.844	1.060	83.89	26.35

I-V Characterization Methods:
JJF 1622-2017: Calibration Specification of Solar Cells: Photoelectric Properties
JJF 2129-2024: Calibration Specification for Perovskite Solar Cells: Photoelectric Characteristic Parameters
Reference Solar Cell:
Type: Mono-Si
Solar Simulator:
Classification: A+AA+ (Double-light source: Xeon and Halogen);
Total irradiance: 1000 W/m^2 based on I_{sc} of the above Reference Solar Cell.

Supplementary Fig. 39| The Certificated results from the National Institute of Metrology, China (NIM, China). The forward scan (reverse scan) was performed from −0.10 V to 1.28 V (1.28 V to −0.1 V).

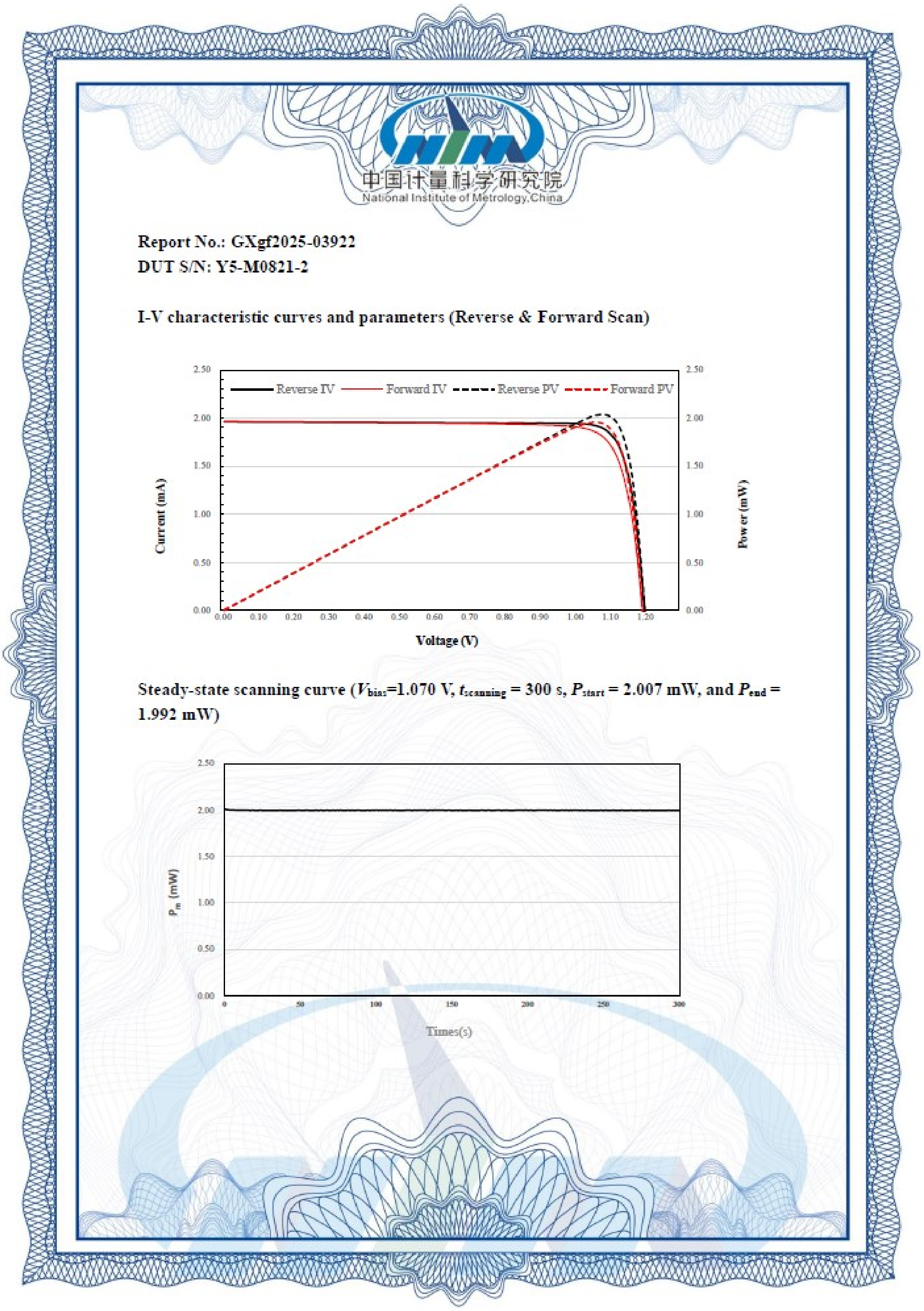
中国计量科学研究院
National Institute of Metrology,China

Report No.: GXgf2025-03922
DUT S/N: Y5-M0821-2

I-V characteristic curves and parameters (Reverse & Forward Scan)

Steady-state scanning curve (V_{bias}=1.070 V, $t_{scanning}$ = 300 s, P_{start} = 2.007 mW, and P_{end} = 1.992 mW)

Supplementary Fig. 40| The Certificated results from the National Institute of Metrology, China (NIM, China). The steady-state scanning was performed for 300 s with one read per second under 1.07 V.

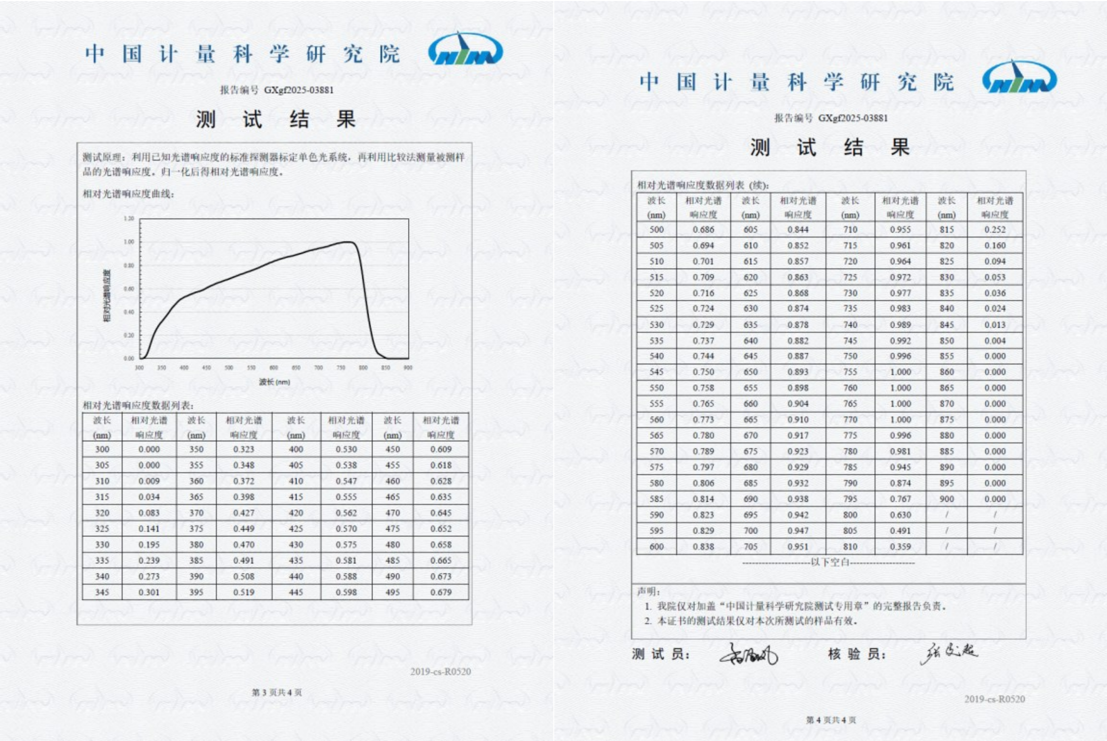

中国计量科学研究院

报告编号 GXgf2025-03881

测 试 结 果

测试原理：利用已知光谱响应度的标准探测器标定单色光系统，再利用比较法测量被测样品的光谱响应度，归一化后得相对光谱响应度。

相对光谱响应度曲线：

相对光谱响应度数据列表：

波长 (nm)	相对光谱响应度	波长 (nm)	相对光谱响应度	波长 (nm)	相对光谱响应度	波长 (nm)	相对光谱响应度
300	0.000	350	0.323	400	0.530	450	0.609
305	0.000	355	0.348	405	0.538	455	0.618
310	0.009	360	0.372	410	0.547	460	0.628
315	0.034	365	0.398	415	0.555	465	0.635
320	0.083	370	0.427	420	0.562	470	0.645
325	0.141	375	0.449	425	0.570	475	0.652
330	0.195	380	0.470	430	0.575	480	0.658
335	0.239	385	0.491	435	0.581	485	0.665
340	0.273	390	0.508	440	0.588	490	0.673
345	0.301	395	0.519	445	0.598	495	0.679

2019-cs-R0520

第 3 页共 4 页

中国计量科学研究院

报告编号 GXgf2025-03881

测 试 结 果

相对光谱响应度数据列表 (续)：

波长 (nm)	相对光谱响应度	波长 (nm)	相对光谱响应度	波长 (nm)	相对光谱响应度	波长 (nm)	相对光谱响应度
500	0.686	605	0.844	710	0.955	815	0.252
505	0.694	610	0.852	715	0.961	820	0.160
510	0.701	615	0.857	720	0.964	825	0.094
515	0.709	620	0.863	725	0.972	830	0.053
520	0.716	625	0.868	730	0.977	835	0.036
525	0.724	630	0.874	735	0.983	840	0.024
530	0.729	635	0.878	740	0.989	845	0.013
535	0.737	640	0.882	745	0.992	850	0.004
540	0.744	645	0.887	750	0.996	855	0.000
545	0.750	650	0.893	755	1.000	860	0.000
550	0.758	655	0.898	760	1.000	865	0.000
555	0.765	660	0.904	765	1.000	870	0.000
560	0.773	665	0.910	770	1.000	875	0.000
565	0.780	670	0.917	775	0.996	880	0.000
570	0.789	675	0.923	780	0.981	885	0.000
575	0.797	680	0.929	785	0.945	890	0.000
580	0.806	685	0.932	790	0.874	895	0.000
585	0.814	690	0.938	795	0.767	900	0.000
590	0.823	695	0.942	800	0.630	/	/
595	0.829	700	0.947	805	0.491	/	/
600	0.838	705	0.951	810	0.359	/	/

--------------------以下空白--------------------

声明：
1. 我院仅对加盖“中国计量科学研究院测试专用章”的完整报告负责。
2. 本证书的测试结果仅对本次所测试的样品有效。

测 试 员： 核 验 员：

2019-cs-R0520

第 4 页共 4 页

Supplementary Fig. 41| Relative spectral response (SR) curves measured at National Institute of Metrology, China (NIM, China) and the corresponding data table. The calculated spectral mismatch factor is 1.0043, indicating negligible mismatch under AM 1.5G conditions.

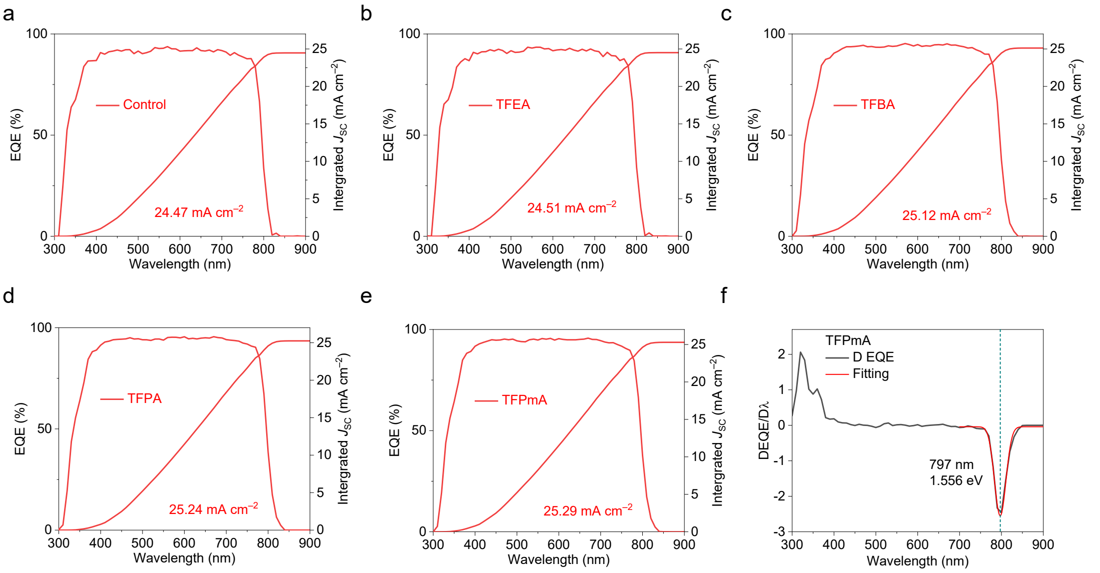

Supplementary Fig. 42| The external quantum efficiency (EQE) measurements. (a-e) EQE spectra and corresponding integrated current density for control and all ligands modified solar cells. (f) the bandgap value extracted from the derived curve of EQE spectra in Figure S42e.

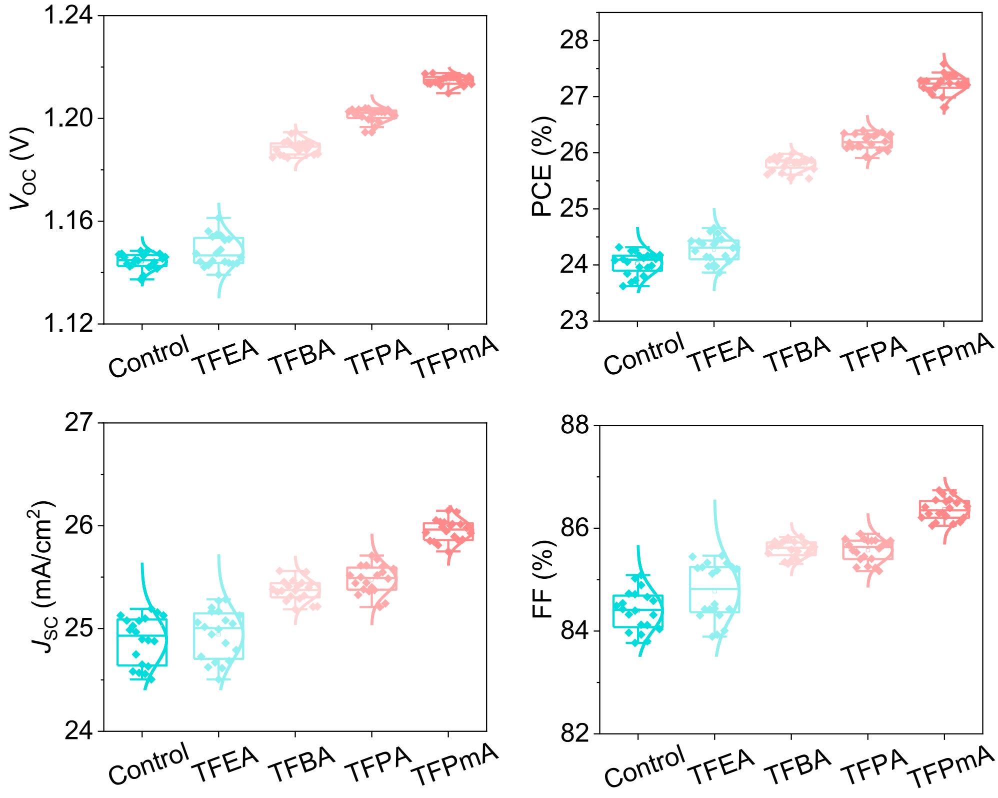

Supplementary Fig. 43| Statistical parameters of 20 independent devices without and with ligands treatment.

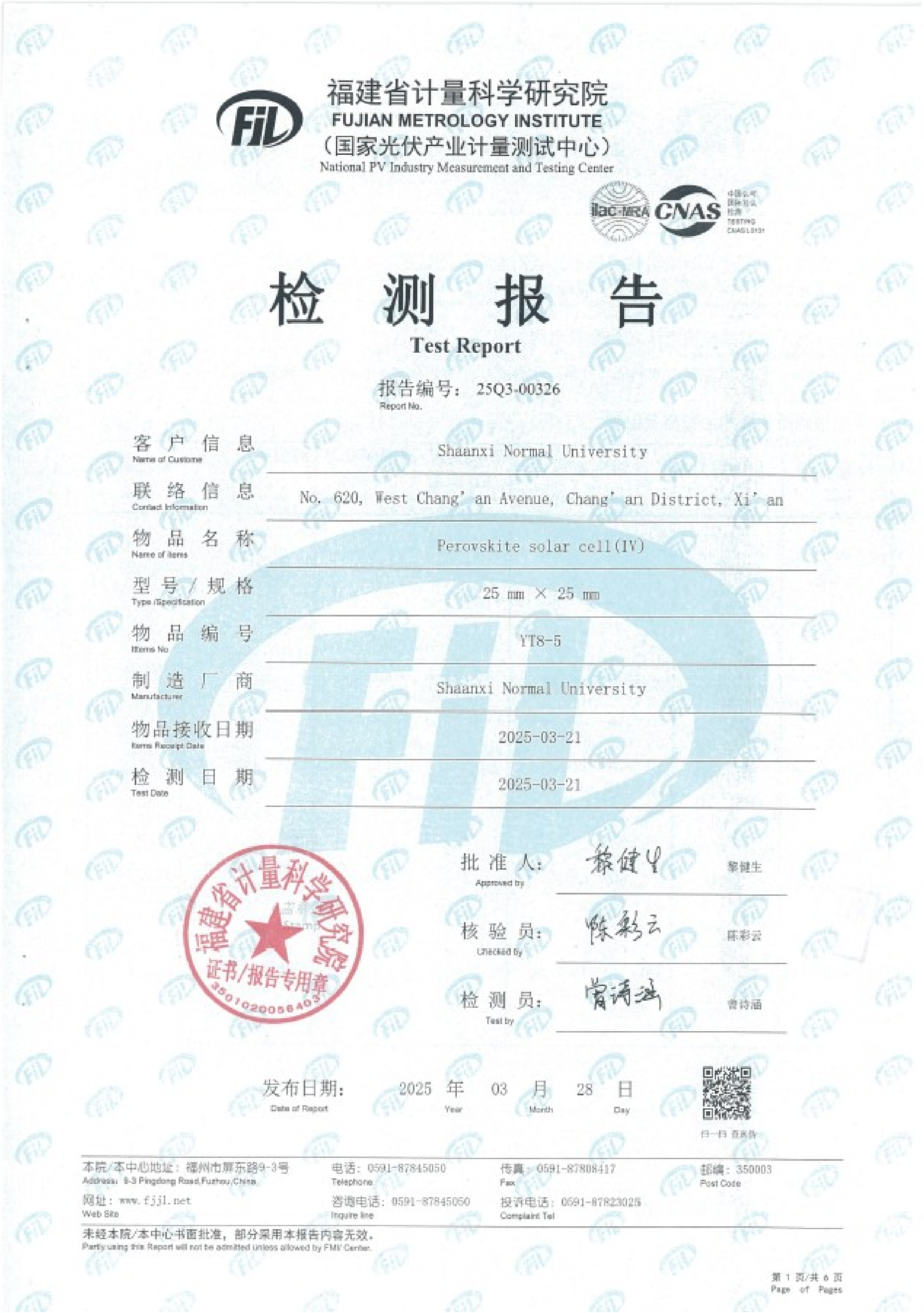
福建省计量科学研究院
FUJIAN METROLOGY INSTITUTE
（国家光伏产业计量测试中心）
National PV Industry Measurement and Testing Center

检 测 报 告
Test Report

报告编号：25Q3-00326
Report No.

客户信息 Name of Custome	Shaanxi Normal University
联络信息 Contact Information	No. 620, West Chang'an Avenue, Chang'an District, Xi'an
物品名称 Name of items	Perovskite solar cell(IV)
型号/规格 Type /Specification	25 mm × 25 mm
物品编号 Items No	YT8-5
制造厂商 Manufacturer	Shaanxi Normal University
物品接收日期 Items Receipt Date	2025-03-21
检测日期 Test Date	2025-03-21

福建省计量科学研究院 证书/报告专用章 3501020056403

批准人：Approved by 黎健生

核验员：Checked by 陈彩云

检测员：Test by 曾诗涵

发布日期：Date of Report 2025 年 Year 03 月 Month 28 日 Day

扫一扫 查真伪

本院/本中心地址：福州市屏东路9-3号 Address: 9-3 Pingdong Road,Fuzhou,China
电话：0591-87845050 Telephone
传真：0591-87808417 Fax
邮编：350003 Post Code
网址：www.fjjl.net Web Site
咨询电话：0591-87845050 Inquire line
投诉电话：0591-87823025 Complaint Tel

未经本院/本中心书面批准，部分采用本报告内容无效。
Partly using this Report will not be admitted unless allowed by FMI/ Center.

第 1 页/共 6 页
Page of Pages

Supplementary Fig. 44| The Certificated results for wide-bandgap device (1.68 eV) from the National PV Industry Measurement and Testing Center (NPVM, China).

福建省计量科学研究院

FUJIAN METROLOGY INSTITUTE

（国家光伏产业计量测试中心）

National PV Industry Measurement and Testing Center

25Q3-00326

检测结果/说明：

Results of Test and additional explanation

1. Standard Test Condition (STC)：Total Irradiance：1000 W/m^2

Temperature：25.0 °C

Spectral Distribution：AM1.5G

2.Measurement Data and I-V/P-V Curves under STC

Forward Scan

I_{sc} (mA)	V_{oc}(V)	I_{MPP}(mA)	V_{MPP}(V)	P_{MPP} (mW)	FF(%)	A (cm^2)
1.551	1.267	1.415	1.062	1.503	76.48	0.0718

Reverse Scan

I_{sc} (mA)	V_{oc}(V)	I_{MPP}(mA)	V_{MPP}(V)	P_{MPP} (mW)	FF(%)	A (cm^2)
1.557	1.266	1.494	1.107	1.654	83.91	0.0718

Mismatch factor:1.013

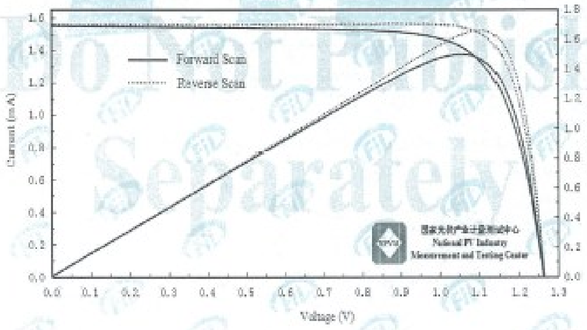

Figure 1. I-V and P-V characteristic curves of the measured sample under STC

检测报告续页专用

Continued page of test report

第 3 页/共 6 页

Page of Pages

Supplementary Fig. 45| The Certificated results for wide-bandgap device (1.68 eV) from the National PV Industry Measurement and Testing Center (NPVM, China). The forward scan (reverse scan) was performed from −0.10 V to 1.4 V (1.4 V to −0.1 V).

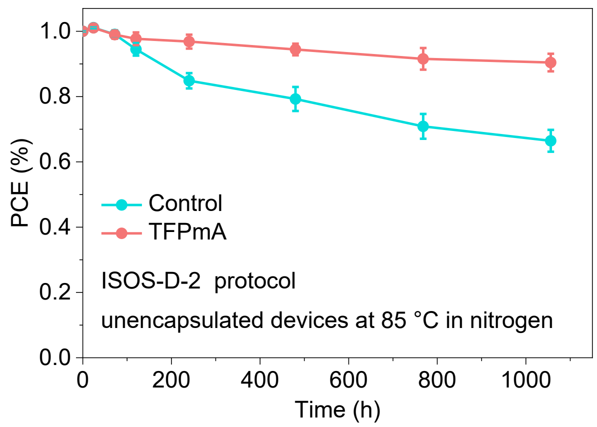

Supplementary Fig.46| Thermal aging test of the control and TFPmA treated devices at 85 °C.

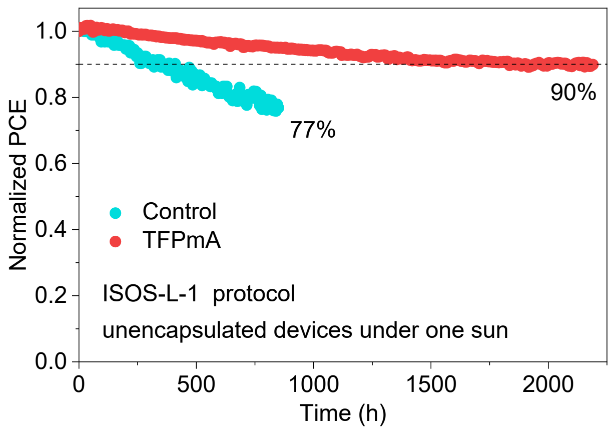

Supplementary Fig. 47| MPP tracking test of the control and TFPmA treated devices under light soaking.

中国计量科学研究院
National Institute of Metrology,China

Appendix: Summary of the Report
Report No.: GXgf2025-03921
Client: Shaanxi Normal University
Sample: Perovskite Solar Cell
Type/Model: Perovskite Solar Cell
DUT S/N: Y4-M0821
Manufacturer: Shaanxi Normal University
Sample temperature: (25±1)°C
Date of Test: 11/28/2025
Environmental conditions: (22 ± 1)°C RH (17± 2)%
Mask: An aperture area of 109.896 mm^2 (M0821, Certificate No.: CDjc2025-05684)

The test has been conducted by the PV Metrology Lab of NIM (National Institute of Metrology, China). Measurement of irradiance intensity and all other measurements are traceable to the International System of Units (SI). The data in this report is only applicable to the samples tested at that time. For more details, please refer to the text of the report.

	Area (mm^2)	I_{sc}(mA)	V_{oc}(V)	P_{max}(mW)
Reverse Scan	109.896	27.870	1.195	28.150
Forward Scan	109.896	27.920	1.193	27.250
	I_{max}(mA)	V_{max}(V)	FF (%)	η (%)
Reverse Scan	26.560	1.060	84.52	25.62
Forward Scan	26.200	1.040	81.83	24.80

I-V Characterization Methods:
JJF 1622-2017: Calibration Specification of Solar Cells: Photoelectric Properties
JJF 2129-2024: Calibration Specification for Perovskite Solar Cells: Photoelectric Characteristic Parameters
Reference Solar Cell:
Type: Mono-Si
Solar Simulator:
Classification: A+AA+ (Double-light source: Xeon and Halogen);
Total irradiance: 1000 W/m^2 based on I_{sc} of the above Reference Solar Cell.

Supplementary Fig. 48| The Certificated results of the 1 cm^2-class devices from the National Institute of Metrology, China (NIM, China). The forward scan (reverse scan) was performed from −0.10 V to 1.28 V (1.28 V to −0.1 V).

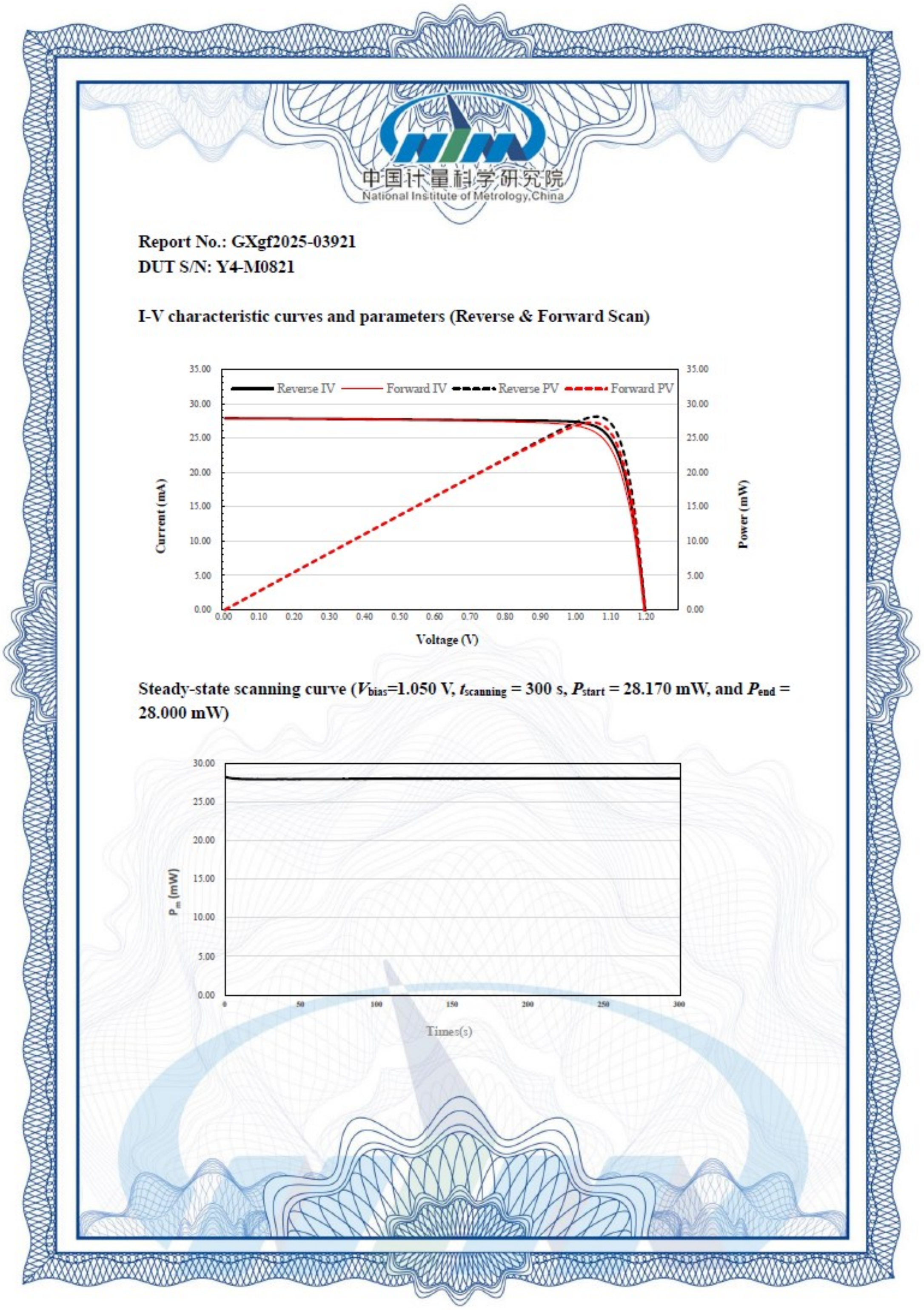
中国计量科学研究院
National Institute of Metrology,China

Report No.: GXgf2025-03921
DUT S/N: Y4-M0821

I-V characteristic curves and parameters (Reverse & Forward Scan)

Steady-state scanning curve (V_{bias}=1.050 V, $t_{scanning}$ = 300 s, P_{start} = 28.170 mW, and P_{end} = 28.000 mW)

Supplementary Fig. 49| The Certificated results of the 1 cm^2-class devices from the National Institute of Metrology, China (NIM, China). The steady-state scanning was performed for 300 s with one read per second under 1.05 V.

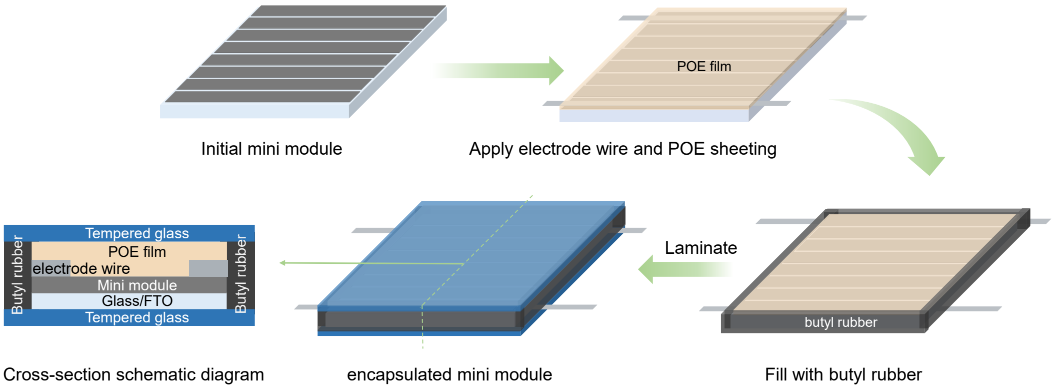

Supplementary Fig. 50| The schematic diagram illustrating the encapsulation sequence and cell interconnections.

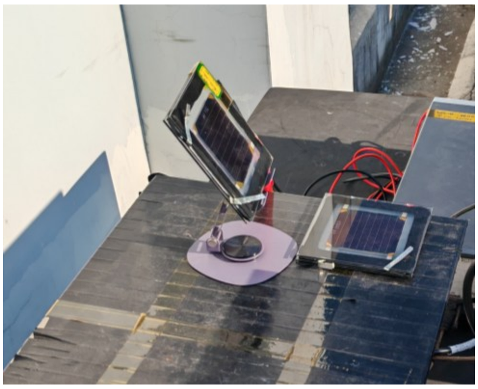

Supplementary Fig. 51| The photo of outdoor real-time field testing for TFPmA treated perovskite modules.

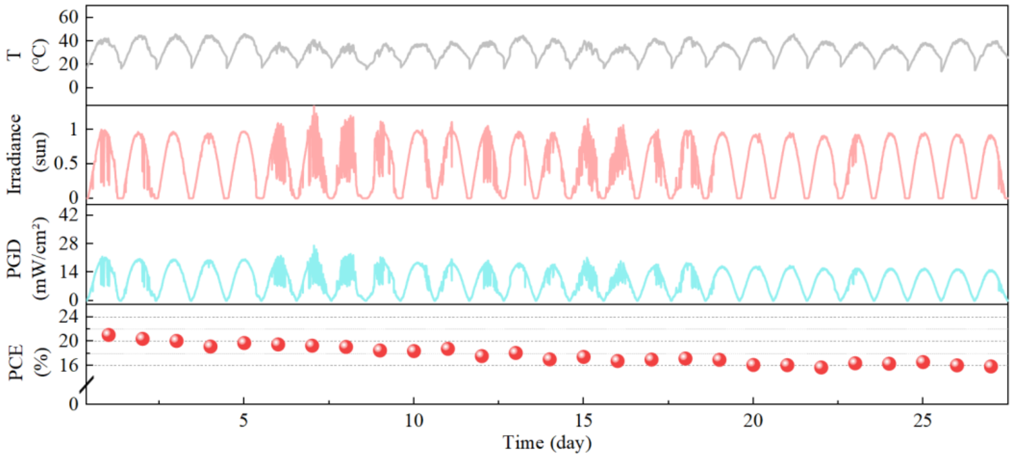

Supplementary Fig. 52| The real-time field testing of control group under actual out-door operating conditions for 27 days.

Supplementary Tables

Supplementary Table 1| Statistical analysis of nanoscale aggregation domains with different sizes, extracted from distinct regions of nano-IR mapping (**Fig. 3c**) of ligand-treated perovskite films.

Sample	Quantity	Maximum (nm)	Average (nm)
TFEA	12	167	100 ± 35
TFBA	22	90	61 ± 13
TFPA	30	85	50 ± 17
TFPmA	35	56	39 ± 9

Supplementary Table 2| Energy level parameters calculated from UPS measurements for the control and ligands-treated perovskite films.

	E_{cutoff} (eV)	E_{feimi} (eV)	WF (eV)	E_f (eV)	VBM (eV)	CBM (eV)
Control	16.37	0.82	4.85	–4.85	–5.67	–4.11
TFEA	16.35	0.81	4.87	–4.87	–5.68	–4.12
TFBA	16.42	0.92	4.80	–4.80	–5.72	–4.16
TFPA	16.47	1.00	4.75	–4.75	–5.75	–4.19
TFPmA	16.50	1.05	4.72	–4.72	–5.77	–4.21

Supplementary Table 3| The LUMO and HOMO energy levels for TFEA, TFBA, TFPA, and TFPmA, respectively.

	TFEA	TFBA	TFPA	TFPmA
LUMO (eV)	–0.63	–1.52	–1.93	–2.50
HOMO (eV)	–7.10	–6.64	–6.91	–7.02

Supplementary Table 4| TRPL carrier decay lifetime parameters fitted by a double exponential function for control and ligands-treated with Glass/perovskite/ligands structure.

Sample	τ_{ave} (μs)	τ_1 (μs)	Intensity τ_1(%)	τ_2 (μs)	Intensity τ_2 (%)
Control	1.08	0.06	6.94	1.15	93.06
TFEA	1.29	0.08	6.49	1.38	93.51
TFBA	1.69	0.05	3.10	1.75	96.90
TFPA	2.33	0.06	1.69	2.37	98.31
TFPmA	3.19	0.06	1.12	3.23	98.88

Supplementary Table 5| TRPL carrier decay lifetime parameters fitted by a double exponential function for control and ligands-treated with Glass/perovskite/ligands/C_{60} structure.

Sample	τ_{ave} (ns)	τ_1 (ns)	Intensity τ_1(%)	τ_2 (ns)	Intensity τ_2 (%)
Control	65.9	7.5	18.08	78.8	81.92
TFEA	45.5	9.0	26.77	58.9	73.23
TFBA	29.2	7.9	41.53	44.3	58.47
TFPA	20.8	5.9	46.87	33.8	53.13
TFPmA	11.0	4.0	52.27	18.7	47.73

Supplementary Table 6| EIS impedance fitting parameters for the control and ligands-treated devices.

Sample	R_s (Ω)	R_{rec} (Ω)	C_{rec} (F)
Control	65.8	3314	2.35×10^{-8}
TFEA	41.4	6209	2.84×10^{-8}
TFBA	25.9	10300	4.26×10^{-8}
TFPA	16.2	15270	7.91×10^{-8}
TFPmA	14.4	29690	11.3×10^{-8}

Supplementary Table 7| Forward and Reverse scan parameters of the control and ligands-treated solar cells.

	Sample	V_{OC} (V)	J_{SC} (mA·cm^{-2})	FF (%)	PCE (%)	HI (%)
Control	Forward	1.134	24.98	80.58	22.83	5.9
	Reverse	1.143	25.07	84.66	24.26	
TFEA	Forward	1.139	25.18	82.43	23.65	4.1
	Reverse	1.154	25.27	84.52	24.65	
TFBA	Forward	1.190	25.48	83.24	25.22	2.9
	Reverse	1.195	25.41	85.58	25.98	
TFPA	Forward	1.202	25.43	83.80	25.61	2.9
	Reverse	1.203	25.55	85.76	26.37	
TFPmA	Forward	1.212	26.07	85.44	27.01	2.1
	Reverse	1.218	26.13	86.69	27.58	

Supplementary Table 8| Statistics of the photovoltaic parameters (optimal, average (ave.) and standard deviation (s.d.)) of the control and ligands-treated solar cells (20 independent cells).

Sample	PCE (%)	V_{OC} (V)	J_{SC} (mA·cm^{-2})	FF (%)
Control	24.26	1.143	25.07	84.66
ave. / s.d.	24.03±0.19	1.144±0.003	24.89±0.23	84.40±0.39
TFEA	24.65	1.154	25.27	84.52
ave. / s.d.	24.27±0.23	1.148±0.006	24.94±0.24	84.77±0.55
4TFBA	25.98	1.195	25.41	85.58
ave. / s.d.	25.80±0.11	1.189±0.003	25.37±0.10	85.59±0.16
4TFPA	26.37	1.203	25.55	85.76
ave. / s.d.	26.19±0.15	1.201±0.002	25.48±0.14	85.57±0.22
TFPmA	27.58	1.218	26.13	86.69
ave. / s.d.	27.23±0.17	1.214±0.002	25.95±0.11	86.36±0.21

Supplementary Table 9| Photovoltaic parameters of wide bandgap (1.68 eV) solar cells treated without/with TFPmA.

	Sample	V_{OC} (V)	J_{SC} (mA·cm^{-2})	FF (%)	PCE (%)	HI (%)
Control	Forward	1.182	21.11	83.92	20.94	2.0
	Reverse	1.193	21.20	84.44	21.37	
TFPmA	Forward	1.275	22.02	85.13	23.91	0.5
	Reverse	1.279	22.03	85.30	24.04	

Supplementary Table 10| Photovoltaic parameters of scalable solar cells treated with TFPmA.

Area (cm^2)	Scan	V_{OC} (V)	J_{SC} ($mA \cdot cm^{-2}$)	FF (%)	PCE (%)	HI (%)
1.098	Forward	1.197	25.46	85.15	25.94	1.7
	Reverse	1.202	25.54	86.04	26.40	
13.95	Forward	7.146	4.181	81.88	24.46	1.1
	Reverse	7.160	4.177	82.73	24.74	

Reference

1 Kresse, G. & Furthmüller, J. Efficient iterative schemes for ab initio total-energy calculations using a plane-wave basis set. *Physical Review B* **54**, 11169-11186 (1996).

2 Kresse, G. & Furthmüller, J. Efficiency of ab-initio total energy calculations for metals and semiconductors using a plane-wave basis set. *Computational Materials Science* **6**, 15-50 (1996).

3 Perdew, J. P., Burke, K. & Ernzerhof, M. Generalized Gradient Approximation Made Simple. *Physical Review Letters* **77**, 3865-3868 (1996).

4 Blöchl, P. E. Projector augmented-wave method. *Physical Review B* **50**, 17953-17979 (1994).

5 Kresse, G. & Joubert, D. From ultrasoft pseudopotentials to the projector augmented-wave method. *Physical Review B* **59**, 1758-1775 (1999).

6 Monkhorst, H. J. & Pack, J. D. Special points for Brillouin-zone integrations. *Physical Review B* **13**, 5188-5192 (1976).

7 Grimme, S., Antony, J., Ehrlich, S. & Krieg, H. A consistent and accurate ab initio parametrization of density functional dispersion correction (DFT-D) for the 94 elements H-Pu. *The Journal of Chemical Physics* **132**, 154104 (2010).

8 Grimme, S., Ehrlich, S. & Goerigk, L. Effect of the damping function in dispersion corrected density functional theory. *Journal of Computational Chemistry* **32**, 1456-1465 (2011).
