# Supplementary material for: Stereoelectronic manipulation of ligands for perovskite solar cells
Source: Nature. 2026 May 13;654(8119):660–7. doi: 10.1038/s41586-026-10626-0 (PMC13275290; doi:10.1038/s41586-026-10626-0)
Supplement: Supplementary file 2 — Reporting Summary [file 41586_2026_10626_MOESM2_ESM.pdf]

nature portfolio

Corresponding author(s): Shengzhong (Frank) Liu
Feng Gao
Kui Zhao

Solar Cells Reporting Summary

Nature Portfolio wishes to improve the reproducibility of the work that we publish. This form is intended for publication with all accepted papers reporting the characterization of photovoltaic devices and provides structure for consistency and transparency in reporting. Some list items might not apply to an individual manuscript, but all fields must be completed for clarity.

For further information on Nature Research policies, including our data availability policy, see Authors & Referees.

Experimental design

Please check the following details are reported in the manuscript, and provide a brief description or explanation where applicable.

1. Dimensions

Area of the tested solar cells

☒ Yes
☐ No

see Methods, 1.6 Solar cell characterization. The contact area of the cell is 0.12 cm2, and the illumination area is 0.074 cm2

Explain why this information is not reported/not relevant.

Method used to determine the device area

☒ Yes
☐ No

The area of our mask was certificated by the National Institute of Metrology (NIM).

Explain why this information is not reported/not relevant.

2. Current-voltage characterization

Current density-voltage (J-V) plots in both forward and backward direction

☒ Yes
☐ No

The J-V plots measured along both scan direction, as shown in Figure 5c and Supplementary Fig. 37.

Voltage scan conditions

☒ Yes
☐ No

The voltage range for forward and reverse scans were 1.4~–0.1 V with a step of 0.02 V and a delay time of 20 ms. See Methods, 1.6 Solar cell characterization

Explain why this information is not reported/not relevant.

Test environment

☒ Yes
☐ No

All devices were tested at room temperature (ca. 25 ?) in N2 glove box. See Methods, 1.6 Solar cell characterization

Explain why this information is not reported/not relevant.

Protocol for preconditioning of the device before its characterization

☒ Yes
☐ No

Figure 5g, the real-time field testing (ISOS-O-3 protocol) of TFPmA-treated module under actual out-door operating conditions for 258 days. Figure S46, thermal aging test (ISOS-D-2 protocol) of the control and TFPmA treated devices at 85 °C. Figure S47, MPP tracking test (ISOS-L-1 protocol) of the control and TFPmA treated devices under light soaking.

Explain why this information is not reported/not relevant.

Stability of the J-V characteristic

☒ Yes
☐ No

The stabilized power output of solar cell is shown in Figure S38, and the stabilized PCE of 26.85% at bias voltage 1.07V.

Explain why this information is not reported/not relevant.

3. Hysteresis or any other unusual behaviour

Description of the unusual behaviour observed during the characterization

☒ Yes
☐ No

see Figure S37 and Table S7. The forward and reverse scan efficiencies of the device are 27.58% and 27.01%, respectively, with a hysteresis factor of 0.021.

Explain why this information is not reported/not relevant.

Related experimental data

☒ Yes
☐ No

The forward and reverse scan data are shown in Figure S37 and Table S7.

Explain why this information is not reported/not relevant.

4. Efficiency

External quantum efficiency (EQE) or incident photons to current efficiency (IPCE)

☒ Yes
☐ No

The external quantum efficiency (EQE) measurements was shown in Figure S42.

Explain why this information is not reported/not relevant.

A comparison between the integrated response under the standard reference spectrum and the response measure under the simulator	☒ Yes<br>☐ No	The external quantum efficiency (EQE) integrated currents agree with J-V measurements (tolerance variation < 2.1%).<br>*Explain why this information is not reported/not relevant.*
For tandem solar cells, the bias illumination and bias voltage used for each subcell	☐ Yes<br>☒ No	*Provide a description of the measurement conditions.*<br>This is not a tandem solar cell

5. Calibration

Light source and reference cell or sensor used for the characterization	☒ Yes<br>☐ No	J-V and I-T curves were measured with source Meter (Keithley 2450) and solar simulator (SAN-EIELECTRIC DM-50S1) under AM1.5G.<br>*Explain why this information is not reported/not relevant.*
Confirmation that the reference cell was calibrated and certified	☒ Yes<br>☐ No	The calibrated Si-reference cell were certificated by NREL.<br>*Explain why this information is not reported/not relevant.*
Calculation of spectral mismatch between the reference cell and the devices under test	☒ Yes<br>☐ No	See Figure S41. The relative spectral response (SR) curves measured at National Institute of Metrology, China (NIM, China) and the corresponding data table.<br>*Explain why this information is not reported/not relevant.*

6. Mask/aperture

Size of the mask/aperture used during testing	☒ Yes<br>☐ No	The size of mask during certification is 0.0742 and 0.0718 cm2, respectively, as shown in Figure S39, S45.<br>*Explain why this information is not reported/not relevant.*
Variation of the measured short-circuit current density with the mask/aperture area	☐ Yes<br>☒ No	*Report the difference in the short-circuit current density values measured with the mask and aperture area.*<br>We did not do the current variation test with different mask areas.

7. Performance certification

Identity of the independent certification laboratory that confirmed the photovoltaic performance	☒ Yes<br>☐ No	Some cells were sent to National Institute of Metrology (NIM, China) and National PV Industry Measurement and Testing Center (NPVM, China) for certification.<br>*Explain why this information is not reported/not relevant.*
A copy of any certificate(s)	☒ Yes<br>☐ No	The related test report are shown in Figure S38-41, S44,45.<br>*Explain why this information is not reported/not relevant.*

8. Statistics

Number of solar cells tested	☒ Yes<br>☐ No	The statistics of the parameters of solar cells includes 20 individual devices are shown in Figure S43.<br>*Explain why this information is not reported/not relevant.*
Statistical analysis of the device performance	☒ Yes<br>☐ No	Statistical parameters of 20 devices without and with ligands treatment, as shown in Figure S43 and Table S8<br>*Explain why this information is not reported/not relevant.*

9. Long-term stability analysis

Type of analysis, bias conditions and environmental conditions	☒ Yes<br>☐ No	Figure 5g, the real-time field testing (ISOS-O-3 protocol) of TFPmA-treated module under actual out-door operating conditions for 258 days. Figure S46, thermal aging test (ISOS-D-2 protocol) of the control and TFPmA treated devices at 85 °C. Figure S47, MPP tracking test (ISOS-L-1 protocol) of the control and TFPmA treated devices under light soaking.<br>*Explain why this information is not reported/not relevant.*
