# Supplementary material for: Stereoelectronic manipulation of ligands for perovskite solar cells
Source: Nature. 2026 May 13;654(8119):660–7. doi: 10.1038/s41586-026-10626-0 (PMC13275290; doi:10.1038/s41586-026-10626-0)
Supplement: Supplementary file 3 — Peer Review File [file 41586_2026_10626_MOESM3_ESM.pdf]

nature portfolio

Peer Review File

Stereoelectronic Manipulation of Ligands for Perovskite Solar Cells

Corresponding Author: Professor Feng Gao

Any redactions in this file are there to maintain patient confidentiality, the confidentiality of unpublished data, or to remove third-party material.

This file contains all reviewer reports in order by version, followed by all author rebuttals in order by version.

Version 0:

Reviewer comments:

Referee #1

(Remarks to the Author)
The manuscript entitled “Stereoelectronic Manipulation of Ligands Toward Shockley–Queisser Limit of Perovskite Solar Cells” by Tinghuan Yang et al. presents a stereoelectronic molecular design strategy for interface engineering in perovskite solar cells (PSCs), achieving near-theoretical Shockley–Queisser efficiencies with record-level certified PCE and excellent outdoor stability. Specifically, the authors embed nitrogen atoms into conjugated ligand backbones to enable dual Pb–N coordination and Pb–I–π interactions, driving planar adsorption geometry. This strategy reconciles two traditionally antagonistic requirements—atomic-scale defect passivation and minimized transport distance—leading to 87.91% of the SQ limit, a certified PCE of 26.98% (27.58% lab), and 92.5% retention after 130 days of outdoor testing.
The work is conceptually novel, methodologically rigorous, and of broad potential interest to the photovoltaics and materials science communities. It addresses a fundamental bottleneck in PSC performance and stability and is supported by strong experimental evidence and theoretical investigations. While the manuscript is strong and timely, by demonstrating high performance, and real-world stability, several issues require clarification, improved contextualization and in some cases tempering of claims before the manuscript can be considered further. I therefore recommend major revision. My detailed comments are provided below.
1. Computational Methodology
The computational workflow is described with sufficient detail, combining molecular-level calculations (Gaussian 16 at the B3LYP-D3BJ/def2-TZVP level with frequency analysis) and periodic perovskite/ligand models (VASP with PBE-GGA, PAW potentials, 450 eV cutoff, Γ-centered k-point sampling, and DFT-D3 dispersion corrections). The adsorption and binding energies are well defined, and the structural optimizations appear carefully conducted.
Nevertheless, some concerns remain regarding the quantitative reliability of the periodic DFT simulations:
• Γ-point only sampling (1×1×1): While this is sometimes acceptable for large supercells, Pb–I perovskite frameworks are sensitive to Brillouin zone sampling, and Γ-only calculations may not capture the electronic redistribution accurately. A denser k-point grid (e.g., 2×2×1 or higher) would improve reliability.
• Plane-wave cutoff (450 eV): This value may be marginal for Pb/I-containing systems, where higher cutoffs (≥500 eV) are often required. Considering that the reported adsorption/binding energy differences are on the order of 0.1–0.2 eV, the current settings may not provide fully converged values.
• Energy interpretation: The manuscript interprets trends in ligand stabilization based on small differences in adsorption/binding energies. Given the computational setup, such differences could be within the numerical uncertainty of the method, and caution should be exercised in drawing strong conclusions..
• Reference consistency: It should be clarified whether identical supercell dimensions, cutoffs, and k-point sampling were applied to both isolated ligands and combined systems, to avoid spurious contributions from vacuum-size or strain effects.
2. Spectroscopic Evidence
The additional experimental evidence significantly strengthens the mechanistic claims of stereoelectronic ligand regulation at the perovskite/CTL interface. Nevertheless, a few aspects require clarification and/or further support before the conclusions can be fully substantiated:
XPS Data Interpretation: The observed monotonic binding energy shifts in Pb 4f and I 3d peaks are attributed exclusively to Pb–N coordination and Pb–I–π interactions. However, XPS binding energies can also be influenced by changes in surface stoichiometry, defect concentration, or sample charging. The authors should discuss these alternative contributions, clarify

whether peak deconvolution was performed, and describe how reproducibility was ensured.
^{1}H NMR Assignments and Interpretation: The reported δHα and δHβ shifts are consistent with progressive multicenter coordination, but solvent effects, dynamic exchange, and concentration-dependent aggregation are not considered. The authors should acknowledge these limitations or indicate whether variable concentration/temperature studies were attempted.
FTIR Evidence of Coordination: The assignments of vibrational shifts (C=N, C–N, ring modes) to Pb–N and Pb–I–π interactions are reasonable but could also result from hydrogen bonding or heterogeneous coordination. Peak broadening should be analyzed more quantitatively (e.g., FWHM fitting), and alternative interpretations explicitly addressed.
Cross-technique consistency: While the manuscript claims excellent agreement between XPS, NMR, FTIR, and DFT, some trends are not fully reconciled. For example, NMR suggests a transition from monodentate to multicenter coordination, while FTIR emphasizes dominant Pb–N interactions. These differences should be explicitly discussed.
3. Structural and Morphological Analyses
GIWAXS, XRD, and SEM results are used to exclude low-dimensional phases or lattice distortions. However, these techniques may lack sensitivity to subtle local rearrangements or minority phases. The conclusions should be tempered accordingly. SEM images support improved film uniformity but remain qualitative; their direct correlation with electronic improvements should be made more cautiously.
4. Energetics and Photophysics
UPS results demonstrate favorable energy-level alignment, but attributing ~0.1 eV shifts to Pb–I–π interactions is speculative without direct evidence of orbital hybridization. The reported improvements in QFLS are corroborated by in situ PL, TRPL mapping (Supplementary Figs. 18–24), and Mott–Schottky analysis (Supplementary Fig. 25), which collectively confirm reduced nonradiative recombination. Nevertheless, the mechanistic interpretation of these improvements as arising from “bandgap homogenization” could be more cautiously framed, as the presented photophysical data capture carrier dynamics and defect passivation but do not unambiguously evidence a homogenization of the electronic bandgap.
In situ PL measurements indicate a two-stage ligand anchoring and defect passivation process, and TRPL lifetimes demonstrate the dual role of ligand adsorption in passivation and charge extraction; however, assigning specific mechanistic steps solely from PL/TRPL intensity and decay trends may be oversimplified, as these signals reflect a convolution of bulk and interfacial processes. Collectively, the spectroscopic data convincingly demonstrate ligand-mediated interfacial effects, but a cautious interpretation acknowledging these potential complexities would strengthen the manuscript.
5. Device-Level Electrical Analyses
Mott–Schottky, EIS, and light-intensity VOC analyses support reduced recombination and improved interface passivation. However, the unusually large changes (78% reduction in Rs, ~9× increase in Rrec) raise the possibility of parasitic or contact-related contributions. The correlation between these results and the proposed “σ/π dual bonding” should therefore be interpreted more carefully.
6. Stability and Module Fabrication
The outdoor stability test (130 days, 0–1.31 sun, –11.5 °C to 65.7 °C) is a remarkable result, with only 7.5% loss in efficiency. However, as the test was not conducted under standardized ISOS or IEC protocols, comparisons with industrial benchmarks should be made cautiously.
Details of module fabrication also require clarification:
• The type of laser and parameters for P1, P2, P3 steps are not specified.
• The encapsulation process is insufficiently described for reproducibility; a schematic of encapsulation steps and interconnections would be valuable.
• Precursor solution protocols are inconsistently reported: the first recipe provides exact masses, while wide-bandgap and mini-module formulations use only nominal concentrations. For reproducibility, absolute quantities (mg or mmol) should be included.
7. Manuscript Structure
The final section, currently titled “Discussion,” functions more as a summary of results. Unless expanded to provide deeper synthesis, it should be retitled “Conclusions” or “Discussion and Conclusions” in line with Nature editorial standards.
Recommendation: Major Revision
The manuscript is highly significant, but the above points should be addressed to ensure methodological rigor, interpretive caution, and reproducibility. With these revisions, the manuscript would be considerably strengthened and well positioned for publication.

Referee #2

(Remarks to the Author)
In this work, the authors explored four amino-based derivatives named TFEA (without conjugated core), TFBA (with a benzene ring), TFPA (pyridine), and TFPmA (pyrimidine) in perovskite solar cells. The importance of stereoelectronic manipulation of ligands is evoked where (i) the integration of N-heteroaromatic motifs into ligands, reorient the molecular adsorption from vertical to planar configuration with respect to the perovskite surface. (ii) This planar interfacial configuration enhances the bonding strength and reduces the charge transport distance. (iii) The description of multivariate analysis (Figures 3d, S12, and S13) is valuable. We have the tendency to foresee a monolayer and full coverage of passivating molecules on perovskites. However, in this work a rather poor coverage of passivating molecules (<50% coverage) is experimentally revealed, which still has impressive impact on device performance. As proof-of-concept, the device performances achieved impressive efficiency with the certified PCE of 26.98%. The authors also performed a thorough and comprehensive characterization, which certainly warrants its publication in Nature. However, I still had a few questions reading the manuscript that would like to receive feedback from the authors.

1. Clarification is needed regarding the statement of “certified” in the main text, which tend to link with the NREL chart.

Otherwise, stating "third-party" institution would be more appropriate to avoid confusion to readers at the first glance of the work.

2. The concept of stereoelectronic manipulation is well-proposed. However, looking at the bottom charge contact layer, the authors employed 4PADCB molecules on FTO, which have a long alkyl chain. 4PADCB seems to be not ideal considering the emphasis of stereoelectronic manipulation concept in this work. The authors are invited to comment on the point above, and whether 3PATAT-C3 molecules [J. Am. Chem. Soc. 145, 7528-7539 (2023). https://doi.org/10.1021/jacs.3c00805], for example, would be ideal for further enhancing the performance of devices?

3. The DFT calculations are based on Pb-I terminated perovskite surfaces (Figure 1) for describing the Pb-N and Pb-I-pi interactions. I wonder regarding the molecular interactions and their orientations considering the FA/MA-I terminated surfaces, which is an important consideration leading to different chemical interactions. Please see [1] Adv. Mater. 36, 2310203 (2024). https://doi.org/https://doi.org/10.1002/adma.202310203; [2] Nat. Commun. 16, 753 (2025). https://doi.org/10.1038/s41467-025-56068-6; [3] ACS Nano 10, 1126-1131 (2016). https://doi.org/10.1021/acsnano.5b06420.

4. Please provide explanation and reference(s) validating the rationale of NMR experiments for studying the chemical interaction of passivating molecules (TFEA, TFBA, TFPA, and TFPmA) with PbI_2 in liquid states (i.e., in d6-DMSO). Are these interactions equivalent to those on the actual perovskite solid films with passivation molecules?

5. Please provide details of experimental conditions for FTIR results (Figure 2d). Description of "blue", "mid", and "red" shown in Figure 3c is lacking in the main text.

6. The poor coverage less than 50% of passivating molecules described in this work is puzzling. I wonder if there are monolayers of the passivating molecules covering the entire perovskite surface, which cannot be detected due to sensitivity limitations of the instrument? At the same time, agglomeration of passivating molecules forms, which can be detected by authors' instrument due to signal enhancement. Authors are invited to comment on the sensitivity of the instrument for generating Figure 3d and perhaps adjust the wordings of "~7.7% coverage … TFPmA, ~46.3%"?

7. The claim of "TFPmA achieved intra-grain uniformity, reducing aggregation size by 42% versus TFEA (~57 vs. ~134 nm), establishing a conformal passivation network that suppresses interfacial traps." needs clarification. Authors provide sizes of aggregated features from TFEA (~134 nm) and TFPmA (~57 nm) images in Figure 3d. The authors are invited to provide the analysis based on statistics. Analyses based on single images seem not conclusive as the image sizes are small (scale bars are 60 nm).

8. Showing only the Pb 4f and I 3d spectra (Figure 2a,b) is not sufficient to claim on the charge redistribution of Pb-I frameworks. Authors are invited to provide the N 1s region of the corresponding TFEA, TFBA, TFPA, and TFPmA molecules after deposition on the perovskite layers. Please provide the additional C 1s and F 1s regions for the above measurements in SI as it could provide important database for readers.

9. A brief discussion on how the conduction band minimum values were extracted for generating Figure 3e is needed based on Figures S15-17. Are the VBM regions in Fig. S15 plotted in linear or log scales? Would it make more sense to represent Figure 3e aligning the Fermi levels of individual functional layers? Please provide authors' rationale for such a vacuum level alignment representation of the device structures.

10. The figure caption text in Figure 4 needs revision. The corresponding figure caption text does not match with the actual individual panels in Figure 4.

11. Please state explicitly the nominal perovskite composition at the beginning of the corresponding text employed for the fundamental characterizations (e.g., Figs. 2a, 3, and 4). Also, in Figure 5a and in the text, please state the nominal perovskite composition in "FTO/4PADCB/Perovskite/ligands/C60/BCP/Ag".

12. Authors are suggested to provide a discussion on hysteresis in the main text (e.g., Figs 5b, 5d). If the authors can present the actual data (J-V of forward and reverse scans), it would be informative for readers (e.g., Fig. S32).

13. Splitting Figure S33 into two pages are recommended so readers can access the information in its contents.

14. Citation of Supplementary Notes 1 to 3 in proper locations in the main text would help readers to access directly the complementary information provided by the authors.

15. Discussion of "PGD (mW/cm^2)" graph shown in Figure 5i is needed.

Referee #3

(Remarks to the Author)
A. Summary of Key Results
The manuscript "Stereoelectronic Manipulation of Ligands Toward Shockley-Queisser Limit of Perovskite Solar Cells" by Yang and coworkers presents a systematic study on the stereoelectronic design of passivation molecules for the perovskite/ETL interface in p-i-n single junction solar cells. Using ESP and DFT for theoretical calculations, to spectroscopic analysis for passivant-perovskite precursor interactions, to various thin film characterization and loss analysis, the paper

ends in presenting a highly efficient single-junction perovskite solar cell with record fill factor on small device area for the target passivation molecule. The approach is tested also for a higher bandgap perovskite absorber suitable for tandem application and integrated in a minimodule. Improved stability data for the target compared to the reference is presented, too.
The molecular design study presents the combination of an amido-moiety for defect passivation, an -CF3 hydrophobic moiety for improved environmental stability with a systematic variation of a central unit ranging from benzene to N-heteroaromats pyridine and pyrimidine. Their main finding is that the introduction of N-heteroaromats yields instead of a vertical a planar interaction with the absorber surface allowing for improved chemical passivation of surface defects but further improves charge transport.
With their work the authors contribute to the field of emerging PV technologies presenting valuable insights into the molecular designs of interfacial passivants as well as a (in-house) record for perovskite solar cells with 27.58% (certified 26.98%). This is on pair with the current certified record of 27% published in the efficiency tables and the NREL chart. I would like to congratulate the authors for this achievement.
Overall, the manuscript is well-written and the quality of the manuscript is high. However, certain areas of the manuscript require further clarification, particularly with the framing of the paradox of molecular design, the novelty of the N-heteroaromatic inclusion, as well as details on the perovskite bulk material and its surface termination. Further some points on language and wording are recommended to reconsider.
I recommend major revisions and think that addressing the points outlined below can help to strengthen the manuscript further to be considered for publication in Nature.

B. Originality and Significance
The authors address a hot topic in the perovskite field with regard to high-efficient perovskite solar cells. The systematic study allows to give more understanding on design rules for interfacial passivation at the perovskite/C60 interface.
However, the authors should make clear which existing literature on planar aligning passivation molecules and which on N-heterocycle containing ones have been reported already in literature. Please make the originality and novelty of the paper more clear.

C. Data and Methodology
The experimental design is generally sound and the broad methodology applied in the paper is definitely a strength. Figures are clear and informative, though Figure 2d could benefit from improved description (colored areas, numbering), as well as full axis labeling in case of Figure 3c, d. Further the inset graph in Figure 4b is too squeezed. In Figure 5 f q Voc instead of Voc Energy as axis label.

D. Use of Statistics and Treatment of Uncertainties
With respect to the little amount of statistics shown in the perovskite field in general, authors consider an appropriate level of data sets and various methods to backup trends/derive findings.
However, authors are encouraged to talk more about errors and reconsider if it is reasonable to state current density values in mA/cm2 with to digits after the comma, similarly the FF.
For outdoor measurements it would be helpful that also the control group is shown as comparison.
A mismatch correction based on SR reference cell and perovskite solar cells needs to be calculated and considered for absolute IV values. Please add EQE data for control and target cell, best would be even for all passivant groups.

E. Conclusions
The conclusion is robust and supported by the broad set of data.
It is recommended that the authors consider also the role of dipole changes at the interface in the light of field effect passivation and impact on charge distribution within the bulk material (doi.org/10.1002/aenm.202201109, doi: 10.1126/science.adx1745, etc).

F. Suggested Improvements
Major
- The core problem presented is named as “fundamental design paradox”, however it seems rather a challenge then a real paradox. The fact that two parameters needs to be optimized at a time (or more) seems not enough; the qthor should clarify rethink the wording/presentation of the research challenge.
- Linked to this point clarification on existing literature on conjugated structures and other planar aligned passivation molecules should be added. As mentioned above the novelty should be clarified.
- The authors talk about defect passivation and name Pb-N and Pb-I-pi interaction, though when stating in the text no direct references are given. As the paper does not address the bulk perovskite at least literature references should be added. Connected to this, generally little info on the perovskite absorber is given. However, the interaction of the molecule is very much dependent of the perovskite crystal termination and should be thus described more. Please add a chemical formula for the perovskite composition (for all perovskites used) and clarify why (only) Pb-I termination has been looked at, e.g. in the DFT study. Would the molecule effect be similarly on MA-terminated surfaces?
- A mismatch factor should be considered for such high-efficiency values to allow fair comparison. Info on how the certified measurements were conducted in terms of considering a SR mismatch factor is recommended.
Minor
- Please reconsider to change the word manipulation to design in the title.
- In the abstract the wording “conjugated backbone of ligands” is used, more precise naming/description could help the reader
- The wording sub-nanometer charge transport pathways seem misleading as KPFM and nano-IR maps indicate inhomogeneous formation of the vertical aligned passivants on the perovskite surface. Wouldn´t this also allow sub-nanometer charge transport channels?

- Please add references in the introduction when naming ranges for lifetime, trap density, transmission coefficient, series resistance.
- Please check the wording “intercalation” of amine terminal into the Pb-I framework.
- It is not clear what is meant by “atomic-scale proximity to the SQ limit”. Can the authors please clarify/adapt the wording.
- Please check/clarify the wording “ambidentate” (four ambidentate ligands were engineered…) as besides the amidine group the -CF3 group is mentioned. Is this a functional group that can be considered a “dent”. Not a classical lewis base/acid to donate/accept free electrons.
- Generally authors are encouraged to check their manuscript for easy accessible language. Some sentences could be improved, e.g. “This dichotomy creates a fundamental material design paradox” or “QFLS analysis quantified the interfacial defect suppression hierarchy,…..arises from a tripartite cooperation with topographic defect healing via multicenter interactions”.
- In the same paragraph the wording bandgap homogenization is used, but the bandgap had not been named described so far in the text. Please clarify pi-sigma interaction and name bandgap and shifts for use of passivants.
- The bandgap of the absorber/formula should be mentioned early in the text.
- As mentioned it is recommended to add a control group for stability testing outdoor and EQE graphs for a cell per group using the passivant variation.
- Please add more info on sample preparation, e.g. H-NMR, FRIT, and always clarify which kind of sample stack was used, e.g. XPS, XRD, PL,etc.
- Please check carefully that claims are backed up with linking Figures or adding references. E.g. “effectively dissipating charge extraction differences from grain boundaries and grains”, please clarify to which data or reference you refer to; or in “optimizing ligand adsorption topology enables the establishment of sub-nanometer charge transport pathways and stronger pero/ligand/C60 electron coupling, leading to more efficient charge extraction” the data on charge extraction has not been shown yet, so rather link/refer or change wording to prerequisite for efficient charge extraction.
- Please reconsider the wording “underscoring operational robustness” in the conclusion. Though the stability data looks promising compared to the control device, no comparison for the outdoor data is given and 130 days with ~7% efficiency loss is still far from what would be needed for “commercial-scale modules” as named in the sentence after. A critical few on what still needs to be mproved in this regard is recommended.

G. References
More references with regard to the molecular design would be recommended to clarify novelty, as mentioned above. Also consider including recent reviews on passivant design to strengthen the introduction.

H. Clarity and Context
The manuscript is concise but as mentioned the abstract could be sharpened in terms of clear naming of the molecular moieties varied, the language could be simplified or clarified. A critical check of the term fundamental paradox is recommended paired with a clarification of existing literature. A clear statement of the hypothesis is recommended after checking this. As mentioned above check the appropriate wording for stability data in the conclusion.

Version 1:

Reviewer comments:

Referee #1

(Remarks to the Author)
I really appreciate the extensive effort made by the authors in revising the manuscript and providing detailed responses to all previous comments. The manuscript has clearly improved and many of the initial concerns have been satisfactorily addressed. In particular, the revised computational methodology strengthens confidence in the reported energetic trends. The expanded XPS analysis and control experiments convincingly rule out alternative interpretations related to stoichiometry or charging. The moderation of previously overstated mechanistic claims is appreciated and results in a more balanced interpretation of the data. The additional stability data significantly improve the practical perspective of the work. However, a few aspects would still benefit from minor clarification before final acceptance:
1. Cross-technique consistency:
While the authors have clarified the coexistence of Pb–N and Pb–I–π interactions, the quantitative weight of each contribution remains somewhat qualitative. A short clarifying paragraph in the main text summarizing the relative contribution inferred from the different techniques would improve coherence.
2. Statistical treatment of the devices:
The statistical treatment of the device data appears generally sound, and the reported improvements are substantial. However, for completeness, the authors should clarify whether the reported uncertainties correspond to standard deviation or standard error of the mean and explicitly state the number of independent devices (n) in the relevant figure captions. Additionally, for the impedance spectroscopy analysis, providing the equivalent circuit model and reporting fitting uncertainties would further strengthen the quantitative rigor of the study.
3. Energetic interpretation:
The electrostatic explanation for the UPS shifts is reasonable, but it would benefit from a clearer schematic energy-level diagram in the main text to guide the reader.
These are relatively minor points that do not undermine the central conclusions of the work. If the authors address these clarifications appropriately in the revised manuscript, I do not consider a further round of review necessary. Overall, the manuscript is now substantially improved and, upon satisfactory implementation of the above minor revisions, can be

considered suitable for publication.

Referee #2

(Remarks to the Author)
The authors have made significant efforts to address my previous concerns, and I appreciate the additional clarity that the authors incorporated to the manuscript. After reading the revised files, I have additional minor questions, which I would appreciate the feedback from the authors.

1. Page 9, Lines 8-9: Authors state “dissipation of surface-aggregated PbI2 domains after interfacial treatment” from Supplementary Fig. 17 images. How can authors identify PbI2 domains solely from the SEM images? Can authors consider adding justification/clarifications in the caption of Supplementary Fig. 17 for the above statement?

2. Page 9, Lines 9-11: Authors state “atomically smooth interfaces with ~1.4-1.7 nm reduced RMS roughness” from Supplementary Fig. 18. The histograms in Supplementary Fig. 18 seems to provide similar height distribution in the range of -40 to +40 nm. Please provide in the caption of Supplementary Fig. 18 the rationale how the range of reduced “~1.4-1.7 nm” was calculated.

3. Supplementary Fig. 23 and Fig. 3c are repeating. Please confirm whether Supplementary Fig. 23 can be replaced by the “statistical analysis” (mentioned in the caption) based on Fig. 3c and Supplementary Table 1(?).

4. In the Methods section (1.5 Film characterization), please provide further details with equations for the sample work function calculations from CPD values based on KPFM measurements (Fig. 3a). This would make clearer for readers when authors compare with work function values determined from the UPS results (Supplementary Fig. 24 and Supplementary Table 2).

5. Because authors provide the CPD distribution (Fig. 3b, Supplementary Fig. 19), the standard deviation estimation for the CPD values presented in Fig. 3a could be provided.

6. Just to make sure I’m understanding correctly: In Fig. 3d, by changing the color of spectrum labeled as “mid” to green color, would it make easier for readers to directly correlate with the corresponding mapping color shown in Supplementary Fig. 21?

7. The field testing under actual outdoor operating conditions are another novelty point in this work. This is not to criticize because of the known challenges involved in the measurements (e.g., weather variations) and analysis. However, I was curious in understanding the variations in the PCE shown in Fig. 5g. From the starting (t = 0) until ~18 days, the PCE seems to keep decaying (from ~24% to 18%), but afterwards, the PCE start to increase and show a rather stable PCE profile. When I compare with the PCE profile of the control sample shown in Supplementary Fig. 53, similar decay (PCE from ~21% to ~16%) is also observed for the first 27 days. I was wondering whether the control sample is also showing similar behavior of PCE recovery after the first 27 days, and it is related to the initial burn-in process. If authors could provide insights for the above point, and further additional insights/discussion from the authors’ experiences/challenges in the outdoor testing would be valuable for the readers to attract further attention.

8. Page 3, Line 7:“are/fundamentally”

9. In the Solar Cells Reporting Summary, the check list regarding to “Current density-voltage (j-V) plots in both forward and backward direction” needs to be updated. Instead of Fig. 5b, is it Supplementary Fig. 38?

Referee #3

(Remarks to the Author)
The authors have responded in a very careful manner to the reviewer´s question, providing additional data, explainations, and references. Answers are satisfactory and the quality of the manuscript has further increased. One point which stays unclear/critical to me is the different, not expected shape of the third party EQE measurement (compared to reasonable shape inhouse data) that can influence setting of lamps/ mismatch factor calculation and thus certified values under 1.5AMG standard testing conditions. It would be recommended to resolve this issue, other then that the paper is meeting the level for publication.

Open Access This Peer Review File is licensed under a Creative Commons Attribution 4.0 International License, which permits use, sharing, adaptation, distribution and reproduction in any medium or format, as long as you give appropriate credit to the original author(s) and the source, provide a link to the Creative Commons license, and indicate if changes were made.
In cases where reviewers are anonymous, credit should be given to 'Anonymous Referee' and the source.
The images or other third party material in this Peer Review File are included in the article's Creative Commons license, unless indicated otherwise in a credit line to the material. If material is not included in the article's Creative Commons license and your intended use is not permitted by statutory regulation or exceeds the permitted use, you will need to obtain permission directly from the copyright holder.
To view a copy of this license, visit https://creativecommons.org/licenses/by/4.0/

We are very pleased that all three reviewers found our manuscript interesting and novel. We are grateful to them for their constructive comments and valuable suggestions, which have helped us to significantly improve the manuscript. In response, we have carried out additional experiments and analyses and have revised the manuscript accordingly. We hope that the reviewers will find the revised version suitable for publication.

List of point-to-point response of reviewers' comments

Referee #1 (Remarks to the Author):

The manuscript entitled "Stereoelectronic Manipulation of Ligands Toward Shockley–Queisser Limit of Perovskite Solar Cells" by Tinghuan Yang et al. presents a stereoelectronic molecular design strategy for interface engineering in perovskite solar cells (PSCs), achieving near-theoretical Shockley–Queisser efficiencies with record-level certified PCE and excellent outdoor stability. Specifically, the authors embed nitrogen atoms into conjugated ligand backbones to enable dual Pb–N coordination and Pb–I–*π* interactions, driving planar adsorption geometry. This strategy reconciles two traditionally antagonistic requirements—atomic-scale defect passivation and minimized transport distance—leading to 87.91% of the SQ limit, a certified PCE of 26.98% (27.58% lab), and 92.5% retention after 130 days of outdoor testing.

The work is conceptually novel, methodologically rigorous, and of broad potential interest to the photovoltaics and materials science communities. It addresses a fundamental bottleneck in PSC performance and stability and is supported by strong experimental evidence and theoretical investigations. While the manuscript is strong and timely, by demonstrating high performance, and real-world stability, several issues require clarification, improved contextualization and in some cases tempering of claims before the manuscript can be considered further. I therefore recommend major revision. My detailed comments are provided below.

1. Computational Methodologys

The computational workflow is described with sufficient detail, combining molecular-level calculations (Gaussian 16 at the B3LYP-D3BJ/def2-TZVP level with frequency analysis) and periodic perovskite/ligand models (VASP with PBE-GGA, PAW potentials, 450 eV cutoff, Γ-centered k-point sampling, and DFT-D3 dispersion corrections). The adsorption and binding energies are well defined, and the structural optimizations appear carefully conducted.

Nevertheless, some concerns remain regarding the **quantitative reliability** of the periodic DFT simulations:

• Γ-point only sampling (1×1×1): While this is sometimes acceptable for large supercells, Pb–I perovskite frameworks are sensitive to Brillouin zone sampling, and Γ-only calculations may not capture the electronic redistribution accurately. A denser k-point grid (e.g., 2×2×1 or higher) would improve reliability.

• Plane-wave cutoff (450 eV): This value may be marginal for Pb/I-containing systems,

where higher cutoffs (≥500 eV) are often required. Considering that the reported adsorption/binding energy differences are on the order of 0.1–0.2 eV, the current settings may not provide fully converged values.

• Energy interpretation: The manuscript interprets trends in ligand stabilization based on small differences in adsorption/binding energies. Given the computational setup, such differences could be within the numerical uncertainty of the method, and caution should be exercised in drawing strong conclusions.

• Reference consistency: It should be clarified whether identical supercell dimensions, cutoffs, and k-point sampling were applied to both isolated ligands and combined systems, to avoid spurious contributions from vacuum-size or strain effects.

***Response*:** We are grateful to you for the valuable suggestions regarding the computational methodology. In response, we have **revised** the DFT setup to strengthen the calculation's reliability: the k-point grid was expanded to 2×2×1 and the plane-wave cutoff energy was increased to 500 eV. The **recomputed results** (now shown in **Figure R1**) better support our conclusions, and we appreciate your guidance in improving this aspect of the work.

(1) Based on the updated computational model, we recalculated the adsorption energies of four ligands on the perovskite Pb–I framework surface in both vertical and planar configurations (**Figure R2**). In the vertical configuration, the adsorption energies of TFEA, TFBA, TFPA, and TFPmA are −0.97, −1.16, −1.13, and −1.10 eV, respectively. In the planar configuration, the corresponding adsorption energies are −0.40, −1.15, −1.31, and −1.37 eV. We further evaluated the energy difference defined as $\Delta E = E_{ads}$ (planar) − E_{ads} (vertical), yielding values of 0.57 eV (TFEA), 0.01 eV (TFBA), −0.18 eV (TFPA), and −0.27 eV (TFPmA). Recalculation yields values and a trend **in consistency with** the original manuscript with reported values (Δ≤0.05 eV) and trend in the original manuscript, confirming the robustness of the calculations and supporting a ligand adsorption configuration transition.

(2) Based on the updated computational model, we also recalculated the interfacial binding energies of the perovskite/ligand/C_{60} interface (**Figure R3**). When the ligands adopt a vertical configuration (e.g., TFBA), the interfacial binding energy is −0.45 eV, whereas a planar configuration (e.g., TFPmA) yields a more negative interfacial binding energy of −0.59 eV. These results indicate that the planar ligand configuration affords stronger interfacial binding. The recalculated values **are consistent with** those reported in the original manuscript, confirming the reliability of the calculations and further supporting that the planar configuration provides the most favorable interfacial energetics.

(3) Notably, for TFBA, ΔE is nearly zero in both original and updated calculations (~0.04 and 0.01 eV), indicating no strong configurational preference. Accordingly, we have revised the manuscript text from "Using TFBA in its thermodynamically favored vertical topology" to "Using TFBA in a vertical topology" for greater precision (**Page 5**, in the revised manuscript).

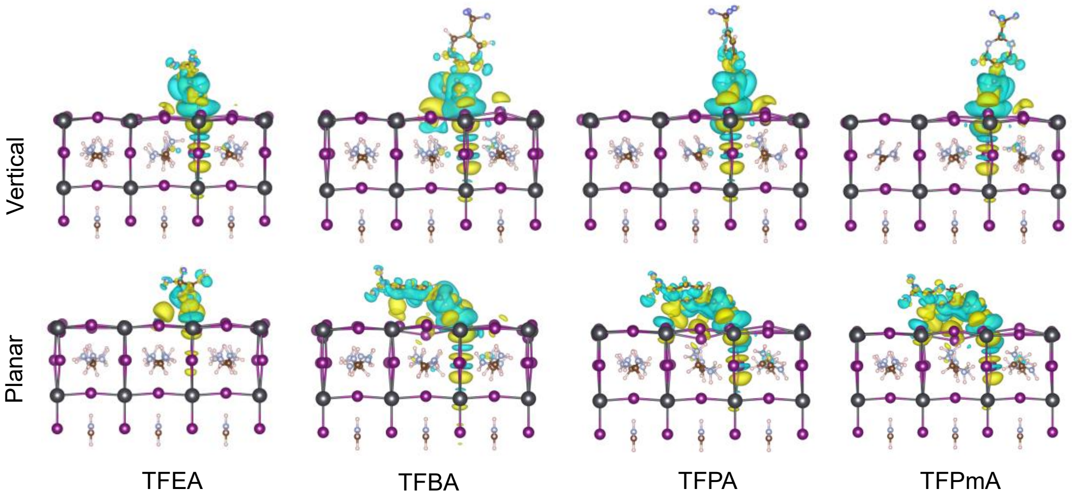

Figure R1. The adsorption configurations of different ligands on the Pb-I terminal of perovskite after expanded the k-point grid to (2×2×1) while increasing the Plane-wave cutoff to 500 eV.

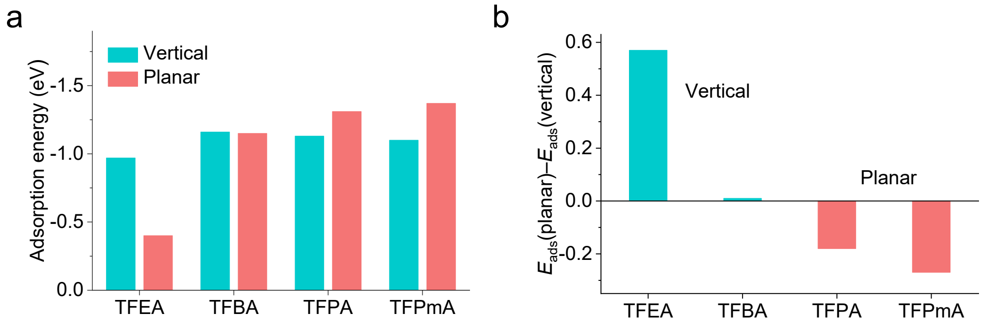

Figure R2. The adsorption energy of different ligands on the Pb-I terminal of perovskite after expanded the k-point grid to (2×2×1) while increasing the Plane-wave cutoff to 500 eV.

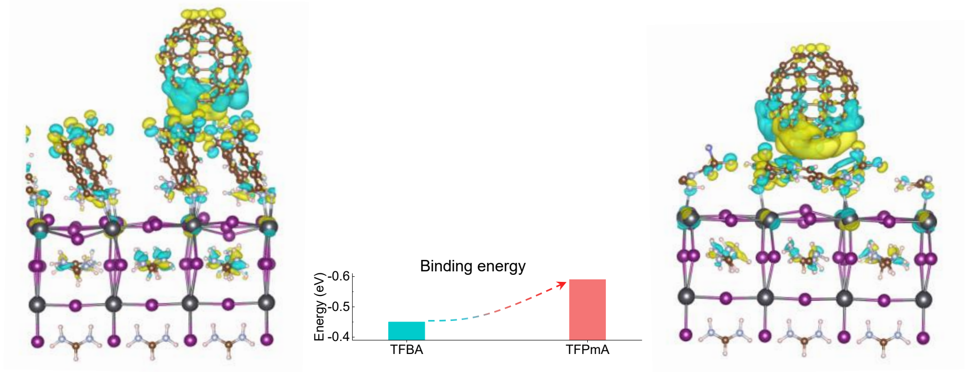

Figure R3. The binding energy of perovskite/TFBA/C_{60} and perovskite/TFPmA/C_{60} based on the Pb-I terminal after expanded the k-point grid to (2×2×1) while increasing the Plane-wave cutoff to 500 eV.

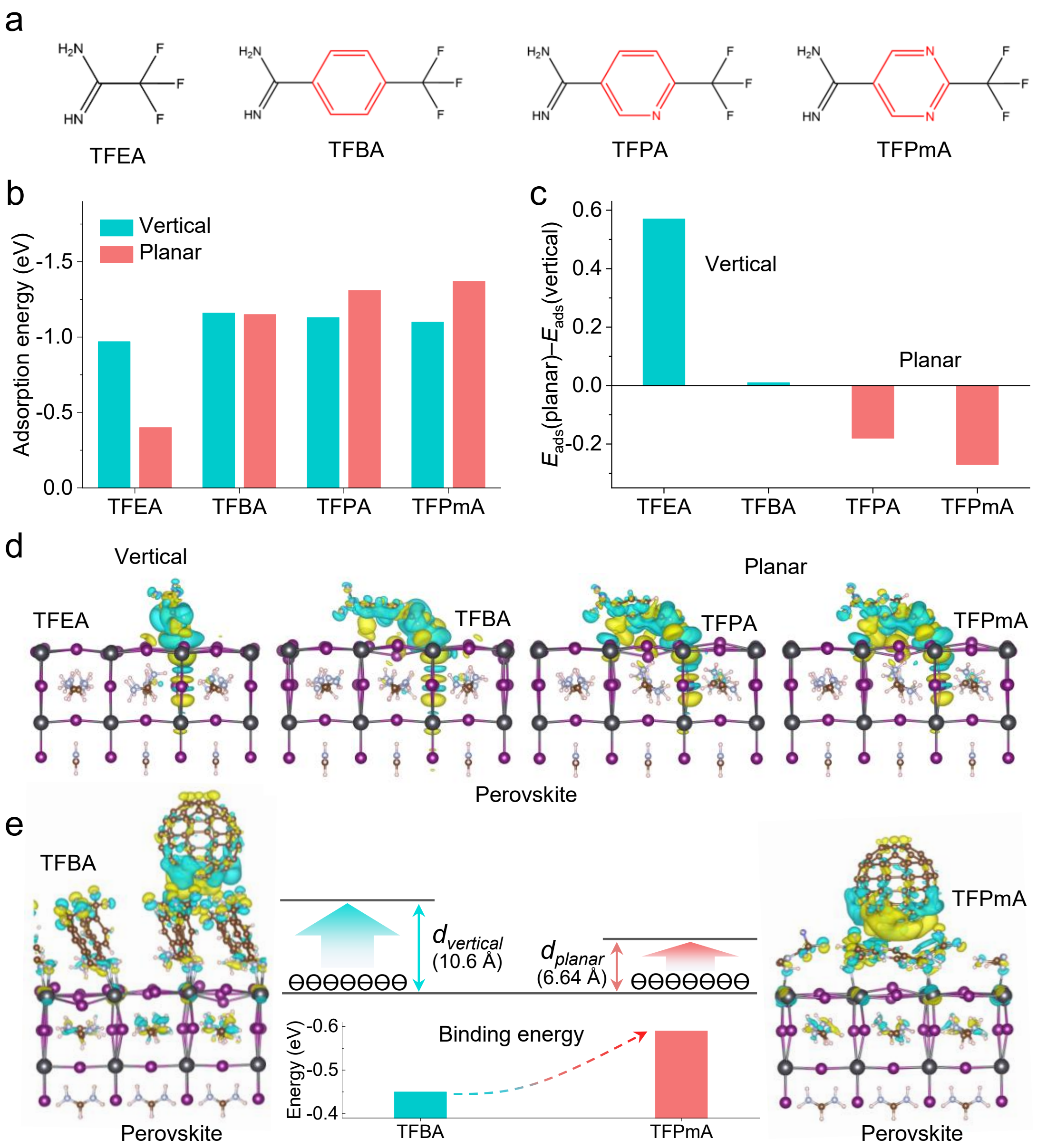

Figure R4. Ligand structures and adsorption topologies on the perovskite surface. (a) Chemical structures of the four ligands. (b) Adsorption energies of each ligand on the perovskite surface in vertical and planar topology. (c) Formation-energy differences between vertical and planar adsorption topology for each ligand on the perovskite surface. (d) Differential charge-density maps for ligands adsorbed in vertical versus planar topology, illustrating regions of charge accumulation and depletion. (e) Differential charge-density maps, binding energies, and charge transport distance of perovskite/ligand/C_{60} interfaces with TFBA or TFPmA. (**Updated Fig. 1**, in the revised manuscript)

(4) In summary, the recalculated results are consistent with our original data. Per your request, we have uniformly updated the DFT model throughout the manuscript; please refer to the **updated Fig. 1 (Figure R4)** for details.

"Vertically adsorbed E_{ads} values were –0.97 eV (TFEA), –1.16 eV (TFBA), –1.13 eV

(TFPA) and –1.10 eV (TFPmA), whereas planar adsorption energies were –0.40 eV, –1.15 eV, –1.31 eV and –1.37 eV, respectively (**Fig. 1b**, **Supplementary Fig. 3**). The difference ΔE = E_{ads}(planar) – E_{ads}(vertical) decreased from 0.57 eV (TFEA) to almost zero 0.01 eV (TFBA) and became negative for TFPA (–0.18 eV) and TFPmA (–0.27 eV), indicating a thermodynamic preference shift from the vertical (TFEA) to random (TFBA) and then planar (TFPA, TFPmA) topology as the number of N-heteroaromatic units increases (**Fig. 1c**).” (**Page 5**, in the revised manuscript)

“and the plane wave cutoff energy was 500 eV.” (In the **Supplementary Note 1**)

“the Brillouin zone integration was performed with a 2 × 2 × 1 Gamma centered sampling.” (In the **Supplementary Note 1**)

“On Pb–I–terminated surfaces, the formation-energy differences (ΔE = $E_{ads(planar)}$ − $E_{ads(vertical)}$) for TFEA, TFBA, TFPA, and TFPmA decrease sequentially from 0.57 to 0.01, −0.18, and −0.27 eV, respectively. For TFBA, ΔE is close to zero (0.01 eV), suggesting no strong configurational preference between vertical and planar adsorption. This trend indicates a gradual thermodynamic shift in preferred adsorption topology from predominantly vertical (TFEA), to nearly non-preferential or mixed configurations (TFBA), and further toward planar geometries (TFPA and TFPmA) as the number of N-heteroaromatic units increases.” (In the **Supplementary Note 1**)

2. Spectroscopic Evidence

The additional experimental evidence significantly strengthens the mechanistic claims of stereoelectronic ligand regulation at the perovskite/CTL interface. Nevertheless, a few aspects require clarification and/or further support before the conclusions can be fully substantiated:

XPS Data Interpretation: The observed monotonic binding energy shifts in Pb 4f and I 3d peaks are attributed exclusively to Pb–N coordination and Pb–I–π interactions. However, XPS binding energies can also be influenced by changes in surface stoichiometry, defect concentration, or sample charging. The authors should discuss these alternative contributions, clarify whether peak deconvolution was performed, and describe how reproducibility was ensured.

***Response*:** Thank you for your insightful comment regarding potential confounding factors (surface stoichiometry, defects, charging) for the XPS binding energy shifts. We agree that this requires careful evaluation and have conducted additional experiments to clarify this issue.

(1) To isolate the effect of surface stoichiometry, we prepared three perovskite films with varying surface Pb/I ratios: a control with +7% excess PbI_2 (as per the manuscript protocol), one with +15% excess PbI_2, and one with a -5% PbI_2 deficiency. All samples were transferred in a sealed, nitrogen-filled chamber to prevent air exposure.

Figure R5 presents the XPS results for these films prior to TFPmA ligand treatment. Compared to the control, the +15% excess PbI_2 film showed only minor shifts (< 0.15

eV) in the Pb 4f and I 3d core levels. In contrast, the -5% PbI_2-deficient film exhibited a more substantial shift of > 0.3 eV. Given that the stoichiometry was identical for all films before surface treatment, the influence of surface stoichiometry variations can be negligible.

Figure R6 shows the XPS results after TFPmA ligand treatment. This treatment induced a positive binding energy shift in the Pb 4*f* peaks by 0.23 eV, 0.17 eV, and 0.33 eV for the +15% excess PbI_2, control (+7%), and -5% deficient films, respectively. These consistent shifts across different starting surfaces confirm that the observed effect originates from ligand-induced surface modification.

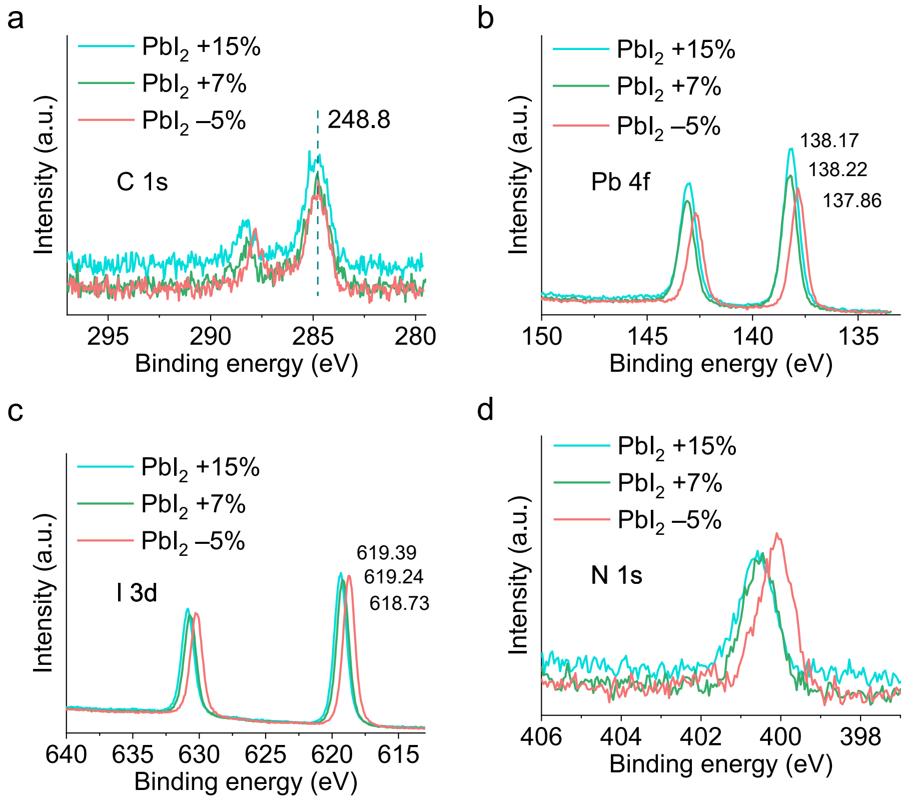

Figure R5 XPS spectra of the three perovskite films. Variations in different surface stoichiometry (i.e., PbI_2 enrichment or iodide deficiency).

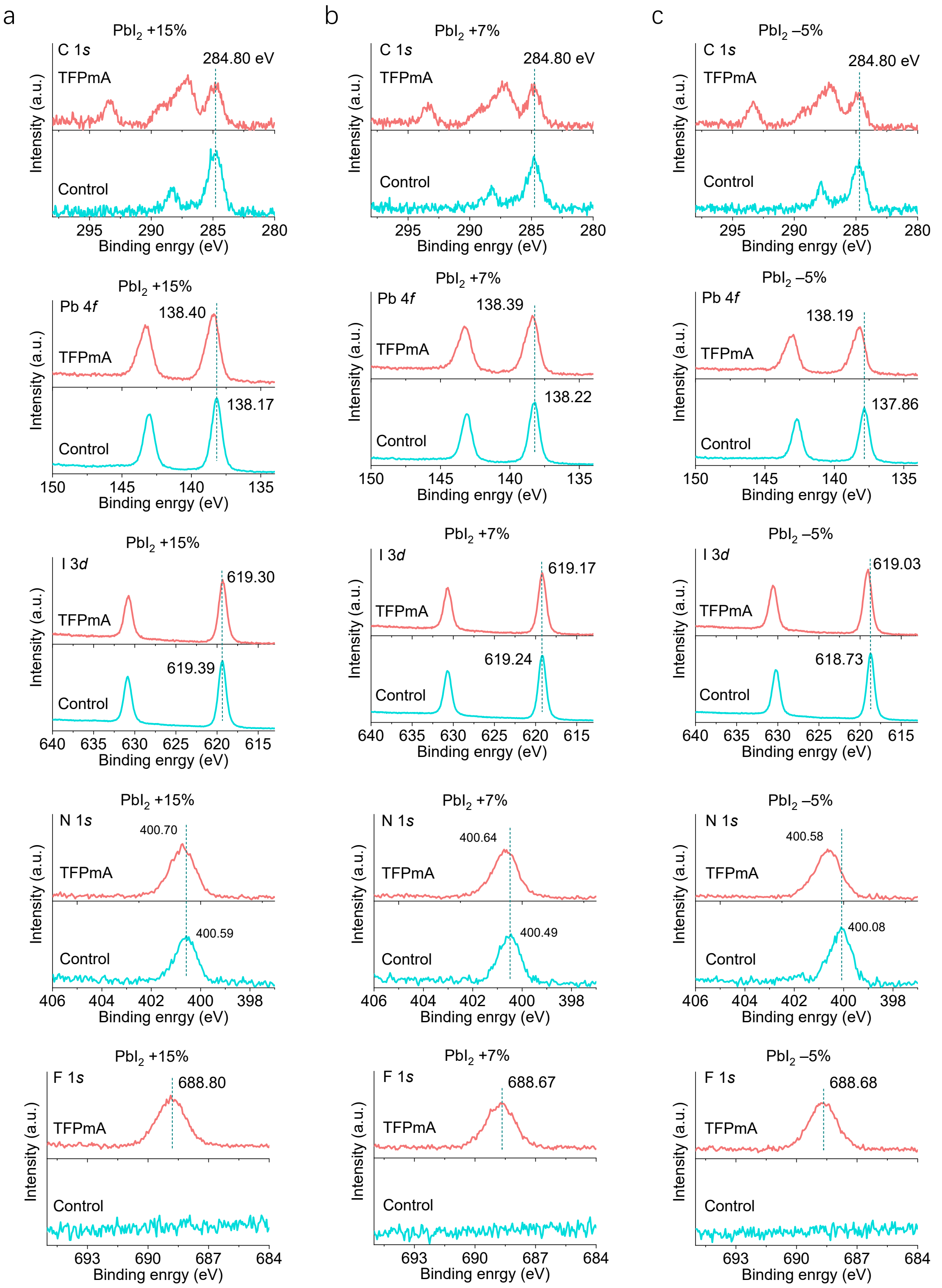

Figure R6 XPS spectra of the three perovskite films (different surface stoichiometry) after surface ligand modification.

In addition, we also checked the impact of precursor composition (e.g., $FA_{0.85}MA_{0.1}Cs_{0.05}Pb(I_{0.9}Br_{0.1})_3$, $FA_{0.9}MA_{0.05}Cs_{0.05}PbI_3$, and $FA_{0.95}Cs_{0.05}PbI_3$). While variations in bulk stoichiometry did induce **minor Pb 4*f* shifts (<0.15 eV)** (**Figure R7**), ligand modification on films of identical composition reproducibly induced a

significantly larger shift of ~0.2 eV in both Pb 4*f* and I 3*d* core levels (**Figure R8**). Since the stoichiometric composition is identical for control and ligand-treated films, the larger shift (~0.2 eV) arises from ligand-induced surface modification, not from bulk compositional changes.

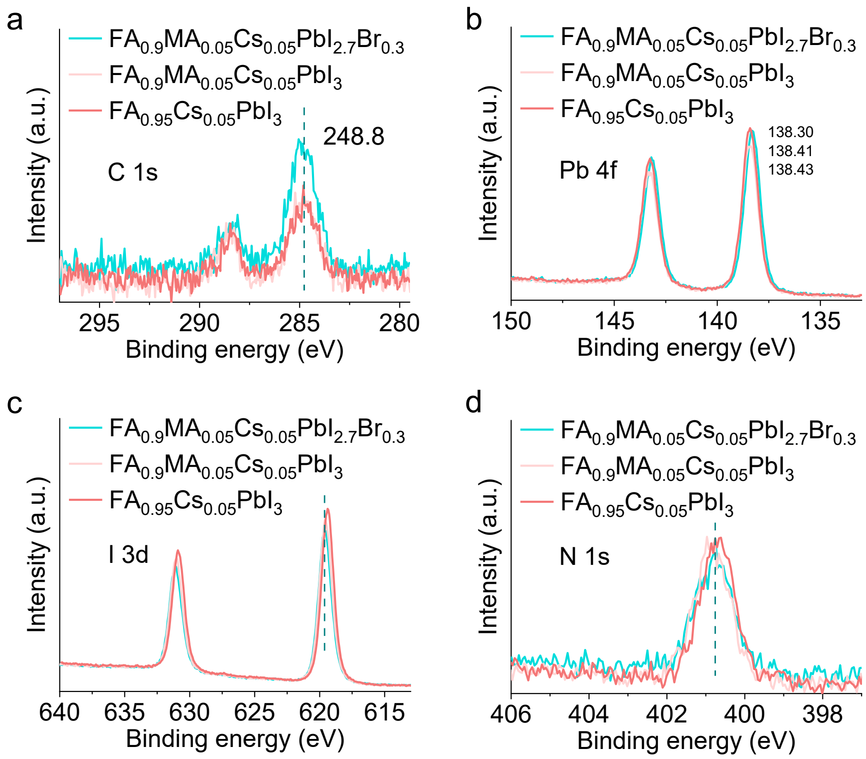

Figure R7. XPS spectra of the three perovskite films. Variations in precursor formulations (i.e., different precursor compositions) lead to **minor Pb 4*f* and I 3*d*** shifts (<0.15 eV) shifts.

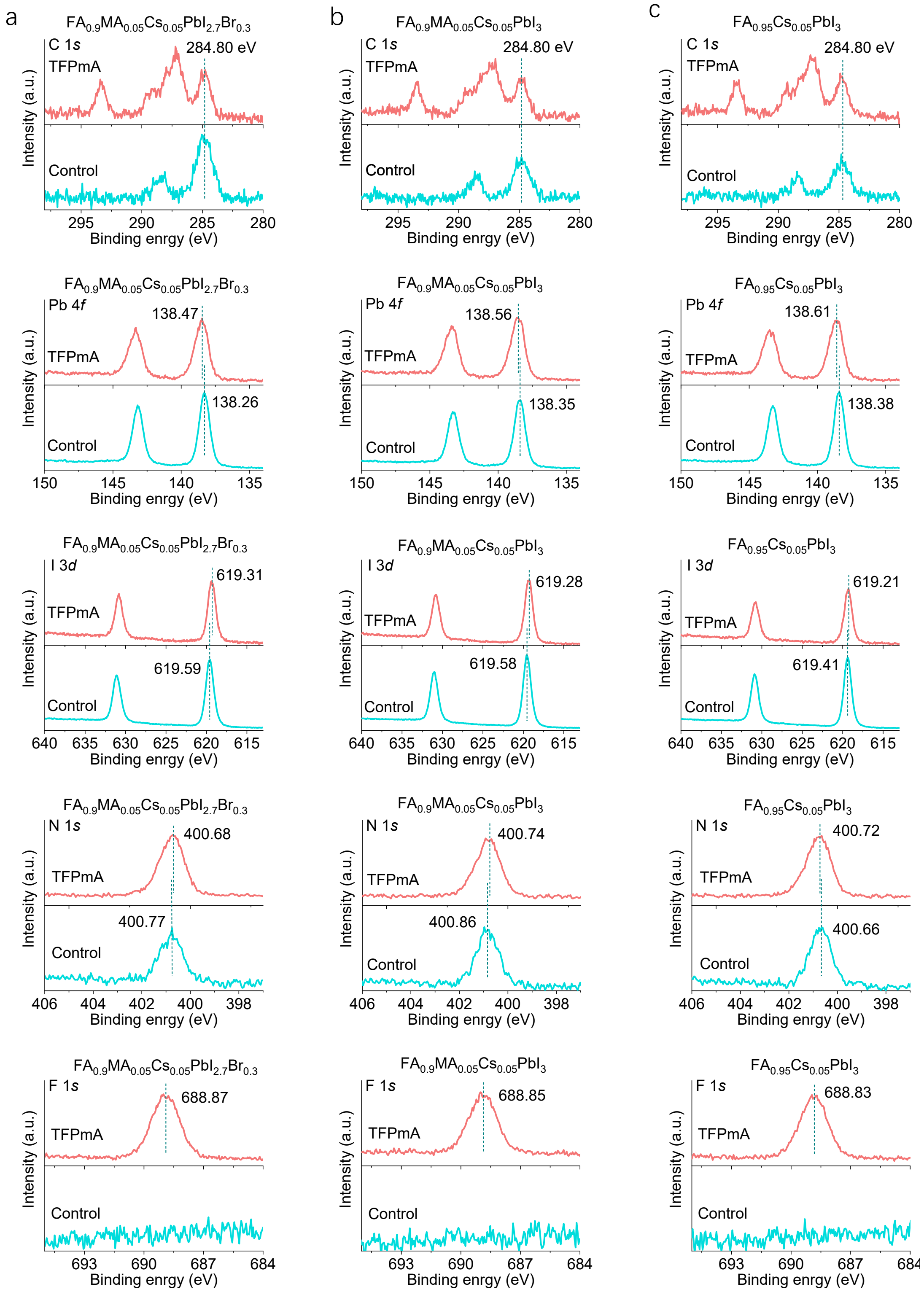

Figure R8. XPS spectra of the three perovskite films (different precursor compositions) after surface ligand modification. All three films exhibit a pronounced binding-energy shift of ~**0.2 eV** in both the Pb 4*f* and I 3*d* peaks following ligand treatment.

(2) We next evaluated the potential influence of defect formation. To introduce defects, unmodified, unencapsulated reference films were aged under continuous illumination at 50 °C for 48 hours. Subsequent XPS analysis (**Figure R9**) revealed

substantial shifts toward lower binding energies for both core levels (Pb 4*f*: 0.28 eV; I 3*d*: 0.48 eV). This pattern is distinct from that induced by ligand modification, where the Pb 4*f* shifts to *higher* binding energy (Δ=0.28 eV) while I 3*d* shifts to *lower* energy (Δ=-0.50 eV). Furthermore, the defect-induced shifts are significantly larger in magnitude, accompanied by a marked decrease in the N 1*s* signal. The opposite direction and reduced magnitude of the Pb 4f shifts in these ligand-treated fresh samples collectively demonstrate that the ~0.2 eV changes originate from intentional surface modification, ruling out defect formation as the primary cause.

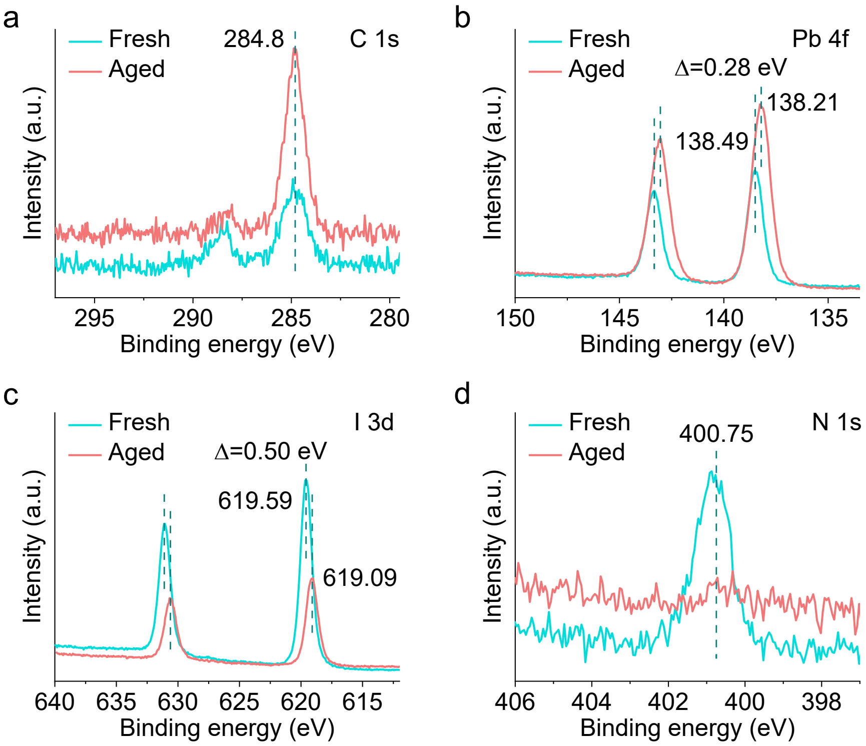

Figure R9. XPS spectra of the aged perovskite films. Upon aging (associated with substantial defect formation), both the Pb 4*f* and I 3*d* binding energies shift markedly toward lower binding energies, accompanied by a pronounced decrease in the N 1*s* signal. These results indicate that surface defects can significantly alter the XPS response.

(3) To exclude the influence from air-exposure, we repeated the sample preparation and XPS measurement three times under rigorous, uninterrupted nitrogen protection. The results (**Figure R10**) consistently reproduced the key signature: ligand treatment induces a shift of the Pb 4f peak to higher binding energy and the I 3*d* peak to lower binding energy. Furthermore, the concomitant shift of the N 1*s* peak to lower binding energy, along with a clear increase in its intensity (**Figure R10e**), provides direct evidence of ligand attachment and strong interaction with the Pb–I framework. This confirms that the observed spectral changes are intrinsic and attributable to ligand bonding, not to surface contamination.

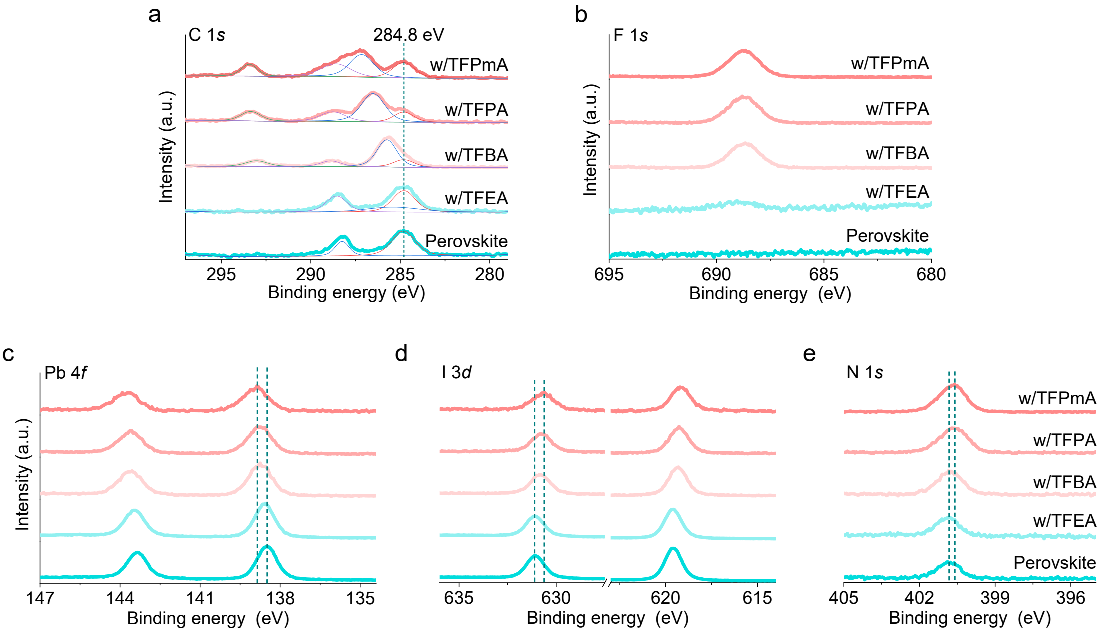

Figure R10. XPS spectra of the ligand-modified perovskite films. The Pb 4*f* peak shifts toward higher binding energy, while the I 3*d* peak shifts toward lower binding energy, both Pb 4*f* and I 3*d* signals show reduced intensity, indicating strong interactions between surface Pb-I framework and the ligand molecules and confirming ligand coverage on the perovskite surface. The N 1*s* peak exhibits a slight shift toward lower binding energy along with increased intensity, consistent with the adsorption of the ligand's N-heterocyclic moieties.

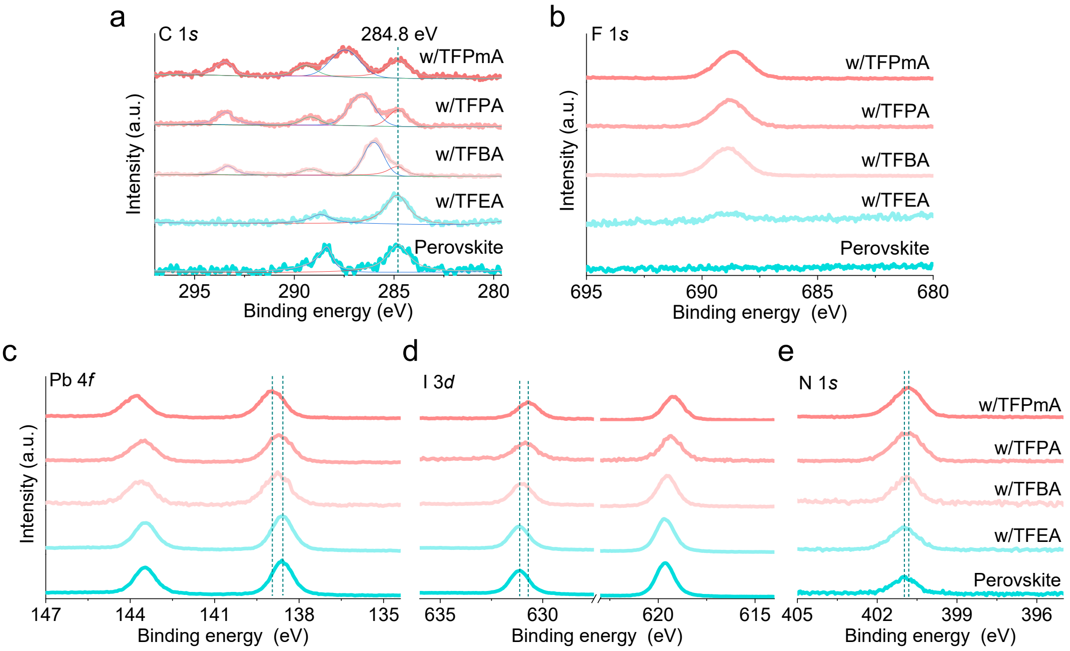

Figure R11. XPS spectra of the perovskite/ligands film after sample charging.

(4) Finally, to assess the impact of sample charging, we intentionally induced charging during an XPS acquisition on a perovskite/ligand film and then repeated the measurement (**Figure R11**). The spectral trends remained consistent with our standard

results (c.f., **Figure R10**): Pb 4*f* shifted to higher binding energy, while I 3*d* and N 1*s* shifted to lower binding energy, with the N 1*s* signal intensity markedly increased. This confirms that the reported shifts are intrinsic and not artifacts of charging, further validating the reliability of our XPS data.

(5) In direct response to the reviewer's comments and supported by these additional validation experiments, we have updated the manuscript accordingly. The main text now incorporates the N 1*s* XPS analysis to strengthen our conclusions. Furthermore, the C 1*s* and F 1*s* spectra have been added to the Supplementary Information (**Supplementary Figs. 6/7**, corresponding to **Figures R12/R13**). An updated version of **Fig. 2** (now **Figure R14**) is also included, and all corresponding textual revisions have been made.

"(see details in **Supplementary Note 2**). The XPS spectra of calibrated C 1*s* and F 1*s* references for $FAPbI_3$-based perovskite with ligands are provided in **Supplementary Figs. 6** and **7**. As shown in **Figs. 2a–c**, monotonic binding energy shifts were observed in Pb $4f_{7/2}$ (138.49 → 138.84 eV), I $3d_{3/2}$ (631.09 → 630.63 eV), and N 1*s* (400.81 → 400.62 eV) of the perovskite/ligand systems with increasing N-heteroaromatic content." (**Page 7**, in the revised manuscript)

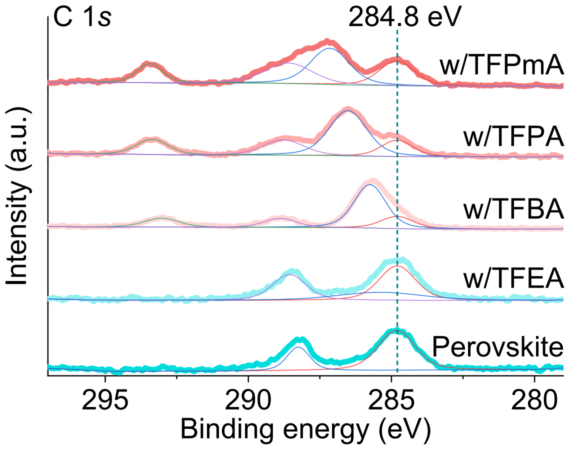

Figure R12. The XPS spectrum of C 1*s* from perovskite films treated with TFEA, TFBA, TFPA, or TFPmA. (**Supplementary Fig. 6**)

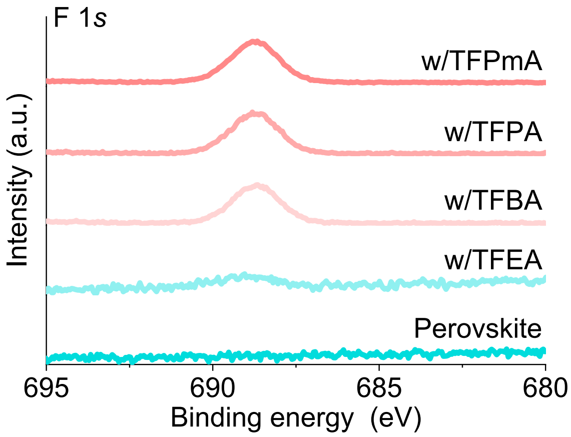

Figure R13. The XPS spectrum of F 1*s* from perovskite films treated with TFEA, TFBA, TFPA, or TFPmA. (**Supplementary Fig. 7**)

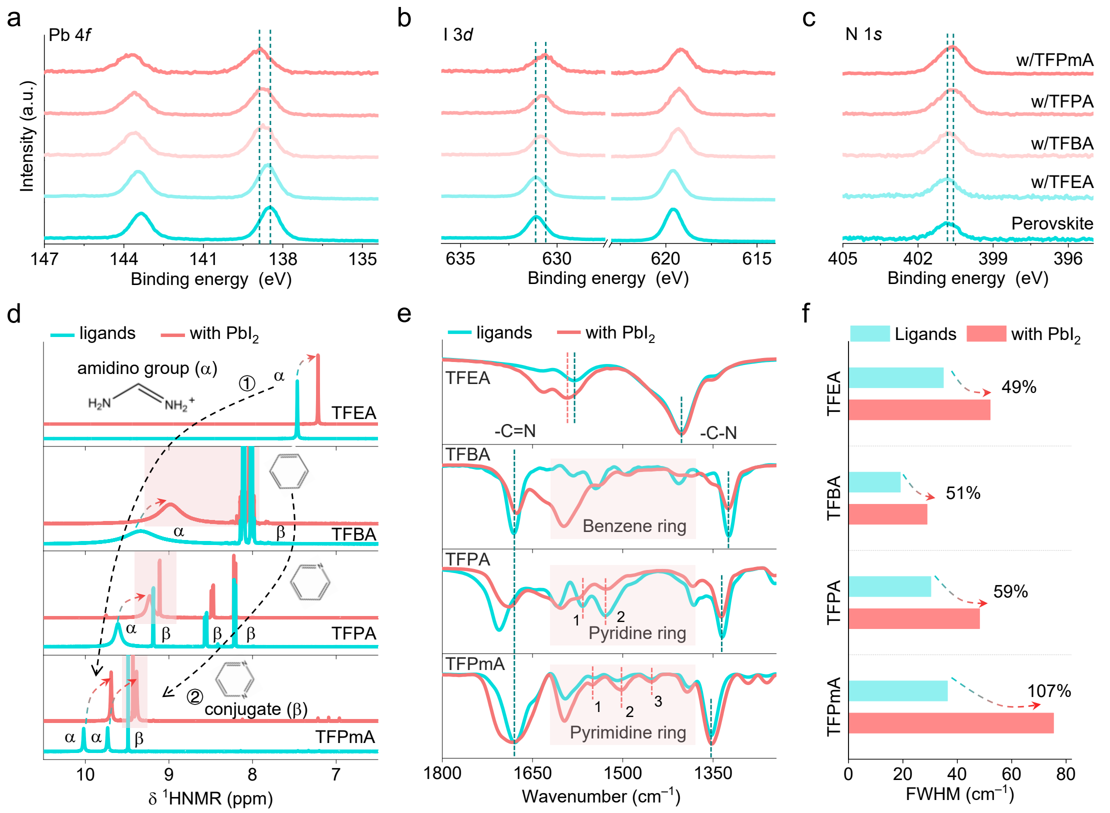

Figure R14. The interaction of ligands with the perovskite framework. XPS spectra of (a) Pb 4*f*, (b) I 3*d* and (c) N 1*s* from perovskite films treated with TFEA, TFBA, TFPA, or TFPmA. (d) 1H-NMR spectra of different ligands interacting with PbI_2. (e) FTIR spectra of different ligands interacting with PbI_2. (f) Full width at half-maximum (FWHM) of the -C=N vibrational peak extracted from the FTIR spectrum. (**Updated Fig. 2**, in the revised manuscript)

(6) To ensure reproducibility and clarity regarding the XPS measurements, we have expanded **Supplementary Note 2** to include a comprehensive description of the sample preparation, transfer protocols, and detailed instrumental measurement conditions.

"XPS measurements and sample preparation: Thin films for XPS characterization were prepared using the same deposition procedures as for functional devices. Following surface ligand modification, all samples were handled entirely within a nitrogen-filled glovebox and transferred to the XPS analysis chamber via an airtight transfer vessel under continuous N_2 atmosphere. This protocol minimized exposure to ambient air, light, and heat, thereby ensuring surface integrity prior to measurement. Spectra were acquired using a monochromatic Al Kα X-ray source (1486.68 eV) with the analysis chamber base pressure maintained between 1.0×10^{-9} and 5.0×10^{-8} mbar. High-resolution scans were recorded at a pass energy of 20 eV." (In the updated **Supplementary Note 2**)

^{1}H NMR Assignments and Interpretation: The reported δHα and δHβ shifts are consistent with progressive multicenter coordination, but solvent effects, dynamic

exchange, and concentration-dependent aggregation are not considered. The authors should acknowledge these limitations or indicate whether variable concentration/temperature studies were attempted.

***Response*:** Thank you very much for raising important points regarding the interpretation of the ^{1}H NMR data. We have conducted additional experiments to address potential confounding factors.

(1) To evaluate solvent effects, we compared spectra in deuterated *d₆*-DMSO and DMF-*d₇*. In both solvents, the addition of PbI_2 induced consistent upfield shifts for δHα and δHβ (**Figures R15–R18**), confirming that the observed shifts originate from Pb–ligand coordination rather than solvent-specific interactions.

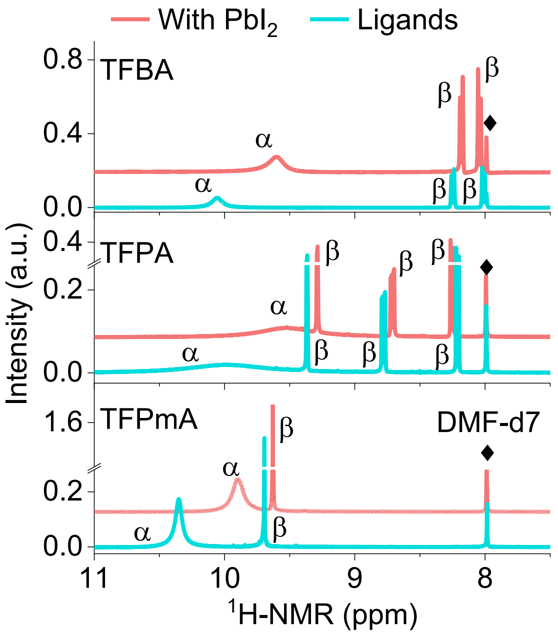

Figure R15. The ^{1}H NMR spectrum of the ligands and PbI_2 dissolved in DMF-d₇. (Note: TFEA data is absent here due to its extremely low solubility in DMF, making signal detection difficult.)

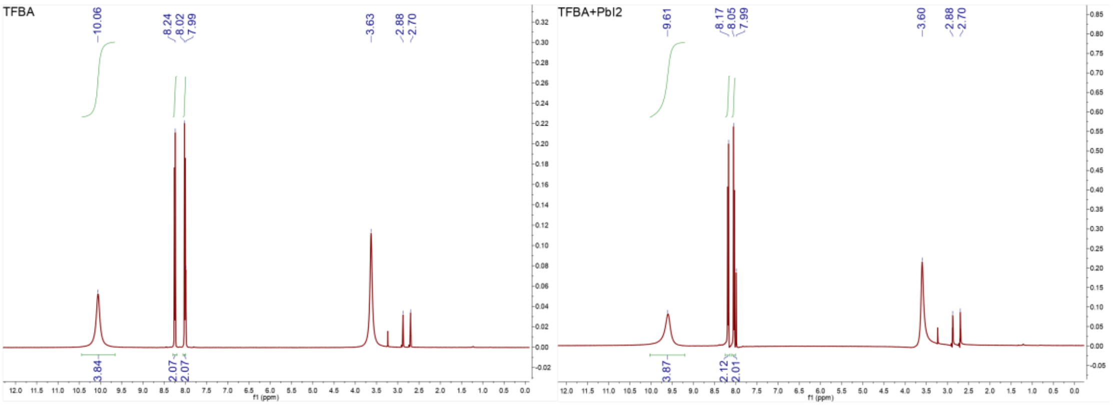

Figure R16. The ^{1}H NMR spectrum of the TFBA and PbI_2 dissolved in DMF-d₇.

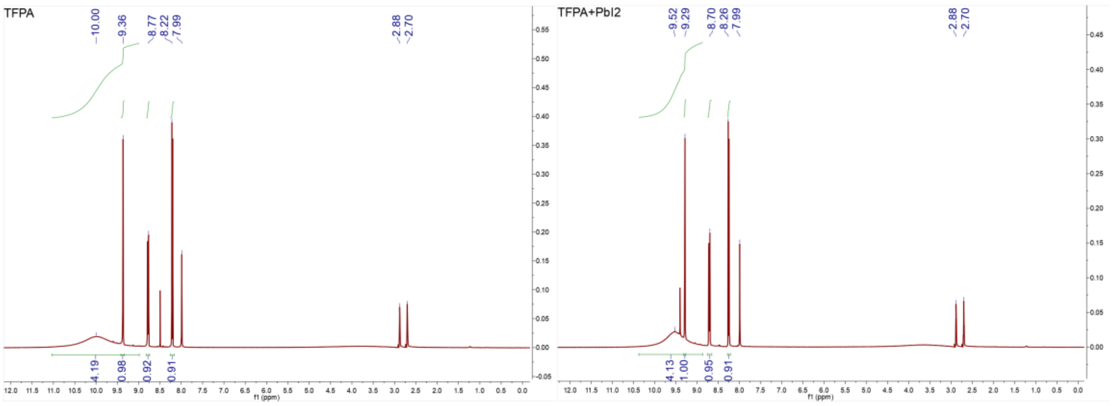

Figure R17. The ^{1}H NMR spectrum of the TFPA and PbI_2 dissolved in DMF-d_7.

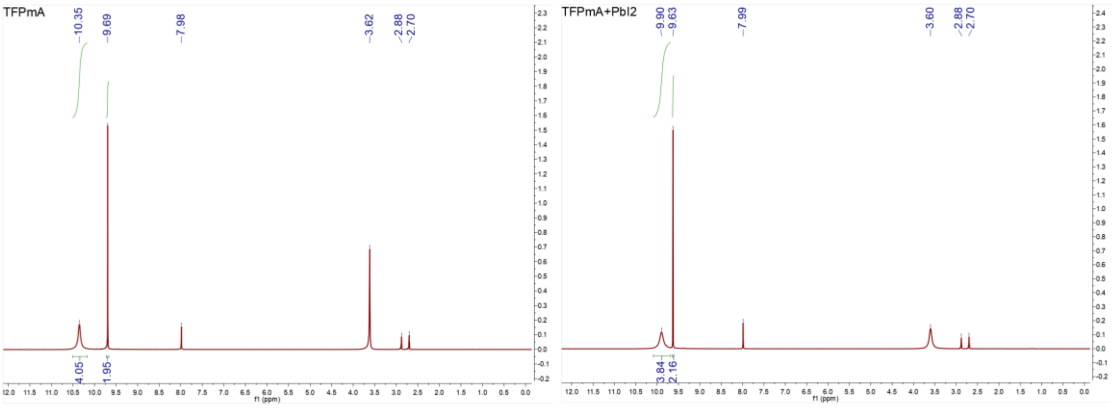

Figure R18. The ^{1}H NMR spectrum of the TFPmA and PbI_2 dissolved in DMF-d_7.

(2) To assess concentration-dependent effects, we measured solutions at 0.02 M, 0.05 M, and 0.1 M in d_6-DMSO. Across all concentrations, PbI_2 addition caused pronounced upfield shifts of δHα and δHβ, with an emerging overlap region between the signals (**Figures R19–R22**). This consistent trend, unaffected by concentration variation, rules out aggregation as the primary cause and reaffirms a strong, specific ligand–Pb^{2+} interaction.

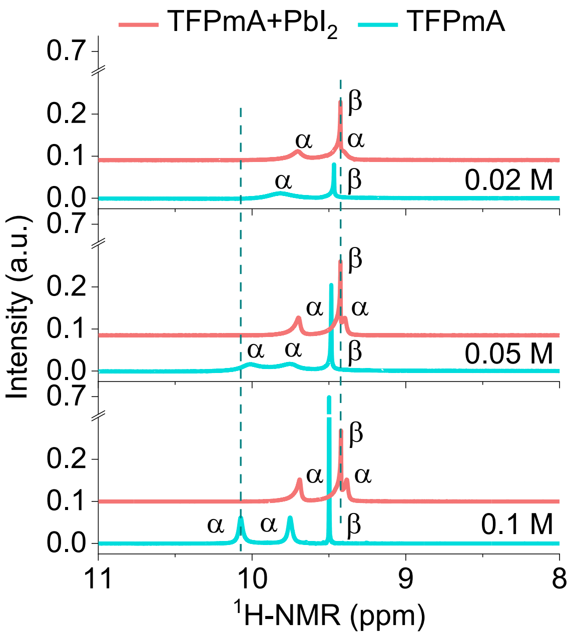

Figure R19. The ^{1}H NMR spectra of different concentrations for TFPmA and TFPmA with PbI_2 (TFPmA : PbI_2=1:1, molar ratio, d6-DMSO).

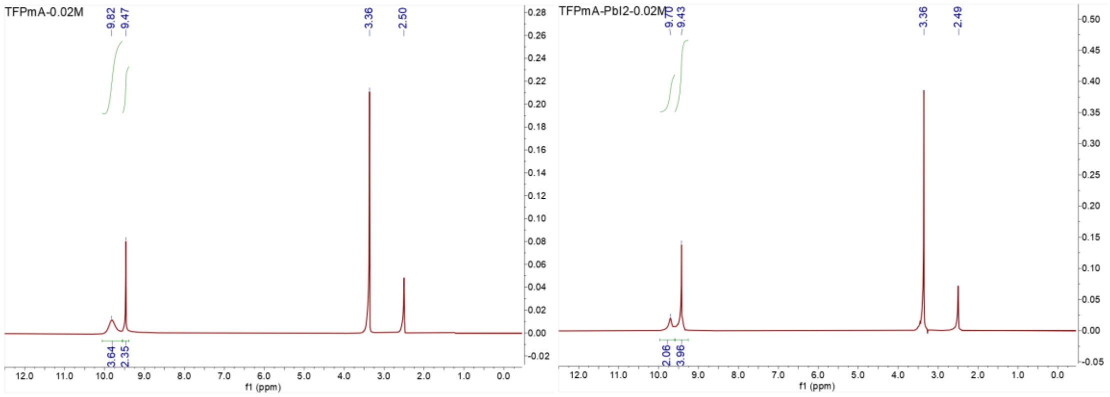

Figure R20. The ^{1}H NMR spectra of TFPmA interacting with PbI_2, solution concentration is 0.02M (TFPmA : PbI_2=1:1, molar ratio, d6-DMSO).

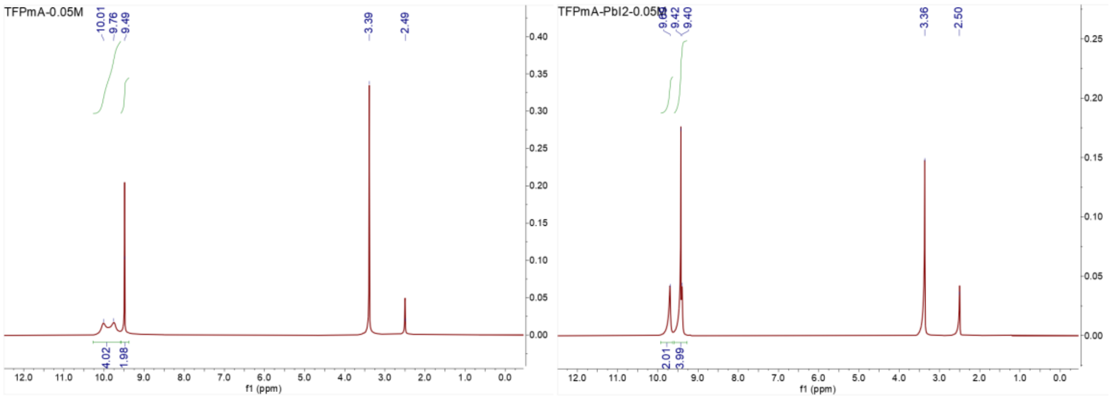

Figure R21. The ^{1}H NMR spectra of TFPmA interacting with PbI_2, solution concentration is 0.05M (TFPmA : PbI_2=1:1, molar ratio, d6-DMSO).

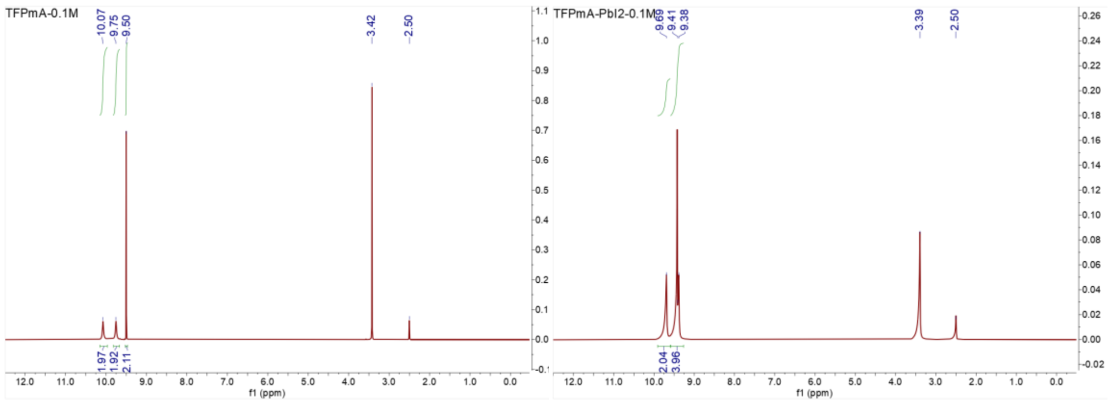

Figure R22. The ^{1}H NMR spectra of TFPmA interacting with PbI_2, solution concentration is 0.1M (TFPmA : PbI_2=1:1, molar ratio, d_6-DMSO).

(3) Collectively, these control experiments demonstrate that the upfield shifts of δHα and δHβ are intrinsic to the ligand–Pb interaction and are not artifacts of solvent, dynamic exchange, or concentration effects. We have incorporated a discussion of these results in the revised **Supplementary Note 2** for clarity.

"NMR measurements and sample preparation: To investigate the coordination interaction between the passivating ligands (TFEA, TFBA, TFPA, and TFPmA) and PbI_2, liquid-state ^{1}H NMR experiments were carried out in deuterated DMSO (d_6-DMSO). Each ligand and its equimolar mixture with PbI_2 (1:1 molar ratio) were dissolved in d_6-DMSO and analyzed by ^{1}H NMR. Upon addition of PbI_2, distinct upfield shifts were observed for the amidino and aromatic proton signals, indicating strong ligand–Pb^{2+} coordination. These ligand-dependent spectral changes reveal a clear hierarchy of coordination strength that correlates with the trends in defect passivation and device performance." (In the updated **Supplementary Note 2**)

FTIR Evidence of Coordination: The assignments of vibrational shifts (C=N, C–N, ring modes) to Pb–N and Pb–I–π interactions are reasonable but could also result from hydrogen bonding or heterogeneous coordination. Peak broadening should be analyzed more quantitatively (e.g., FWHM fitting), and alternative interpretations explicitly addressed.

***Response*:** Thank you very much for your valuable comments on the spectroscopic analyses. We have systematically addressed each point raised, as detailed below.

(1) Distinguishing Bonding Contributions

To evaluate the relative influence of hydrogen bonding versus heterogeneous coordination on the observed vibrational shifts (C=N, C–N, ring modes), we examined the N–H vibration upon ligand interaction with PbI_2. A clear shift confirms the presence of Pb-N/hydrogen-bonding interactions between amidino and Pb-I framework (**Figure R23**). Notably, introducing N-heterocycles (TFBA, TFPA, TFPmA) significantly amplified the shift, indicating the multicenter interactions included the added Pb–I–π interactions and stronger Pb–N contribution become the primary factor altering the

vibrational signals.

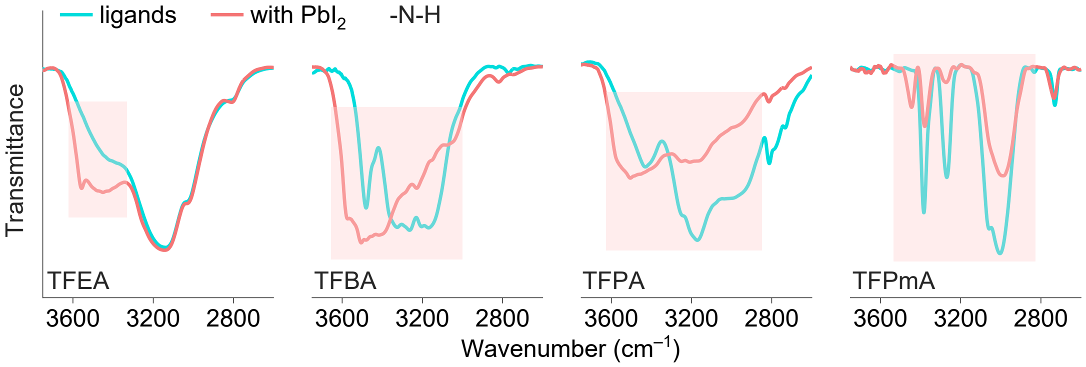

Figure R23. FTIR spectra (3750~2600 cm^{-1}) of different ligands interacting with PbI_2. (**Supplementary Fig. 12**)

(2) Quantitative Analysis of Peak Broadening

We performed a precise spectral deconvolution of the –C=N vibration (**Figure R24**) and compared the full-width-at-half-maximum (FWHM) values (**Figure R25**). Interaction with PbI_2 induced significant peak broadening for all four ligands: FWHM increased by 49% (TFEA), 51% (TFBA), 59% (TFPA), and 107% (TFPmA). The 49% increase for TFEA reflects the contribution of Pb-N/hydrogen bonding between amidino and Pb-I framework. The substantially larger increases (51–107%) for the N-heterocyclic ligands highlight the multicenter interactions included the added Pb–I–π interactions and stronger Pb–N coordination.

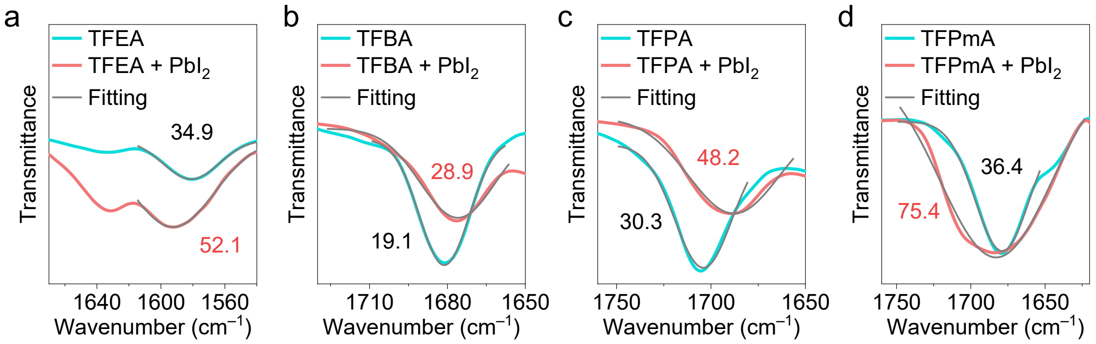

Figure R24. Fitting of -C=N vibrational peak extracted from the FTIR spectrum and corresponding full width at half-maximum (FWHM). (**Supplementary Fig. 13**)

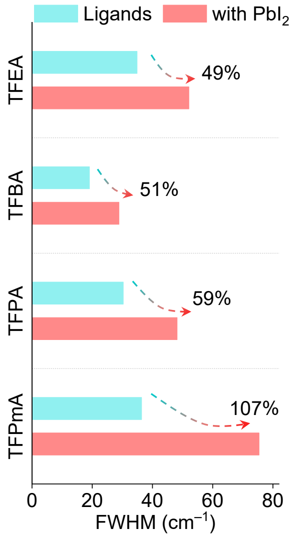

Figure R25. Full width at half-maximum (FWHM) of the -C=N vibrational peak extracted from the FTIR spectrum. **(Updated Fig. 2f)**

(3) Per your suggestion, we have integrated **Figure R25** into the main text as **Fig. 2f**, and included **Figures R23** and **R24** in the Supplementary Information as **Supplementary Figs. 12** and **13**. Corresponding descriptions have been added to the revised manuscript and **Supplementary Note 2**.

"FTIR spectroscopy of ligands/PbI_2 complexes (**Fig. 2e** and **Supplementary Figs. 12 and 13**) …… Introducing N-heteroaromatic motifs reversed this trend: TFBA and TFPA exhibited C=N downshifts (Δν = –4/–17 cm^{-1}) with full width at half-maximum broadening (FWHM: 19→29/30→48 cm^{-1}) (**Fig. 2f**). TFPmA further amplified this effect, displaying strong broadening by 107% (FWHM: 36→75 cm^{-1}) of C=N stretch and a –3 cm^{-1} redshift in the C–N vibration (1,354→1,351 cm^{-1})." (**Page 7-8**, in the revised manuscript)

"FTIR measurements and sample preparation: FTIR measurements were performed on KBr pellets (sample:KBr = 1:100) pressed under vacuum, with spectra acquired from 4000–400 cm^{-1} at 0.4 cm^{-1} resolution. Interaction with PbI_2 broadened the –C=N stretching vibration, with FWHM increases of 49% (TFEA), 51% (TFBA), 59% (TFPA), and 107% (TFPmA). This trend reflects stronger ligands-perovskite interactions at the presence of multicenter interactions (TFBA, TFPA, TFPmA)" (In the updated **Supplementary Note 2**)

Cross-technique consistency: While the manuscript claims excellent agreement between XPS, NMR, FTIR, and DFT, some trends are not fully reconciled. For example, NMR suggests a transition from monodentate to multicenter coordination, while FTIR emphasizes dominant Pb–N interactions. These differences should be explicitly discussed.

Response: Thanks for insightful comments. The FTIR results indeed provided direct evidence for the presence of both Pb–N and Pb-I-π multicenter interactions. First, the

observed red-shifts (Δν = –4/–17 cm^{-1}) and significant FWHM broadening of the amidino C=N stretching vibration demonstrate the **formation of Pb–N bonds** between the amidino group and the Pb–I framework (**Fig. 2f**). Second, the emergence of split vibrational modes for the pyridine and pyrimidine C=N stretches indicated the **direct participation of the N-heteroaromatic motifs** in Pb–I–π interactions (**Fig. 2e**). Together, these two spectroscopic features confirm the presence of multicenter coordination and illustrate the evolution from a primarily monodentate bonding mode (in TFEA) to a multicenter coordination mode (in TFBA, TFPA, and TFPmA).

To present a clearer argument, we have implemented the corresponding revisions throughout the manuscript. The new FTIR analysis has been incorporated into both the main text and the Supplementary Information. The manuscript has been updated as detailed below, ensuring a coherent interpretation of all observed trends.

"Concurrently, N-heteroaromatics participation emerged via split vibrational modes (1,590–1,400 cm^{-1}): pyridine N-Pb interaction softened ring breathing (1,571/1,526 cm^{-1}), while pyrimidine distortion split the symmetric stretch into a 1,550/1,502/1,452 cm^{-1} triple (**Fig. 2e,** shaded area lines 1-3), which demonstrate **direct engagement** of N-heteroaromatic motifs in Pb–I–π **interaction**. The spectroscopic observations align with DFT-predicted multicenter interactions between ligands and Pb-I framework, …" (**Page 8**, in the revised manuscript)

"Finally, a consistent coordination hierarchy among the ligands is established through multiple complementary techniques. Solution-phase ^{1}H NMR (chemical shifts of δHα and δHβ) and solid-state characterizations—including core-level shifts of Pb, I, and N in XPS and vibrational changes of key functional groups in FTIR—provide convergent evidence. Together, these results substantiate strong and multicenter ligand and Pb-I coordination at the perovskite interface." (In the updated **Supplementary Note 2**)

3. Structural and Morphological Analyses

GIWAXS, XRD, and SEM results are used to exclude low-dimensional phases or lattice distortions. However, these techniques may lack sensitivity to subtle local rearrangements or minority phases. The conclusions should be tempered accordingly. SEM images support improved film uniformity but remain qualitative; their direct correlation with electronic improvements should be made more cautiously.

***Response*:** We really appreciate your insightful comment on the sensitivity of morphological and structural characterization. To answer your question, we have conducted a complementary investigation to address the concern about potential low-dimensional phases.

(1) Acknowledging the inherently limited sensitivity of conventional GIWAXS and XRD for detecting minor low-dimensional phases, we employed ultrafast transient absorption spectroscopy (TAS) as a more sensitive probe. TAS results (**Figure R26**) show that after ligand modification, only the characteristic ground-state bleach signal of the 3D bulk phase was observed at ~790 nm, with no detectable signatures below

700 nm that would indicate low-dimensional phases. This confirms the absence of such phases induced by our ligand treatment.

Based on this finding, the TAS data (**Figure R26**) have been added to the Supplementary Information as **Supplementary Fig. 16**. Corresponding discussion has been incorporated into the main text to strengthen the accuracy and reliability of our structural conclusions.

"and ultrafast transient absorption spectroscopy (TAS)" and "*with no detectable low-dimensional phase formation or lattice distortion (**Supplementary Figs. 14-16**).*" (**Page 9**, in the revised manuscript)

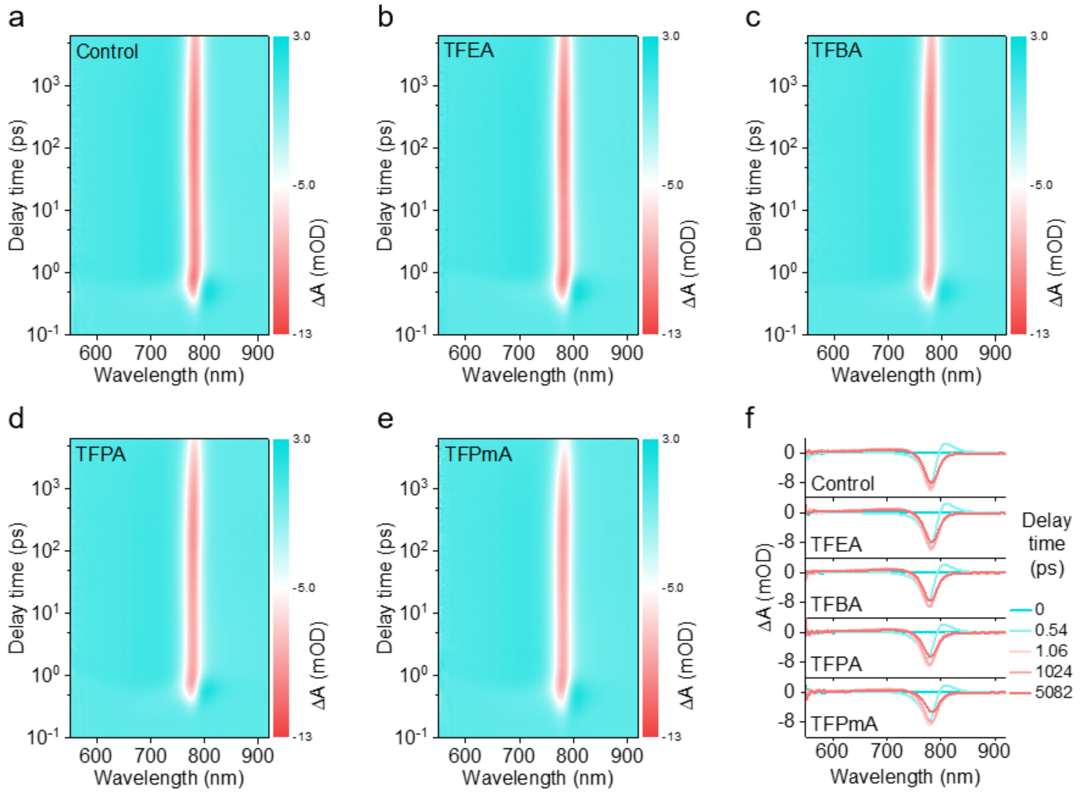

Figure R26. TAS spectrum of the film with ligands modifications. (**Supplementary Fig. 16**)

(2) Thank you very much for the rigorous and logical considerations regarding the correlation between SEM morphology and electronic improvements. To enhance the rigor of the manuscript, we have revised the wording in the main text accordingly, as described below:

"The improved surface smoothness and potential homogenization, driven by the N-heteroaromatic motifs, enhance the contact-related interfacial quality and is beneficial for interfacial charge extraction." (**Page 9**, in the revised manuscript)

4. Energetics and Photophysics

UPS results demonstrate favorable energy-level alignment, but attributing ~0.1 eV shifts to Pb–I–π interactions is speculative without direct evidence of orbital

hybridization.

Response**:** Thanks for the rigorous and insightful considerations, which have provided us with an excellent opportunity to further validate our conclusions through theoretical calculations. Below, we present the results of these calculations in detail.

(1) We employed molecular orbital theory to calculate the HOMO and LUMO energy levels of the four ligands (**Figure R27**). The LUMO and HOMO energies of TFEA are −0.63 eV and −7.10 eV, respectively, whereas those of TFBA are −1.52 eV and −6.64 eV. Upon the gradual introduction of one N heteroatom (TFPA) and two N heteroatoms (TFPmA) into the aromatic ring, the LUMO levels further decrease to −1.93 eV and −2.50 eV, respectively (**Table R1**). These results clearly indicate that incorporating N heteroatoms into the conjugated aromatic framework effectively tunes the intrinsic electronic structure of the ligands, particularly by progressively lowering their LUMO levels. When such ligands are introduced at the perovskite surface, the modified molecular energy levels can induce interfacial electrostatic regulation, leading to a downward shift of the perovskite conduction band minimum (CBM). This trend is fully consistent with the energy-level alignment derived independently from UPS measurements.

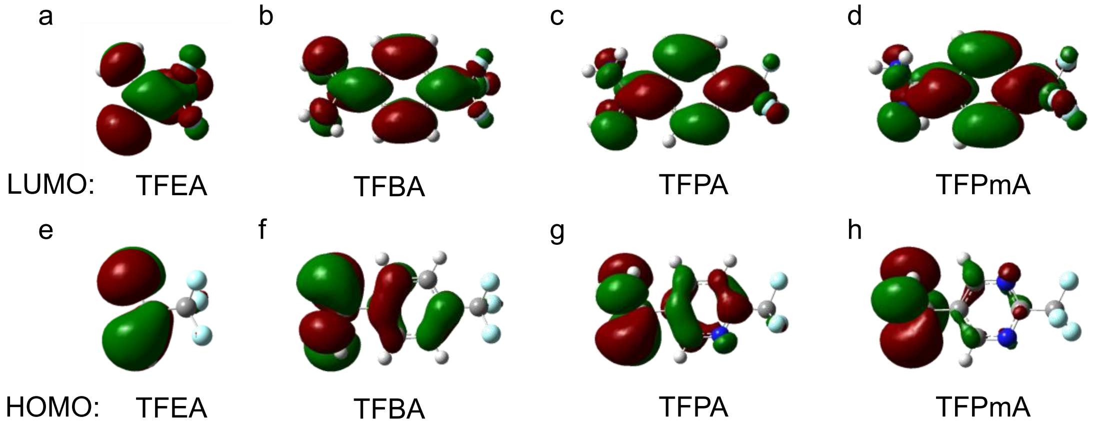

Figure R27. Molecular orbital calculations. **a–d**, the LUMO and HOMO energy levels for TFEA, TFBA, TFPA, and TFPmA ligands, respectively. (**Supplementary Figs. 27**)

Table R1. The LUMO and HOMO energy levels for TFEA, TFBA, TFPA, and TFPmA, respectively.

	TFEA	TFBA	TFPA	TFPmA
LUMO (eV)	-0.63	-1.52	-1.93	-2.50
HOMO (eV)	-7.10	-6.64	-6.91	-7.02

(2) To further examine whether the observed energy-level shifts originate from orbital hybridization or electrostatic effects, we calculated the projected density of states (pDOS) for the perovskite/C_{60}, perovskite/TFBA/C_{60}, and perovskite/TFPmA/C_{60} interfaces (**Figure R28**). For the pristine perovskite/C_{60} interface, the CBM is predominantly composed of Pb-derived states, in agreement with the established electronic structure of lead halide perovskites. Upon introducing the TFBA ligand, the CBM shifts downward from 1.32 to 1.26 eV, while its orbital character remains

dominated by Pb states. A further downward shift of the Pb-derived CBM to 1.23 eV is observed upon incorporation of the TFPmA ligand, corresponding to a band offset of approximately 0.09 eV relative to the perovskite/C_{60} interface. Importantly, although N-derived states appear at higher energies in the pDOS, they do not form continuous band-edge states at the CBM. This indicates that the intrinsic CBM remains governed by Pb orbitals and that no direct orbital hybridization between ligand π states and perovskite band-edge states occurs. Instead, the progressive CBM shift is attributed to interfacial charge redistribution including electrostatic effects, ligand-induced dipole formation, and associated band bending, rather than to the formation of hybridized electronic states.

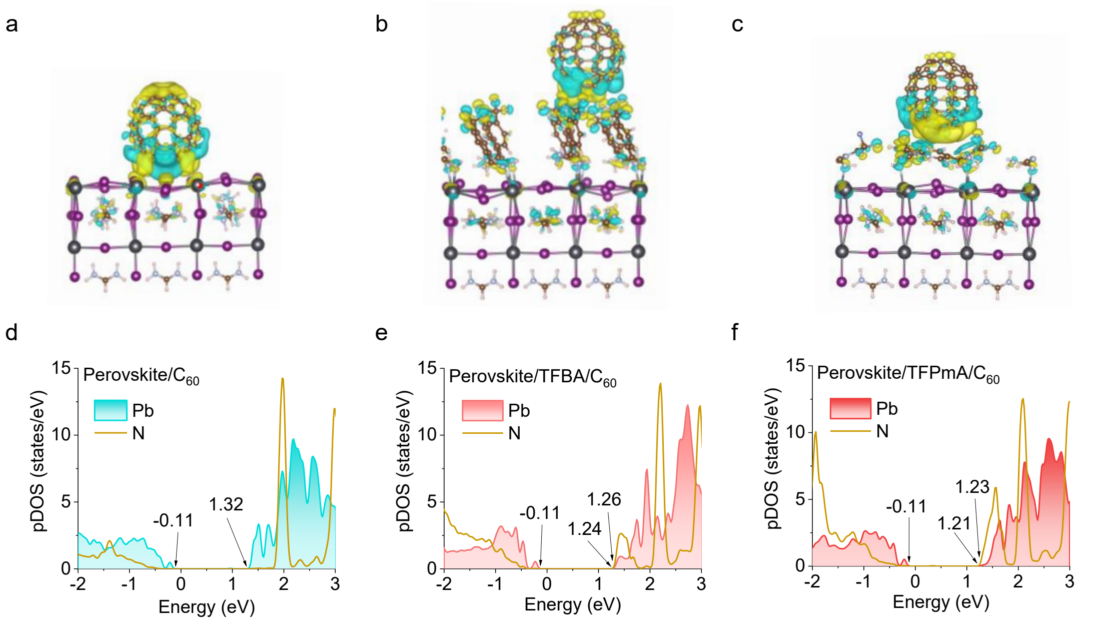

Figure R28. Differential charge-density maps and computed pDOS plots of the Pb and N in perovskite/C_{60}, perovskite/TFBA/C_{60} and perovskite/TFPmA/C_{60}, respectively. (**Supplementary Figs. 28**)

(3) Based on the combined molecular orbital calculations and pDOS analysis, we conclude that the ~0.1 eV energy-level shifts observed in UPS originate from ligand-induced electrostatic modulation of the interfacial energy landscape, rather than from direct Pb–I–π orbital hybridization. Accordingly, we have revised the manuscript to clarify this mechanism and to avoid overinterpretation of orbital coupling effects. **Figures R27** and **R28**, along with **Table R1**, have been incorporated into the Supplementary Information as **Supplementary Figs. 27** and **28**, and **Supplementary Table 3**, respectively. Corresponding clarifications and additional discussion have been added to the UPS results section, as detailed below:

"Molecular orbital calculations and differential charge-density maps indicated that ligand–perovskite multicenter interactions induced interfacial charge redistribution, which in turn promoted interfacial energy-level realignment (**Supplementary Figs. 27** and **28,** and **Supplementary Table 3).** The sub-0.1 eV energy offset at the perovskite/TFPmA/C_{60} interface drives the energy-barrier to the theoretical minimum,

enabling quasi-ideal charge extraction across the heterojunction." (**Page 10**, in the revised manuscript)

The reported improvements in QFLS are corroborated by in situ PL, TRPL mapping (Supplementary Figs. 18–24), and Mott–Schottky analysis (Supplementary Fig. 25), which collectively confirm reduced nonradiative recombination. Nevertheless, the mechanistic interpretation of these improvements as arising from "bandgap homogenization" could be more cautiously framed, as the presented photophysical data capture carrier dynamics and defect passivation but do not unambiguously evidence a homogenization of the electronic bandgap.

Response**:** Thank you for providing us with the opportunity to reflect more deeply on this point, and we agree with your assessment. The reduction in nonradiative recombination should not be attributed to bandgap homogenization, but rather to interfacial defect passivation. To ensure a more rigorous and accurate presentation, we have revised the relevant statements in the manuscript accordingly.

"This 61 meV enhancement, enabled by stereoelectronic manipulation, stems from above observed synergistic interfacial mechanisms: topographic defect healing through multicenter interactions, delocalized electronic screening mediated by Pb–I–π interactions, a reduction in the interfacial energetic barrier,[13,24] and a rise in molecular dipole moment that reshapes the local electrostatic landscape.[38,39]" (**Page 10**, in the revised manuscript)

In situ PL measurements indicate a two-stage ligand anchoring and defect passivation process, and TRPL lifetimes demonstrate the dual role of ligand adsorption in passivation and charge extraction; however, assigning specific mechanistic steps solely from PL/TRPL intensity and decay trends may be oversimplified, as these signals reflect a convolution of bulk and interfacial processes. Collectively, the spectroscopic data convincingly demonstrate ligand-mediated interfacial effects, but a cautious interpretation acknowledging these potential complexities would strengthen the manuscript.

Response**:** Thanks for the rigorous evaluation. In response to your comments on the PL analysis, we have conducted a thorough assessment of the PL probing depth and examined whether it could influence our interpretation of defect passivation. The detailed analysis is provided below:

(1) We first analyzed the PL probing depth to determine whether the detected defects originate from the surface and near-surface regions. It is known that the detection depth of PL depends on the excitation wavelength and the material's absorption coefficient (**Figure R29**). For the same material (i.e., an identical absorption coefficient), higher-energy (shorter-wavelength) lasers are typically used to probe surfaces and interfaces owing to their more limited penetration depth. In this study, the excitation wavelengths used for in situ PL and TRPL measurements are 405 nm and 510 nm, respectively. The corresponding probing depths are approximately 100 nm. Therefore, the detected PL

signals **predominantly originate from the top surface region** of the thin film, with a minor contribution from the bulk region, consistent with previous reports (*Nat. Mater.* 2017, 16, 115–120; *J. Phys. Chem. Lett.* 2015, 6, 4171–4177; *J. Am. Chem. Soc.* 2015, 137, 10456–10459).

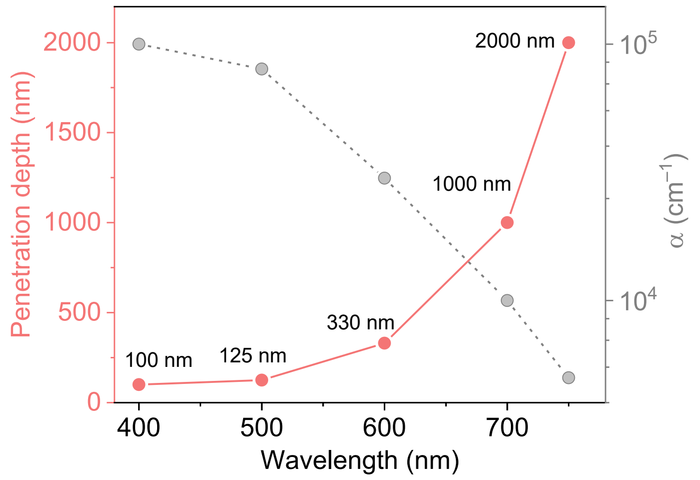

Figure R29. Relationship between laser wavelength and the probing depth and absorption coefficient in perovskite materials.

(2) Based on the probing depth analysis, we further evaluated whether variations in PL can be used to assess changes in surface defect states. Since the bulk properties of the perovskite films are **almost identical** for *in situ* diagnosis, **the contribution from the bulk can be largely excluded**, allowing the observed PL variations to be predominantly associated with the top surface region. Therefore, the PL enhancement induced by surface modification is primarily attributed to surface defect passivation.

(3) Based on the reviewer's comments and our further evaluation, we now include the excitation wavelengths and measurement conditions in the Experimental section to further enhance the rigor of the manuscript. The added information is provided below:

"*In-situ* PL spectra were performed with a short-wavelength laser diode (405 nm) which aims to detect the surface region of films to minimize interference from the bulk material," (1.5 Film characterization, in the Supplementary Information)

"Moreover, the initial state of all perovskite films measured was identical." (1.5 Film characterization, in the Supplementary Information)

"were investigated using *in situ* photoluminescence (PL) spectroscopy with a 405 nm laser (see details in **Supplementary Information**)." (**Page 12**, in the revised manuscript)

"A subsequent, slower stage (~3–20 s) driven by solvent evaporation then ensued. The PL intensity further increased (up to 28% for TFPmA; **Fig. 4c**), consistent with ongoing defect passivation that may involve Pb–N coordination and Pb–I–π interactions.[12,40]" (**Page 12**, in the revised manuscript)

"in the unmodified control (Fig. 4d)—indicating enhanced charge extraction efficiency at the perovskite/ligand/CTL interface with a planar adsorption topology." (**Page 12**, in the revised manuscript)

5. Device-Level Electrical Analyses

Mott–Schottky, EIS, and light-intensity V_{OC} analyses support reduced recombination and improved interface passivation. However, the unusually large changes (78% reduction in Rs, ~9× increase in Rrec) raise the possibility of parasitic or contact-related contributions. The correlation between these results and the proposed "σ/π dual bonding" should therefore be interpreted more carefully.

***Response*:** Thank you very much for the insightful comment. We agree that contact-related effects can influence Rs and Rres, as documented in prior studies (*Nat. Commun.* 2023, 14, 3738; *Energy Environ. Sci.* 2024, 17, 1153; *Energy Environ. Sci.* 2024,17, 1570-1579). We acknowledge that attributing these changes solely to "σ/π dual bonding" oversimplifies the interpretation. To ensure rigor, we have refined the relevant discussion in the manuscript accordingly.

"in line with the improvement in charge-separation efficiency suggested by the QFLS data." (**Page 13**, in the revised manuscript).

"demonstrating suppression of interfacial recombination which stems from N-heteroaromatic motif contribution including contact-related morphological optimization and multicenter coordination-induced electronic microenvironment enhancement.[40,41]" (**Page 14**, in the revised manuscript).

6. Stability and Module Fabrication

The outdoor stability test (130 days, 0–1.31 sun, –11.5 °C to 65.7 °C) is a remarkable result, with only 7.5% loss in efficiency. However, as the test was not conducted under standardized ISOS or IEC protocols, comparisons with industrial benchmarks should be made cautiously.

***Response*:** Thank you very much for acknowledging the significance of the long-term outdoor stability data. We will provide a detailed response below:

(1) We fully agree that standardized ISOS and IEC protocols provide reliable framework for benchmarking stability. The outdoor test was therefore designed as a **complementary real-world practical evaluation** to the ISOS-type accelerated aging experiments already included in our study. Specifically, bare devices without encapsulation retained **90%** of their initial PCE after 1,056 h at 85 °C (**ISOS-D-2**) and 2,190 h under MPP tracking (**ISOS-L-1**) (**Supplementary Figs. 47** and **48**), confirming excellent operational durability under standardized conditions. The outdoor experiment further validated the robustness of the multicenter interactions under realistic environmental fluctuations for future real-world practical operation.

(2) As an additional note, up to the time of manuscript revision and resubmission, the outdoor real-time field testing of our perovskite module has been extended to **258** days

(**Figure R30**). During this period, the device experienced realistic environmental conditions, including temperature cycling between −11.5 °C and 68.9 °C, irradiance fluctuations from 0 to 1.39 sun, and plane-of-array global irradiance (PGD) variations from 0 to 34.7 mW cm^{-2}. After 258 days of continuous outdoor operation, the efficiency decreased from an initial value of 23.10% to 19.82%. To our knowledge, **this represents the first >23%-efficiency perovskite module to demonstrate such prolonged operational stability.**

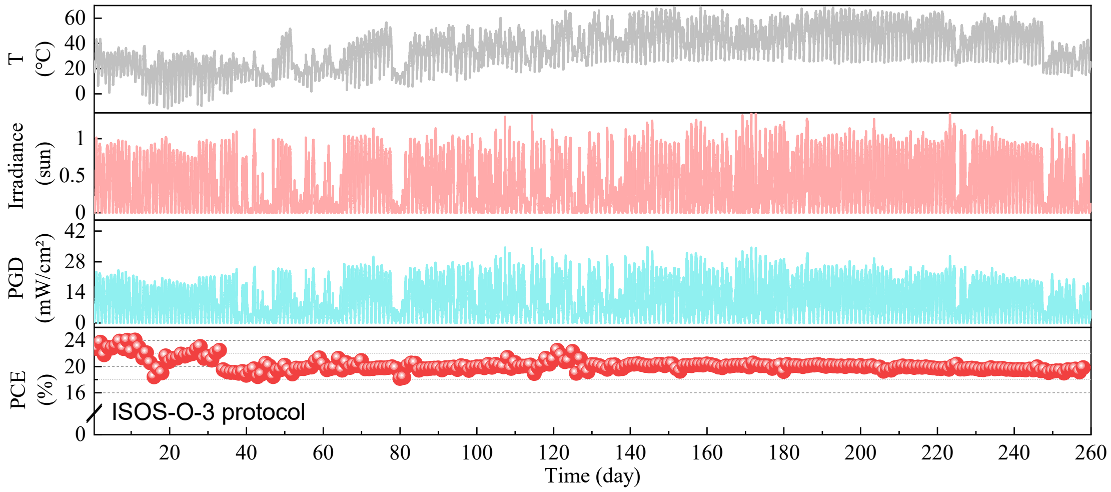

Figure R30. The real-time field testing of TFPmA-treated module under actual outdoor operating conditions for 258 days. (**Updated Fig. 5g**, in the revised manuscript)

(3) In the revised manuscript, we have updated the outdoor operational stability data (**Fig. 5g**) and provided an appropriate clarification distinguishing ISOS or IEC protocols from real-world outdoor stability testing. We also thank the reviewer for helping us further improve the rigor of the manuscript.

"(**Fig. 5g, Supplementary Figs. 51** and **52**). Subjected to 258 days of continuous operation with temperature cycling (–11.5 to 68.9 °C) and irradiance fluctuations (0–1.39 sun; plane of array global irradiance (PGD) of 0–34.7 mW cm^{-2}), the TFPmA-treated module exhibited only a 14.2% loss (from 23.10% to 19.82% PCEs). In sharp contrast, the control module degraded by 24.6% (from 21.03% to 15.85% PCEs) after merely 27 days of outdoor testing (**Supplementary Fig. 53**)." (**Page 16**, in the revised manuscript).

"The results from ISOS-D-2 testing, ISOS-L-1 testing, and real time field testing collectively demonstrate that stereoelectronic manipulation of ligands not only minimizes energy-loss pathways at the perovskite/CTL interface but also endows devices with remarkable operation robustness at practical operating conditions, which would provide a compelling technical foundation for reliable large-scale perovskite photovoltaics deployment." (**Page 16**, in the revised manuscript)

Details of module fabrication also require clarification:
• The type of laser and parameters for P1, P2, P3 steps are not specified.
• The encapsulation process is insufficiently described for reproducibility; a schematic of encapsulation steps and interconnections would be valuable.
• Precursor solution protocols are inconsistently reported: the first recipe provides exact masses, while wide-bandgap and mini-module formulations use only nominal concentrations. For reproducibility, absolute quantities (mg or mmol) should be included.

***Response*:** We have revised the Methods and Supplementary Information sections to include full details of the laser scribing, encapsulation, and precursor formulations, as follows:

(1) Laser scribing parameters (P1–P3):

The process parameters have now been fully specified. The P1 (nanosecond green laser) scribing parameters were: scan speed = 1.0 m s^{-1}, laser power = 1.5 W, and repetition rate = 350 kHz. The P2 (picosecond green laser) scribing parameters were: scan speed = 0.5 m s^{-1}, laser power = 0.2 W, and repetition rate = 200 kHz. The P3 (picosecond green laser) parameters were: scan speed = 0.5 m s^{-1}, laser power = 0.15 W, and repetition rate = 200 kHz.

We have also added the above-mentioned laser types and parameters to the section of "Fabrication of perovskite solar modules" in Supplementary Information.

"the P1 laser (nanosecond green laser, scan speed = 1.0 m s^{-1}, laser power = 1.5 W, and repetition rate = 350 kHz.)"

"The P2 laser (picosecond green laser, scan speed = 0.5 m s^{-1}, laser power = 0.2 W, and repetition rate = 200 kHz.)"

"P3 laser (picosecond green laser, scan speed = 0.5 m s^{-1}, laser power = 0.15 W, and repetition rate = 200 kHz.)"

(1.4 Fabrication of perovskite solar modules, in the Supplementary Information)

(2) Encapsulation process:

A detailed description of the encapsulation procedure has been added, including materials (UV-curable epoxy, barrier glass, edge-sealing adhesive). A schematic diagram illustrating the encapsulation sequence and cell interconnections is now provided in **Figure R31**.

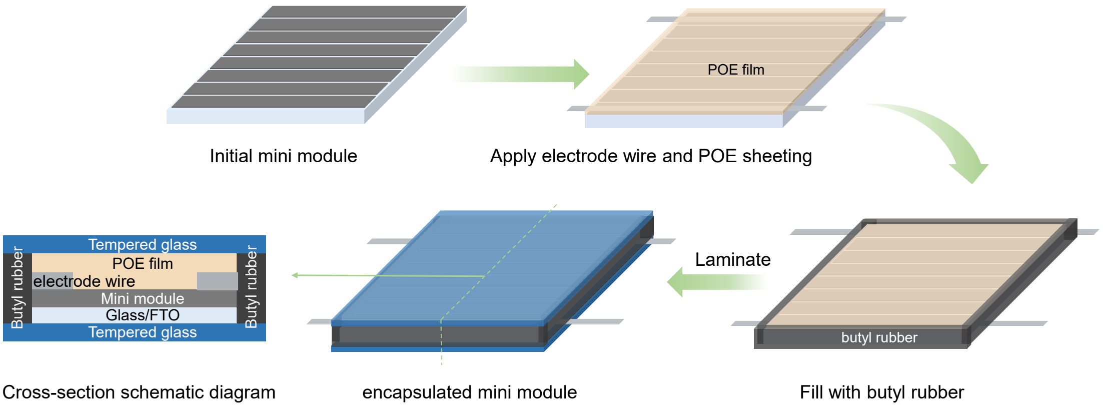

Figure R31. The schematic diagram illustrating the encapsulation sequence and cell interconnections. (**Supplementary Fig. 51**)

(3) Precursor solution formulations:

All precursor recipes have been standardized to report absolute quantities (mg) alongside molar concentrations for full reproducibility. These details have been updated in the Supplementary Methods, covering both the wide-bandgap and mini-module compositions.

"Normal-bandgap $FAPbI_3$-based perovskite (1.56 eV) solution (1.5 M, $FA_{0.85}MA_{0.1}Cs_{0.05}Pb(I_{0.98}Br_{0.02})_3$) was prepared by mixing 807 mg $FAPbI_3$ single-crystal powder, 138 mg PbI_2, 23.9mg MAI, 19.5 mg CsI, 16.5 mg $PbBr_2$, and 12.3 mg MACl in the 1 ml mixed solvent of DMF and DMSO (volume ratio: 4:1) and stirred for 2 h." (1.2 Perovskite precursor solution, in the Supplementary Information)

"Wide-bandgap perovskite (1.68 eV) solution (1.3 M, $Cs_{0.22}FA_{0.78}Pb(I_{0.85}Br_{0.15})_3$) was prepared by mixing 641.8 mg $FAPbI_3$ single-crystal powder, 20.3 mg PbI_2, 74.3 mg CsI, and 107.3 mg $PbBr_2$ in mixed solvent of DMF and DMSO (volume ratio: 4:1) and stirred for 5 h, 5 mol% MACl (4.4 mg) and $PbCl_2$ (18.1 mg) with molar ratio were added to increase the crystallinity." (1.2 Perovskite precursor solution, in the Supplementary Information)

"Mini-modules perovskite: 1.2 M $FA_{0.9}Cs_{0.1}PbI_3$ was prepared by mixing 31.2 mg CsI, 55.3 mg PbI_2 and 683.6 mg $FAPbI_3$ single-crystal powder in mixed solvent of DMF and DMSO (volume ratio: 4:1) and stirred for 2 h, 15 mol % MACl (12.2 mg) and 3 mol % $PbCl_2$ (10.0 mg) with molar ratio were added to increase the crystallinity." (1.2 Perovskite precursor solution, in the Supplementary Information)

7. Manuscript Structure

The final section, currently titled "Discussion," functions more as a summary of results.

Unless expanded to provide deeper synthesis, it should be retitled “Conclusions” or “Discussion and Conclusions” in line with Nature editorial standards.

***Response*:** We agree that the current section primarily summarizes key findings. To better align with Nature’s editorial standards, we have **retitled the section as “Conclusions”** in the revised manuscript (**Page 18**, in the revised manuscript).

Referee #2 (Remarks to the Author):

In this work, the authors explored four amino-based derivatives named TFEA (without conjugated core), TFBA (with a benzene ring), TFPA (pyridine), and TFPmA (pyrimidine) in perovskite solar cells. The importance of stereoelectronic manipulation of ligands is evoked where (i) the integration of N-heteroaromatic motifs into ligands, reorient the molecular adsorption from vertical to planar configuration with respect to the perovskite surface. (ii) This planar interfacial configuration enhances the bonding strength and reduces the charge transport distance. (iii) The description of multivariate analysis (Figures 3d, S12, and S13) is valuable. We have the tendency to foresee a monolayer and full coverage of passivating molecules on perovskites. However, in this work a rather poor coverage of passivating molecules (<50% coverage) is experimentally revealed, which still has impressive impact on device performance. As proof-of-concept, the device performances achieved impressive efficiency with the certified PCE of 26.98%. The authors also performed a thorough and comprehensive characterization, which certainly warrants its publication in Nature. However, I still had a few questions reading the manuscript that would like to receive feedback from the authors.

1. Clarification is needed regarding the statement of "certified" in the main text, which tend to link with the NREL chart. Otherwise, stating "third-party" institution would be more appropriate to avoid confusion to readers at the first glance of the work.

***Response*:** Thanks for this careful evaluation.

Based on your suggestion, and to avoid any potential misunderstanding with the NREL efficiency chart, we have revised the relevant sentence in the main text to read as follows (following further encapsulation optimization, the **certified PCE has now increased to 27.41%** (from 26.98%) (**Figures R32–R34**).)

"We sent the best-performing device to an accredited PV laboratory (the PV Metrology Lab of National Institute of Metrology, China) for third-party certification and obtained an efficiency of 27.41% a with stabilized power output efficiency of 26.85% (**Fig. 5c, Supplementary Figs. 39-42**)." (**Page 15**, in the revised manuscript)

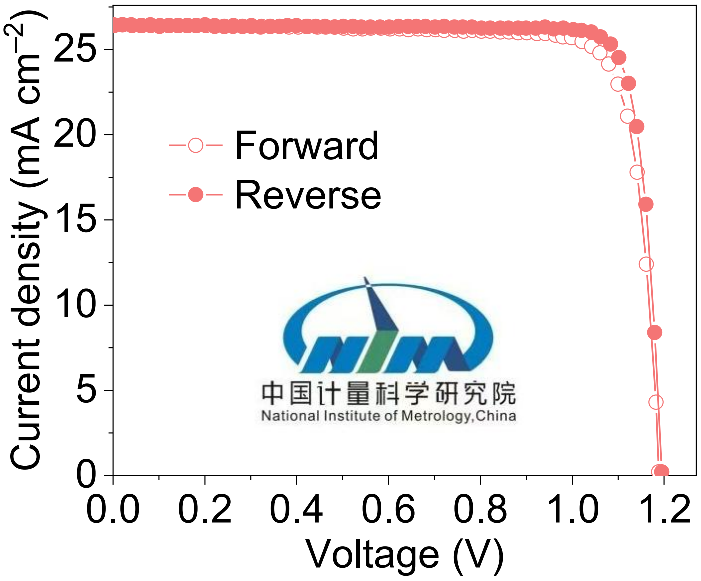

Figure R32. Certified photovoltaic performance of devices obtained at NIM. (**Updated Fig. 5c**)

中国计量科学研究院
National Institute of Metrology,China

Appendix: Summary of the Report
Report No.: GXgf2025-03922
Client: Shaanxi Normal University
Sample: Perovskite Solar Cell
Type/Model: Perovskite Solar Cell
DUT S/N: Y5-M0821-2
Manufacturer: Shaanxi Normal University
Sample temperature: (25±1)°C
Date of Test: 11/28/2025
Environmental conditions: (22 ± 1)°C RH (17± 2)%
Mask: An aperture area of 7.420 mm² (M0821-2, Certificate No.: CDjc2025-05685)

The test has been conducted by the PV Metrology Lab of NIM (National Institute of Metrology, China). Measurement of irradiance intensity and all other measurements are traceable to the International System of Units (SI). The data in this report is only applicable to the samples tested at that time. For more details, please refer to the text of the report.

	Area (mm^2)	I_{sc}(mA)	V_{oc}(V)	P_{max}(mW)
Reverse Scan	7.420	1.961	1.194	2.034
Forward Scan	7.420	1.961	1.188	1.955
	I_{max}(mA)	V_{max}(V)	FF (%)	η (%)
Reverse Scan	1.884	1.080	86.90	27.41
Forward Scan	1.844	1.060	83.89	26.35

I-V Characterization Methods:
JJF 1622-2017: Calibration Specification of Solar Cells: Photoelectric Properties
JJF 2129-2024: Calibration Specification for Perovskite Solar Cells: Photoelectric Characteristic Parameters
Reference Solar Cell:
Type: Mono-Si
Solar Simulator:
Classification: A+AA+ (Double-light source: Xeon and Halogen);
Total irradiance: 1000 W/m² based on I_{sc} of the above Reference Solar Cell.

Figure R33. Certified photovoltaic performance of devices obtained at NIM. (Updated **Supplementary Fig. 40**)

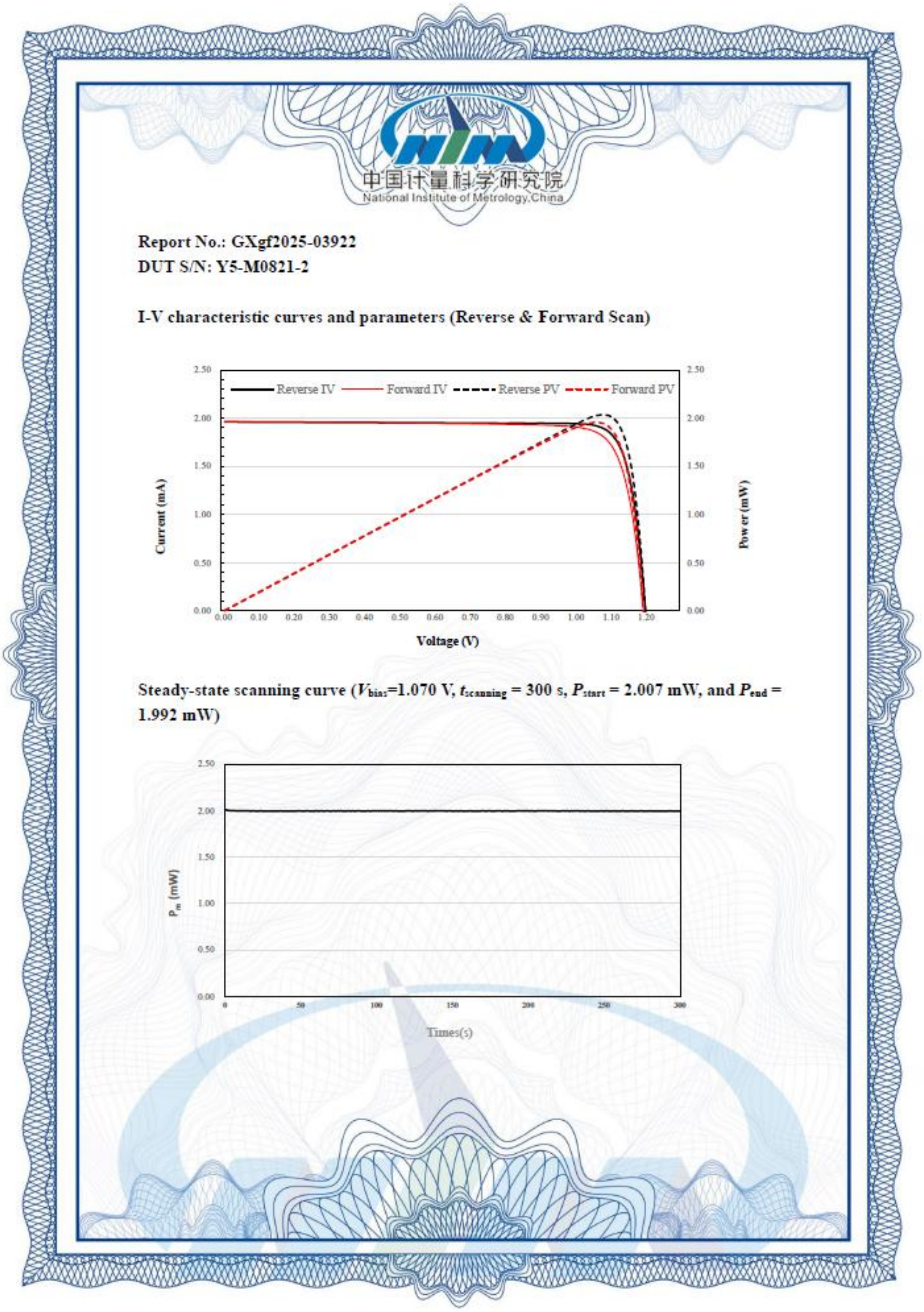
中国计量科学研究院
National Institute of Metrology,China

Report No.: GXgf2025-03922
DUT S/N: Y5-M0821-2

I-V characteristic curves and parameters (Reverse & Forward Scan)

Steady-state scanning curve (V_{bias}=1.070 V, $t_{scanning}$ = 300 s, P_{start} = 2.007 mW, and P_{end} = 1.992 mW)

Figure R34. Certified photovoltaic performance of devices obtained at NIM. (Updated **Supplementary Fig. 41**)

2. The concept of stereoelectronic manipulation is well-proposed. However, looking at the bottom charge contact layer, the authors employed 4PADCB molecules on FTO, which have a long alkyl chain. 4PADCB seems to be not ideal considering the emphasis of stereoelectronic manipulation concept in this work. The authors are invited to comment on the point above, and whether 3PATAT-C3 molecules [J. Am. Chem. Soc. 145, 7528-7539 (2023). https://doi.org/10.1021/jacs.3c00805], for example, would be ideal for further enhancing the performance of devices?

Response: Thanks for strong endorsement of our stereoelectronic manipulation concept and expert insights, which have strengthened the manuscript. The suggestion regarding buried-interface SAMs is indeed a fascinating direction. Our analysis is as follows:

(1) We have carefully reviewed and analyzed the article suggested by the reviewer (*J. Am. Chem. Soc.* 2023, 145, 7528–7539, https://doi.org/10.1021/jacs.3c00805). This is a highly interesting work in which Prof. Atsushi Wakamiya's group designed and synthesized a SAM molecule (3PATAT-C3) featuring multiple phosphonic acid anchoring groups. They demonstrated that multipodal derivatives adopt a face-on alignment on the electrode surface, whereas the monopodal counterpart exhibits a more tilted configuration. Owing to the favorable alignment of the multipodal derivatives, the devices achieved enhanced operational stability and high PCE of up to 23.0%. This study clearly reveals the critical role of SAM molecular conformation at buried hole-transport interfaces in facilitating efficient hole extraction, which conceptually aligns with our stereoelectronic design strategy for optimizing the electron-transport interface. The study's focus on SAM conformation for **hole extraction at a buried interface** is complementary to our stereoelectronic strategy for **passivation at the top interface**. We **cited this study as reference 41** in the revised manuscript (**Page 14**, in the revised manuscript).

(2) Looking ahead, the reviewer's suggestion provides a clear roadmap. A synergistic "dual-interface" approach—combining SAM conformational control at the bottom interface with our stereoelectronic ligands at the top—is a highly promising path toward the efficiency ceiling. As the 3PATAT-C3 molecule is not yet commercially available, we are keen to pursue this through collaboration in subsequent work with Prof. Atsushi Wakamiya's group or other teams with relevant expertise.

3. The DFT calculations are based on Pb-I terminated perovskite surfaces (Figure 1) for describing the Pb-N and Pb-I-pi interactions. I wonder regarding the molecular interactions and their orientations considering the FA/MA-I terminated surfaces, which is an important consideration leading to different chemical interactions. Please see [1] Adv. Mater. 36, 2310203 (2024). https://doi.org/https://doi.org/10.1002/adma.202310203; [2] Nat. Commun. 16, 753 (2025). https://doi.org/10.1038/s41467-025-56068-6; [3] ACS Nano 10, 1126-1131 (2016). https://doi.org/10.1021/acsnano.5b06420.

Response: Thank you very much for your important comment and for drawing our attention to the role of FA/MA–I terminated perovskite surfaces.

(1) We have carefully examined the references highlighted by the reviewer. In the

first two publications (**Figures R35** and **R36**), the authors intentionally selected FAI-terminated surfaces in order to construct specific defect configurations, such as V_{FA} and V_I'. In their DFT models, the amine groups of the ligands were inserted into the V_{FA} sites. However, these models have an inherent limitation in that the vertically inserted configurations result in relatively long interfacial charge-transport distances. In the third reference, the authors constructed an MAI-terminated surface model based on the pristine lattice parameters of $MAPbI_3$ single crystals (**Figure R37**) to investigate the intercalation behavior of MA cations within the Pb–I octahedral framework. Nevertheless, this study did not consider the presence of surface ligands. Despite these limitations, the above works provide valuable model references for our subsequent DFT calculations, particularly for comparing the thermodynamic stability of ligands on FA/MA–I-terminated surfaces versus Pb–I-terminated surfaces.

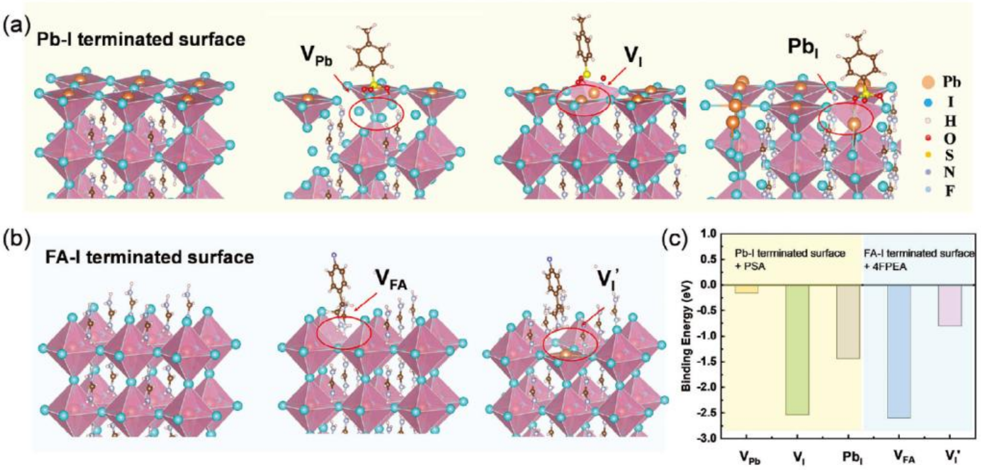

Figure R35. (a) Schematic diagram of the Pb-I terminated surfaces without defect sites and binding interaction between PSA^- and Pb-I terminated surface with the defects of V_{Pb}, V_I and Pb_I. (b) Schematic diagram of FA-I terminated surface without defect sites, and binding interaction between $4FPEA^+$ and FA-I terminated surface with defects of V_{FA} and V_I'. (c) Binding energy comparison between 4FPEAPSA and various defects, including V_{Pb}, V_{FA}, V_I, V_I' and Pb-I. And V_I and V_I' refer to the iodine vacancy at the Pb-I and FA-I terminated surface, respectively. (Copyright from *Adv. Mater.* 2024, 36, 2310203)

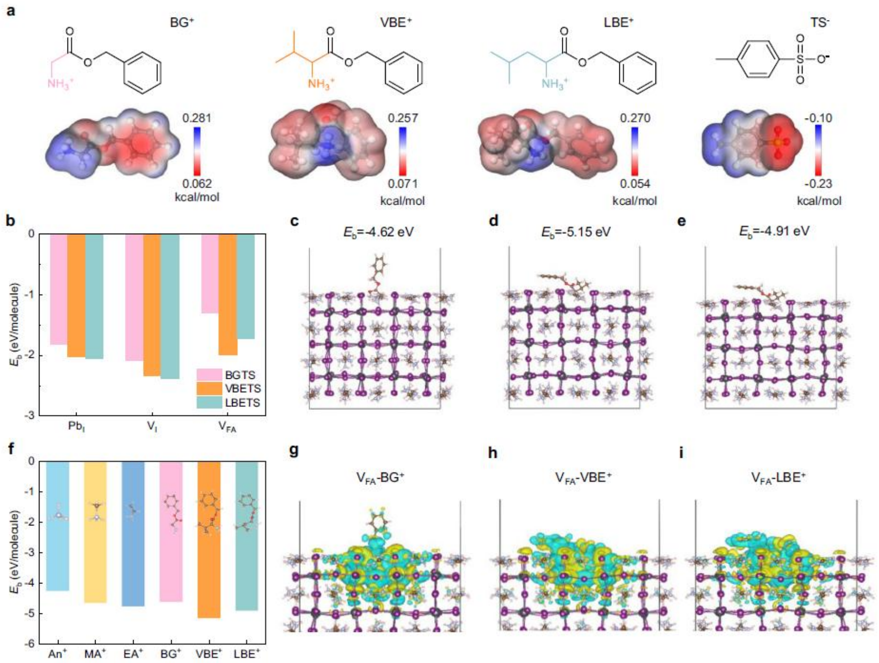

Figure R36. Theoretical screening of organic cations. (a**)** The chemical structures and electrostatic potential of BG^+, VBE^+, LBE^+, and TS^-. (b**)** The binding energy of BGTS, VBETS, and LBETS with $FAPbI_3$ containing Pb_I anti-site, V_I, and V_{FA} defects. (c–e**)** The binding energy of (c) BG^+, (d) VBE^+, and (e) LBE^+ cations with $FAPbI_3$ containing VFA defects. (f**)** Binding energy of An^+, MA^+, EA^+, BG^+, VBE^+, and LBE^+ with $FAPbI_3$ containing VFA defects. (g–i**)** Charge density difference of (g) BG^+, (h) VBE^+, and (i) LBE^+ with $FAPbI_3$ surface encompassing V_{FA} defects (cyan indicates a decrease in charge density, yellow indicates an increase in charge density). (Copyright from *Nat. Commun.* 2025, 16, 753)

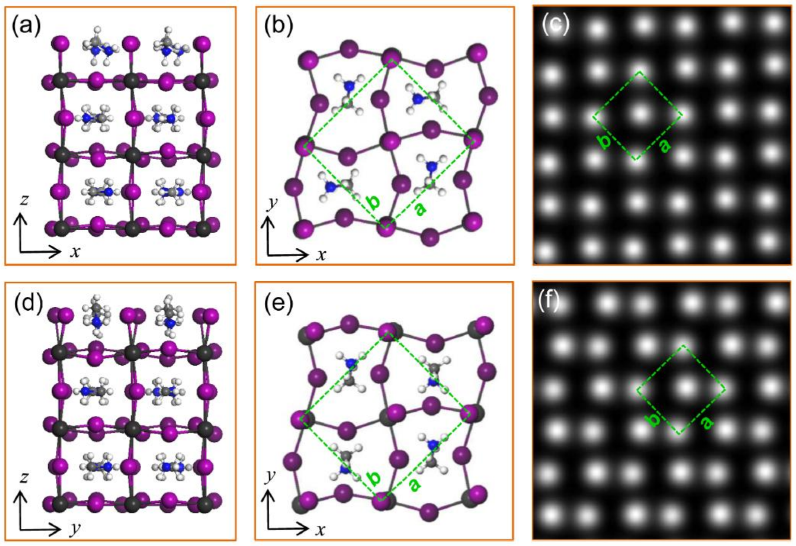

Figure R37. Geometrical structures of MA−I-terminated $MAPbI_3$ (001) surfaces optimized by DFT calculations. (a−c) Side view, top view, and simulated STM image of the zigzag phase. (d−f) Dimer phase. The simulated STM images were acquired by integrating the density of states from 0 to 2.5 eV. (Copyright from *ACS Nano* 2016, 10,

1126−1131)

(2) We have performed DFT calculations to compare the thermodynamic stability of ligand adsorption on Pb–I–terminated and MA/FA–I–terminated perovskite surfaces (see **Figure R38**). The results suggest that ligand adsorption on FA/MA–terminated facets is primarily governed by relatively weak hydrogen-bonding and dipolar interactions (**Table R2**), consistent with previous reports (**Science**, https://www.science.org/doi/10.1126/science.adx1745). As a consequence of these weaker interactions, the calculated adsorption energies on FA/MA–I–terminated surfaces are comparatively small, with E_{ads} (vertical) = –0.45, –0.65, –0.52, and –0.51 eV and E_{ads} (planar) = –0.21, –0.46, –0.36, and –0.44 eV for TFEA, TFBA, TFPA, and TFPmA, respectively. In contrast, stronger adsorption is obtained on Pb–I–terminated surfaces (**Figure R39** and **Table R3**), where E_{ads} (vertical) = –0.97, –1.18, –1.13, and –1.10 eV and E_{ads} (planar) = –0.40, –1.20, –1.31, and –1.37 eV for the same ligands. Taken together, these calculations indicate that ligand adsorption on Pb–I-terminated surfaces is a **consistent thermodynamic preference** relative to that on MA/FA–I-terminated surfaces.

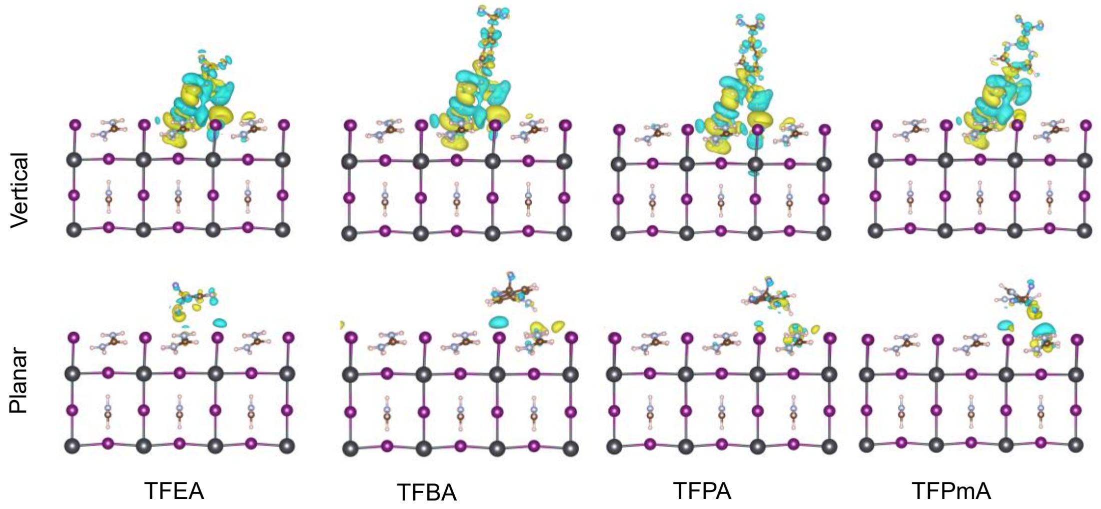

Figure R38. The adsorption configurations of different ligands on the FA-I terminal of perovskite after expanded the k-point grid to (2×2×1) while increasing the Plane-wave cutoff to 500 eV.

Table R2. The adsorption energy of different ligands on the FA-I terminal of perovskite.

	TFEA	TFBA	TFPA	TFPmA
E_{ads} (vertical)	-0.45	-0.65	-0.52	-0.51
E_{ads} (planar)	-0.21	-0.46	-0.36	-0.44
E_{ads} (planar)-E_{ads} (vertical)	**0.24**	**0.19**	**0.16**	**0.07**

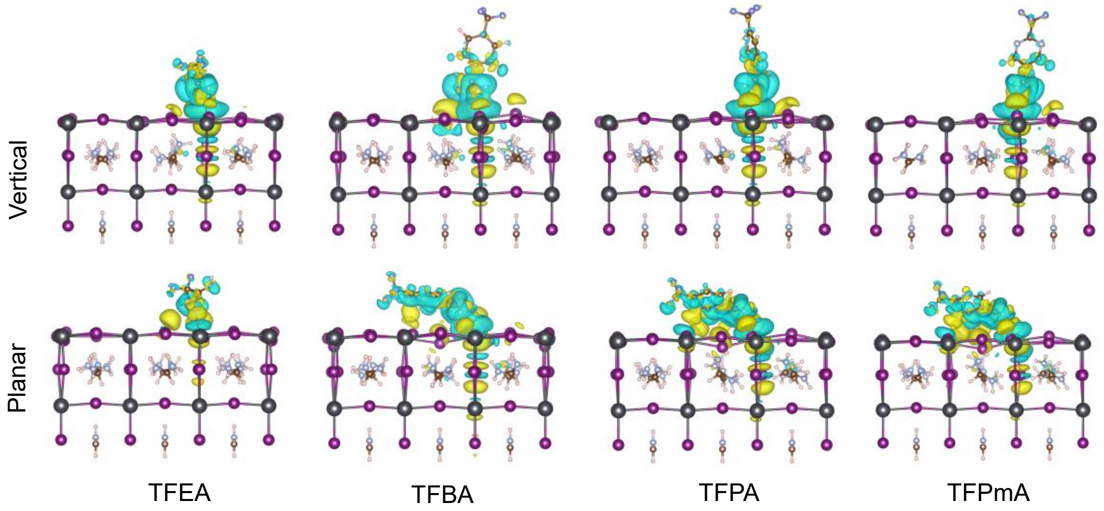

Figure R39. The adsorption configurations of different ligands on the Pb-I terminal of perovskite with the k-point grid to (2×2×1) and the Plane-wave cutoff to 500 eV.

Table R3. The adsorption energy of different ligands on the Pb-I terminal of perovskite.

	TFEA	TFBA	TFPA	TFPmA
E_{ads} (vertical)	-0.97	-1.16	-1.13	-1.10
E_{ads} (planar)	-0.40	-1.15	-1.31	-1.37
E_{ads} (planar)-E_{ads} (vertical)	**0.57**	**0.01**	**-0.18**	**-0.27**

(3) We also notice that our approach of using Pb–I–terminated surfaces is also consistent with previous reports for perovskite surface modelling. For example, a Pb–I–terminated surface was employed to model the perovskite/C_{60} interface when investigating the role of Nd@C_{82}–PMMA in ultrafast interfacial electron extraction, where the authors achieved good agreement with experimental observations (**Figure R40a**, ***Nature*** 2025, 642, 78–84). Similarly, Pb–I–terminated surfaces were used to elucidate the adsorption behavior of different ligands on perovskite surfaces (**Figure R40b**, ***Science*** 2024, 384, 189–193). Recent reports also adopted Pb–I–terminated surface models to describe the interfacial binding energetics at PbI_2-rich $FAPbI_3$ (100)/ligand interfaces (**Figure R40c**, ***Sci. Adv***. 2024, 10, eadq1150) and to investigate ligand-mediated interactions at perovskite/SnO_2 interfaces (**Figure R40d**, ***Science*** 2024, 386, 187–192).

The prevalence of Pb–I-terminated surfaces in perovskite DFT modeling stems from two principal factors. First, high-efficiency devices are often fabricated with slightly PbI_2-rich precursor compositions, which favor Pb–I-rich terminations on the actual perovskite surface. Second, under typical experimental conditions, Pb–I-terminated surfaces are thermodynamically more stable than FA/MA-I-terminated ones. For these reasons, Pb–I-terminated surfaces have become a widely adopted and physically relevant model in high-impact studies of perovskite interface energetics.

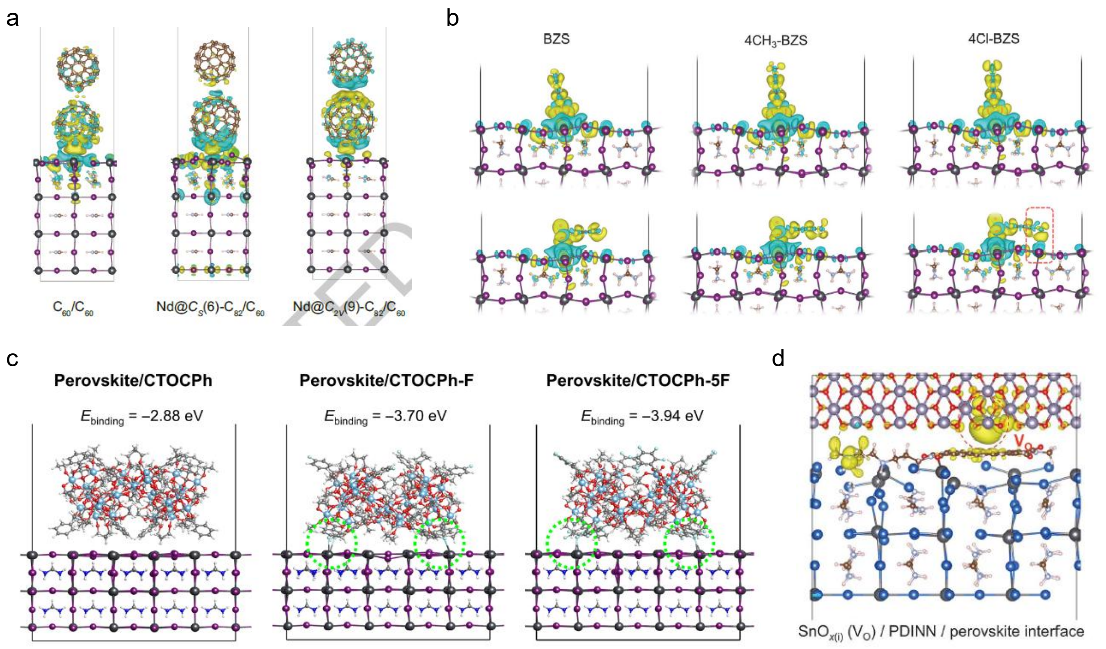

Figure R40. (a) Charge density difference (CDD) distributions of perovskite (with PbI2- terminated $FAPbI_3$ surface)/fullerene slabs (Copyright from *Nature* 2025, 642, 78–84). (b) DFT studies of ligand binding and predicted orientation (Copyright from *Science* 2024, 384, 189–193). (c) Calculated binding energy ($E_{binding}$) for PbI_2-rich $FAPbI_3$ (100)/CT OCPh, PbI_2-rich $FAPbI_3$ (100)/CT OCPh-F, PbI_2-rich $FAPbI_3$ (100)/CT OCPh-5F (Copyright from *Sci. Adv.* 2024, 10, eadq1150). (d) Illustration of the simulated charge distribution (corresponding to CBM) at the $SnO_{x(i)}$ (V_O)/PDINN/perovskite interface (Copyright from *Science* 2024, 386, 187–192).

(4) To further address the reviewer's point, we also extended our analysis to FA/MA–I-terminated surfaces, comparing the stability of planar and vertical adsorption modes $\Delta E = E_{ads}(planar) - E_{ads}(vertical)$ (**Table R2**, **Figure R41**). We find that ΔE becomes less positive with higher N heteroatom incorporation, demonstrating **a reduced stability cost for planar adsorption**. Thus, increasing N content promotes a transition toward planar geometry, thereby corroborating the central adsorption trend established in the main text for different surfaces.

In summary, in light of the reviewer's comments and a broader survey of the literature, we performed complementary calculations on FA–I-terminated surfaces. Together with our original Pb–I-terminated model, these results confirm that: **regardless of the surface termination (Pb–I or FA–I), a higher N heteroatom content in the ligand systematically promotes a transition from vertical to planar adsorption geometries**. This additional analysis indicates the general validity of our proposed adsorption mechanism.

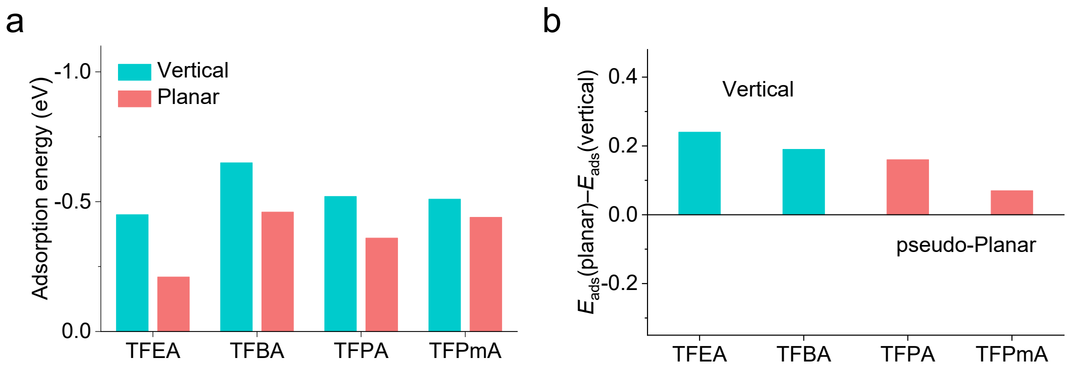

Figure R41. (a) The adsorption energy of different ligands on the FA-I terminal of perovskite after expanded the k-point grid to (2×2×1) while increasing the Plane-wave cutoff to 500 eV. (b) Formation-energy differences ($\Delta E = E_{ads}$(planar) − E_{ads}(vertical)) between vertical and planar adsorption topology for each ligand on the FA-I terminal perovskite surface.

We have also included **Figures R38** and **R41** as **Supplementary Figs. 4** and **5** in the Supplementary Information. Corresponding descriptions have been added both in the main text and in **Supplementary Note 1**, as detailed below:

"This thermodynamic preference shift is also observed on FA–I-terminated surfaces (**Supplementary Figs. 4** and **5,** and **Supplementary Note 1**)." (**Page 5**, in the revised manuscript)

"On Pb–I–terminated surfaces, the formation-energy differences ($\Delta E = E_{ads(planar)} - E_{ads(vertical)}$) for TFEA, TFBA, TFPA, and TFPmA decrease sequentially from 0.57 to 0.01, −0.18, and −0.27 eV, respectively. For TFBA, ΔE is close to zero (0.01 eV), suggesting no strong configurational preference between vertical and planar adsorption. This trend indicates a gradual thermodynamic shift in preferred adsorption topology from predominantly vertical (TFEA), to nearly non-preferential or mixed configurations (TFBA), and further toward planar geometries (TFPA and TFPmA) as the number of N-heteroaromatic units increases.

In comparison, the calculated adsorption energies on FA–I–terminated surfaces are relatively smaller, with E_{ads}(vertical) values of −0.45, −0.65, −0.52, and −0.51 eV and E_{ads}(planar) values of −0.21, −0.46, −0.36, and −0.44 eV for TFEA, TFBA, TFPA, and TFPmA, respectively. Further analysis of the corresponding formation-energy differences (ΔE) reveals a similar decreasing trend with increasing N content, indicating that higher N incorporation progressively lowers the energetic penalty for planar adsorption. Collectively, these results suggest that increasing N heteroatom content promotes a tendency toward planar adsorption geometries across different surface terminations, thereby corroborating the central adsorption trend established in the main text." (In the **Supplementary Note 1**)

4. Please provide explanation and reference(s) validating the rationale of NMR experiments for studying the chemical interaction of passivating molecules (TFEA,

TFBA, TFPA, and TFPmA) with PbI_2 in liquid states (i.e., in d6-DMSO). Are these interactions equivalent to those on the actual perovskite solid films with passivation molecules?

***Response*:** Thanks for this important methodological point, and we have given it thorough consideration based on the suggestions:

(1) Value of Solution-Phase NMR: Liquid-state NMR in d^6-DMSO is a sensitive, quantitative probe for Pb^{2+} coordination, revealing ligand-specific chemical-shift perturbations, peak broadening, and diffusion changes. It identifies coordinating donor atoms (e.g. pyridinic N), estimates binding stoichiometry, and ranks relative binding strengths—information often difficult to obtain with equivalent resolution by solid-state techniques.

(2) Distinction Between Solution and Solid States: We explicitly acknowledge that solution-phase measurements are not identical to solid-state surface chemistry. DMSO can compete for coordination, and molecular dynamics in solution differ from the constrained interface geometry. Therefore, we interpret NMR data as reflecting the **intrinsic coordination propensity** of each ligand toward Pb^{2+}, not as direct proof of identical configurations on films.

(3) Corroboration with Solid-State Evidence: To bridge this gap, we correlate NMR trends with multiple complementary solid-state characterizations: XPS shifts of Pb/N, FTIR signatures, nano-IR mapping, TRPL/QFLS improvements, and device metrics. Consistently, ligands showing stronger Pb-coordination in solution yield stronger interfacial passivation and better optoelectronic outcomes in films. This combined approach is also consistent with previous reports (e.g. *Science* **2024**, 384, 189; *Nat. Energy* **2023**, 8, 515).

(4) For better clarification, we have added a concise methodological justification and discussion of these points to the **Supplementary Note 2**.

"NMR measurements and sample preparation: To investigate the coordination interaction between the passivating ligands (TFEA, TFBA, TFPA, and TFPmA) and PbI_2, liquid-state ^1H NMR experiments were carried out in deuterated DMSO (d_6-DMSO). Each ligand and its equimolar mixture with PbI_2 (1:1 molar ratio) were dissolved in d_6-DMSO and analyzed by ^1H NMR. Upon addition of PbI_2, distinct upfield shifts were observed for the amidino and aromatic proton signals, indicating strong ligand–Pb^{2+} coordination. These ligand-dependent spectral changes reveal a clear hierarchy of coordination strength that correlates with the trends in defect passivation and device performance." (In the updated **Supplementary Note 2**)

"Finally, a consistent coordination hierarchy among the ligands is established through multiple complementary techniques. Solution-phase ^1H NMR (chemical shifts of $\delta H\alpha$ and $\delta H\beta$) and solid-state characterizations—including core-level shifts of Pb, I, and N in XPS and vibrational changes of key functional groups in FTIR—provide convergent evidence. Together, these results substantiate strong and multicenter ligand and Pb-I coordination at the perovskite interface." (In the updated **Supplementary Note 2**)

5. Please provide details of experimental conditions for FTIR results (Figure 2d). Description of "blue", "mid", and "red" shown in Figure 3c is lacking in the main text.

Response**:** Thanks for meticulous review. We have incorporated these missing details into the updated version of the manuscript:

(1) The experimental conditions for FTIR measurements (**Fig. 2e**) have been added to the Methods section of the revised manuscript. Specifically, the spectra were recorded using a FTIR spectrometer employing the standard KBr pellet method. All powder samples were finely ground with spectroscopic-grade KBr, and spectra were collected under ambient conditions in the range of 4000–400 cm^{-1} with a resolution of 0.4 cm^{-1}.

"Fourier-transform infrared (FTIR) spectra were collected using a Bruker Tensor II spectrometer equipped with KBr beam splitter." (1.5 Film characterization, in the Methods section of **Supplementary Information**)

"FTIR measurements and sample preparation: FTIR measurements were performed on KBr pellets (sample:KBr = 1:100) pressed under vacuum, with spectra acquired from 4000–400 cm^{-1} at 0.4 cm^{-1} resolution. Interaction with PbI_2 broadened the –C=N stretching vibration, with FWHM increases of 49% (TFEA), 51% (TFBA), 59% (TFPA), and 107% (TFPmA). This trend reflects stronger ligands-perovskite interactions at the presence of multicenter interactions (TFBA, TFPA, TFPmA)." (In the **Supplementary Note 2**)

(2) Regarding the color-coded "blue", "mid", and "red" regions in **Fig. 3c (updated Fig. 3d)**, we have clarified these descriptions in the **Fig. 3d caption**. These regions correspond to spatially resolved infrared spectra extracted from the areas highlighted in **Supplementary Fig. 21** with the nano-IR. "Blue" represents the infrared spectral curve extracted from the blue region in **Supplementary Fig. 21**; "red" denotes the infrared spectral curve extracted from the red region; and "mid" indicates the infrared spectral curve extracted from the intermediate transition region between blue and red.

"Nano-infrared (nano-IR) line-plot spectra acquired from the perovskite surface mappings (**Supplementary Fig. 21,** the blue, mid, and red lines corresponding to the blue, intermediate, and red mapping, respectively)" (**Fig. 3d caption**, **Page 11**, in the revised manuscript)

6. The poor coverage less than 50% of passivating molecules described in this work is puzzling. I wonder if there are monolayers of the passivating molecules covering the entire perovskite surface, which cannot be detected due to sensitivity limitations of the instrument? At the same time, agglomeration of passivating molecules forms, which can be detected by authors' instrument due to signal enhancement. Authors are invited to comment on the sensitivity of the instrument for generating Figure 3d and perhaps adjust the wordings of "~7.7% coverage … TFPmA, ~46.3%"?

Response**:** Thanks for insightful question regarding interfacial effects, Below, we provide a detailed discussion addressing this point:

(1) Instrumental Sensitivity and Data Interpretation: We agree that the precision of

many characterization techniques is inherently limited by instrumental sensitivity. In our case, the hyperspectral nano-IR technique, based on tip-enhanced near-field photothermal detection with a depth of only a few nanometers, lacks the sensitivity to resolve a true monolayer. Instead, it detects regions with sufficient signal contrast from collective vibrational modes or molecular aggregates. Therefore, the "<50% coverage" in Figure 3d reflects **relative molecular coverage** rather than an absolute geometric value.

(2) Clarification in the Revised Manuscript: We have revised the text to explicitly state that the reported coverage values (e.g., 7.7%–46.3%) represent **relative signal-active surface fractions** from multivariate analysis, not absolute geometric coverage. This clarification now appears on Page 9 and in **Supplementary Note 3**:

"Multivariate analysis (**Fig. 3c**, **Supplementary Fig. 22**, **and Supplementary Note 3**) resolved four relatively signal-active adsorption modes: sparse fractal aggregates (TFEA, ~7.7% relative signal-active fractions), discontinuous superstructures (TFBA, ~25.7%), aligned nanowires (TFPA, ~33.6%), and near-complete epitaxial nanodomains (TFPmA, ~46.3%)." (**Page 9**, in the revised manuscript)

"The relative signal-active fractions of the ligands on the perovskite surface were performed using the image processing software Photoshop. We used the ratio of the near red pixel area representing the distribution of ligands to the total pixels of the image to estimate the relative signal-active fractions of ligands. In this work, the total pixels of the image were 41208 and the near red area pixels representing TFEA, TFBA, TFPA and TFPmA were 3179, 10584, 13849 and 19074, respectively. And hence the corresponding relative signal-active fractions values were determined to be 7.7%, 25.7%, 33.6% and 46.3%, respectively." (In the **Supplementary Note 3**)

7. The claim of "TFPmA achieved intra-grain uniformity, reducing aggregation size by 42% versus TFEA (~57 vs. ~134 nm), establishing a conformal passivation network that suppresses interfacial traps." needs clarification. Authors provide sizes of aggregated features from TFEA (~134 nm) and TFPmA (~57 nm) images in Figure 3d. The authors are invited to provide the analysis based on statistics. Analyses based on single images seem not conclusive as the image sizes are small (scale bars are 60 nm).

Response**:** We appreciate the reviewer's careful and insightful examination of the nano-IR spectra. We have conducted an in-depth analysis as detailed below:

(1) We have now performed statistical analysis of the nanoscale aggregation domains across multiple aggregation size obtained from distinct regions (12~35 areas for different sample, **Figure R42**). The statistics of domain size are provided in **Table R4**. The average lateral aggregation sizes were determined as 100 ± 35 nm for TFEA, 61 ± 13 nm for TFBA, 50 ± 17 nm for TFPA and 39 ± 9 nm for TFPmA, confirming a statistically significant 39% reduction in domain size. These results demonstrate that the data presented in **Fig. 3d** are representative of the region surface morphology rather than a single maximum value.

(2) For the concern about the relatively small image size, we need to provide further clarification here. Given the sensitivity limits of the current instrumentation, it remains

challenging to resolve extremely subtle variations within the adsorption region at larger spatial scales. Precisely for this reason, we employ smaller dimensions to magnify microscopic areas, thereby facilitating clearer observation of nanoscale surface topography.

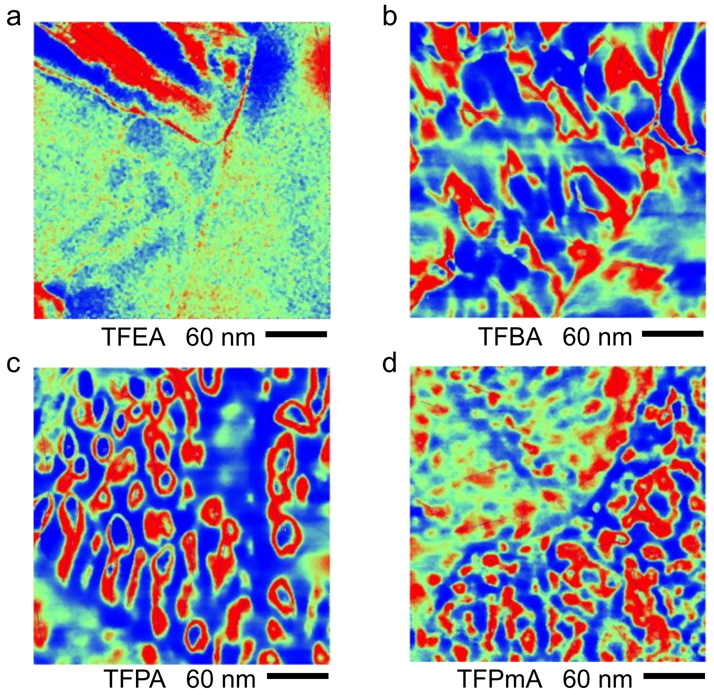

Figure R42. Statistical analysis of the nanoscale aggregation domains across multiple aggregation size obtained from distinct regions. (**Supplementary Fig. 23**)

Table R4. The statistics of domain size obtained from distinct regions.

Sample	Quantity	Maximum (nm)	Average (nm)
TFEA	12	167	100 ± 35
TFBA	22	90	61 ± 13
TFPA	30	85	50 ± 17
TFPmA	35	56	39 ± 9

(3) Guided by the reviewer's suggestions, we updated the manuscript accordingly to further enhance its rigor. We have added **Figure R42** and **Table R4** as **Supplementary Fig. 23** and **Supplementary Table 1** to the supporting information for the revision, and made the following modifications to the corresponding sections in the main text.

"TFPmA achieved intra-grain uniformity, reducing average aggregation size by 39% versus TFEA (~39 vs. ~100 nm, **Supplementary Fig. 23**, and **Supplementary Table 1**)" (**Page 9**, in the revised manuscript)

8. Showing only the Pb 4f and I 3d spectra (Figure 2a,b) is not sufficient to claim on the charge redistribution of Pb-I frameworks. Authors are invited to provide the N 1s region of the corresponding TFEA, TFBA, TFPA, and TFPmA molecules after deposition on the perovskite layers. Please provide the additional C 1s and F 1s regions for the above measurements in SI as it could provide important database for readers.

***Response*:** We appreciate insightful comment regarding potential confounding factors (surface stoichiometry, defects, charging) for the XPS binding energy shifts. We agree this requires careful evaluation and have conducted additional experiments to clarify this issue.

(1) To isolate the effect of surface stoichiometry, we prepared three perovskite films with varying surface Pb/I ratios: a control with +7% excess PbI_2 (as per the manuscript protocol), one with +15% excess PbI_2, and one with a -5% PbI_2 deficiency. All samples were transferred in a sealed, nitrogen-filled chamber to prevent air exposure.

Figure R5 presents the XPS results for these films prior to TFPmA ligand treatment. Compared to the control, the +15% excess PbI_2 film showed only minor shifts (< 0.15 eV) in the Pb 4f and I 3d core levels. In contrast, the -5% PbI_2-deficient film exhibited a more substantial shift of > 0.3 eV. Given that the stoichiometry was identical for all films before surface treatment, the influence of surface stoichiometry variations can be negligible.

Figure R6 shows the XPS results after TFPmA ligand treatment. This treatment induced a positive binding energy shift in the Pb 4*f* peaks by 0.23 eV, 0.17 eV, and 0.33 eV for the +15% excess PbI_2, control (+7%), and -5% deficient films, respectively. These consistent shifts across different starting surfaces confirm that the observed effect originates from ligand-induced surface modification.

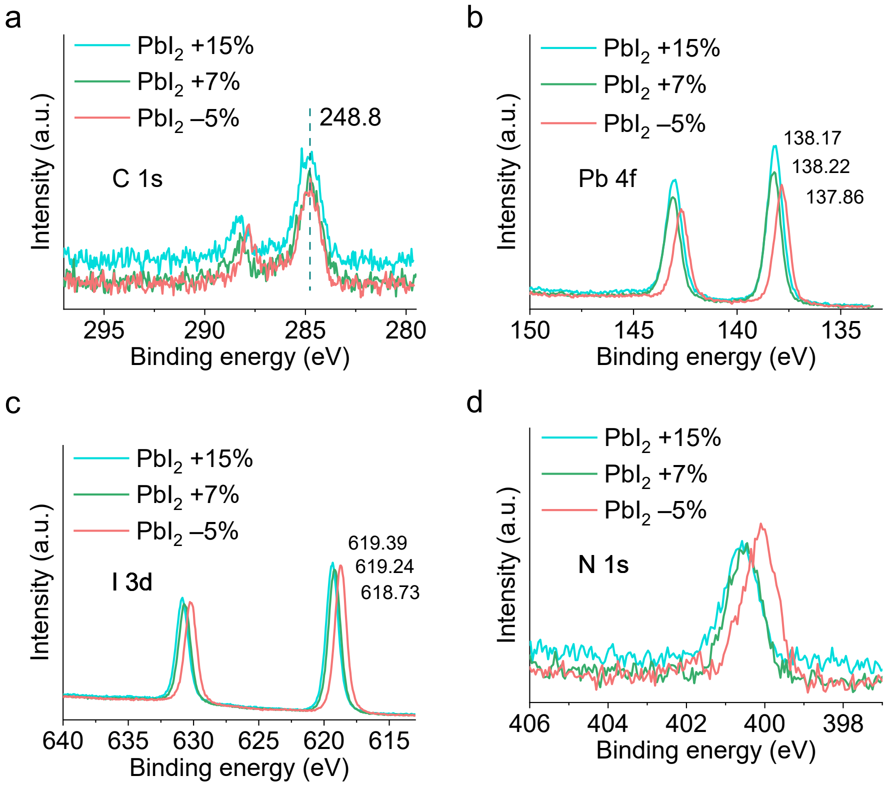

Figure R43 XPS spectra of the three perovskite films. Variations in different surface stoichiometry (i.e., PbI_2 enrichment or iodide deficiency).

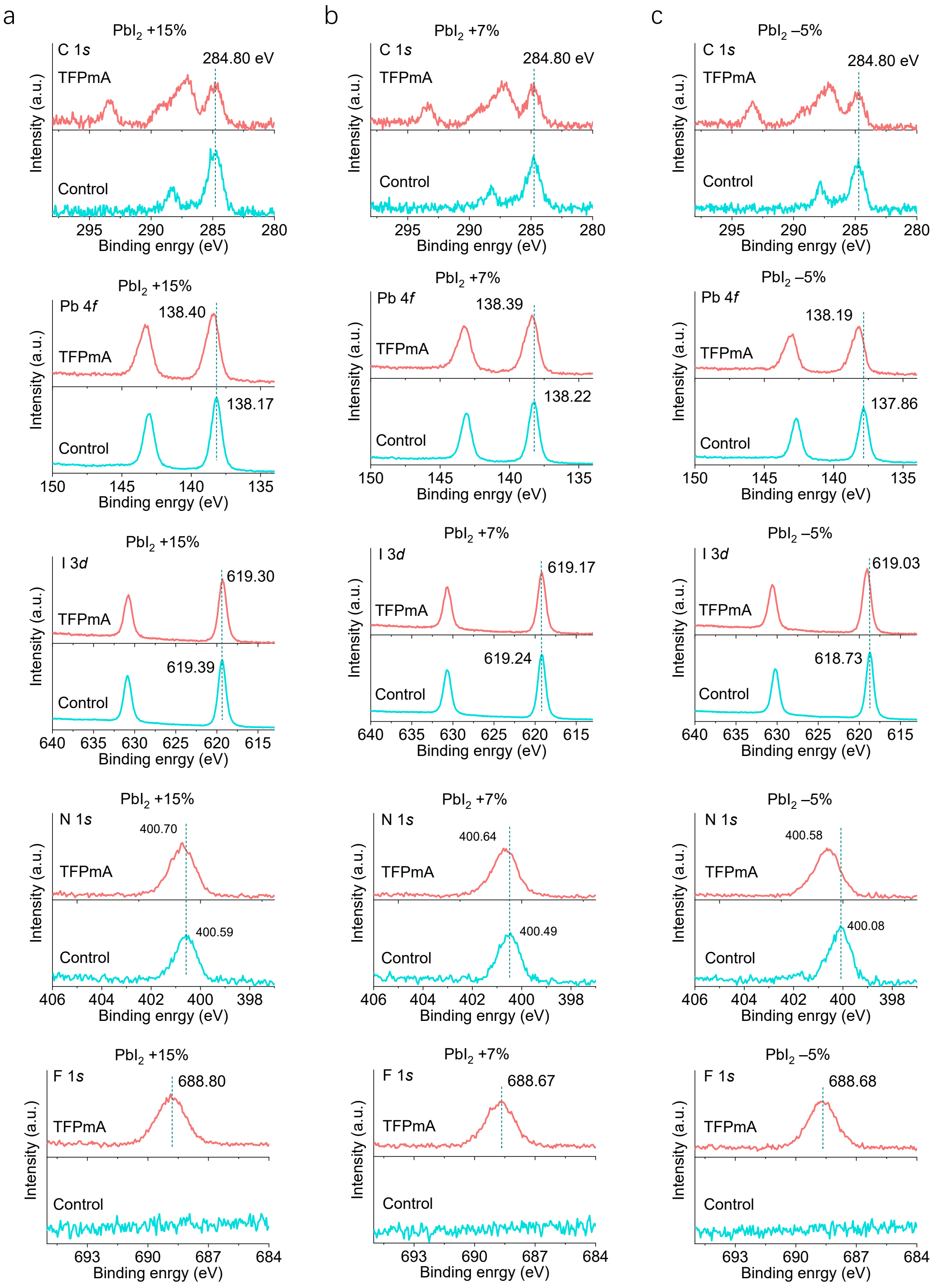

Figure R44 XPS spectra of the three perovskite films (different surface stoichiometry) after surface ligand modification.

In addition, we also checked the impact of precursor composition (e.g., $FA_{0.85}MA_{0.1}Cs_{0.05}Pb(I_{0.9}Br_{0.1})_3$, $FA_{0.9}MA_{0.05}Cs_{0.05}PbI_3$, and $FA_{0.95}Cs_{0.05}PbI_3$). While variations in bulk stoichiometry did induce **minor Pb 4*f* shifts (<0.15 eV)** (**Figure R45**), ligand modification on films of identical composition reproducibly induced a

significantly larger shift of ~0.2 eV in both Pb 4*f* and I 3*d* core levels (**Figure R46**). Since the stoichiometric composition is identical for control and ligand-treated films, the larger shift (~0.2 eV) arises from ligand-induced surface modification, not from bulk compositional changes.

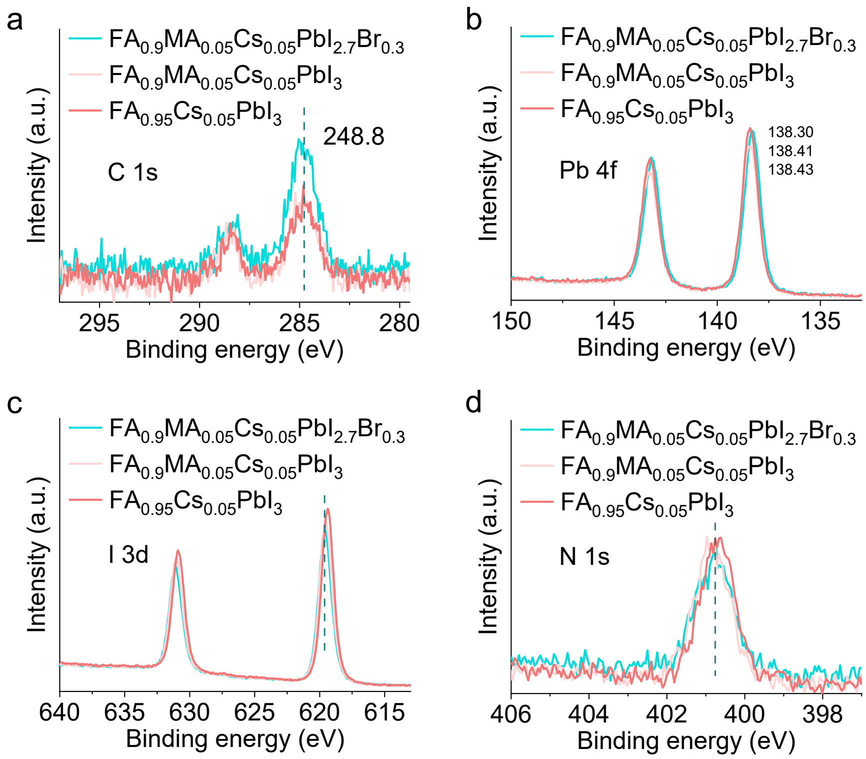

Figure R45. XPS spectra of the three perovskite films. Variations in precursor formulations (i.e., different precursor compositions) lead to detectable shifts in the Pb 4*f* and I 3*d* binding energies.

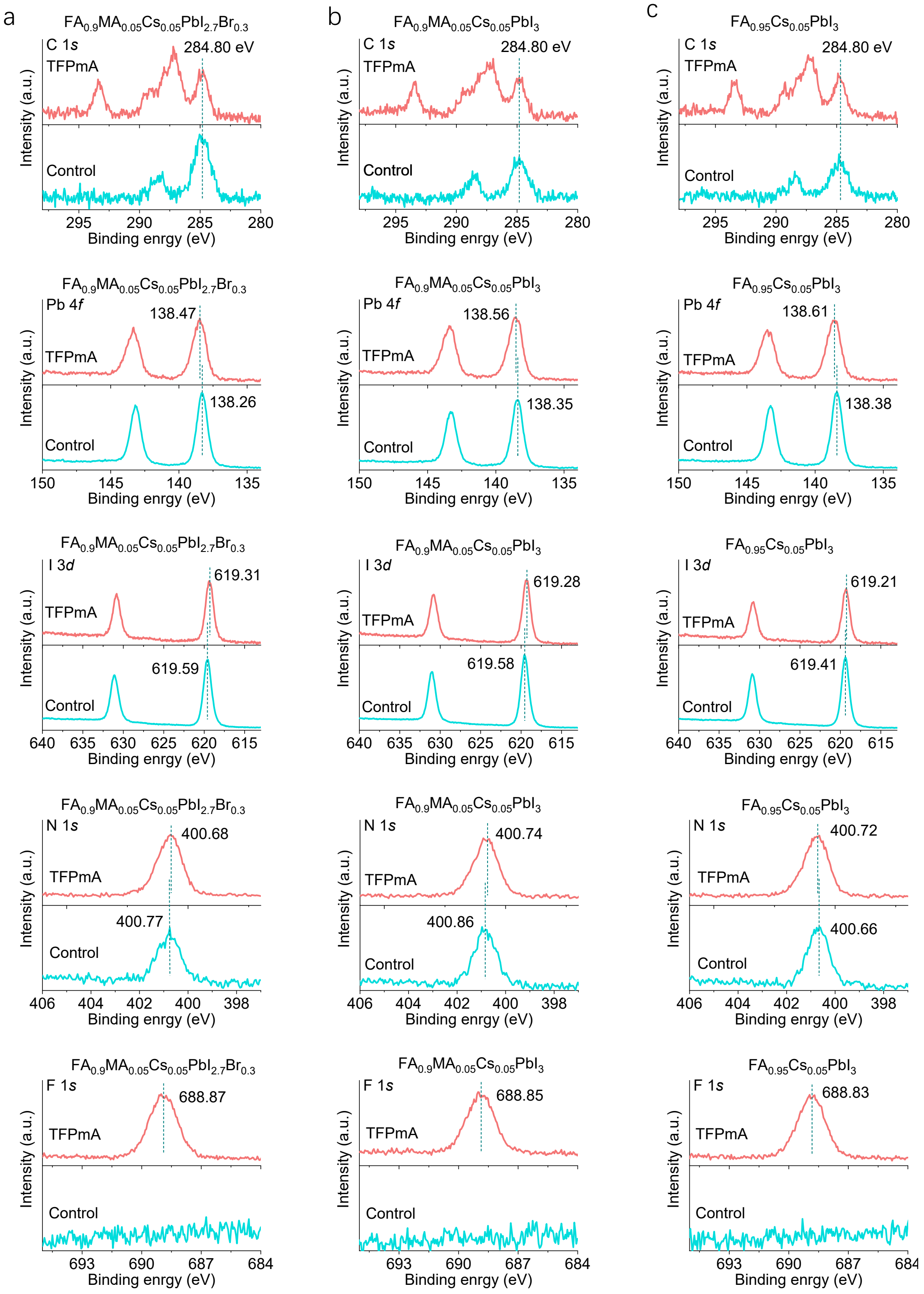

Figure R46. XPS spectra of the three perovskite films (different precursor compositions) after surface ligand modification. All three films exhibit a pronounced binding-energy shift of ~0.2 eV in both the Pb 4*f* and I 3*d* peaks following ligand treatment.

(2) We next evaluated the potential influence of defect formation. To introduce

defects, unmodified, unencapsulated reference films were aged under continuous illumination at 50 °C for 48 hours. Subsequent XPS analysis (**Figure R47**) revealed substantial shifts toward lower binding energies for both core levels (Pb 4f: 0.28 eV; I 3d: 0.48 eV). This pattern is distinct from that induced by ligand modification, where the Pb 4f shifts to *higher* binding energy while I 3d shifts to *lower* energy. Furthermore, the defect-induced shifts are significantly larger in magnitude, accompanied by a marked decrease in the N 1s signal. These results confirm that while defects can alter binding energies, the opposite direction of the Pb 4f shift and the smaller magnitude of change observed in our ligand-treated samples indicate that the ~0.2 eV shifts originate primarily from surface modification, not defect formation.

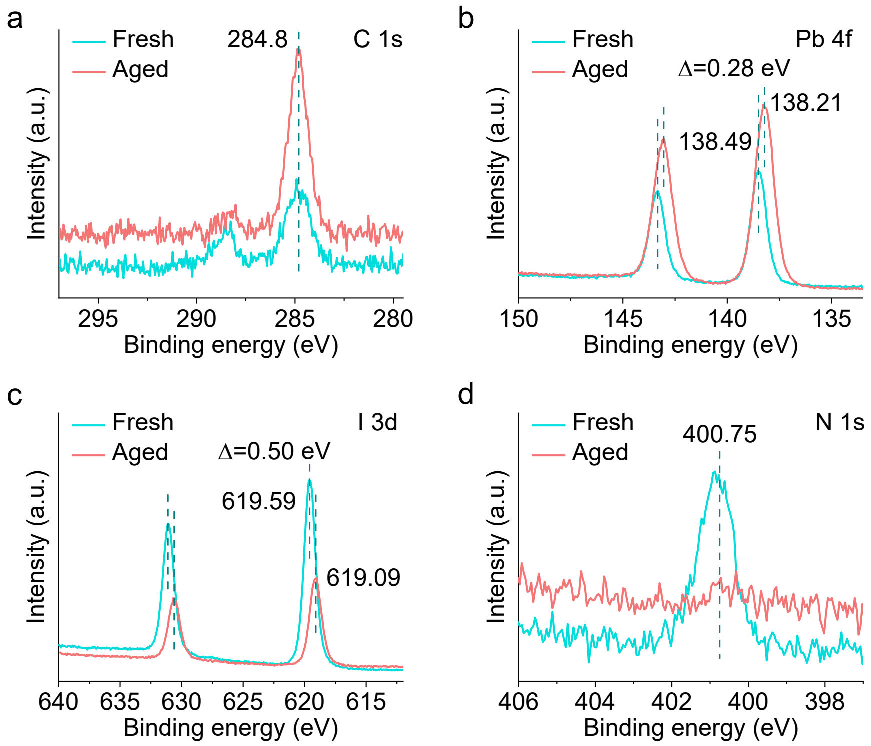

Figure R47. XPS spectra of the aged perovskite films. Upon aging (associated with substantial defect formation), both the Pb 4*f* and I 3*d* binding energies shift markedly toward lower binding energies, accompanied by a pronounced decrease in the N 1s signal. These results indicate that surface defects can significantly alter the XPS response.

(3) To definitively address the possibility of air-exposure artifacts, we repeated the sample preparation and XPS measurement three times under rigorous, uninterrupted nitrogen protection. The results (**Figure R48**) consistently reproduced the key signature: ligand treatment induces a shift of the Pb 4f peak to higher binding energy and the I 3d peak to lower binding energy. Furthermore, the concomitant shift of the N 1s peak to lower binding energy, along with a clear increase in its intensity (**Figure R48e**), provides direct evidence of ligand attachment and strong interaction with the Pb–I framework. This confirms that the observed spectral changes are intrinsic and attributable to ligand bonding, not to surface contamination during transfer.

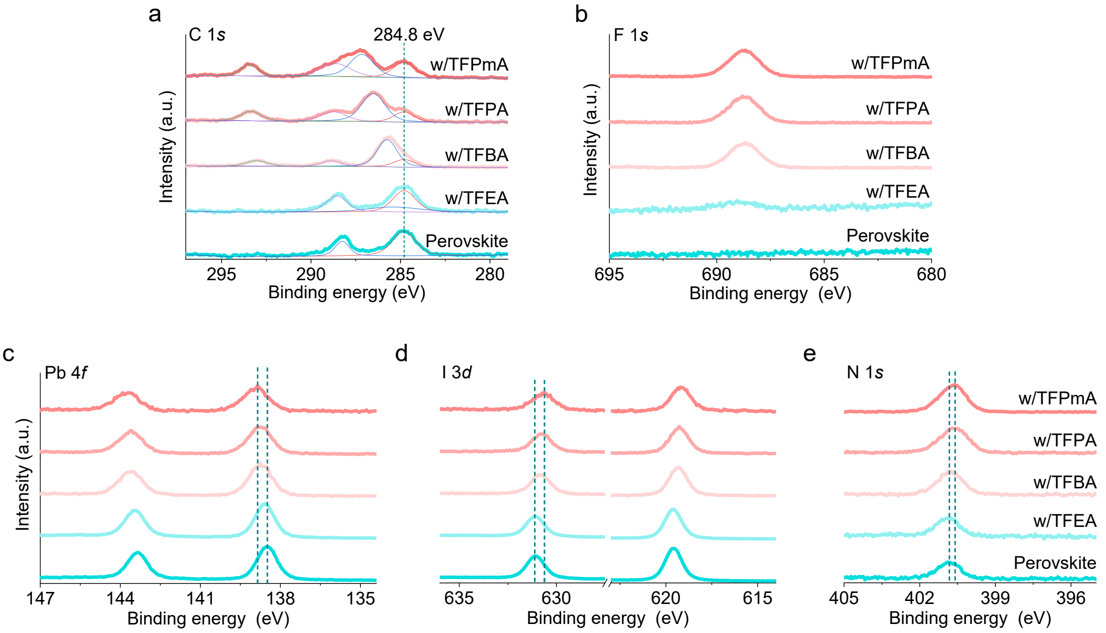

Figure R48. XPS spectra of the ligand-modified perovskite films. The Pb 4*f* peak shifts toward higher binding energy, while the I 3*d* peak shifts toward lower binding energy, both Pb 4*f* and I 3*d* signals show reduced intensity, indicating strong interactions between surface Pb-I framework and the ligand molecules and confirming ligand coverage on the perovskite surface. The N 1*s* peak exhibits a slight shift toward lower binding energy along with increased intensity, consistent with the adsorption of the ligand's N-heterocyclic moieties.

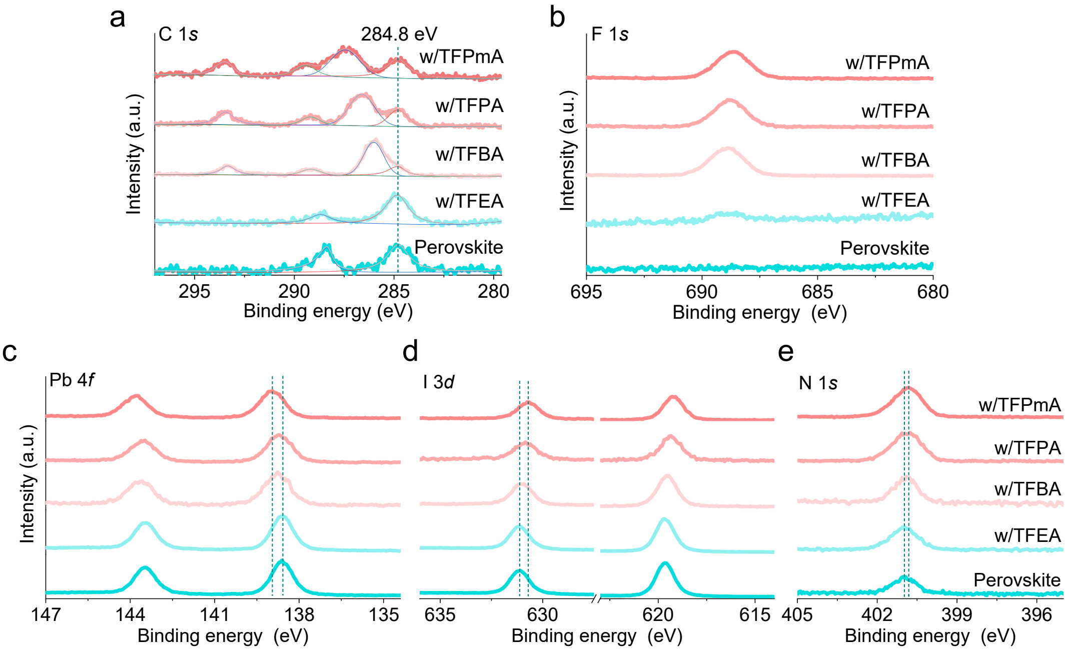

Figure R49. XPS spectra of the perovskite/ligands film after sample charging.

(4) Finally, to assess the impact of sample charging, we intentionally induced charging during an XPS acquisition on a perovskite/ligand film and then repeated the measurement (**Figure R49**). The spectral trends remained consistent with our standard

results (c.f., **Figure R48**): Pb 4f shifted to higher binding energy, while I 3d and N 1s shifted to lower binding energy, with the N 1s signal intensity markedly increased. This confirms that the reported shifts are intrinsic and not artifacts of charging, further validating the reliability of our XPS data.

(5) In direct response to the reviewer's comments and supported by these additional validation experiments, we have updated the manuscript accordingly. The main text now incorporates the N 1s XPS analysis to strengthen our conclusions. Furthermore, the C 1s and F 1s spectra have been added to the Supplementary Information (**Supplementary Figs. 6/7**, corresponding to **Figures R50/R51**). An updated version of **Fig. 2c** (now **Figure R52**) is also included, and all corresponding textual revisions have been made.

"(see details in **Supplementary Note 2**). The XPS spectra of calibrated C 1*s* and F 1*s* references for $FAPbI_3$-based perovskite with ligands are provided in **Supplementary Figs. 6** and **7**. As shown in **Figs. 2a–c**, monotonic binding energy shifts were observed in Pb $4f_{7/2}$ (138.49 → 138.84 eV), I $3d_{3/2}$ (631.09 → 630.63 eV), and N 1*s* (400.81 → 400.62 eV) of the perovskite/ligand systems with increasing N-heteroaromatic content." (**Page 7**, in the revised manuscript)

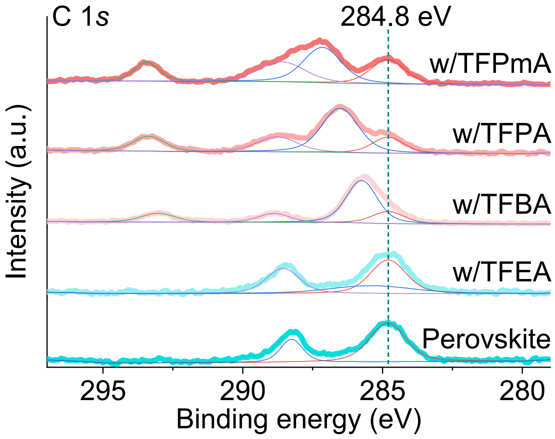

Figure R50. The XPS spectrum of C 1*s* from perovskite films treated with TFEA, TFBA, TFPA, or TFPmA. (**Supplementary Fig. 6**)

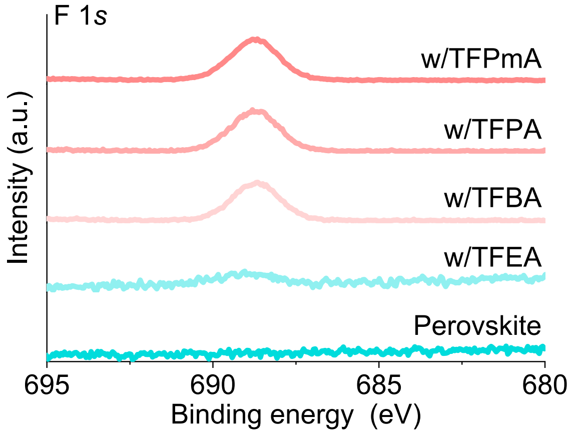

Figure R51. The XPS spectrum of F 1*s* from perovskite films treated with TFEA, TFBA, TFPA, or TFPmA. (**Supplementary Fig. 7**)

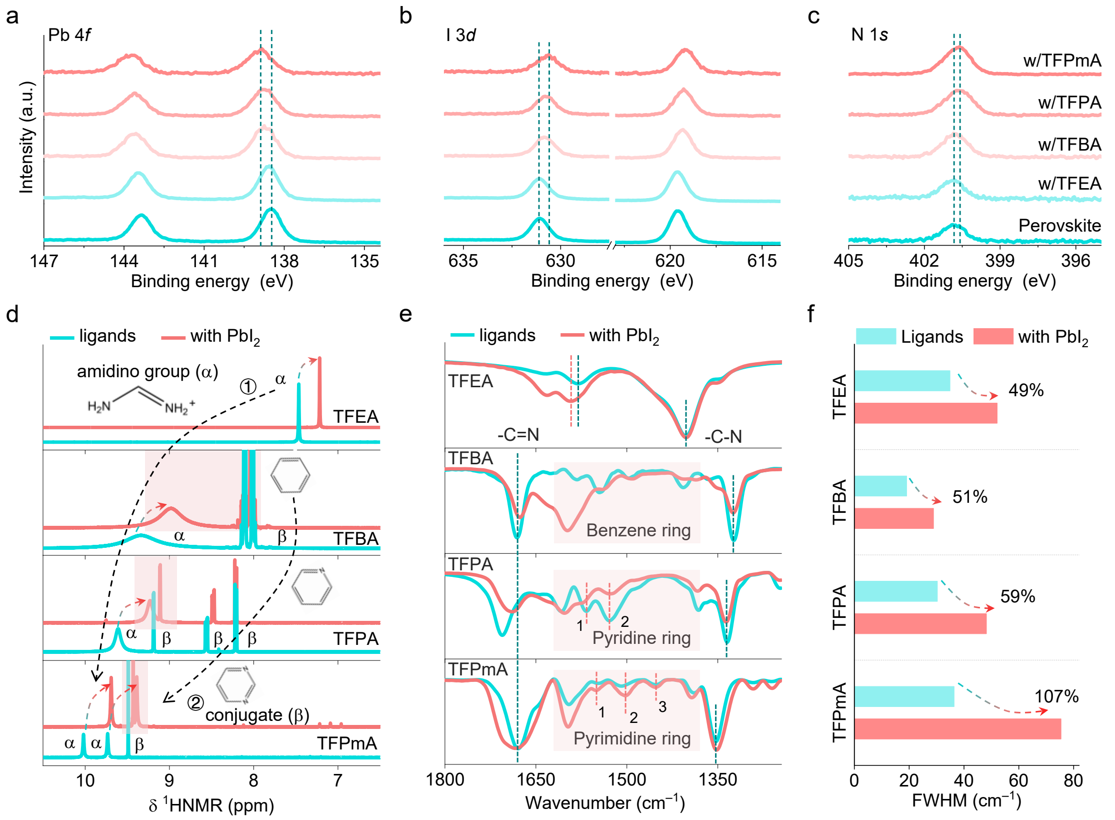

Figure R52. The interaction of ligands with the perovskite framework. XPS spectra of (a) Pb 4*f*, (b) I 3*d* and (c) N 1*s* from perovskite films treated with TFEA, TFBA, TFPA, or TFPmA. (d) 1H-NMR spectra of different ligands interacting with PbI_2. (e) FTIR spectra of different ligands interacting with PbI_2. (f) Full width at half-maximum (FWHM) of the -C=N vibrational peak extracted from the FTIR spectrum. (**Updated Fig. 2**, in the revised manuscript)

(6) To ensure reproducibility and clarity regarding the XPS measurements, we have expanded **Supplementary Note 2** to include a comprehensive description of the sample preparation, transfer protocols, and detailed instrumental measurement conditions.

"XPS measurements and sample preparation. Thin films for XPS characterization were prepared using the same deposition procedures as for functional devices. Following surface ligand modification, all samples were handled entirely within a nitrogen-filled glovebox and transferred to the XPS analysis chamber via an airtight transfer vessel under continuous N_2 atmosphere. This protocol minimized exposure to ambient air, light, and heat, thereby ensuring surface integrity prior to measurement. Spectra were acquired using a monochromatic Al Kα X-ray source (1486.68 eV) with the analysis chamber base pressure maintained between 1.0×10^{-9} and 5.0×10^{-8} mbar. High-resolution scans were recorded at a pass energy of 20 eV." (In the updated **Supplementary Note 2**)

9. A brief discussion on how the conduction band minimum values were extracted for generating Figure 3e is needed based on Figures S15-17.
Are the VBM regions in Fig. S15 plotted in linear or log scales?
Would it make more sense to represent Figure 3e aligning the Fermi levels of individual functional layers?
Please provide authors' rationale for such a vacuum level alignment representation of the device structures.

***Response*:** Thanks for this insightful comment. We have clarified the methodology used for extracting the energy levels and the rationale for the alignment scheme.

(1) The work functions (WF) were derived from the secondary electron cutoff (E_{cutoff}) region (**Supplementary Fig. 24a**). The valence band maximum (VBM) values were determined as VBM = 21.22 − E_{cutoff} + E_f, where 21.22 represents the photon energy of the HeI resonance ray (wavelength 584 Å), E_f derived from the linear extrapolation of the low-binding-energy onset in UPS spectra (**Supplementary Fig. 24b**, plotted on a **linear scale**). Similarly, **Supplementary Fig. 25** shows the UPS measurements for 4PADCB and C_{60}, respectively. The conduction band minima (CBM) were calculated as CBM = VBM + Eg, where Eg was obtained from the optical Tauc plots (**Supplementary Fig. 26**). And the corresponding energy level parameters are presented in **Supplementary Table 2** in the Supplementary Information.

(2) In **Fig. 3e**, we present the vacuum-level alignment because it most directly reflects the intrinsic electronic states of individual layers prior to Fermi-level equilibration. This representation thereby best highlights the shifts in vacuum level and work function induced by ligand adsorption and facilitates a clear comparison of the resulting interfacial energy alignment across different ligands.

(3) While Fermi-level alignment is appropriate for describing fully equilibrated device junctions, the vacuum-level scheme is more suitable for emphasizing the relative energy-level shifts and band-offset modulation observed experimentally in UPS measurements. We also reviewed relevant literature and found that both vacuum energy level alignment and Fermi level alignment are well-suited for representing the energy level arrangement in perovskite solar cells. **Figure R53** illustrates two different approaches for depicting the perovskite energy levels. In **Figure R53a–e** (*Nature* 2024, 635, 860–866; *Nat. Energy* 2023, 8, 839–849; *Nature* 2024, 628, 93–98; DOI: 10.1038/s41560-025-01912-8; *Sci. Adv.* 2024, 10, eadq1150), the authors adopt a vacuum-level alignment scheme, which allows for a more straightforward comparison of changes in the conduction band minimum (CBM). In **Figure R53f, g** (*Nature* 2022, 611, 278–283; *Nat. Energy* 2025, 10, 513–525), both vacuum-level alignment and Fermi-level alignment are employed, the latter enables clear visualization of band bending.

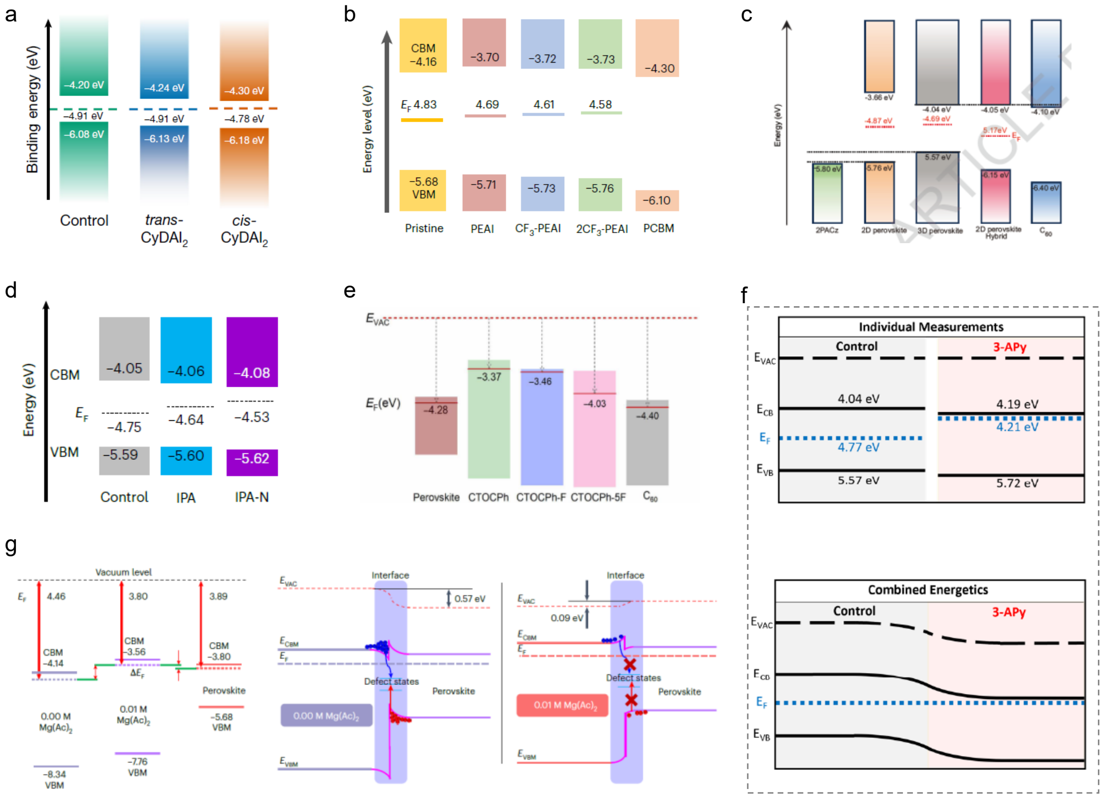

Figure R53. **(a)** Band alignment of the control, trans-CyDAI2-treated and cis-CyDAI2-treated perovskite films compared with C_{60}. The values are obtained from UPS measurements. (Copyright from *Nature* 2024, 635, 860–866). **(b)** Energy levels of valence band maximum (VBM) and conduction band minimum (CBM) derived from UPS spectra of the pristine and PEAI-, CF3-PEAI- and 2CF3-PEAI-treated perovskite films. (Copyright from *Nat. Energy* 2023, 8, 839–849). **(c)** the energetic alignment between 3D perovskite and charge-selective contacts. (Copyright from *Nature* 2024, 628, 93–98). **(d)** Schematic diagram of energy levels of conduction band minimum (CBM) and valence band maximum (VBM), along with E_F, derived from ultraviolet photoelectron spectra. (Copyright from https://doi.org/**10.1038/s41560-025-01912-8**). **(e)** Schematic representation of the band edge positions of the clusters and perovskite based on values from UPS results, referenced to the vacuum level. E_F and E_{VAC} represent Fermi and vacuum levels, respectively. (Copyright from *Sci. Adv.* 2024, 10, eadq1150). **(f)** Schematics of energy level diagram. Individual measurements of energy levels of perovskite films without (control) and with 3-APy treatment (3-APy), and the corresponding band-bending schematics. (Copyright from *Nature* 2022, 611, 278–283). **(g)** Schematic diagram of energy level alignment and carrier recombination at interfacial contact of ETL and perovskite with a CBO− (left) and CBO+ (right) structure. (Copyright from *Nat. Energy* 2025, 10, 513–525).

(4) In summary, both plotting approaches are commonly used in the field. In this work, we retain the vacuum-level alignment scheme, as it provides a more direct visualization of how the surface ligand treatment modulates the CBM of the perovskite. Altogether, we have added (**Nature 2024, 635, 860–866**) and (**Nat. Energy 2023, 8,**

839–849) to the bibliography as reference 22 and 37, and a brief methodological clarification and rationale have been added to the revised manuscript (see below).

"Concomitantly, the perovskite conduction band minimum (CBM), which was derived from the UPS-determined valence band maximum (VBM) and the optical bandgap (**Supplementary Figs. 24-26**, and **Supplementary Table 2**),[22,37] shifted downward from –4.11 eV (control) to –4.21 eV (TFPmA) (**Fig. 3e**), thereby narrowing the energy offset with C_{60} (–4.24 eV) from 0.13 to 0.03 eV." (**Page 10**, in the revised manuscript)

10. The figure caption text in Figure 4 needs revision. The corresponding figure caption text does not match with the actual individual panels in Figure 4.

Response: We have carefully rechecked and revised the **Fig. 4** caption to ensure that each description now accurately corresponds to the correct panel. The updated caption is now fully consistent with the figure layout and content.

"**Fig. 4| *In situ* passivation and carrier behavior.** (a) *In situ* photoluminescence (PL) spectroscopy evolution of the $FAPbI_3$-based perovskite surface during ligands and the consequent charge transport layer (CTL) spin-coating processes. (b) Normalized PL intensity extracted from *in situ* PL evolution maps. (c) The ratio of the PL intensity before (I_0) and after ligands modification ($I_{ligands}$). (d) The ratio of the PL intensity before (I'_{max}) and after CTL spin coating (I'_{min}). (e) Time-resolved photoluminescence (TRPL) curves of control and TFEA, TFBA, TFPA, TFPmA treated films with glass/perovskite/ligands structure. (f) TRPL curves of control and ligand-treated films with glass/perovskite/ligands/C_{60} structure." (**Page 14**, in the revised manuscript)

11. Please state explicitly the nominal perovskite composition at the beginning of the corresponding text employed for the fundamental characterizations (e.g., Figs. 2a, 3, and 4). Also, in Figure 5a and in the text, please state the nominal perovskite composition in "FTO/4PADCB/Perovskite/ligands/C60/BCP/Ag".

Response: In direct response to your valuable suggestion, we have updated the manuscript to explicitly detail the nominal perovskite compositions employed.

(1) The compositions used for the core characterizations and devices are:

- **Normal-bandgap $FAPbI_3$-based perovskite (1.56 eV):** $FA_{0.85}MA_{0.1}Cs_{0.05}Pb(I_{0.98}Br_{0.02})_3$
- **Wide-bandgap perovskite (1.68 eV):** $Cs_{0.22}FA_{0.78}Pb(I_{0.85}Br_{0.15})_3$

The corresponding text sections have been revised in the Supplementary Information and main text:

"Normal-bandgap $FAPbI_3$-based perovskite (1.56 eV) solution (1.5 M, $FA_{0.85}MA_{0.1}Cs_{0.05}Pb(I_{0.98}Br_{0.02})_3$) was prepared…

Wide-bandgap perovskite (1.68 eV) solution (1.3 M, $Cs_{0.22}FA_{0.78}Pb(I_{0.85}Br_{0.15})_3$) was prepared…" (1.2 Perovskite precursor solution, in the Supplementary Information).

(2) Regarding the fundamental characterizations shown in **Figs. 2a**, **3**, and **4**, all

measurements were performed using a normal-bandgap $FAPbI_3$-based perovskite composition ($FA_{0.85}MA_{0.1}Cs_{0.05}Pb(I_{0.98}Br_{0.02})_3$). To further clarify this point for readers, we have added an explicit description in the film characterization section of the Supplementary Information.

"Unless otherwise specified, all characterizations were performed on $FAPbI_3$-based perovskite ($FA_{0.85}MA_{0.1}Cs_{0.05}Pb(I_{0.98}Br_{0.02})_3$) films fabricated following our standard one-step spin-coating protocol." (1.5 Film characterization, in the Supplementary Information)

"The XPS spectra of calibrated C 1*s* and F 1*s* references for $FAPbI_3$-based perovskite with ligands are provided in **Supplementary Figs. 6** and **7**." (**Page 7**, in the revised manuscript)

"analyses of $FAPbI_3$-based perovskite films verified that …" (**Page 9**, in the revised manuscript)

"Following our device protocol, we spin-coated the ligand solution onto the $FAPbI_3$-based perovskite film …" (**Page 12**, in the revised manuscript)

(3) Regarding **Fig. 5a**, we believe that specifying a particular perovskite composition in the schematic is not appropriate. This is because, under the same device architecture, we subsequently investigate perovskite devices with different compositions and bandgaps. To avoid potential confusion and to maintain generality, we therefore keep the label as "perovskite" in the figure. For clarity and completeness, the exact chemical compositions of each device have been explicitly provided in the Supplementary Information, Section 1.2 Perovskite precursor solution.

"(FTO/4PADCB/Perovskite ($FAPbI_3$-based)/ligands/C_{60}/BCP/Ag, 0.074 cm^2 cell, **Fig. 5a**) achieving PCEs of …" (**Page 15**, in the revised manuscript)

12. Authors are suggested to provide a discussion on hysteresis in the main text (e.g., Figs 5b, 5d). If the authors can present the actual data (J-V of forward and reverse scans), it would be informative for readers (e.g., Fig. S32).

***Response*:** We appreciate valuable suggestion. In the revised manuscript, we have included *J*–*V* curves of forward and reverse scans a discussion of the hysteresis behavior in the main text, and the corresponding forward and reverse *J*–*V* curves and photovoltaic parameters are now presented in below.

(1) Our measurements show that all ligand-treated devices exhibit improved hysteresis (**Figure R54** and **Table R5**), indicating stabilized charge extraction and suppressed interfacial trap density. In particular, TFPmA-modified devices show a negligible hysteresis index (<2.1%), consistent with the efficient interfacial carrier management revealed by TRPL and DFT analyses. A brief discussion has been added to the main text.

"In addition, ligand-modified devices exhibited progressively reduced hysteresis indices (5.9%→2.1%) (Supplementary Fig. 38, and Supplementary Table 7)." (**Page 15**, in the revised manuscript)

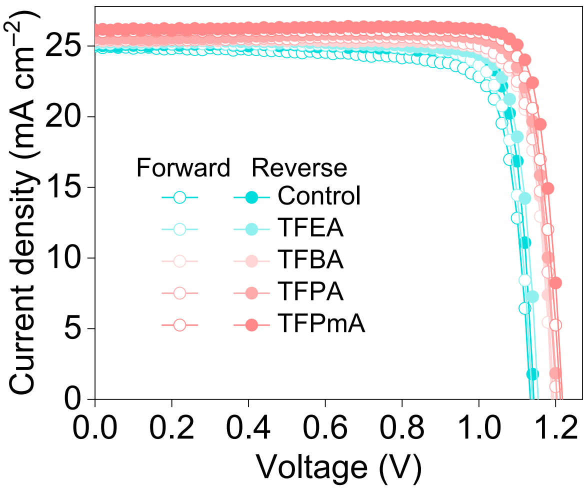

Figure R54. Forward and Reverse scan parameters of the control and four ligands treated solar cells. (**Supplementary Fig. 38**)

Table R5. Forward and Reverse scan parameters of the control and four ligands treated solar cells. (added **Supplementary Table 7**)

	Sample	V_{OC} (V)	J_{SC} (mA·cm^{-2})	FF (%)	PCE (%)	HI (%)
Control	Forward	1.134	24.98	80.58	22.83	5.9
	Reverse	1.143	25.07	84.66	24.26	
TFEA	Forward	1.139	25.18	82.43	23.65	4.1
	Reverse	1.154	25.27	84.52	24.65	
TFBA	Forward	1.190	25.48	83.24	25.22	2.9
	Reverse	1.195	25.41	85.58	25.98	
TFPA	Forward	1.202	25.43	83.80	25.61	2.9
	Reverse	1.203	25.55	85.76	26.37	
TFPmA	Forward	1.212	26.07	85.44	27.01	2.1
	Reverse	1.218	26.13	86.69	27.58	

(2) As for wide-bandgap (1.68 eV) $FAPbI_3$-based devices, we have updated the device efficiency, achieving a champion efficiency of **24.04%** through TFPmA modification. Additionally, we have supplemented the *J-V* curves for **both forward and reverse scans,** as shown in **Figure R55**. Compared to the control, the hysteresis

factor of the TFPmA-modified device decreased from 2.0% to 0.5% (**Table R6**).

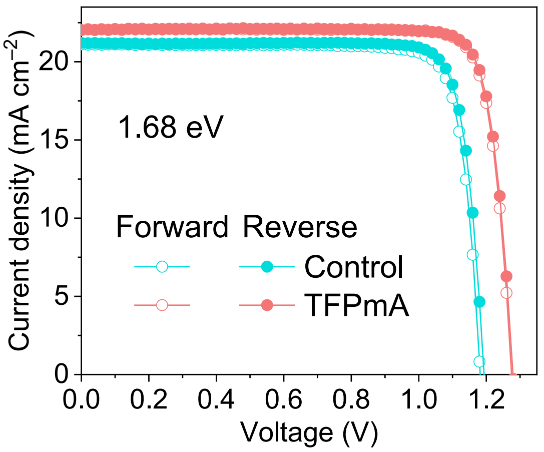

Figure R55. *J-V* curves of wide-bandgap (1.68 eV) devices for control and TFPmA treated devices. (Updated **Fig. 5d**, in the revised manuscript)

Table R6. Forward and Reverse scan parameters of the wide bandgap (1.68 eV) solar cells without and with TFPmA. (Added **Supplementary Table 9**, in the Supplementary Information)

	Sample	V_{OC} (V)	J_{SC} (mA·cm^{-2})	FF (%)	PCE (%)	HI (%)
Control	Forward	1.182	21.11	83.92	20.94	2.0
	Reverse	1.193	21.20	84.44	21.37	
TFPmA	Forward	1.275	22.02	85.13	23.91	0.5
	Reverse	1.279	22.03	85.30	24.04	

Correspondingly, we have also added **Figure R55** (Updated **Fig. 5d**) and **Table R6** (**Supplementary Table 9**) to the revised manuscript as follows:

"The strategy is readily applicable to wide-bandgap perovskite systems (1.68 eV), yielding 24.04% PCE (certified at 23.04%, **Fig. 5d**, **Supplementary Figs. 45** and **46**, and **Supplementary Table 9**)." (**Page 15**, in the revised manuscript)

(3) Inspired by this comment, we have carefully reconsidered the "Visualizing performance loss analysis" that is based on the peak-performance data point. Even though this has been widely adopted in the literatures, we realized that if intrinsic hysteresis is not properly accounted for—even with a hysteresis index as low as 2.1%—such a detailed SQ-limit-based loss analysis may deviate from the actual performance losses in operating devices.

Therefore, to ensure greater rigor in data presentation and to facilitate accurate interpretation by readers, we have decided to remove the corresponding dataset (original **Fig. 5e, f** and Supplementary **Figs. 36–39**) from the manuscript and have revised the title to "Stereoelectronic Manipulation of Ligands Toward Efficient

Perovskite Solar Cells." The updated **Fig. 5** is provided below for clarity.

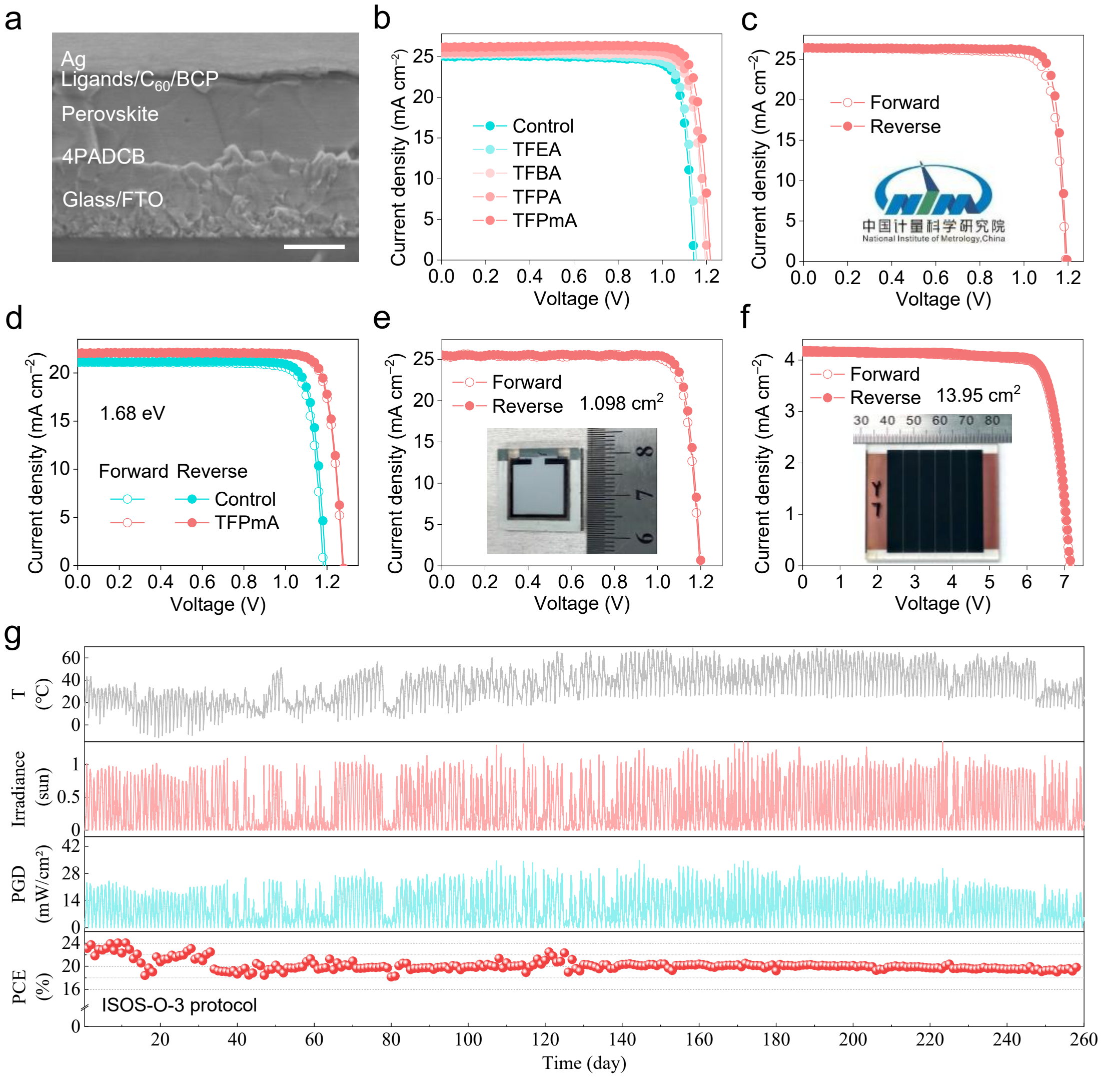

Figure R56 De vice performance and stability. (a) Cross-sectional structural image of perovskite solar cell (the scale bar is 500 nm). (b) *J-V* curves of the control and ligands treated devices. (c) Certified photovoltaic performance of devices obtained at NIM. (d) J-V curves of wide-bandgap (1.68 eV) devices for control and TFPmA treated devices. (e) *J-V* curves of 1.098 cm^2 devices for TFPmA-treated solar cells. (f) *J-V* curves of the TFPmA-treated module devices. (g) The real-time field testing of TFPmA-treated module under actual out-door operating conditions for 258 days. (**Updated Fig. 5**, in the revised manuscript)

13. Splitting Figure S33 into two pages are recommended so readers can access the information in its contents.

Response: Thank you for the helpful suggestion. In the revised Supplementary Information, the original **Supplementary Fig. 33** has been divided into two separate figures (now **Supplementary Figs. 45** and **46**) to improve readability and ensure that all details and annotations are clearly visible.

14. Citation of Supplementary Notes 1 to 3 in proper locations in the main text would help readers to access directly the complementary information provided by the authors.

***Response*:** In the revised manuscript, we have added explicit cross-references to **Supplementary Notes 1–3** in the main text at appropriate locations to guide readers to the corresponding complementary discussions and analyses. Specifically, **Supplementary Note 1** (Theoretical calculations) is now cited in the section describing the stereoelectronic manipulation of ligands in **Figure 1**. **Supplementary Note 2** (XPS, NMR and FTIR measurement and analysis) is now cited in the computational modeling section of **Figure 2**. **Supplementary Note 3** (Nano IR measurement and analysis) is now cited in the discussion of **Figure 3c, d**. These citations improve the accessibility and traceability of supplementary materials, as recommended.

"adsorption topology on the Pb-I framework of perovskites (See details in **Supplementary Note 1**)." (**Page 4**, in the revised manuscript)

"(see analysis details in **Supplementary Note 2**)." (**Page 7**, in the revised manuscript)

"Hyperspectral nano-infrared imaging (nano-IR; <10 nm resolution, **Fig. 3c**, and **Supplementary Note 3**)" (**Page 9**, in the revised manuscript)

15. Discussion of "PGD (mW/cm^2)" graph shown in Figure 5i is needed.

***Response*:** We thank the reviewer for pointing this out. In the revised manuscript, we have defined the full name of PGD and specified the corresponding range of measured values to improve clarity and completeness.

"Subjected to 258 days of continuous operation with temperature cycling (–11.5 to 68.9 °C) and irradiance fluctuations (0–1.39 sun; plane of array global irradiance (PGD) of 0–34.7 mW cm^{-2})" (**Page 16**, in the revised manuscript)

Referee #3 (Remarks to the Author):

A. Summary of Key Results

The manuscript "Stereoelectronic Manipulation of Ligands Toward Shockley-Queisser Limit of Perovskite Solar Cells" by Yang and coworkers presents a systematic study on the stereoelectronic design of passivation molecules for the perovskite/ETL interface in p-i-n single junction solar cells. Using ESP and DFT for theoretical calculations, to spectroscopic analysis for passivant-perovskite precursor interactions, to various thin film characterization and loss analysis, the paper ends in presenting a highly efficient single-junction perovskite solar cell with record fill factor on small device area for the target passivation molecule. The approach is tested also for a higher bandgap perovskite absorber suitable for tandem application and integrated in a minimodule. Improved stability data for the target compared to the reference is presented, too.

The molecular design study presents the combination of an amido-moiety for defect passivation, an -CF3 hydrophobic moiety for improved environmental stability with a systematic variation of a central unit ranging from benzene to N-heteroaromats pyridine and pyrimidine. Their main finding is that the introduction of N-heteroaromats yields instead of a vertical a planar interaction with the absorber surface allowing for improved chemical passivation of surface defects but further improves charge transport.

With their work the authors contribute to the field of emerging PV technologies presenting valuable insights into the molecular designs of interfacial passivants as well as a (in-house) record for perovskite solar cells with 27.58% (certified 26.98%). This is on pair with the current certified record of 27% published in the efficiency tables and the NREL chart. I would like to congratulate the authors for this achievement.

Overall, the manuscript is well-written and the quality of the manuscript is high. However, certain areas of the manuscript require further clarification, particularly with the framing of the paradox of molecular design, the novelty of the N-heteroaromatic inclusion, as well as details on the perovskite bulk material and its surface termination. Further some points on language and wording are recommended to reconsider.

I recommend major revisions and think that addressing the points outlined below can help to strengthen the manuscript further to be considered for publication in Nature.

B. Originality and Significance

The authors address a hot topic in the perovskite field with regard to high-efficient perovskite solar cells. The systematic study allows to give more understanding on design rules for interfacial passivation at the perovskite/C60 interface.

However, the authors should make clear which existing literature on planar aligning passivation molecules and which on N-heterocycle containing ones have been reported already in literature. Please make the originality and novelty of the paper more clear.

Response: We sincerely thank the reviewer for this insightful comment, which has helped us better articulate the conceptual advance of our work. We fully agree on the importance of clarifying the originality and novelty of our study relative to existing literature on planar-aligned and N-heteroaromatic passivation molecules.

The prevailing molecular design strategies for perovskite interfaces have largely relied on two established pillars: (1) vertical alignment for strong ionic bonding, and (2) the use of amine as terminal passivation groups. The present work fundamentally transcends both approaches by introducing a third dimension: the active control of adsorption topology through the electronic structure of the molecular core itself, thereby resolving the fundamental passivation-transport trade-off.

Below, we delineate the key conceptual advances:

(1) **A New Design Rule: Replacing Size-Matching with Electronic Driving Force**

Two publications demonstrated that planar molecules may improve performance based on 4ABS (Sargent et al., *Science* 2024, 384, 189–193) or CO-BSA (Peng et al., *Adv. Mater.* 2025, 2507918). However, their design logic was based on **static geometric compatibility**—finding a molecule whose pre-defined length fits the perovskite lattice.

We have established a more dynamic and predictive design rule. By progressively incorporating nitrogen atoms into the conjugated core, we actively inject a thermodynamic driving force that flips the molecular orientation from vertical to planar. The DFT calculations (**Fig. 1c**) provide evidence of this engineered switch, showing a reversal in adsorption energy preference (ΔE). Importantly we confirmed the **underlying passivation mechanism for the planar adsorption is multi-center interaction (Pb-N coordination *and* Pb-I-π), rather than size-matching**. Consequently, we have also advanced the field from *discovering* two molecules that happen to lie flat to *designing* molecules that are compelled to do so.

(2) **Redefining the Role of the N-Heterocycle: From Passive Site to Active Dictator**

The conventional role of N-heterocycles, as seen in works like 3APy (Zhu et al., *Nature* 2022, 611, 278–283), is a **passive defect-passivating functional group**, leaving room for the optimization of the charge transport distance.

We fundamentally repurpose the N-heterocycle. In our design, it is not a terminal group but **an integral component of the molecular backbone that acts as a stereoelectronic switch**. This critical repositioning unlocks **synergistic multicenter interactions (Pb-N coordination *and* Pb-I-π)**, which are the *direct cause* of both the planar alignment and the superior passivation. This transforms the N-heterocycle from a mere "defect healer" into an "**interface architect**".

(3) **The Synergistic Outcome: Decoupling the Fundamental Trade-off**

The aforementioned advances culminate in a decisive resolution of the passivation-transport paradox. Our "stereoelectronic manipulation" creates a unique interfacial architecture where the **same molecular feature** that enhances binding energy for passivation **simultaneously minimizes charge transport distance by ~37% (Figure 1e**).

(4) In short, while previous three Nature/Science publications identified *three* molecules could work as passivants for solar cells, the state-of-the-art strategies focus on vertical ligands and above geometric size-matching passivants. It remains unclear how to synergistically manage interfacial energy losses through combined defect passivation and transport-geometry optimization to approach the SQ limit.

Our work **provide** the precise mechanism behind effective passivation. This establishes **"stereoelectronic manipulation" as a guiding principle for future**

interfacial design, charting a path toward the SQ limit.

Per your suggestion, we have expressed this perspective by adding the following into the revised manuscript.

"Yet, even state-of-the-art remedies including those employing vertical ligands or geometric size-matching passivants,[13,24-26] leave this fundamental dilemma unresolved." (**Page 3**, in the revised manuscript)

C. Data and Methodology

The experimental design is generally sound and the broad methodology applied in the paper is definitely a strength.

Figures are clear and informative, though Figure 2d could benefit from improved description (colored areas, numbering), as well as full axis labeling in case of Figure 3c, d. Further the inset graph in Figure 4b is too squeezed. In Figure 5 f q Voc instead of Voc Energy as axis label.

Response: Thanks for the careful examination and constructive suggestions regarding figure clarity and labeling.

(1) As for **Fig. 2d (updated as Fig. 2e)**, the colored areas and numbered regions have been explicitly described in the revised caption and annotated within the figure for better readability (**Figure R57**). Below is the revised main text.

"pyridine N-Pb interaction softened ring breathing (1,571/1,526 cm^{-1}), while pyrimidine distortion split the symmetric stretch into a 1,550/1,502/1,452 cm^{-1} triple (**Fig. 2e,** shaded area lines 1-3), which demonstrate **direct engagement** of N-heteroaromatic motifs **in** Pb–I–π **interaction**." (**Page 8**, in the revised manuscript)

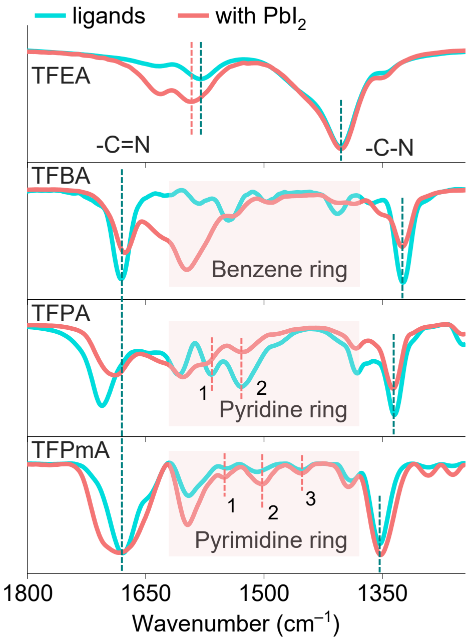

Figure R57. FTIR spectra of different ligands interacting with PbI_2. (**Updated Fig. 2e,** in the revised manuscript).

(2) Regarding the **Fig. 3c (updated Fig. 3d)** and **3d (updated Fig. 3c)**, the full axis labels (including units) have been added, and font sizes were adjusted for consistency with the other panels (**Figure R58**).

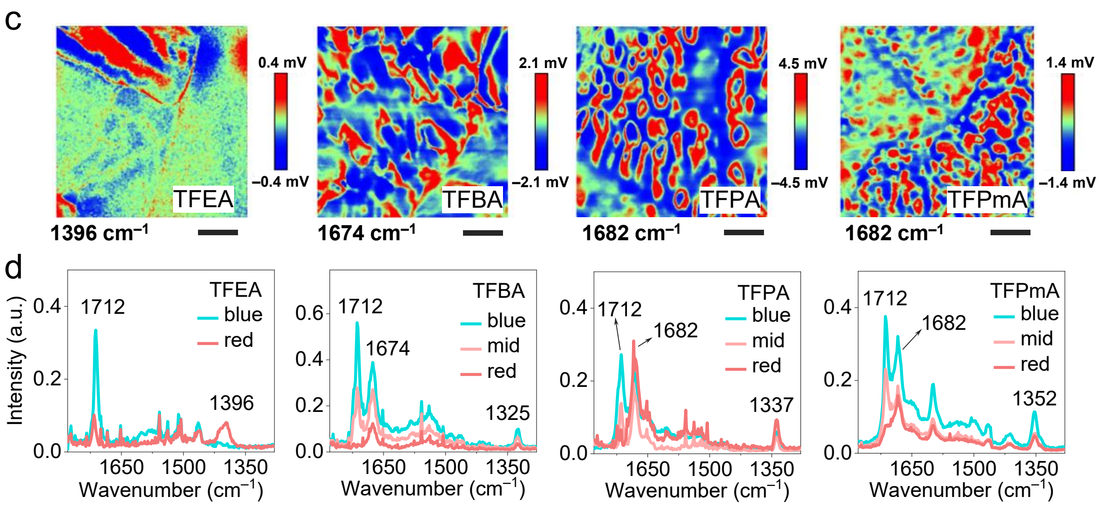

Figure R58. (c) Nano-IR chemical mapping of films modified with TFEA, TFBA, TFPA, and TFPmA (scale bar: 60 nm), showing spatial distribution of key IR absorptions. (d) Nano-IR line-plot spectra acquired from the perovskite surface mappings (**Supplementary Fig. 21**, the blue, mid, and red lines corresponding to the blue, intermediate, and red mapping, respectively), highlighting vibrational signatures of each ligand. (Updated **Fig. 3c** and **3d,** in the revised manuscript).

(3) Regarding the **Fig. 4b**, the inset plot has been resized and repositioned to avoid visual crowding, ensuring that all data points and axes are clearly visible (**Figure R59**).

(4) Regarding the **Fig. 5f (updated Supplementary Fig. 48)**, the y-axis label has been corrected from "Voc Energy" to "q V_{OC} (eV)" as suggested (**Figure R60**). However, inspired by **Reviewer 2's** comment #12, we have carefully reconsidered the "Visualizing performance loss analysis" that is based on the peak-performance data point. Even though this has been widely adopted in the literatures (e.g., *Nat. Photon*. 2019, 13, 501–505; *Nat. Mater*. 2024, 23, 391–397; *Joule* **2022**, 6, 756-771; *Adv. Mater.* **2022**, 34, 2108132), we realized that if intrinsic hysteresis is not properly accounted for—even with a hysteresis index as low as 2.1%—such a detailed SQ-limit-based loss analysis may deviate from the actual performance losses in operating devices.

Therefore, to ensure greater rigor in data presentation and to facilitate accurate interpretation by readers, we have decided to remove the corresponding dataset (original **Fig. 5e, f** and Supplementary **Figs. 36–39**) from the manuscript and have revised the title to "Stereoelectronic Manipulation of Ligands Toward Efficient Perovskite Solar Cells."

(5) We truly appreciate the reviewer for helping us refine the figures and textual details. The revisions have indeed made the presentation clearer and more informative, and the updated figures and text have now been incorporated into the main manuscript.

We respectfully invite the reviewer to review the revised content.

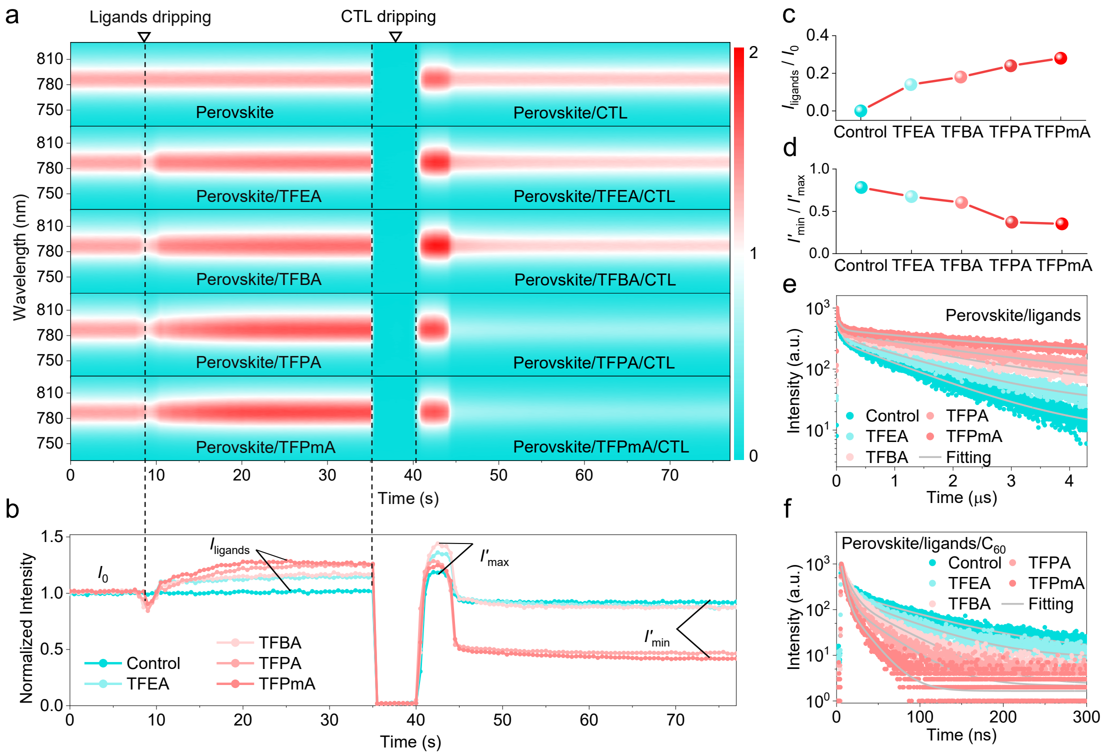

Figure R59. *In situ* passivation and carrier behavior. (a) *In situ* photoluminescence (PL) spectroscopy evolution of the $FAPbI_3$-based perovskite surface during ligands and the consequent charge transport layer (CTL) spin-coating processes. (b) Normalized PL intensity extracted from *in situ* PL evolution maps. (c) The ratio of the PL intensity before (I_0) and after ligands modification ($I_{ligands}$). (d) The ratio of the PL intensity before (I'_{max}) and after CTL spin coating (I'_{min}). (e) Time-resolved photoluminescence (TRPL) curves of control and TFEA, TFBA, TFPA, TFPmA treated films with glass/perovskite/ligands structure. (f) TRPL curves of control and ligand-treated films with glass/perovskite/ligands/C_{60} structure. (**Updated Fig. 4**, in the revised manuscript)

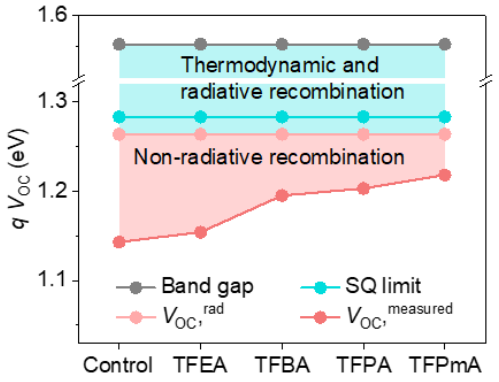

Figure R60. The V_{OC} loss analysis of the devices.

D. Use of Statistics and Treatment of Uncertainties

With respect to the little amount of statistics shown in the perovskite field in general,

authors consider an appropriate level of data sets and various methods to backup trends/derive findings.

However, authors are encouraged to talk more about errors and reconsider if it is reasonable to state current density values in mA/cm2 with two digits after the comma, similarly the FF.

***Response*:** Thanks for thoughtful consideration of statistics and uncertainties. Per your suggestions, we have implemented the corresponding revisions as detailed below:

(1) To validate the repeatability of the devices and assess data errors, we explicitly present statistical analysis of 20 devices in the main text (**Page 15**, in the revised manuscript), with statistical averages and error values provided in **Supplementary Fig. 46** and **Supplementary Table 7**.

(2) Regarding current density, we find that retaining two decimal places in the unit mA cm^{-2} is accepted practice. For instance, perovskite solar cell parameters listed in authoritative tables from NREL and Martin Green also employ current density values with two decimal places, as seen at URL https://www.nrel.gov/pv/cell-efficiency and https://www.researchgate.net/publication/392104283_Solar_Cell_Efficiency_Tables_Version_66. Additionally, we reviewed previously published literature in Nature (*Nature* 2023, 616, 724–730; *Nature* 2023, 624, 289–294; *Nature* 2024, 632, 536–542; *Nature* 2024, 635, 82–88; *Nature* 2025, 642, 78–84; *Nature* 2025, 646, 95–101), which similarly retained two decimal places. Similarly, in these authoritative tables and literature, retaining FF values to two decimal places remains reasonable. So, we follow the tradition in the field to adopt the accepted practice following NREL and Martin Green tables and other Nature publications.

The *J*–*V* characteristics were measured using a high-resolution Keithley 2450 SourceMeter®, which provides voltage and current resolutions of 1 μV and 10 nA, respectively, with measurement accuracies of ±0.012% (voltage) and ±0.020% (current) at 23 ± 5 °C., as shown in **Figure R61**. In this work, the raw *J*–*V* data were recorded with six decimal places. Accordingly, to reflect the instrumental resolution and measurement accuracy, we report the photovoltaic parameters using four significant figures in the manuscript, with current density and fill factor presented to two decimal places.

2450 SourceMeter® SMU Instrument

Datasheet

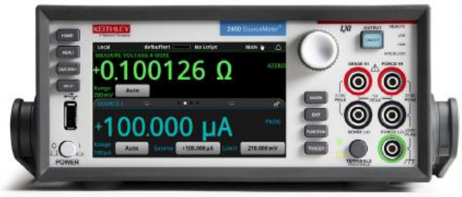

KEITHLEY
A Tektronix Company

The 2450 is Keithley's next-generation SourceMeter source measure unit (SMU) instrument that truly brings Ohm's law (current, voltage, and resistance) testing right to your fingertips. Its innovative graphical user interface (GUI) and advanced, capacitive touchscreen technology allow intuitive usage and minimize the learning curve to enable engineers and scientists to learn faster, work smarter, and invent easier. The 2450 is the SMU for everyone: a versatile instrument, particularly well-suited for characterizing modern scaled semiconductors, nano-scale devices and materials, organic semiconductors, printed electronics, and other small-geometry and low-power devices. All this combined with Keithley SMU precision and accuracy allows users to Touch, Test, Invent® with the new favorite go-to instrument in the lab for years to come.

Key Features

- Capabilities of analyzers, curve tracers, and I-V systems at a fraction of their cost
- Five-inch, high resolution capacitive touchscreen GUI
- 0.012% basic measure accuracy with 6½-digit resolution
- Enhanced sensitivity with new 20mV and 10nA source/measure ranges
- Source and sink (four-quadrant) operation
- Four "Quickset" modes for fast setup and measurements
- Built-in, context-sensitive front panel help
- Front panel input banana jacks; rear panel input triaxial connections
- 2450 SCPI and TSP® scripting programming modes
- 2400 SCPI-compatible programming mode
- Front panel USB memory port for storing data, programming, instrument configurations, and to upgrade the unit

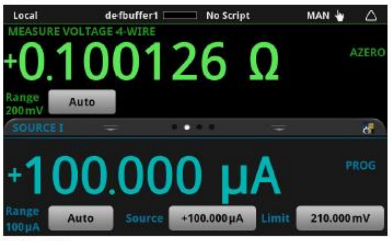

2450 main home screen.

Voltage Specifications [1, 2]

	Source			Measure [3]		
Range	Resolution	Accuracy (23° ± 5°C) 1 Year ±(% setting + volts)	Noise (RMS) (<10 Hz)	Resolution	Input Resistance	Accuracy (23° ± 5°C) 1 Year ±(% rdg. + volts)
20.00000 mV	500 nV	0.100% + 200 µV	1 µV	10 nV	>10 GΩ	0.100% + 150 µV
200.0000 mV	5 µV	0.015% + 200 µV	1 µV	100 nV	>10 GΩ	0.012% + 200 µV
2.000000 V	50 µV	0.020% + 300 µV	10 µV	1 µV	>10 GΩ	0.012% + 300 µV
20.00000 V	500 µV	0.015% + 2.4 mV	100 µV	10 µV	>10 GΩ	0.015% + 1 mV
200.0000 V	5 mV	0.015% + 24 mV	1 mV	100 µV	>10 GΩ	0.015% + 10 mV

Current Specifications [1, 2]

	Source			Measure [3]		
Range	Resolution	Accuracy (23° ±5°C) [4] 1 Year ±(% setting + amps)	Noise (RMS) (<10 Hz)	Resolution	Voltage Burden	Accuracy (23° ±5°C) 1 Year ±(% rdg. + amps)
10.00000 nA [5]	500 fA	0.100% + 100 pA	500 fA	10 fA	<100 µV	0.100% + 50 pA
100.0000 nA [5]	5 pA	0.060% + 150 pA	500 fA	100 fA	<100 µV	0.060% + 100 pA
1.000000 µA	50 pA	0.025% + 400 pA	5 pA	1 pA	<100 µV	0.025% + 300 pA
10.00000 µA	500 pA	0.025% + 1.5 nA	40 pA	10 pA	<100 µV	0.025% + 700 pA
100.0000 µA	5 nA	0.020% + 15 nA	400 pA	100 pA	<100 µV	0.020% + 6 nA
1.000000 mA	50 nA	0.020% + 150 nA	5 nA	1 nA	<100 µV	0.020% + 60 nA
10.00000 mA	500 nA	0.020% + 1.5 µA	40 nA	10 nA	<100 µV	0.020% + 600 nA
100.0000 mA	5 µA	0.025% + 15 µA	100 nA	100 nA	<100 µV	0.025% + 6 µA
1.000000 A	50 µA	0.067% + 900 µA	3 µA	1 µA	<100 µV	0.030% + 500 µA

Temperature Coefficient (0°–18°C and 28°–50°C) ±(0.15 × accuracy specification)/°C.

Figure R61. Instrument description and voltage/current measurement accuracy specifications of the Keithley 2450 SourceMeter®.

For outdoor measurements it would be helpful that also the control group is shown as comparison.

***Response*:** Thanks for valuable suggestion. In the revised manuscript:

(1) we have included the performance evolution of the control group under outdoor

measurements. The corresponding data and plots have been added in **Supplementary Fig. 58** in the Supplementary Information (**Figure R62**). After 27 days of outdoor monitoring, the control device exhibits a 24.6% reduction in module efficiency, decreasing from an initial value of 21.03% to 15.85%. This comparison clearly highlights the improved interfacial stability of the modified devices relative to the control, further supporting our conclusions regarding the effectiveness of the stereoelectronic passivation strategy.

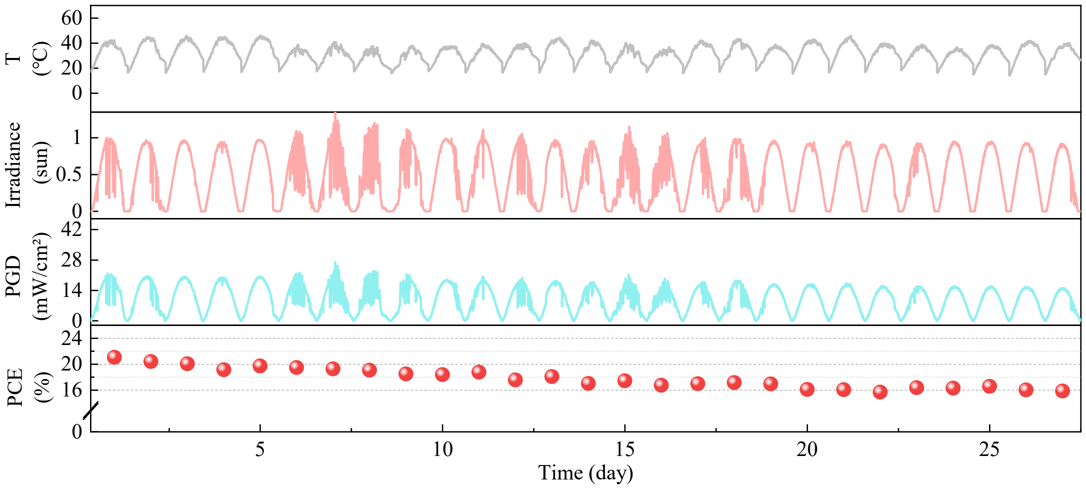

Figure R62. The real-time field testing of control group under actual out-door operating conditions for 27 days. (**Supplementary Fig. 58**)

(2) In addition, we continued the outdoor operational monitoring of the optimized devices, extending the testing duration to **258** days. The device efficiency decreased to 19.82%, corresponding to a 14.2% loss relative to the initial efficiency of 23.10%. To our knowledge, this represents **the first >23%-efficiency perovskite module to demonstrate such prolonged operational stability**, as shown in **Figure R63**.

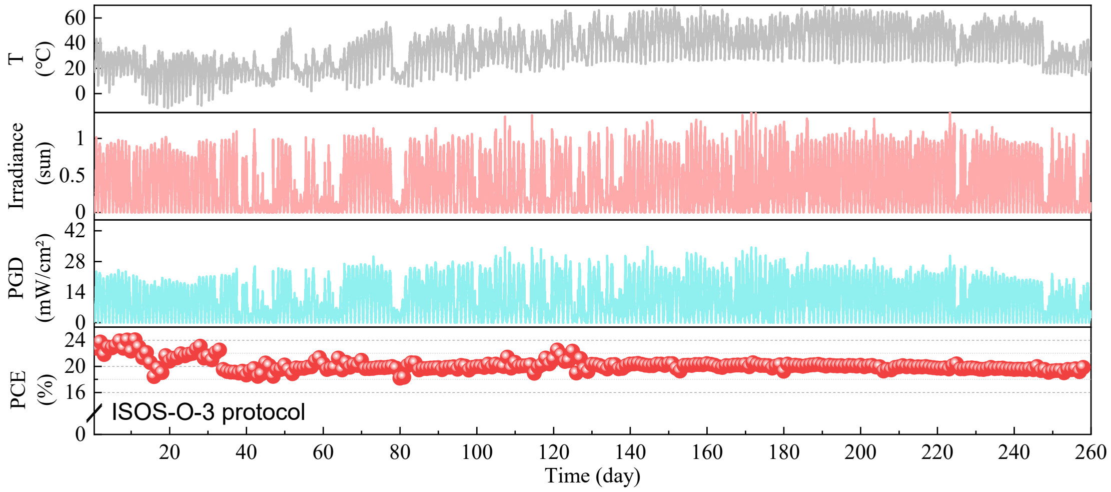

Figure R63. The real-time field testing of TFPmA-treated module under actual out-door operating conditions for 258 days. (**Updated Fig. 5g**, in the revised manuscript)

(3) These outdoor, real-world operational data further substantiate the significant enhancement in long-term device stability enabled by the optimized ligand treatment. Accordingly, **Figure R63** has been incorporated into the main text as **Fig. 5g**, and the relevant sections of the manuscript have been updated to reflect these results, as detailed below:

"(**Fig. 5g, Supplementary Figs. 51** and **52**). Subjected to 258 days of continuous operation with temperature cycling (–11.5 to 68.9 °C) and irradiance fluctuations (0–1.39 sun; plane of array global irradiance: 0–34.7 mW cm^{-2}), the TFPmA-treated module exhibited only a 14.2% loss (from 23.10% to 19.82% PCEs). In sharp contrast, the control module degraded by 24.6% (from 21.03% to 15.85% PCEs) after merely 27 days of outdoor testing (**Supplementary Fig. 53**)." (**Page 16**, in the revised manuscript)

(4) In addition, we further optimized the device encapsulation process. For standard small-area cells, the **certified PCE was increased to 27.41%**, which is the **highest values reported to date** (**Figure R64**). Upon scaling up, the efficiency of 1 cm^2–class device was improved to **26.40%**, with a V_{OC} of 1.202 V, a J_{SC} of 25.54 mA cm^{-2}, and an FF of 86.04% (**Figure R65**), yielding a certified efficiency of 25.61% (**Figures R66** and **R67**). Furthermore, the efficiency of the mini-module (13.95 cm^2) was increased to **24.74%**, also representing one of the highest values reported to date, with device parameters of V_{OC} = 7.16 V, J_{SC} = 4.18 mA cm^{-2}, and an FF of 82.73% (**Figure R68**).

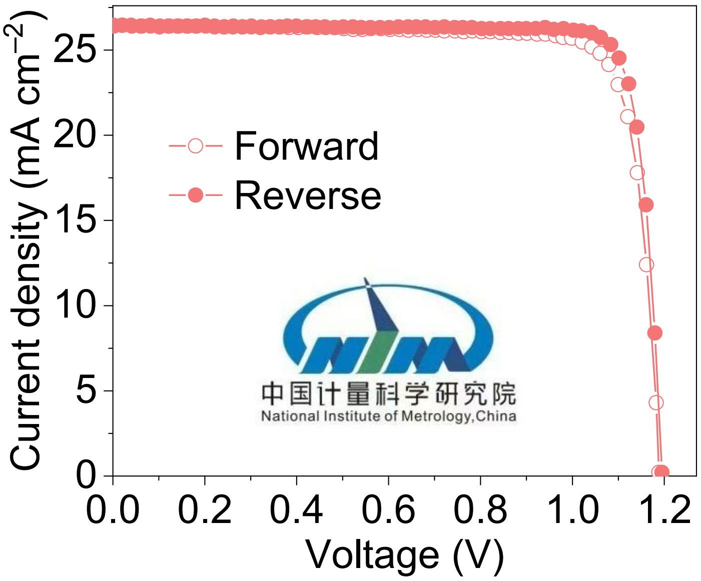

Figure R64. Certified photovoltaic performance of devices obtained at NIM. (**Updated Fig. 5c**)

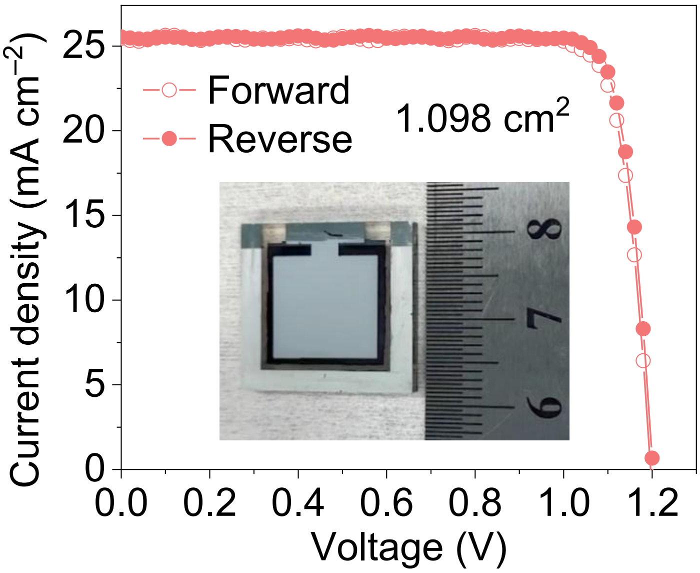

Figure R65. The *J-V* curves of 1.098 cm^2 devices for TFPmA-treated solar cells. (**Fig. 5e**, in the revised manuscript)

中国计量科学研究院
National Institute of Metrology,China

Appendix: Summary of the Report
Report No.: GXgf2025-03921
Client: Shaanxi Normal University
Sample: Perovskite Solar Cell
Type/Model: Perovskite Solar Cell
DUT S/N: Y4-M0821
Manufacturer: Shaanxi Normal University
Sample temperature: (25±1)°C
Date of Test: 11/28/2025
Environmental conditions: (22 ± 1)°C RH (17± 2)%
Mask: An aperture area of 109.896 mm² (M0821, Certificate No.: CDjc2025-05684)

The test has been conducted by the PV Metrology Lab of NIM (National Institute of Metrology, China). Measurement of irradiance intensity and all other measurements are traceable to the International System of Units (SI). The data in this report is only applicable to the samples tested at that time. For more details, please refer to the text of the report.

	Area (mm^2)	I_{sc}(mA)	V_{oc}(V)	P_{max}(mW)
Reverse Scan	109.896	27.870	1.195	28.150
Forward Scan	109.896	27.920	1.193	27.250
	I_{max}(mA)	V_{max}(V)	FF (%)	η (%)
Reverse Scan	26.560	1.060	84.52	25.62
Forward Scan	26.200	1.040	81.83	24.80

I-V Characterization Methods:
JJF 1622-2017: Calibration Specification of Solar Cells: Photoelectric Properties
JJF 2129-2024: Calibration Specification for Perovskite Solar Cells: Photoelectric Characteristic Parameters

Reference Solar Cell:
Type: Mono-Si

Solar Simulator:
Classification: A+AA+ (Double-light source: Xeon and Halogen);
Total irradiance: 1000 W/m² based on I_{sc} of the above Reference Solar Cell.

Figure R66. The Certificated results of the 1 cm^2-class devices from the National Institute of Metrology, China (NIM, China). The forward scan (reverse scan) was performed from −0.10 V to 1.28 V (1.28 V to −0.1 V). (**Supplementary Fig. 49**)

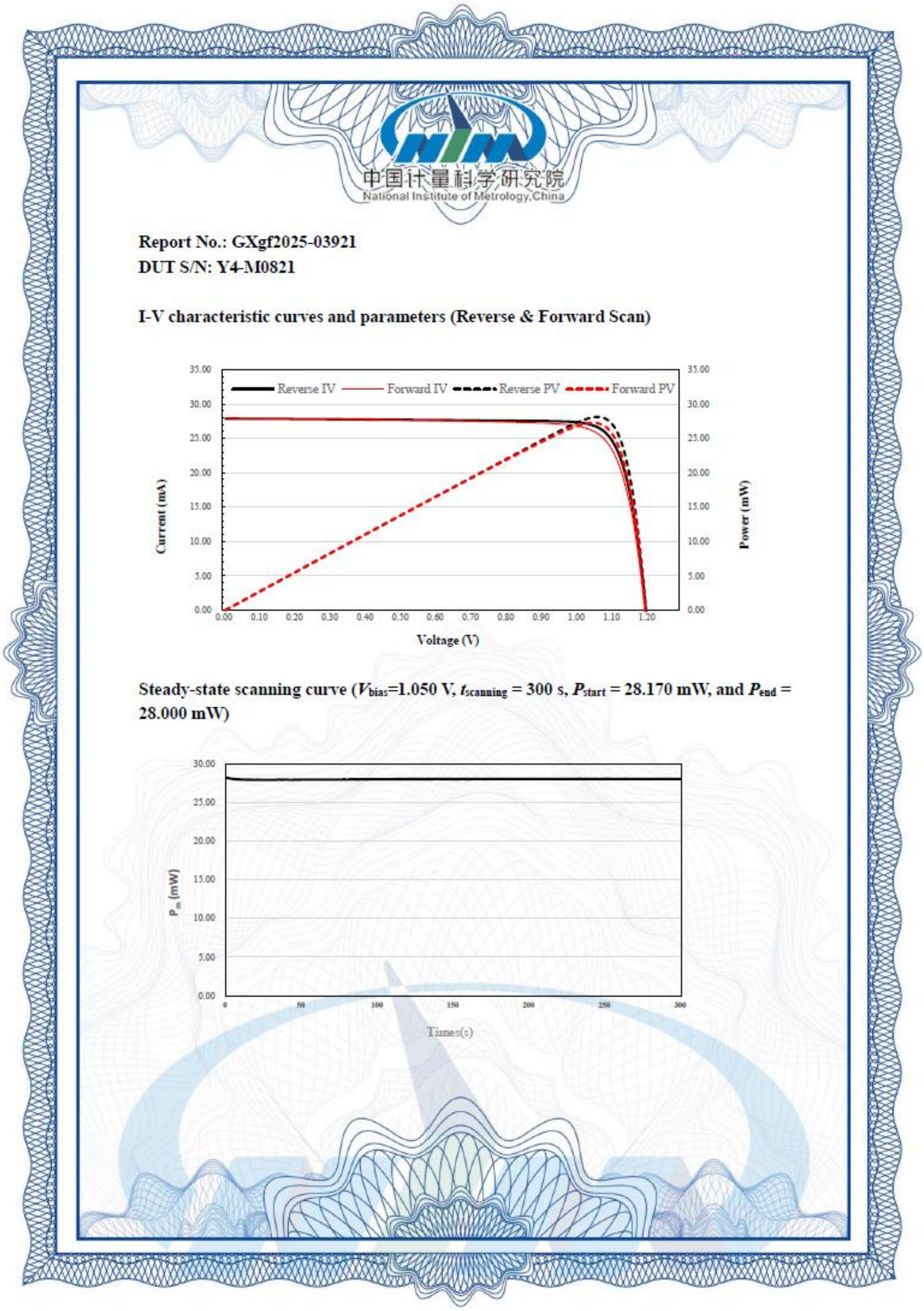
中国计量科学研究院
National Institute of Metrology,China

Report No.: GXgf2025-03921
DUT S/N: Y4-M0821

I-V characteristic curves and parameters (Reverse & Forward Scan)

Steady-state scanning curve (V_{bias}=1.050 V, $t_{scanning}$ = 300 s, P_{start} = 28.170 mW, and P_{end} = 28.000 mW)

Figure R67. The Certificated results of the 1 cm^2-class devices from the National Institute of Metrology, China (NIM, China). The steady-state scanning was performed for 300 s with one read per second under 1.05 V. (**Supplementary Fig. 50**)

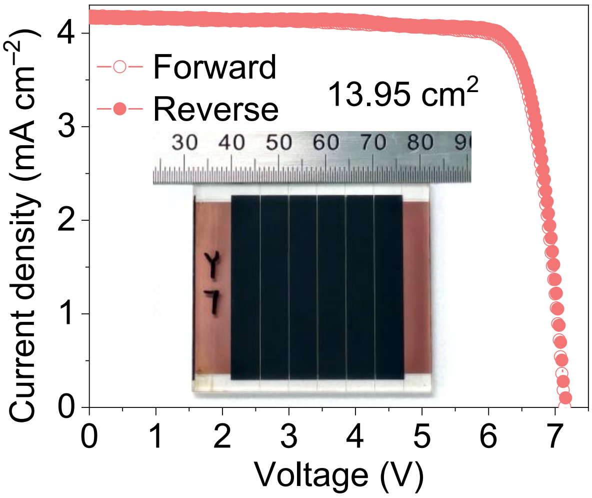

Figure R68. The *J-V* curves of the TFPmA-treated module devices. (**Fig. 5f**, in the revised manuscript)

(5) We have updated the manuscript to include the latest performance metrics for all devices, along with the corresponding revisions in the relevant sections of the text, as detailed below:

"We sent the best-performing device to an accredited PV laboratory (the PV Metrology Lab of National Institute of Metrology, China) for third-party certification and obtained an efficiency of 27.41% a with stabilized power output efficiency of 26.85% (**Fig. 5c**, **Supplementary Figs. 39-42**)." (**Page 15**, in the revised manuscript)

"In 1.098 cm^2 single-junction cells, we achieved a champion PCE of 26.40% (certified at 25.62%, **Fig. 5e**, **Supplementary Figs. 49** and **50**) with V_{OC} = 1.202 V, J_{SC} = 25.54 mA cm^{-2}, FF = 86.04%) (reverse scan)." (**Page 15**, in the revised manuscript)

"Extending the active area to 13.95 cm^2, modules achieved a champion PCE of 24.74% (V_{OC} = 7.160 V, J_{SC} = 4.18 mA cm^{-2}, FF = 82.73% (reverse scan), **Fig. 5f, Supplementary Table 10**)." (**Page 15-16**, in the revised manuscript)

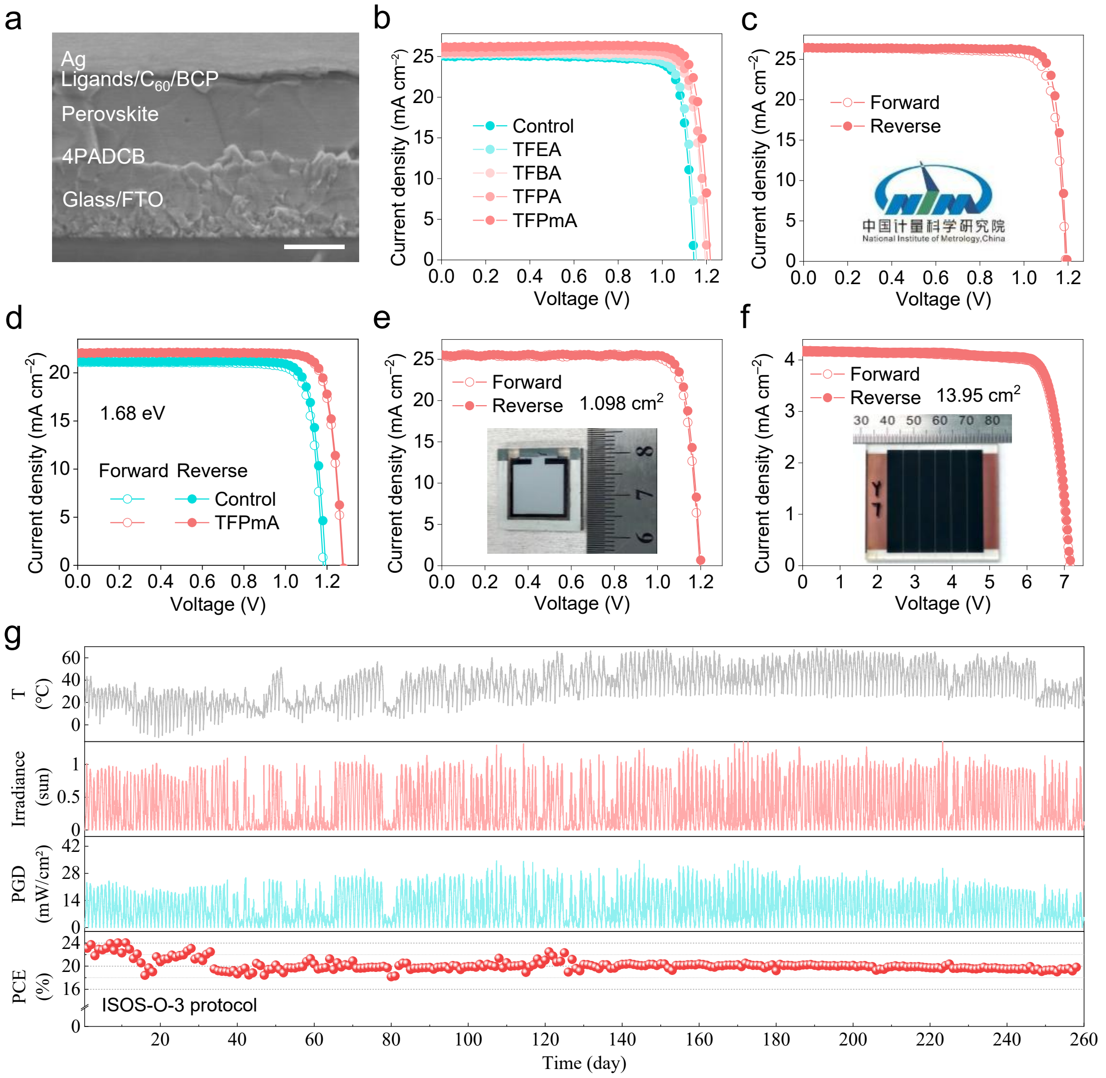

Figure R69 De vice performance and stability. (a) Cross-sectional structural image of perovskite solar cell (the scale bar is 500 nm). (b) *J-V* curves of the control and ligands treated devices. (c) Certified photovoltaic performance of devices obtained at NIM. (d) J-V curves of wide-bandgap (1.68 eV) devices for control and TFPmA treated devices. (e) *J-V* curves of 1.098 cm^2 devices for TFPmA-treated solar cells. (f) *J-V* curves of the TFPmA-treated module devices. (g) The real-time field testing of TFPmA-treated module under actual out-door operating conditions for 258 days. (**Updated Fig. 5**, in the revised manuscript)

A mismatch correction based on SR reference cell and perovskite solar cells needs to be calculated and considered for absolute IV values. Please add EQE data for control and target cell, best would be even for all passivant groups.

***Response*:** We thank the reviewer for this important point.

(1) After optimizing the encapsulation process, we resent the best-performing devices to the National Institute of Metrology (NIM) for certification again. The certified efficiency was further increased to **27.41%**, with a stabilized efficiency of 26.85%, as shown in **Figures R70** and **R71**. Spectral response (SR) calibration was also

performed (**Figure R72**), and spectral mismatch calculations yielded a mismatch factor of 1.0043.

中国计量科学研究院
National Institute of Metrology,China

Appendix: Summary of the Report
Report No.: GXgf2025-03922
Client: Shaanxi Normal University
Sample: Perovskite Solar Cell
Type/Model: Perovskite Solar Cell
DUT S/N: Y5-M0821-2
Manufacturer: Shaanxi Normal University
Sample temperature: (25±1)°C
Date of Test: 11/28/2025
Environmental conditions: (22 ± 1)°C RH (17± 2)%
Mask: An aperture area of 7.420 mm^2 (M0821-2, Certificate No.: CDjc2025-05685)

The test has been conducted by the PV Metrology Lab of NIM (National Institute of Metrology, China). Measurement of irradiance intensity and all other measurements are traceable to the International System of Units (SI). The data in this report is only applicable to the samples tested at that time. For more details, please refer to the text of the report.

	Area (mm^2)	I_{sc}(mA)	V_{oc}(V)	P_{max}(mW)
Reverse Scan	7.420	1.961	1.194	2.034
Forward Scan	7.420	1.961	1.188	1.955
	I_{max}(mA)	V_{max}(V)	FF (%)	η (%)
Reverse Scan	1.884	1.080	86.90	27.41
Forward Scan	1.844	1.060	83.89	26.35

I-V Characterization Methods:
JJF 1622-2017: Calibration Specification of Solar Cells: Photoelectric Properties
JJF 2129-2024: Calibration Specification for Perovskite Solar Cells: Photoelectric Characteristic Parameters
Reference Solar Cell:
Type: Mono-Si
Solar Simulator:
Classification: A+AA+ (Double-light source: Xeon and Halogen);
Total irradiance: 1000 W/m^2 based on I_{sc} of the above Reference Solar Cell.

Figure R70. Certified photovoltaic performance of devices obtained at NIM. (Updated **Supplementary Fig. 40**)

中国计量科学研究院
National Institute of Metrology, China

Report No.: GXgf2025-03922
DUT S/N: Y5-M0821-2

I-V characteristic curves and parameters (Reverse & Forward Scan)

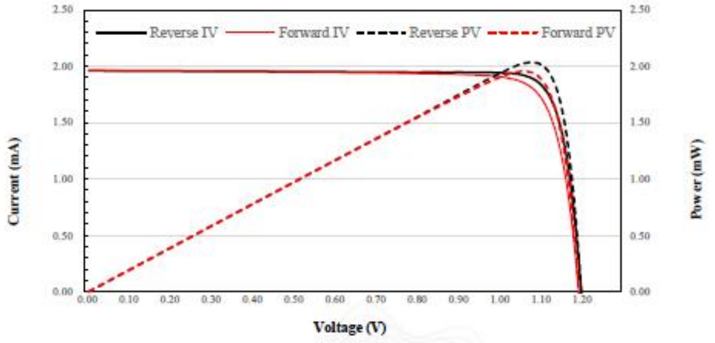

Steady-state scanning curve (V_{bias}=1.070 V, $t_{scanning}$ = 300 s, P_{start} = 2.007 mW, and P_{end} = 1.992 mW)

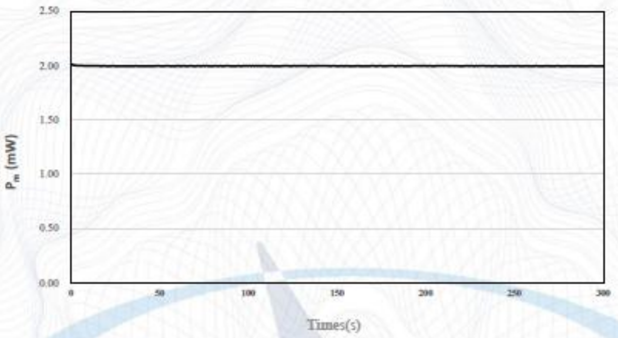

Figure R71. Certified photovoltaic performance of devices obtained at NIM. (Updated **Supplementary Fig. 41**)

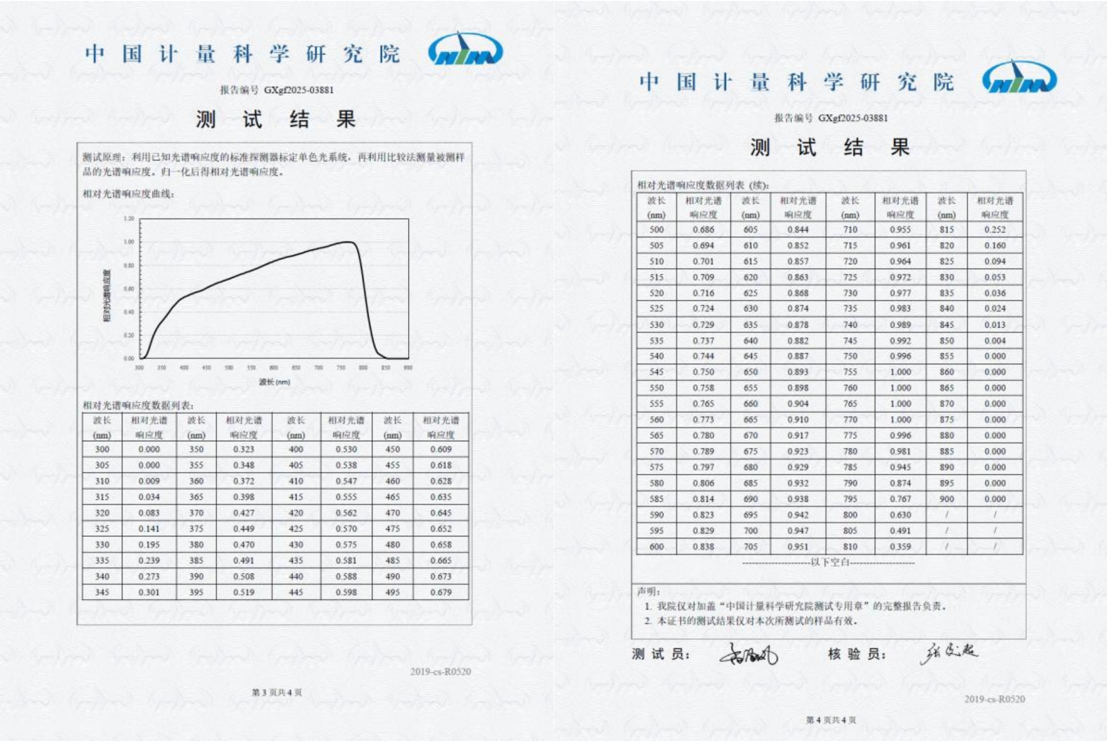

中 国 计 量 科 学 研 究 院

报告编号 GXgf2025-03881

测 试 结 果

测试原理：利用已知光谱响应度的标准探测器标定单色光系统，再利用比较法测量被测样品的光谱响应度。归一化后得相对光谱响应度。

相对光谱响应度曲线：

相对光谱响应度数据列表：

波长 (nm)	相对光谱响应度	波长 (nm)	相对光谱响应度	波长 (nm)	相对光谱响应度	波长 (nm)	相对光谱响应度
300	0.000	350	0.323	400	0.530	450	0.609
305	0.000	355	0.348	405	0.538	455	0.618
310	0.009	360	0.372	410	0.547	460	0.628
315	0.034	365	0.398	415	0.555	465	0.635
320	0.083	370	0.427	420	0.562	470	0.645
325	0.141	375	0.449	425	0.570	475	0.652
330	0.195	380	0.470	430	0.575	480	0.658
335	0.239	385	0.491	435	0.581	485	0.665
340	0.273	390	0.508	440	0.588	490	0.673
345	0.301	395	0.519	445	0.598	495	0.679

2019-cs-R0520

第 3 页共 4 页

中 国 计 量 科 学 研 究 院

报告编号 GXgf2025-03881

测 试 结 果

相对光谱响应度数据列表 (续):

波长 (nm)	相对光谱响应度	波长 (nm)	相对光谱响应度	波长 (nm)	相对光谱响应度	波长 (nm)	相对光谱响应度
500	0.686	605	0.844	710	0.955	815	0.252
505	0.694	610	0.852	715	0.961	820	0.160
510	0.701	615	0.857	720	0.964	825	0.094
515	0.709	620	0.863	725	0.972	830	0.053
520	0.716	625	0.868	730	0.977	835	0.036
525	0.724	630	0.874	735	0.983	840	0.024
530	0.729	635	0.878	740	0.989	845	0.013
535	0.737	640	0.882	745	0.992	850	0.004
540	0.744	645	0.887	750	0.996	855	0.000
545	0.750	650	0.893	755	1.000	860	0.000
550	0.758	655	0.898	760	1.000	865	0.000
555	0.765	660	0.904	765	1.000	870	0.000
560	0.773	665	0.910	770	1.000	875	0.000
565	0.780	670	0.917	775	0.996	880	0.000
570	0.789	675	0.923	780	0.981	885	0.000
575	0.797	680	0.929	785	0.945	890	0.000
580	0.806	685	0.932	790	0.874	895	0.000
585	0.814	690	0.938	795	0.767	900	0.000
590	0.823	695	0.942	800	0.630	/	/
595	0.829	700	0.947	805	0.491	/	/
600	0.838	705	0.951	810	0.359	/	/

--------------------以下空白--------------------

声明：

1. 我院仅对加盖"中国计量科学研究院测试专用章"的完整报告负责。
2. 本证书的测试结果仅对本次所测试的样品有效。

测 试 员： 核 验 员：

2019-cs-R0520

第 4 页共 4 页

Figure R72. Relative spectral response (SR) curves measured at NIM and the corresponding data table. (Updated **Supplementary Fig. 42**)

We have also added the **Figure R70, R71, R72** as **Supplementary Figs. 40-42** to the Supplementary Information and made the following corresponding revisions in the main text. The details are as follows:

"We sent the best-performing device to an accredited PV laboratory (the PV Metrology Lab of National Institute of Metrology, China) for third-party certification and obtained an efficiency of 27.41% a with stabilized power output efficiency of 26.85% (**Fig. 5c**, **Supplementary Figs. 39-42**)." (**Page 15**, in the revised manuscript)

(2) We supplemented the EQE data for the control and all ligand-passivated devices, as shown in **Figure R73**. The integral current for the control device was 24.47 mA cm^{-2}, while the integral currents for devices modified with TFEA, TFBA, TFPA, and TFPMA ligands were 24.51, 25.12, 25.24 and 25.29 mA cm^{-2}, respectively. These results are consistent with the current densities from the *J*-*V* curves (tolerance factors all below 3.2%).

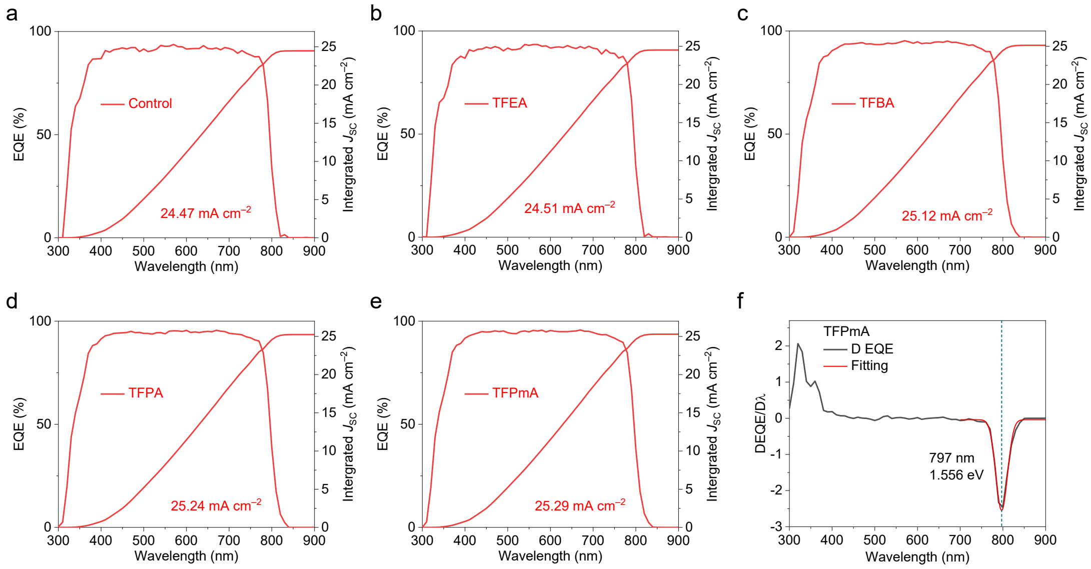

Figure R73. EQE spectrum for control and all ligand-passivated groups. (**Supplementary Fig. 43**)

We have added all devices EQE data (**Figure R73)** as **Supplementary Fig. 43** to the Supplementary Information and made the following corresponding revisions in the main text. The details are as follows:

"The external quantum efficiency (EQE) integrated currents agree with *J-V* measurements (tolerance variation < 3.2%) and the calculated bandgap of 1.556 eV confirmed a voltage loss of only 0.338 V (**Supplementary Fig. 43**)." (**Page 15**, in the revised manuscript)

E. Conclusions

The conclusion is robust and supported by the broad set of data.

It is recommended that the authors consider also the role of dipole changes at the interface in the light of field effect passivation and impact on charge distribution within the bulk material (doi.org/10.1002/aenm.202201109, doi: 10.1126/science.adx1745, etc).

***Response*:** Thanks for multi-dimensional perspective and important suggestions provided, which indeed serve as a valuable and constructive complement to our mechanistic understanding.

(1) We first reviewed the references recommended by the reviewers. Lars Korte et al. (doi.org/10.1002/aenm.202201109) investigated the interfacial energy and defect formation at the perovskite/C_{60} interface, achieving interfacial dipole regulation and field-effect passivation by introducing LiF intercalation at the interface. Qussama Er-raji et al. (10.1126/science.adx1745) treated the perovskite surface with 1,3-propanediamine iodide, increasing the concentration of majority carriers and reducing interfacial recombination. Simultaneously, this dipole changes enhanced the overall conductivity of the perovskite while reducing transport losses.

(2) Based on the above mechanism, we consider that changes in interfacial dipoles may also contribute to the field-effect passivation in our devices. The stereoelectronic design of the passivant molecules can alter the interfacial dipole moment, which modifies the local electric field at the perovskite/ETL boundary. Such a built-in field can repel minority carriers from the interface and suppress interfacial recombination, while simultaneously influencing charge distribution at the perovskite interface. The observed improvement in device performance can be further rationalized in terms of field-effect passivation arising from interfacial dipole modulation (**Supplementary Fig. 2**).

(3) Guided by the reviewer's helpful suggestions, we have incorporated the effect of dipole changes into our proposed mechanism. Specifically, we have added the corresponding discussion in the manuscript and cited the two references recommended by the reviewer. The details are provided below:

"…and a rise in molecular dipole moment that reshapes the local electrostatic landscape.[38,39]" (**Page 10**, in the revised manuscript)

F. Suggested Improvements

Major

- The core problem presented is named as "fundamental design paradox", however it seems rather a challenge then a real paradox. The fact that two parameters needs to be optimized at a time (or more) seems not enough; the author should clarify rethink the wording/presentation of the research challenge.

***Response*:** We appreciate your insightful comment. We agree that the term "fundamental design paradox" might be overstated. In the revised manuscript, we have rephrased this concept as a "materials design challenge", which more accurately reflects the need to simultaneously optimize multiple interdependent parameters.

"This trade-off poses a fundamental challenge in molecular design:" (**Page 3**, in the revised manuscript)

- Linked to this point clarification on existing literature on conjugated structures and other planar aligned passivation molecules should be added. As mentioned above the novelty should be clarified.

Response: We agree on the importance of clarifying the originality and novelty of our study relative to existing literature on planar-aligned and N-heteroaromatic passivation molecules.

The prevailing molecular design strategies for perovskite interfaces have largely relied on two established pillars: (1) vertical alignment for strong ionic bonding, and (2) the use of amine as terminal passivation groups. The present work fundamentally transcends both approaches by introducing a third dimension: the active control of adsorption topology through the electronic structure of the molecular core itself, thereby resolving the fundamental passivation-transport trade-off.

Below, we delineate the key conceptual advances:

(1) **A New Design Rule: Replacing Size-Matching with Electronic Driving Force**
Two publications demonstrated that planar molecules may improve performance based on 4ABS (Sargent et al., *Science* 2024, 384, 189–193) or CO-BSA (Peng et al., *Adv. Mater.* 2025, 2507918). However, their design logic was based on **static geometric compatibility**—finding a molecule whose pre-defined length fits the perovskite lattice.

We have established a more dynamic and predictive design rule. By progressively incorporating nitrogen atoms into the conjugated core, we actively inject a thermodynamic driving force that flips the molecular orientation from vertical to planar. The DFT calculations (**Figure 1c**) provide irrefutable evidence of this engineered switch, showing a reversal in adsorption energy preference (ΔE). Importantly we confirmed the **underlying passivation mechanism for the planar adsorption is multi-center interaction (Pb-N coordination *and* Pb-I-π), rather than size-matching**. Consequently, we have also advanced the field from *discovering* two molecules that happen to lie flat to *designing* molecules that are compelled to do so.

(2) **Redefining the Role of the N-Heterocycle: From Passive Site to Active Dictator**
The conventional role of N-heterocycles, as seen in works like 3APy (Zhu et al., *Nature* 2022, 611, 278–283), is a **passive defect-passivating functional group**, often leading to suboptimal charge transport distance.

We fundamentally repurpose the N-heterocycle. In our design, it is not a terminal group but **an integral component of the molecular backbone that acts as a stereoelectronic switch**. This critical repositioning unlocks **synergistic multicenter interactions (Pb-N coordination *and* Pb-I-π)**, which are the *direct cause* of both the planar alignment and the superior passivation. This transforms the N-heterocycle from a mere "defect healer" into an "**interface architect**".

(3) **The Synergistic Outcome: Decoupling the Fundamental Trade-off**
The aforementioned advances culminate in a decisive resolution of the passivation-transport paradox. Our "stereoelectronic manipulation" creates a unique interfacial architecture where the **same molecular feature** that enhances binding energy for passivation **simultaneously minimizes charge transport distance by ~37% (Figure 1e)**.

(4) In short, while previous three Nature/Science publications identified *three* molecules could work as passivants for solar cells, the state-of-the-art strategies including traditional vertical ligands and above geometric size-matching passivants **still fail to resolve the core dilemma**: how to synergistically manage interfacial energy losses through combined defect passivation and transport-geometry optimization to approach the SQ limit.

Our work **provide** the precise mechanism behind effective passivation. This establishes **"stereoelectronic manipulation" as a guiding principle for future interfacial design, charting a path toward the SQ limit**.

Per your suggestion, we have expressed this perspective by adding the following into the revised manuscript.

"Yet, even state-of-the-art remedies including those employing vertical ligands or geometric size-matching passivants,[13,24-26] leave this fundamental dilemma unresolved." (**Page 3**, in the revised manuscript)

- The authors talk about defect passivation and name Pb-N and Pb-I-pi interaction, though when stating in the text no direct references are given. As the paper does not address the bulk perovskite at least literature references should be added.

Response: We thank the reviewer for this helpful remark. In the revised manuscript, we have added explicit references supporting the proposed Pb–N coordination and Pb–I–π interaction mechanisms. These citations are now included in the revised manuscript:

"consistent with ongoing defect passivation that may involve Pb–N coordination and Pb–I–π interactions.[12,40]" (**Page 12**, in the revised manuscript).

Connected to this, generally little info on the perovskite absorber is given. However, the interaction of the molecule is very much dependent of the perovskite crystal termination and should be thus described more. Please add a chemical formula for the perovskite composition (for all perovskites used) and clarify why (only) Pb-I termination has been looked at, e.g. in the DFT study. Would the molecule effect be similarly on MA-terminated surfaces?

Response: In direct response to your valuable suggestion, we have updated the manuscript to explicitly detail the nominal perovskite compositions employed.

(1) The compositions used for the core characterizations and devices are:

- **Normal-bandgap $FAPbI_3$-based perovskite (1.56 eV):** $FA_{0.85}MA_{0.1}Cs_{0.05}Pb(I_{0.98}Br_{0.02})_3$
- **Wide-bandgap perovskite (1.68 eV):** $Cs_{0.22}FA_{0.78}Pb(I_{0.85}Br_{0.15})_3$

The corresponding text sections have been revised in the Supplementary Information and main text:

"Normal-bandgap $FAPbI_3$-based perovskite (1.56 eV) solution (1.5 M, $FA_{0.85}MA_{0.1}Cs_{0.05}Pb(I_{0.98}Br_{0.02})_3$) was prepared…

Wide-bandgap perovskite (1.68 eV) solution (1.3 M, $Cs_{0.22}FA_{0.78}Pb(I_{0.85}Br_{0.15})_3$) was prepared…" (1.2 Perovskite precursor solution, in the Supplementary Information).

(2) Regarding the fundamental characterizations, all measurements were performed using a normal-bandgap perovskite composition ($FA_{0.85}MA_{0.1}Cs_{0.05}Pb(I_{0.98}Br_{0.02})_3$). To further clarify this point for readers, we have added an explicit description in the film characterization section of the Supplementary Information.

"Unless otherwise specified, all characterizations were performed on $FAPbI_3$-based perovskite ($FA_{0.85}MA_{0.1}Cs_{0.05}Pb(I_{0.98}Br_{0.02})_3$) films fabricated following our standard one-step spin-coating protocol." (1.5 Film characterization, in the Supplementary Information)

"The XPS spectra of calibrated C 1*s* and F 1*s* references for $FAPbI_3$-based perovskite with ligands are provided in **Supplementary Figs. 6** and **7**." (**Page 7**, in the revised manuscript)

"analyses of $FAPbI_3$-based perovskite films verified that …" (**Page 9**, in the revised manuscript)

"Following our device protocol, we spin-coated the ligand solution onto the $FAPbI_3$-

based perovskite film …” (**Page 12**, in the revised manuscript)

(3) We have performed DFT calculations to compare the thermodynamic stability of ligand adsorption on Pb–I–terminated and FA–I–terminated perovskite surfaces (see **Figure R74**). The results suggest that ligand adsorption on FA/MA–terminated facets is primarily governed by relatively weak hydrogen-bonding and dipolar interactions (**Table R7**), consistent with previous reports (**Science**, https://www.science.org/doi/10.1126/science.adx1745). As a consequence of these weaker interactions, the calculated adsorption energies on FA/MA–I–terminated surfaces are comparatively small, with E_{ads} (vertical) = –0.45, –0.65, –0.52, and –0.51 eV and E_{ads} (planar) = –0.21, –0.46, –0.36, and –0.44 eV for TFEA, TFBA, TFPA, and TFPmA, respectively. In contrast, stronger adsorption is obtained on Pb–I–terminated surfaces (**Figure R75** and **Table R8**), where E_{ads} (vertical) = –0.97, –1.16, –1.13, and –1.10 eV and E_{ads} (planar) = –0.40, –1.15, –1.31, and –1.37 eV for the same ligands. Taken together, these calculations are consistent with a thermodynamic preference for ligand adsorption on Pb–I–terminated surfaces relative to FA–I–terminated facets.

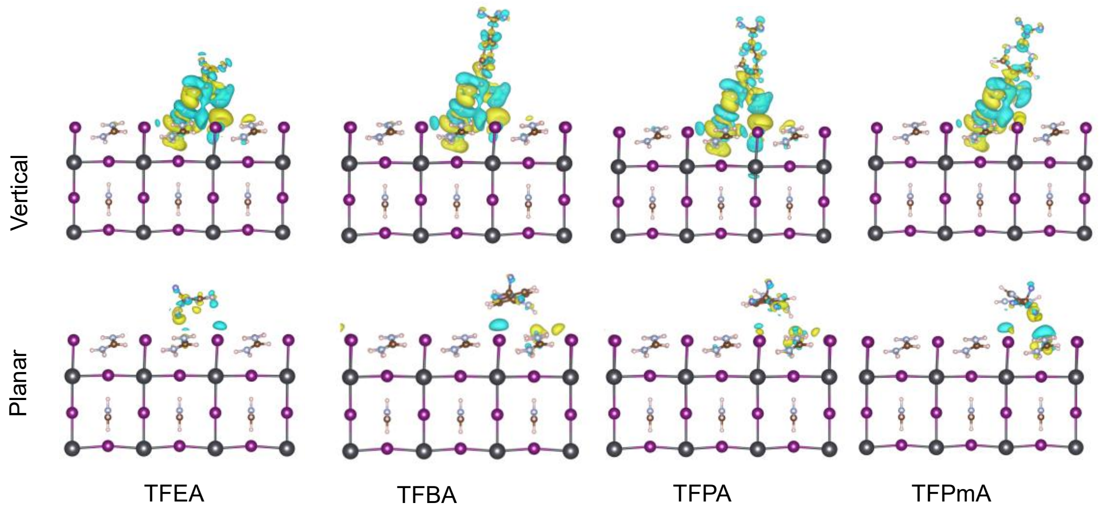

Figure R74. The adsorption configurations of different ligands on the FA-I terminal of perovskite after expanded the k-point grid to (2×2×1) while increasing the Plane-wave cutoff to 500 eV.

Table R7. he adsorption energy of different ligands on the FA-I terminal of perovskite.

	TFEA	TFBA	TFPA	TFPmA
E_{ads} (vertical)	-0.45	-0.65	-0.52	-0.51
E_{ads} (planar)	-0.21	-0.46	-0.36	-0.44
E_{ads} (planar)- E_{ads} (vertical)	**0.24**	**0.19**	**0.16**	**0.07**

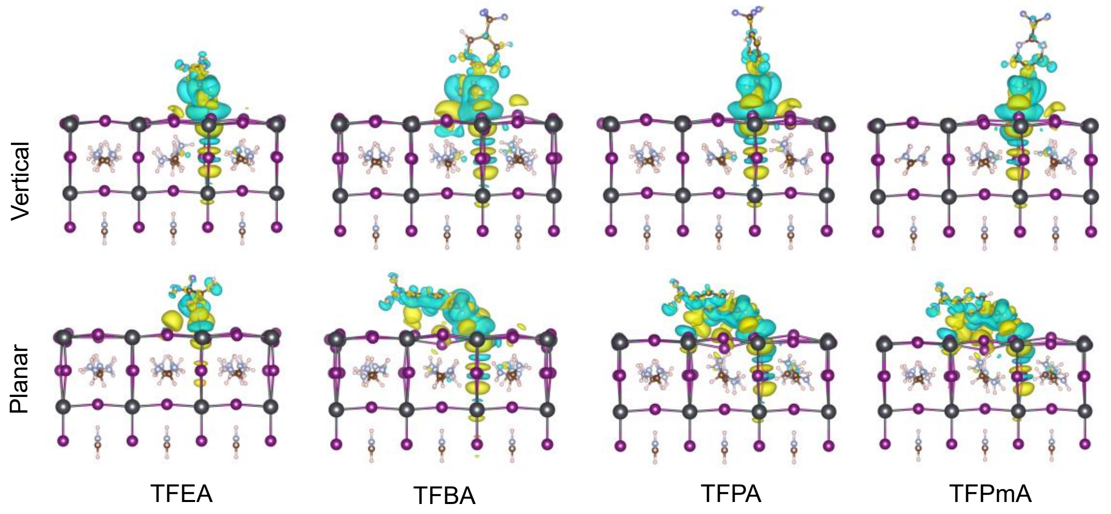

Figure R75. The adsorption configurations of different ligands on the Pb-I terminal of perovskite after expanded the k-point grid to (2×2×1) while increasing the Plane-wave cutoff to 500 eV.

Table R8. he adsorption energy of different ligands on the Pb-I terminal of perovskite.

	TFEA	TFBA	TFPA	TFPmA
E_{ads} (vertical)	-0.97	-1.16	-1.13	-1.10
E_{ads} (planar)	-0.40	-1.15	-1.31	-1.37
E_{ads} (planar)-E_{ads} (vertical)	**0.57**	**0.01**	**-0.18**	**-0.27**

(3) We also notice that our approach of using Pb–I–terminated surfaces is also consistent with previous reports for perovskite surface modelling. For example, a Pb–I–terminated surface was employed to model the perovskite/C_{60} interface when investigating the role of Nd@C_{82}–PMMA in ultrafast interfacial electron extraction, where the authors achieved good agreement with experimental observations (**Figure R76a**, ***Nature*** 2025, 642, 78–84). Similarly, Pb–I–terminated surfaces were used to elucidate the adsorption behavior of different ligands on perovskite surfaces (**Figure R76b**, ***Science*** 2024, 384, 189–193). Recent reports also adopted Pb–I–terminated surface models to describe the interfacial binding energetics at PbI_2-rich $FAPbI_3$ (100)/ligand interfaces (**Figure R76c**, ***Sci. Adv***. 2024, 10, eadq1150) and to investigate ligand-mediated interactions at perovskite/SnO_2 interfaces (**Figure R76d**, ***Science*** 2024, 386, 187–192).

The prevalence of Pb–I-terminated surfaces in perovskite DFT modeling stems from two principal factors. First, high-efficiency devices are often fabricated with slightly PbI_2-rich precursor compositions, which favor Pb–I-rich terminations on the actual perovskite surface. Second, under typical experimental conditions, Pb–I-terminated surfaces are thermodynamically more stable than FA/MA-I-terminated ones. For these reasons, Pb–I-terminated surfaces have become a widely adopted and physically relevant model in high-impact studies of perovskite interface energetics.

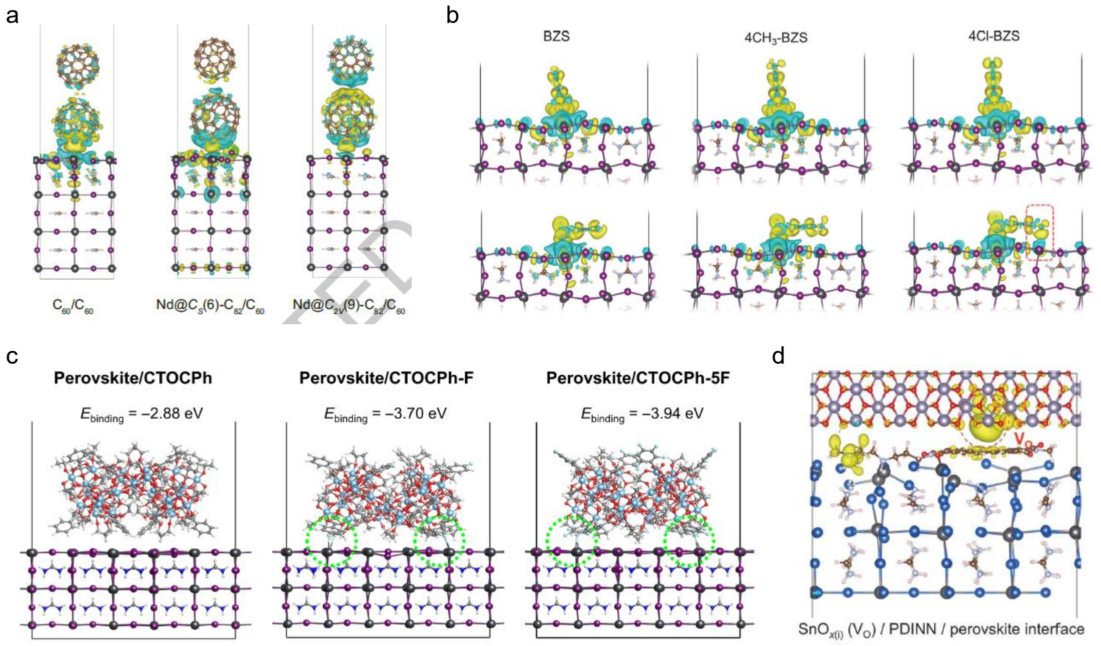

Figure R76. (a) Charge density difference (CDD) distributions of perovskite (with PbI2- terminated $FAPbI_3$ surface)/fullerene slabs (Copyright from *Nature* 2025, 642, 78–84). (b) DFT studies of ligand binding and predicted orientation (Copyright from *Science* 2024, 384, 189–193). (c) Calculated binding energy ($E_{binding}$) for PbI_2-rich $FAPbI_3$ (100)/CT OCPh, PbI_2-rich $FAPbI_3$ (100)/CT OCPh-F, PbI_2-rich $FAPbI_3$ (100)/CT OCPh-5F (Copyright from *Sci. Adv.* 2024, 10, eadq1150). (d) Illustration of the simulated charge distribution (corresponding to CBM) at the $SnO_{x(i)}$ (V_O)/PDINN/perovskite interface (Copyright from *Science* 2024, 386, 187–192).

(4) To further address the reviewer's point, we also extended our analysis to FA/MA–I-terminated surfaces, comparing the stability of planar and vertical adsorption modes $\Delta E = E_{ads}(planar) - E_{ads}(vertical)$ (**Table R7**, **Figure R77**). We find that ΔE becomes less positive with higher N heteroatom incorporation, demonstrating **a reduced stability cost for planar adsorption**. Thus, increasing N content promotes a transition toward planar geometry, thereby corroborating the central adsorption trend established in the main text for different surfaces.

In summary, in light of the reviewer's comments and a broader survey of the literature, we performed complementary calculations on FA–I-terminated surfaces. Together with our original Pb–I-terminated model, these results confirm that: **regardless of the surface termination (Pb–I or FA–I), a higher N heteroatom content in the ligand systematically promotes a transition from vertical to planar adsorption geometries**. This additional analysis indicates the general validity of our proposed adsorption mechanism.

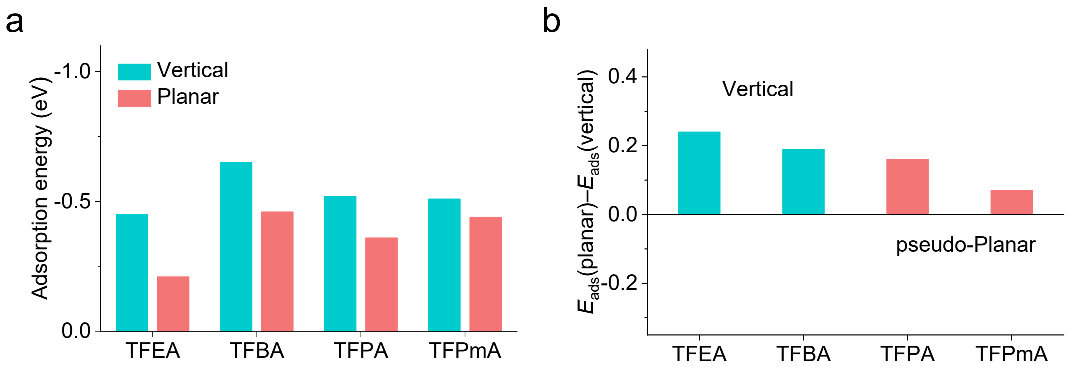

Figure R77. (a) The adsorption energy of different ligands on the FA-I terminal of perovskite after expanded the k-point grid to (2×2×1) while increasing the Plane-wave cutoff to 500 eV. (b) Formation-energy differences (ΔE = E_{ads}(planar) − E_{ads}(vertical)) between vertical and planar adsorption topology for each ligand on the FA-I terminal perovskite surface.

We have also included **Figures R74** and **R77** as **Supplementary Figs. 4** and **5** in the Supplementary Information. Corresponding descriptions have been added both in the main text and in **Supplementary Note 1**, as detailed below:

"This thermodynamic preference shift is also observed on FA–I-terminated surfaces (**Supplementary Figs. 4** and **5,** and **Supplementary Note 1**)." (**Page 5**, in the revised manuscript)

"On Pb–I–terminated surfaces, the formation-energy differences (ΔE = $E_{ads(planar)}$ − $E_{ads(vertical)}$) for TFEA, TFBA, TFPA, and TFPmA decrease sequentially from 0.57 to 0.01, −0.18, and −0.27 eV, respectively. For TFBA, ΔE is close to zero (0.01 eV), suggesting no strong configurational preference between vertical and planar adsorption. This trend indicates a gradual thermodynamic shift in preferred adsorption topology from predominantly vertical (TFEA), to nearly non-preferential or mixed configurations (TFBA), and further toward planar geometries (TFPA and TFPmA) as the number of N-heteroaromatic units increases.

In comparison, the calculated adsorption energies on FA–I–terminated surfaces are relatively smaller, with E_{ads}(vertical) values of −0.45, −0.65, −0.52, and −0.51 eV and E_{ads}(planar) values of −0.21, −0.46, −0.36, and −0.44 eV for TFEA, TFBA, TFPA, and TFPmA, respectively. Further analysis of the corresponding formation-energy differences (ΔE) reveals a similar decreasing trend with increasing N content, indicating that higher N incorporation progressively lowers the energetic penalty for planar adsorption. Collectively, these results suggest that increasing N heteroatom content promotes a tendency toward planar adsorption geometries across different surface terminations, thereby corroborating the central adsorption trend established in the main text." (In the **Supplementary Note 1**)

- A mismatch factor should be considered for such high-efficiency values to allow fair comparison. Info on how the certified measurements were conducted in terms of considering a SR mismatch factor is recommended.

***Response*:** Thanks for your comments. After optimizing the encapsulation process, we resend the best-performing devices to the National Institute of Metrology (NIM) for certification. The **certified efficiency was further increased to 27.41%**, with a stabilized efficiency of 26.85%, as shown in **Figures R78** and **R79**. Spectral response (SR) calibration was also performed (**Figure R80**), and spectral mismatch calculations yielded a mismatch factor of 1.0043.

中国计量科学研究院
National Institute of Metrology,China

Appendix: Summary of the Report
Report No.: GXgf2025-03922
Client: Shaanxi Normal University
Sample: Perovskite Solar Cell
Type/Model: Perovskite Solar Cell
DUT S/N: Y5-M0821-2
Manufacturer: Shaanxi Normal University
Sample temperature: (25±1)°C
Date of Test: 11/28/2025
Environmental conditions: (22 ± 1)°C RH (17± 2)%
Mask: An aperture area of 7.420 mm^2 (M0821-2, Certificate No.: CDjc2025-05685)

The test has been conducted by the PV Metrology Lab of NIM (National Institute of Metrology, China). Measurement of irradiance intensity and all other measurements are traceable to the International System of Units (SI). The data in this report is only applicable to the samples tested at that time. For more details, please refer to the text of the report.

	Area (mm^2)	I_{sc}(mA)	V_{oc}(V)	P_{max}(mW)
Reverse Scan	7.420	1.961	1.194	2.034
Forward Scan	7.420	1.961	1.188	1.955
	I_{max}(mA)	V_{max}(V)	FF (%)	η (%)
Reverse Scan	1.884	1.080	86.90	27.41
Forward Scan	1.844	1.060	83.89	26.35

I-V Characterization Methods:
JJF 1622-2017: Calibration Specification of Solar Cells: Photoelectric Properties
JJF 2129-2024: Calibration Specification for Perovskite Solar Cells: Photoelectric Characteristic Parameters
Reference Solar Cell:
Type: Mono-Si
Solar Simulator:
Classification: A+AA+ (Double-light source: Xeon and Halogen);
Total irradiance: 1000 W/m^2 based on I_{sc} of the above Reference Solar Cell.

Figure R78. Certified photovoltaic performance of devices obtained at NIM. (Updated **Supplementary Fig. 40**)

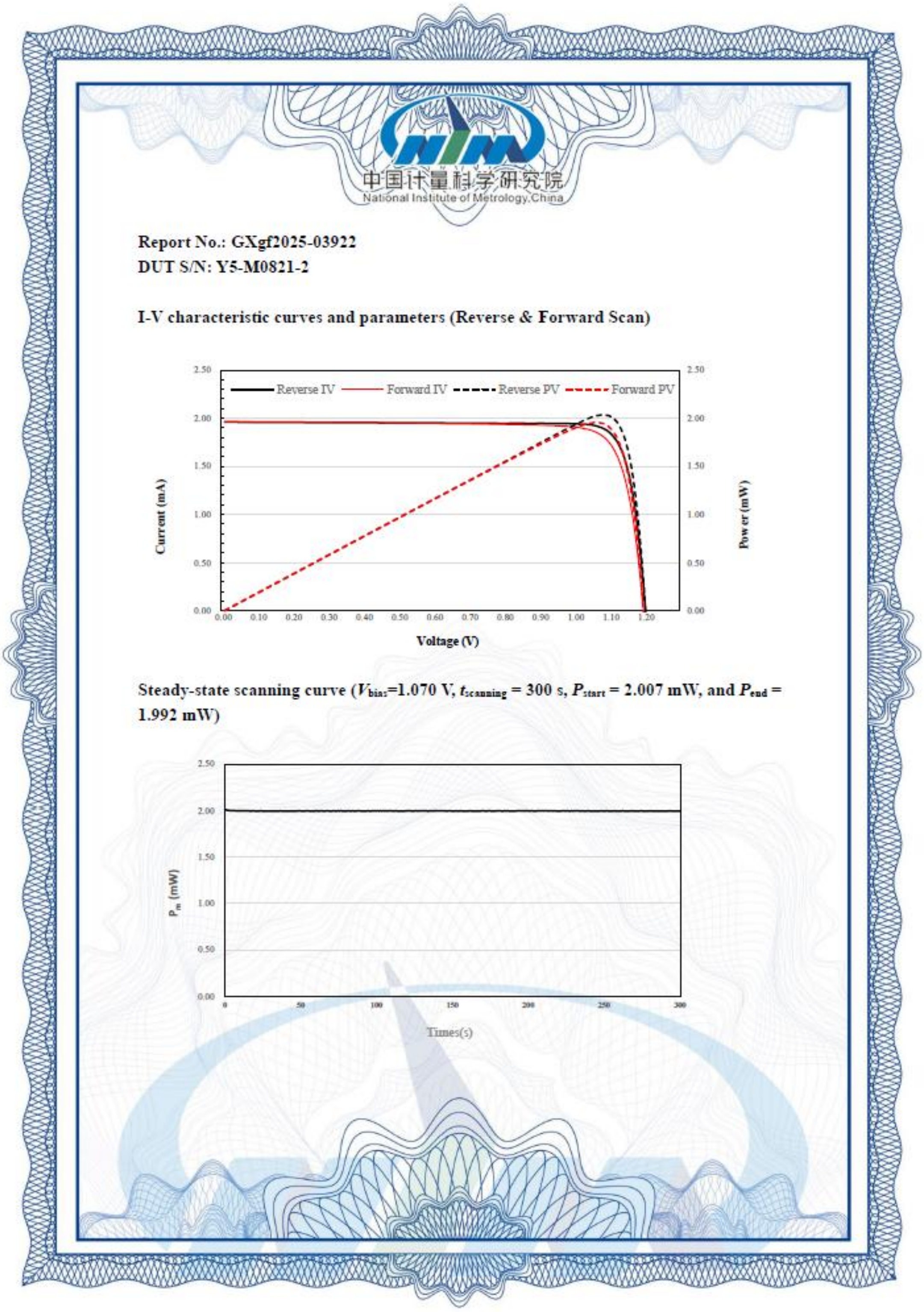
中国计量科学研究院
National Institute of Metrology, China

Report No.: GXgf2025-03922
DUT S/N: Y5-M0821-2

I-V characteristic curves and parameters (Reverse & Forward Scan)

Steady-state scanning curve (V_{bias}=1.070 V, $t_{scanning}$ = 300 s, P_{start} = 2.007 mW, and P_{end} = 1.992 mW)

Figure R79. Certified photovoltaic performance of devices obtained at NIM. (Updated **Supplementary Fig. 41**)

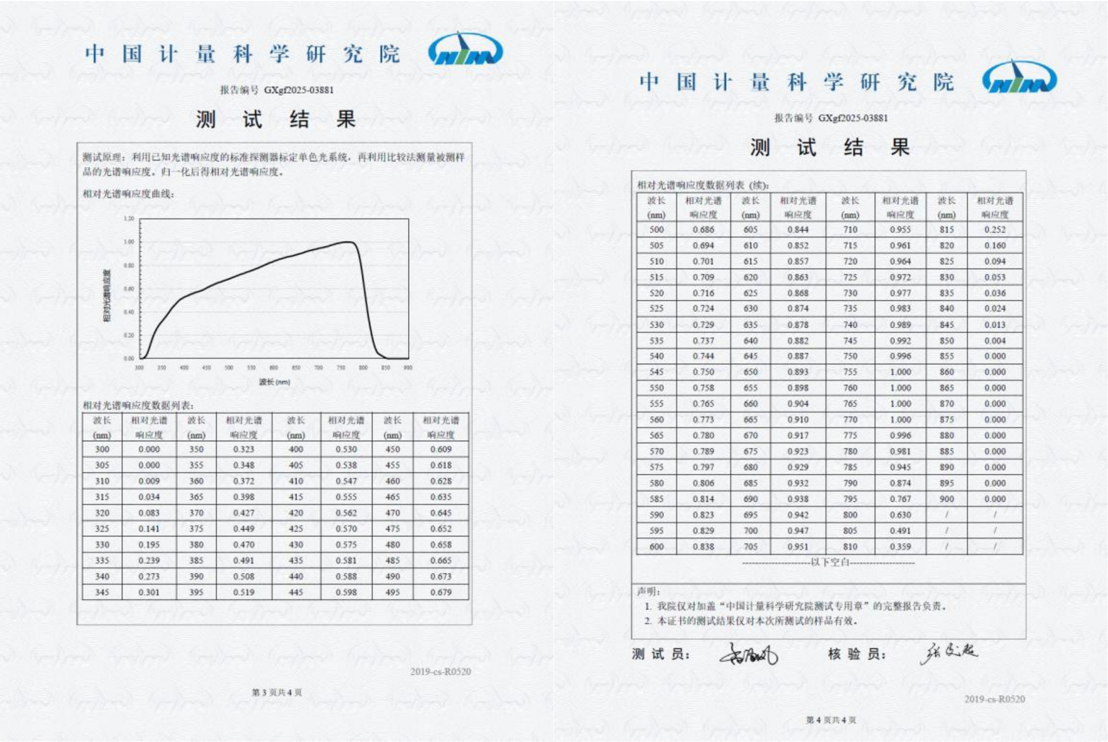

中 国 计 量 科 学 研 究 院

报告编号 GXgf2025-03881

测 试 结 果

测试原理：利用已知光谱响应度的标准探测器标定单色光系统，再利用比较法测量被测样品的光谱响应度，归一化后得相对光谱响应度。

相对光谱响应度曲线：

相对光谱响应度数据列表：

波长 (nm)	相对光谱响应度	波长 (nm)	相对光谱响应度	波长 (nm)	相对光谱响应度	波长 (nm)	相对光谱响应度
300	0.000	350	0.323	400	0.530	450	0.609
305	0.000	355	0.348	405	0.538	455	0.618
310	0.009	360	0.372	410	0.547	460	0.628
315	0.034	365	0.398	415	0.555	465	0.635
320	0.083	370	0.427	420	0.562	470	0.645
325	0.141	375	0.449	425	0.570	475	0.652
330	0.195	380	0.470	430	0.575	480	0.658
335	0.239	385	0.491	435	0.581	485	0.665
340	0.273	390	0.508	440	0.588	490	0.673
345	0.301	395	0.519	445	0.598	495	0.679

2019-cs-R0520

第 3 页共 4 页

中 国 计 量 科 学 研 究 院

报告编号 GXgf2025-03881

测 试 结 果

相对光谱响应度数据列表 (续)：

波长 (nm)	相对光谱响应度	波长 (nm)	相对光谱响应度	波长 (nm)	相对光谱响应度	波长 (nm)	相对光谱响应度
500	0.686	605	0.844	710	0.955	815	0.252
505	0.694	610	0.852	715	0.961	820	0.160
510	0.701	615	0.857	720	0.964	825	0.094
515	0.709	620	0.863	725	0.972	830	0.053
520	0.716	625	0.868	730	0.977	835	0.036
525	0.724	630	0.874	735	0.983	840	0.024
530	0.729	635	0.878	740	0.989	845	0.013
535	0.737	640	0.882	745	0.992	850	0.004
540	0.744	645	0.887	750	0.996	855	0.000
545	0.750	650	0.893	755	1.000	860	0.000
550	0.758	655	0.898	760	1.000	865	0.000
555	0.765	660	0.904	765	1.000	870	0.000
560	0.773	665	0.910	770	1.000	875	0.000
565	0.780	670	0.917	775	0.996	880	0.000
570	0.789	675	0.923	780	0.981	885	0.000
575	0.797	680	0.929	785	0.945	890	0.000
580	0.806	685	0.932	790	0.874	895	0.000
585	0.814	690	0.938	795	0.767	900	0.000
590	0.823	695	0.942	800	0.630	/	/
595	0.829	700	0.947	805	0.491	/	/
600	0.838	705	0.951	810	0.359	/	/

--------------------以下空白--------------------

声明：
1. 我院仅对加盖"中国计量科学研究院测试专用章"的完整报告负责。
2. 本证书的测试结果仅对本次所测试的样品有效。

测 试 员：　　　　核 验 员：

2019-cs-R0520

第 4 页共 4 页

Figure R80. Relative spectral response (SR) curves measured at NIM and the corresponding data table. (Updated **Supplementary Fig. 42**)

We have also added the **Figure R78, R79, R80 as Supplementary Figs. 40-42** to the Supplementary Information and made the following corresponding revisions in the main text. The details are as follows:

"We sent the best-performing device to an accredited PV laboratory (the PV Metrology Lab of National Institute of Metrology, China) for third-party certification and obtained an efficiency of 27.41% a with stabilized power output efficiency of 26.85% **(Fig. 5c**, **Supplementary Figs. 39-42**)." (**Page 15**, in the revised manuscript)

Minor

- Please reconsider to change the word manipulation to design in the title.

Response**:** We appreciate your suggestion regarding the title wording. In this work, the term "manipulation" was chosen to **emphasize the active tuning** of electronic structure and adsorption configuration of the ligands through stereoelectronic control. The study focuses **not only on designing new molecular structures but also on actively manipulating** their charge distribution and interfacial alignment to achieve desired electronic effects. We thus think that "Stereoelectronic Manipulation" reflects the core concept of this research and the integrated experimental–theoretical approach presented.

That said, **we remain open** to reconsidering this wording should the reviewer has further specific concerns or suggestions regarding the term's interpretation.

- In the abstract the wording "conjugated backbone of ligands" is used, more precise naming/description could help the reader.

Response**:** Thank you very much for your helpful comment. In the revised version, we have clarified the molecular structure of the ligands by specifying the type of conjugated backbone used. Specifically, we now describe the ligands as follows:

"By strategically replacing benzene carbons with nitrogen atoms to create pyridine or pyrimidine rings" (**Abstract**, in the revised manuscript)

- The wording sub-nanometer charge transport pathways seem misleading as KPFM and nano-IR maps indicate inhomogeneous formation of the vertical aligned passivants on the perovskite surface. Wouldn´t this also allow sub-nanometer charge transport channels?

Response**:** Thanks for this insightful observation.

(1) "Sub-nanometer charge transport pathways" refers to the short tunneling distance across planar-oriented passivant molecules. While we observed nanoscale heterogeneity in the KPFM and nano-IR maps, this does not preclude the formation of such pathways. The heterogeneity reflects spatial variations in molecular density, not the absence of local molecular alignment. **Crucially, in aggregated regions, the statistically dominant sub-nanometer tunnels of planar-oriented molecules is highly required for efficient charge transfer across the interface.**

(2) To avoid possible misinterpretation, we have also slightly revised the wording in the manuscript to:

"Theoretical simulation reveals that the ligand adsorption topology opens both sub-nanometer charge-transfer channels and enhances the perovskite-ligand-C_{60} electrostatic coupling, yielding an interfacial electronic landscape that favours efficient charge extraction." (**Page 5**, in the revised manuscript)

- Please add references in the introduction when naming ranges for lifetime, trap density, transmission coefficient, series resistance.

Response**:** We appreciate your careful attention to quantitative accuracy. In the revised manuscript, we have added representative literature references to support the numerical ranges of carrier lifetime, trap density, transmission coefficient, and series resistance mentioned in the Introduction. To avoid overly absolute statements, we have also made the following modifications to the original text:

"One is defect-mediated non-radiative recombination, which is dominated by Shockley-Read-Hall (SRH) kinetics with characteristic abundant interfacial trap states at untreated interfaces.[14-16] Another is interfacial transport resistance limited by quantum-mechanical transmission and wavefunction mismatch across the heterojunction.[17,18]" (**Page 3**, in the revised manuscript)

- Please check the wording "intercalation" of amine terminal into the Pb-I framework.

Response**:** Thank you very much for your careful attention to the language used in the

manuscript. With respect to the term "intercalation", it is used here to **describe the insertion of the amine end groups** of the ligands into the perovskite framework. In fact, within commonly accepted structural models of halide perovskites, the insertion of MA^+- or FA^+-like ammonium head groups into the Pb–I lattice has been widely recognized, and the term "intercalation" has been frequently used in the literature to describe this process.

For example, in conventional two-dimensional perovskites (as illustrated in **Figure R81**), the ammonium end groups of organic cations insert into the Pb–I framework, forming layered perovskite structures with different n values (*Chemical Reviews* 2021**,** *121*, 2230-2291; *Adv. Optical Mater.* 2023, *11*, 2202449). Similarly, following surface passivation treatments (**Figure R82**), the MA/FA-type end groups of ligand molecules can also insert into the topmost Pb–I lattice, thereby passivating defects such as V_{MA} and V_{FA} (***Nat. Photon***. 2019, 13, 460–466; ***Adv. Mater***. 2024, 36, 2310203; ***Nat. Energy*** 2024, 9, 779–792; ***Nature*** 2024, 635, 867–873).

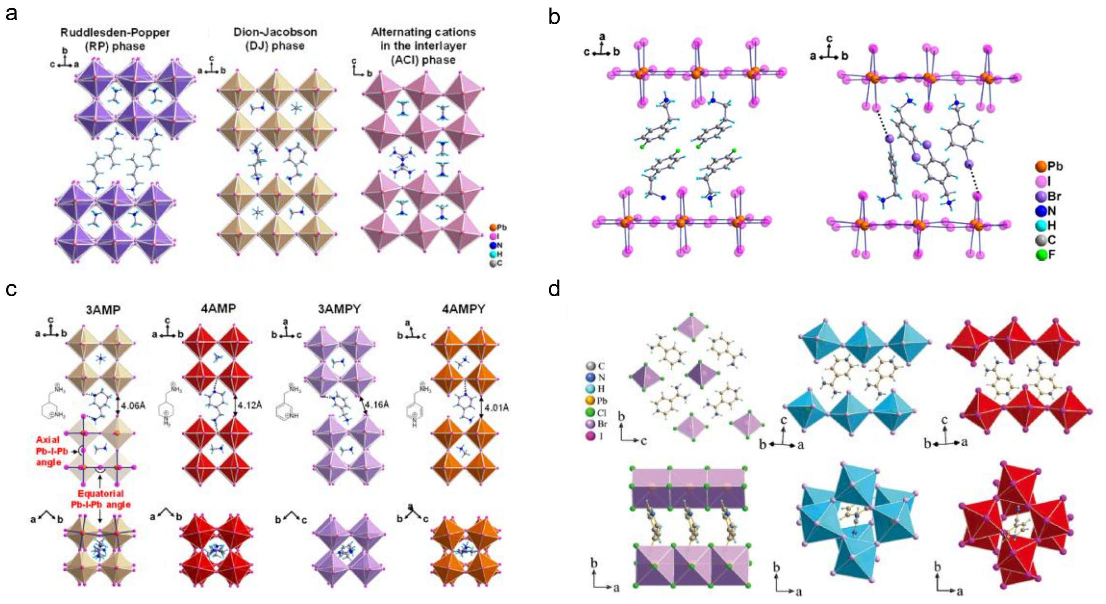

Figure R81. a, b, c. The crystal structure of different ligand molecules intercalate into lead-iodine framework in 2D perovskites. (Copyright from *Chemical Reviews* 2021**,** *121*, 2230-2291). d The crystal structure of (4AP)PbX_4 (X = Cl, Br, I) single crystals. (Copyright from *Adv. Optical Mater.* 2023, *11*, 2202449)

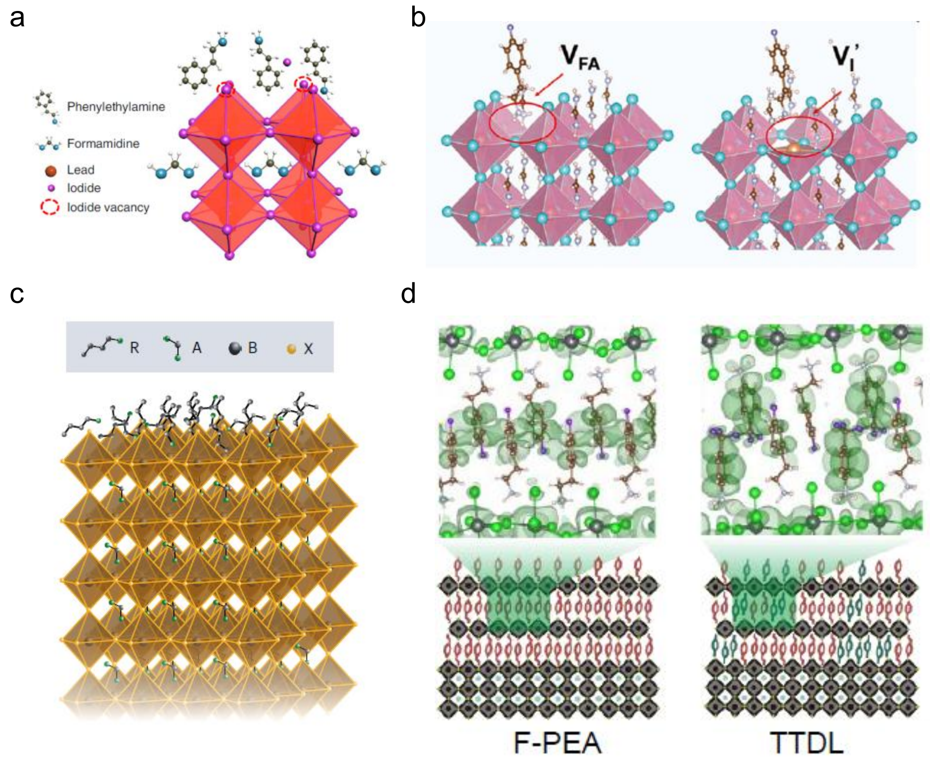

Figure R82. **a.** Possible passivation mechanism of the PEAI layer for the perovskite film. (Copyright from *Nat. Photon.* 2019, 13, 460–466). **b.** Schematic diagram of binding interaction between $4FPEA^+$ and FA-I terminated surface with defects of V_{FA} and V_I' (Copyright from *Adv. Mater.* 2024, 36, 2310203). **c.** Perovskite crystal surfaces passivated by either a molecular cation passivation layer (Copyright from *Nat. Energy* 2024, 9, 779–792). **d.** $Bohr^{-3}$ of F-PEA-based 2D perovskite and a possible molecular arrangement in a portion of the TTDL. (*Nature* 2024, 635, 867–873)

- It is not clear what is meant by "atomic-scale proximity to the SQ limit". Can the authors please clarify/adapt the wording.

Response**:** Thanks for your valuable comment. The phrase "atomic-scale proximity to the SQ limit" describes the concept of using atomic/molecular-scale interfacial control to mitigate energy loss, thereby pushing device efficiency toward the Shockley-Queisser (SQ) limit. Our stereoelectronic modulation strategy aims to achieve precisely this by enabling atomic-scale management of interfacial losses.

"thereby enabling minimum interfacial losses" (**Page 3**, in the revised manuscript)

"The sub-0.1 eV energy offset at the TFPmA/C_{60} interface drives the energy-barrier to the theoretical minimum, enabling quasi-ideal charge extraction across the heterojunction." (**Page 10**, in the revised manuscript)

- Please check/clarify the wording "ambidentate" (four ambidentate ligands were engineered…) as besides the amidine group the -CF3 group is mentioned. Is this a functional group that can be considered a "dent". Not a classical lewis base/acid to

donate/accept free electrons.

***Response*:** Thank you for the careful evaluation of our manuscript and for the professional guidance provided. We fully agree with the reviewer's suggestion, and our considerations and corresponding revisions are outlined below:

(1) After reviewing the relevant literature, **we agree** that the $–CF_3$ group does not act as a coordination site in the classical sense and therefore should not be regarded as a "dent." Our original intention was to emphasize the multifunctional nature of the ligands rather than to imply the presence of multiple classical coordination atoms.

(2) To avoid this ambiguity, we followed the reviewer's suggestion and **have replaced the term "ambidentate" with "multifunctional"**, which more accurately reflects their distinct roles: the amidino group ($–C(=NH)NH_2^+$) coordinates with undercoordinated Pb^{2+} for defect passivation, while the $–CF_3$ terminal contributes to enhanced interfacial hydrophobicity and environmental stability. The revised text now reads:

"Four multifunctional ligands were engineered…." (**Page 4**, in the revised manuscript)

- Generally authors are encouraged to check their manuscript for easy accessible language. Some sentences could be improved, e.g. "This dichotomy creates a fundamental material design paradox" or "QFLS analysis quantified the interfacial defect suppression hierarchy, …..arises from a tripartite cooperation with topographic defect healing via multicenter interactions".

***Response*:** We once again thank you very much for your extensive expertise and patience throughout the review process. We have carefully reread the manuscript in its entirety and made further refinements to the language at multiple points.

In the revised manuscript, we have simplified and clarified several sentences to enhance readability while maintaining scientific precision. Specifically:

(1) "This dichotomy creates a fundamental material design paradox" has been revised to "This trade-off poses a fundamental challenge in molecular design:" (**Page 3**, in the revised manuscript)

(2) "Quasi-Fermi level splitting (QFLS) analysis for FTO/4PADCB/perovskite/ligands samples quantified a hierarchy of perovskite/ligands interfacial defect suppression, showing a monotonic increase from 1.175±0.012 eV (control, without ligands) to 1.236±0.009 eV (TFPmA) (**Fig. 3f**). This 61 meV enhancement, enabled by stereoelectronic manipulation, stems from above observed synergistic interfacial mechanisms: topographic defect healing through multicenter interactions, delocalized electronic screening mediated by Pb–I–π interactions, a reduction in the interfacial energetic barrier,[13,24] and a rise in molecular dipole moment that reshapes the local electrostatic landscape.[38,39]" (**Page 10**, in the revised manuscript)

We have also carefully reviewed the full manuscript to ensure concise and reader-

accessible language throughout.

- In the same paragraph the wording bandgap homogenization is used, but the bandgap had not been named described so far in the text. Please clarify pi-sigma interaction and name bandgap and shifts for use of passivants.

***Response*:** Thanks for this insightful comment.

(1) Based on the reviewer's comments, we reconsidered the interpretation related to bandgap homogenization. Our experimental results indicate a reduction in the interfacial energetic barrier; however, in the absence of direct evidence for bandgap variations among different micro-regions within the perovskite film, we acknowledge that invoking bandgap homogenization is not sufficiently supported by the current data. Accordingly, we have revised the corresponding description and replaced "bandgap homogenization" with "a reduction in the interfacial energetic barrier" throughout the manuscript.

(2) We have clarified both the physical meaning of the π–σ interactions and the definition of "bandgap homogenization." Specifically, the σ-type interaction corresponds to Pb–N coordination bonding, which provides localized defect passivation. This assignment is well supported by classic and recent literature reports (*J. Solid State Chem.* 1994, 111, 370–379; *APL Mater.* 2018, 6, 114203). In contrast, the π-type interaction corresponds to Pb–I–π coupling, which promotes charge-density delocalization and enhances interfacial electrostatic coupling (*Nat. Chem.* 2023, 15, 1745–1753).

(3) Based on the reviewer's valuable suggestion, we have incorporated the above references into the revised manuscript to support and clarify the interpretation of the π/σ interactions. The corresponding revisions are detailed below:

"and multicenter coordination-induced electronic microenvironment enhancement.[40,41] (*Nat. Chem.* 2023, 15, 1745–1753; *J. Solid State Chem.* 1994, 111, 370–379; *APL Mater.* 2018, 6, 114203)" (**Page 14**, in the revised manuscript)

- The bandgap of the absorber/formula should be mentioned early in the text.

***Response*:** We appreciate the reviewer's suggestions. We have updated the manuscript to explicitly detail the nominal perovskite compositions employed.

(1) The compositions used for the core characterizations and devices are:

- **Normal-bandgap $FAPbI_3$-based perovskite (1.56 eV):** $FA_{0.85}MA_{0.1}Cs_{0.05}Pb(I_{0.98}Br_{0.02})_3$
- **Wide-bandgap perovskite (1.68 eV):** $Cs_{0.22}FA_{0.78}Pb(I_{0.85}Br_{0.15})_3$

The corresponding text sections have been revised in the Supplementary Information and main text:

"Normal-bandgap $FAPbI_3$-based perovskite (1.56 eV) solution (1.5 M, $FA_{0.85}MA_{0.1}Cs_{0.05}Pb(I_{0.98}Br_{0.02})_3$) was prepared…

Wide-bandgap perovskite (1.68 eV) solution (1.3 M, $Cs_{0.22}FA_{0.78}Pb(I_{0.85}Br_{0.15})_3$) was prepared…" (1.2 Perovskite precursor solution, in the Supplementary Information).

(2) Regarding the fundamental characterizations, all measurements were performed using a normal-bandgap perovskite composition ($FA_{0.85}MA_{0.1}Cs_{0.05}Pb(I_{0.98}Br_{0.02})_3$). To further clarify this point for readers, we have added an explicit description in the film characterization section of the Supplementary Information.

"Unless otherwise specified, all characterizations were performed on $FAPbI_3$-based perovskite ($FA_{0.85}MA_{0.1}Cs_{0.05}Pb(I_{0.98}Br_{0.02})_3$) films fabricated following our standard one-step spin-coating protocol." (1.5 Film characterization, in the Supplementary Information)

"The XPS spectra of calibrated C 1*s* and F 1*s* references for $FAPbI_3$-based perovskite with ligands are provided in **Supplementary Figs. 6** and **7**." (**Page 7**, in the revised manuscript)

"analyses of $FAPbI_3$-based perovskite films verified that …" (**Page 9**, in the revised manuscript)

"Following our device protocol, we spin-coated the ligand solution onto the $FAPbI_3$-based perovskite film …" (**Page 12**, in the revised manuscript)

- As mentioned it is recommended to add a control group for stability testing outdoor and EQE graphs for a cell per group using the passivant variation.

***Response*:** We agree with the comment has made revisions accordingly, as detailed in our response to Section D.

- Please add more info on sample preparation, e.g. H-NMR, FRIT, and always clarify which kind of sample stack was used, e.g. XPS, XRD, PL,etc.

***Response*:** Thanks, we have added **Supplementary Note 2** to the Supplementary Information to provide further details on the sample preparation procedures for XPS, HNMR, and FTIR.

(1) "XPS measurements and sample preparation: Thin films for XPS characterization were prepared using the same deposition procedures as for functional devices. Following surface ligand modification, all samples were handled entirely within a nitrogen-filled glovebox and transferred to the XPS analysis chamber via an airtight transfer vessel under continuous N_2 atmosphere. This protocol minimized exposure to ambient air, light, and heat, thereby ensuring surface integrity prior to measurement. Spectra were acquired using a monochromatic Al Kα X-ray source (1486.68 eV) with the analysis chamber base pressure maintained between 1.0×10^{-9} and 5.0×10^{-8} mbar. High-resolution scans were recorded at a pass energy of 20 eV." (In the updated **Supplementary Note 2**)

(2) "NMR measurements and sample preparation: To investigate the coordination interaction between the passivating ligands (TFEA, TFBA, TFPA, and TFPmA) and PbI_2, liquid-state ^1H NMR experiments were carried out in deuterated DMSO (d_6-DMSO). Each ligand and its equimolar mixture with PbI_2 (1:1 molar ratio) were dissolved in d_6-DMSO and analyzed by ^1H NMR. Upon addition of PbI_2, distinct upfield shifts were observed for the amidino and aromatic proton signals, indicating strong ligand–Pb^{2+} coordination. These ligand-dependent spectral changes reveal a clear hierarchy of coordination strength that correlates with the trends in defect passivation and device performance." (In the updated **Supplementary Note 2**)

(3) "FTIR measurements and sample preparation: FTIR measurements were performed on KBr pellets (sample:KBr = 1:100) pressed under vacuum, with spectra acquired from 4000–400 cm^{-1} at 0.4 cm^{-1} resolution. Interaction with PbI_2 broadened the –C=N stretching vibration, with FWHM increases of 49% (TFEA), 51% (TFBA), 59% (TFPA), and 107% (TFPmA). This trend reflects stronger ligands-perovskite interactions at the presence of multicenter interactions (TFBA, TFPA, TFPmA)." (In the updated **Supplementary Note 2**)

Additionally, we have included descriptions of the perovskite components used for characterization and the thin-film preparation process in the main text.

"(see details in **Supplementary Note 2**)." (**Page 7**, in the revised manuscript)

- Please check carefully that claims are backed up with linking Figures or adding references. E.g. "effectively dissipating charge extraction differences from grain boundaries and grains", please clarify to which data or reference you refer to; or in "optimizing ligand adsorption topology enables the establishment of sub-nanometer charge transport pathways and stronger pero/ligand/C60 electron coupling, leading to more efficient charge extraction" the data on charge extraction has not been shown yet, so rather link/refer or change wording to prerequisite for efficient charge extraction.

***Response*:** We appreciate your careful reading.

(1) These sentences have been revised to avoid implying a direct experimental verification of charge extraction efficiency. Accordingly, the revised sentence now reads:

"The improved surface smoothness and potential homogenization, driven by the N-heteroaromatic motifs, enhance the contact-related interfacial quality and is beneficial for interfacial charge extraction." (**Page 9**, in the revised manuscript)

(2) Furthermore, our DFT calculations indicate enhanced electrostatic coupling across the perovskite/ligand/C_{60} interface, which are favorable prerequisites for efficient charge extraction. Accordingly, the revised sentence now reads:

"Theoretical simulation reveals that the ligand adsorption topology opens both sub-nanometer charge-transfer channels and enhances the perovskite-ligand-C_{60}

electrostatic coupling, yielding an interfacial electronic landscape that favours efficient charge extraction." (**Page 5**, in the revised manuscript)

- Please reconsider the wording "underscoring operational robustness" in the conclusion. Though the stability data looks promising compared to the control device, no comparison for the outdoor data is given and 130 days with ~7% efficiency loss is still far from what would be needed for "commercial-scale modules" as named in the sentence after. A critical few on what still needs to be improved in this regard is recommended.

Response**:** Thank you for this valuable comment.

(1) To address the concern, we have added outdoor stability data for the control device, which shows 24.6% efficiency degradation after 27 days of continuous operation under actual outdoor conditions (**Figure R83).**

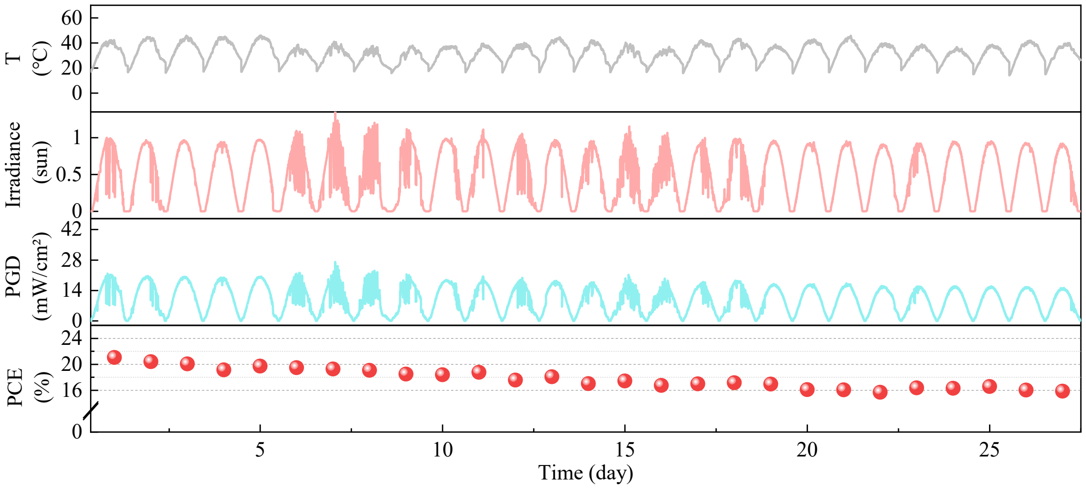

Figure R83 The real-time field testing of control group under actual out-door operating conditions for 27 days. (**Supplementary Fig. 53** In the Supplementary Information)

(2) In addition, we continued the outdoor operational monitoring of the optimized devices, extending the testing duration to **258 days**. The device efficiency decreased to 19.82%, corresponding to a 14.2% loss relative to the initial efficiency of 23.1%. To our knowledge, this represents the **first >23%-efficiency perovskite module to demonstrate such prolonged operational stability**, as shown in **Figure R84.**

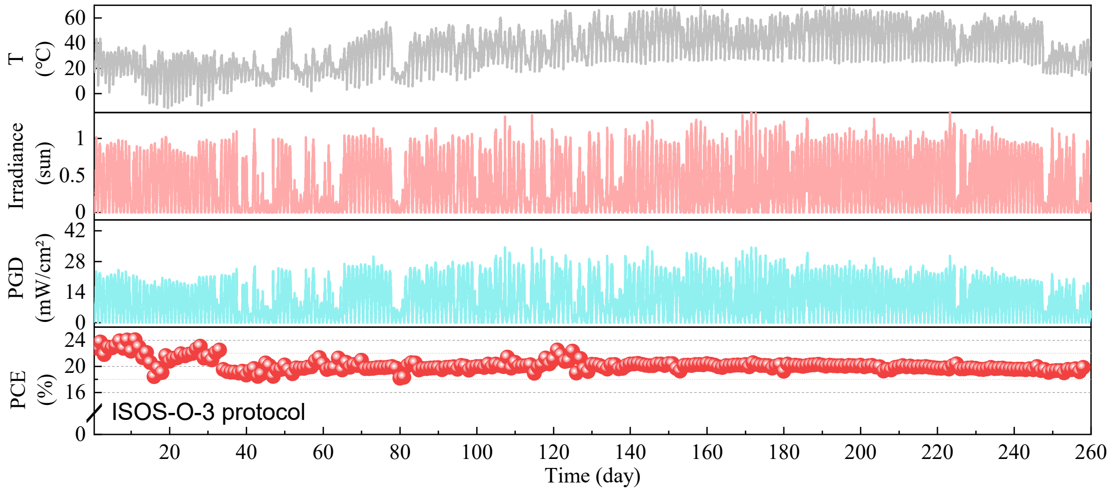

Figure R84 The real-time field testing of TFPmA-treated module under actual out-door operating conditions for 258 days. (**Fig. 5g**, in the revised manuscript)

(3) These outdoor, real-world operational data further substantiate the significant enhancement in long-term device stability enabled by the optimized ligand treatment. Accordingly, **Figure R84** as been incorporated into the main text as **Fig. 5g**, and the relevant sections of the manuscript have been updated to reflect these results, as detailed below:

"(**Fig. 5g, Supplementary Figs. 51** and **52**): Subjected to 258 days of continuous operation with temperature cycling (–11.5 to 68.9 °C) and irradiance fluctuations (0–1.39 sun; plane of array global irradiance: 0–34.7 mW cm^{-2}), the TFPmA-treated module exhibited only a 14.2% loss (from 23.10% to 19.82% PCEs). In sharp contrast, the control module degraded by 24.6% (from 21.03% to 15.85% PCEs) after merely 27 days of outdoor testing (**Supplementary Fig. 53**). The results from ISOS-D-2 testing, ISOS-L-1 testing, and real-time outdoor field testing collectively demonstrate that …" (**Page 16**, in the revised manuscript)

(4) Regarding the phrases "underscoring operational robustness" and "commercial-scale modules" in the Conclusion, we have made the corresponding revisions as detailed below:

"Extended outdoor validation reveals an interfacial robustness during 258 days real-time field testing. This atomic-scale interface control paradigm provides new design principles for minimizing interfacial energy losses to advance perovskite photovoltaics toward their theoretical efficiency limit while ensuring scalability and durability." (**Page 18**, in the revised manuscript)

G. References

More references with regard to the molecular design would be recommended to clarify novelty, as mentioned above. Also consider including recent reviews on passivant design to strengthen the introduction.

***Response*:** We appreciate your valuable suggestion. In the revised manuscript, we have substantially expanded the Introduction to better position our work within the context of recent advances in interfacial ligand engineering. Specifically, we have added several new references on conjugated and planar-aligned passivation molecules and recent comprehensive reviews on molecular passivant design strategies (*Nature* 2022, 611, 278–283; *Energy Environ. Sci.* 2022,15, 4700-4709; Science 2024, 384, 189–193; *Nat. Commun.* 2025, 16, 1042; Joule 2025, 9, 102009; *Adv. Mater.* 2025, 2507918). These additions clarify the originality of our stereoelectronic manipulation approach relative to existing molecular design concepts and strengthen the overall scholarly framing of the study. The revised paragraph in the Introduction are as follows:

"Yet, even state-of-the-art remedies including those employing vertical ligands or geometric size-matching passivants,[13,24-26] leave this fundamental dilemma unresolved." (**Page 3**, in the revised manuscript)

H. Clarity and Context

The manuscript is concise but as mentioned the abstract could be sharpened in terms of clear naming of the molecular moieties varied, the language could be simplified or clarified. A critical check of the term fundamental paradox is recommended paired with a clarification of existing literature. A clear statement of the hypothesis is recommended after checking this. As mentioned above check the appropriate wording for stability data in the conclusion.

***Response*:** Following the suggestion, we have critically revised the Abstract, Introduction, and Conclusion to improve clarity, precision, and conceptual framing. Additionally, we have supplemented the existing literature with relevant references.

(1) Regarding the abstract, we have provided further elaboration on the introduced molecules.

"By strategically replacing benzene carbons with nitrogen atoms to create pyridine or pyrimidine rings," (**Abstract**, in the revised manuscript)

(2) Regarding the statement on the fundamental paradox, we have revised the relevant sections:

"This trade-off poses a fundamental challenge in molecular design:" (**Page 3**, in the revised manuscript)

(3) "Yet, even state-of-the-art remedies including those employing vertical ligands or geometric size-matching passivants,[13,24-26] leave this fundamental dilemma unresolved." (**Page 3**, in the revised manuscript)

(4) As mentioned above, we have revised the conclusion section as follows:

"This work demonstrates that the ligand adsorption topology on perovskite surfaces, governed by ligand-perovskite interactions, enables minimization of interfacial energy losses. Introducing N-heteroaromatic motifs into ligands triggers a stereoelectronic reconfiguration from vertical to planar alignment via synergistic Pb-N coordination and

Pb-I-π interactions. This topology change concurrently annihilates defects while sustaining sub-nanometer charge transport channels, achieving certified 27.41% PCE (reverse scan), with a stabilized output power of 26.85%. Notably, the strategy is both bandgap-universal and readily scalable, delivering high efficiencies for wide-bandgap (1.68 eV) devices and large-area cells and modules. Extended outdoor validation reveals an interfacial robustness during 258 days real-time field testing. This atomic-scale interface control paradigm provides new design principles for minimizing interfacial energy losses to advance perovskite photovoltaics toward their theoretical efficiency limit while ensuring scalability and durability." (**Page 18,** in the revised manuscript)

List of point-to-point response of reviewers' comments

Response**:** We are very pleased that all three reviewers are satisfied with the improvements. We are also grateful to them for raising several minor points that need clarification. Below, we provide a point-by-point response to each comment and describe the corresponding revisions made to the manuscript. We thank the reviewers again for their time and consideration.

Referee #1 (Remarks to the Author):

I really appreciate the extensive effort made by the authors in revising the manuscript and providing detailed responses to all previous comments. The manuscript has clearly improved and many of the initial concerns have been satisfactorily addressed. In particular, the revised computational methodology strengthens confidence in the reported energetic trends. The expanded XPS analysis and control experiments convincingly rule out alternative interpretations related to stoichiometry or charging. The moderation of previously overstated mechanistic claims is appreciated and results in a more balanced interpretation of the data. The additional stability data significantly improve the practical perspective of the work.

However, a few aspects would still benefit from minor clarification before final acceptance:

1. Cross-technique consistency:

While the authors have clarified the coexistence of Pb–N and Pb–I–π interactions, the quantitative weight of each contribution remains somewhat qualitative. A short clarifying paragraph in the main text summarizing the relative contribution inferred from the different techniques would improve coherence.

Response**:** We thank the reviewer for this insightful suggestion. We agree that clarifying the relative contributions of Pb–N coordination and Pb–I–π interactions would improve cross-technique consistency and mechanistic clarity. Based on the combined evidence from DFT calculations and complementary spectroscopic analyses (XPS, FTIR, HNMR and optical measurements), we infer that Pb–N coordination primarily contributes to defect passivation by anchoring undercoordinated Pb^{2+} sites, whereas Pb–I–π interactions play a more prominent role in facilitating electronic delocalization and interfacial charge coupling. Although a strict quantitative separation is challenging, the consistent trends observed across multiple techniques support this functional differentiation.

Accordingly, we have added a concise summary in the main text to clarify this point. "topographic defect healing through multicenter interactions via Pb-N coordination, delocalized electronic screening mediated by Pb–I–π interactions" (Page 10, in the revised manuscript)

2. Statistical treatment of the devices:

The statistical treatment of the device data appears generally sound, and the reported improvements are substantial. However, for completeness, the authors should clarify

whether the reported uncertainties correspond to standard deviation or standard error of the mean and explicitly state the number of independent devices (n) in the relevant figure captions.

Additionally, for the impedance spectroscopy analysis, providing the equivalent circuit model and reporting fitting uncertainties would further strengthen the quantitative rigor of the study.

***Response*:** We thank the reviewer for this valuable suggestion.

For the statistical analysis of device performance, we have clarified that the reported uncertainties correspond to the standard deviation (s.d.) of independent devices. A total of 20 independent devices (n = 20) were measured for each condition. These details have now been explicitly stated in the main text (page 15, Line 17), as well as in the captions of **Supplementary Fig. 43** and **Table S8**, where both the average values and standard deviations are reported.

For the impedance spectroscopy (EIS) analysis, the equivalent circuit model (R(CR)) used for fitting has been provided as an inset in **Supplementary Fig. 36**. The fitting uncertainties were obtained using ZSimDemo software. The extracted relative errors for the control, TFEA-, TFBA-, TFPA-, and TFPmA-treated devices are 6.8%, 2.9%, 2.6%, 4.8%, and 1.4%, respectively. To improve the quantitative rigor of the analysis, we have added the corresponding description to the figure caption as follows:

"**Supplementary Fig. 36|** The Nyquist plots of the control and ligands-treated devices measured under dark conditions. The inset shows the equivalent circuit model (R(CR)) used to fit the impedance spectra, where R_s represents the series resistance, R_{rec} corresponds to the recombination resistance and C_{rec} denotes the recombination capacitance. The extracted fitting uncertainties for the control, TFEA-, TFBA-, TFPA-, and TFPmA-treated devices are 6.8%, 2.9%, 2.6%, 4.8%, and 1.4%, respectively." (In the revised **Supplementary Fig. 36** caption)

3. Energetic interpretation:

The electrostatic explanation for the UPS shifts is reasonable, but it would benefit from a clearer schematic energy-level diagram in the main text to guide the reader.

***Response*:** We thank the reviewer for this helpful suggestion. The energy-level diagram of the device is presented in **Fig. 3e**. To improve clarity and better guide the reader, we have revised the corresponding figure caption to explicitly indicate that the energy levels are derived from UPS measurements and optical absorption data. The revised description as follows:

"(e) Energy-level diagrams comparing the valence- and conduction–band edges of untreated (control) versus ligands-treated perovskite films, derived from UPS measurements and optical absorption data." (Page 12, in the revised manuscript)

These are relatively minor points that do not undermine the central conclusions of the work. If the authors address these clarifications appropriately in the revised manuscript, I do not consider a further round of review necessary. Overall, the manuscript is now substantially improved and, upon satisfactory implementation of the above minor revisions, can be considered suitable for publication.

***Response*:** We sincerely thank the reviewer for the positive evaluation of our work and for the careful and professional assessment throughout the review process. The insightful comments have significantly helped improve the quality and clarity of the manuscript. We greatly appreciate the reviewer’s time and consideration.

Referee #2 (Remarks to the Author):

The authors have made significant efforts to address my previous concerns, and I appreciate the additional clarity that the authors incorporated to the manuscript. After reading the revised files, I have additional minor questions, which I would appreciate the feedback from the authors.

1. Page 9, Lines 8-9: Authors state “dissipation of surface-aggregated PbI2 domains after interfacial treatment” from Supplementary Fig. 17 images. How can authors identify PbI2 domains solely from the SEM images? Can authors consider adding justification/clarifications in the caption of Supplementary Fig. 17 for the above statement?

***Response*:** We thank the reviewer for this suggestion. We have run additional experiments to confirm the PbI_2 domain.

(1) We performed EDS analysis on the perovskite film, as shown in **Figure R1**. First, we selected two points (L3, L5) within the grains on the surface of the film, and two additional points (L2, L9) in the bright, irregular regions at the grain boundaries. EDS elemental analysis revealed that, aside from carbon (C), we detected significant amounts of lead (Pb), iodine (I), and nitrogen (N) at points L3 and L5, with the atomic percentage of N being significantly higher than that of Pb. However, at points L2 and L9, we detected only Pb and I, with no N present. This indicates that the bright, irregular regions at the grain boundaries consist of excess PbI_2. In addition, in our manuscript, the GIWAXS (Supplementary Fig. 14, where a characteristic diffraction feature at $q \approx 0.9$ Å^{-1}) and XRD (Supplementary Fig. 15, which shows a diffraction peak at $2\theta \approx 12.8°$) results are also confirmed the exist of PbI_2.

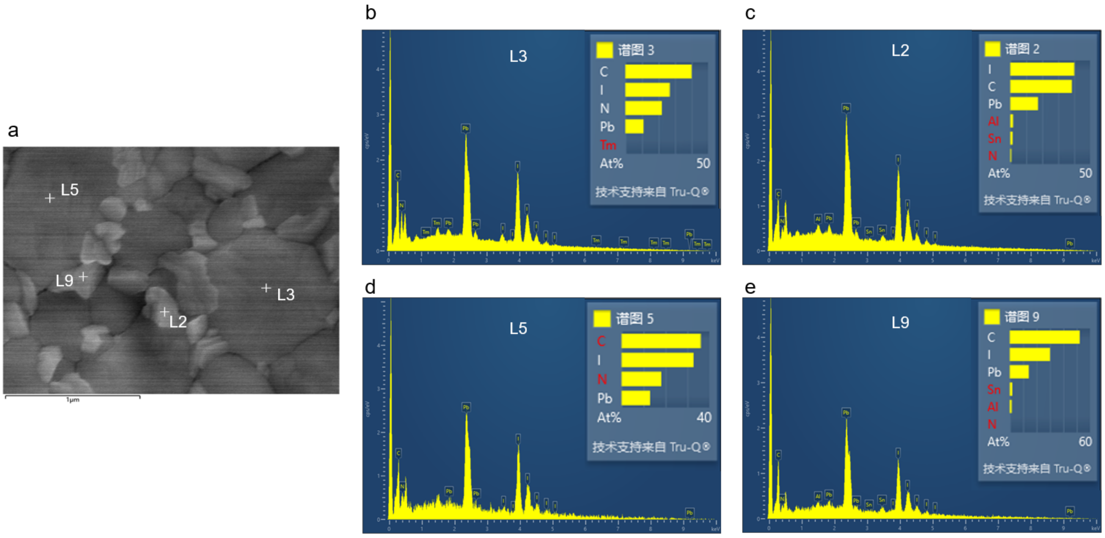

Figure R1 The EDS results of different points on the surface of perovskite film with excess PbI_2.

(2) Based on the above structural characterizations and prior reports [**Nature** 2022, 611, 278–283; **Nature** 2023, 620, 545–551; **Nature Energy** 2024, 9, 1540–1550; **Nature Energy** 2025, 10, 342–353], these features are attributed to surface-aggregated PbI_2 domains. After ligand treatment, the density and size of these bright domains are significantly reduced, suggesting a redistribution or consumption of excess PbI_2. This interpretation is further supported by the decreased intensity of the PbI_2 diffraction peak in XRD (Supplementary Fig. 15).

To address the reviewer's concern, we have added a brief clarification in the caption of Supplementary Fig. 17 to explicitly clarify the basis of this assignment.

"**Supplementary Fig. 17|** The SEM images of the control and ligands-treated perovskite films. The scale bar is 500 nm. In contrast to the control sample, there is a distinct presence of PbI_2 (Bright, irregular domains) at the grain boundaries. After ligand treatment, these domains are significantly reduced, indicating redistribution or dissipation of excess PbI_2 and a more homogeneous surface morphology." (In the revised **Supplementary Fig. 17** caption)

2. Page 9, Lines 9-11: Authors state "atomically smooth interfaces with ~1.4-1.7 nm reduced RMS roughness" from Supplementary Fig. 18. The histograms in Supplementary Fig. 18 seems to provide similar height distribution in the range of -40 to +40 nm. Please provide in the caption of Supplementary Fig. 18 the rationale how the range of reduced "~1.4-1.7 nm" was calculated.

Response**:** Thanks reviewer for the comments. In Supplementary Fig. 18, all AFM images were analyzed using the NanoScope Analysis 3.0 software. The histograms represent the height distribution of the film surface over the scanned area, while the reported roughness values correspond to the root-mean-square (RMS) roughness calculated from these height distributions. Specifically, the control sample exhibits an RMS roughness of 11.7 nm, whereas the ligand-treated films show reduced RMS values of 10.3, 10.2, 10.0, and 10.3 nm. The reported reduction of ~1.4–1.7 nm is therefore derived from the difference between the control and ligand-treated samples.

To avoid potential confusion arising from the similar height distribution ranges in the histograms, we have added the following explanation in the caption of **Supplementary Fig. S18**:

"**Supplementary Fig. 18|** The AFM images and corresponding height sensor distribution for the control and ligands-treated perovskite films. Compared to the control sample, which had a root-mean-square (RMS) roughness of 11.7 nm, the roughness of the ligands-modified films decreased by 1.4, 1.5, 1.7, and 1.4 nm, respectively." (Caption of Supplementary Fig. 18, in the revised Supplementary Information)

3. Supplementary Fig. 23 and Fig. 3c are repeating. Please confirm whether Supplementary Fig. 23 can be replaced by the "statistical analysis" (mentioned in the caption) based on Fig. 3c and Supplementary Table 1(?).

Response**:** We thank the reviewer for this helpful suggestion. Supplementary Fig. 23 presents the statistical analysis of the aggregation domain sizes extracted from the red

regions in Fig. 3c. To avoid redundancy, we have removed Supplementary Fig. 23 from the Supplementary Information and incorporated the corresponding statistical description into Supplementary Table 1. The caption of Supplementary Table 1 has been revised as follows:

"**Supplementary Table 1 |** Statistical analysis of nanoscale aggregation domains with different sizes, extracted from distinct regions of nano-IR mapping (**Fig. 3c**) of ligand-treated perovskite films." (In the revised **Supplementary Table 1** caption)

4. In the Methods section (1.5 Film characterization), please provide further details with equations for the sample work function calculations from CPD values based on KPFM measurements (Fig. 3a). This would make clearer for readers when authors compare with work function values determined from the UPS results (Supplementary Fig. 24 and Supplementary Table 2).

***Response*:** We thank the reviewer for this helpful suggestion. We have now added a detailed description in the Methods section, including the relationship between the contact potential difference (CPD) measured by KPFM and the work function difference between the tip and the sample, expressed as: CPD = $(\Phi_{tip} - \Phi_{sample})/e$ where Φ_{tip} and Φ_{sample} are the work functions of the tip and the sample, respectively, and e is the elementary charge.

In this work, due to the uncertainty in the absolute tip work function, the KPFM measurements are primarily used to evaluate the relative surface potential distribution and spatial heterogeneity (e.g., mean absolute deviation (Ra) and the standard deviation (σ)), rather than to determine absolute work function values. The absolute work functions are independently obtained from UPS measurements. Importantly, both techniques exhibit consistent trends, where the reduced potential fluctuation observed in KPFM correlates with the more uniform interfacial energy landscape revealed by UPS.

We have added the corresponding description in the revised Methods section (1.5 Film characterization) as follows:

"AFM and KPFM images were obtained using a Bruker Dimension Icon system. The contact potential difference (CPD) measured by KPFM is related to the work function difference between the tip and the sample according to: CPD = $(\Phi_{tip} - \Phi_{sample})/e$, where Φ_{tip} and Φ_{sample} are the work functions of the tip and sample, respectively, and e is the elementary charge. Due to the uncertainty in the absolute tip work function, KPFM is used here to evaluate the relative surface potential distribution and spatial heterogeneity. Statistical parameters, including the mean absolute deviation (Ra) and standard deviation (σ), are extracted from pixel-by-pixel CPD data. " (Page 23, Section Film characterization, in the revised manuscript)

In addition, we also revised the sentence in the main text regarding the correspondence between UPS and KPFM, as follows:

"This trend reflects flattened surface energy undulation, consistent with the reduced spatial potential fluctuations observed by KPFM," (Page 10, in the revised manuscript)

5. Because authors provide the CPD distribution (Fig. 3b, Supplementary Fig. 19), the standard deviation estimation for the CPD values presented in Fig. 3a could be provided.

***Response*:** We thank the reviewer for this valuable suggestion. In the revised manuscript, we have now quantified the statistical dispersion of the CPD values by extracting the standard deviation (σ) from the pixel-by-pixel CPD data. To maintain the clarity of the main figure, we have kept **Fig. 3a** as a direct visualization of the spatial CPD distribution, while the quantitative statistical parameters, including the mean absolute deviation (Ra) and the standard deviation (σ), are now explicitly provided in the caption of **Supplementary Fig. 19**. These values are directly derived from the same datasets used for the CPD histograms shown in Fig. 3a, b and Supplementary Fig. 19.

The reduced σ values consistently confirm the suppressed spatial potential fluctuations upon ligand modification, in agreement with the visibly narrowed CPD distributions. The **updated Fig. 3a** and caption of **Supplementary Fig. 19** are provided below.

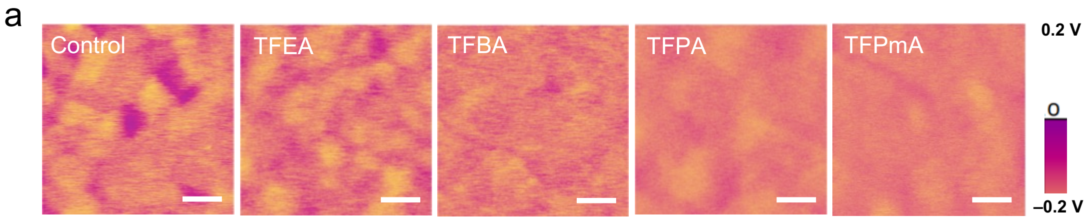

Figure R2 Kelvin probe force microscopy (KPFM) topography showing surface potential variations for control and perovskite films treated with TFEA, TFBA, TFPA, and TFPmA ligands (scale bar: 400 nm). (Page 11, updated Figure 3a, in the revised manuscript)

"**Supplementary Fig. 19|** The potential distribution extracted from the KPFM test for the control and ligands-treated perovskite films. The histograms are constructed from pixel-by-pixel KPFM data and the mean absolute deviation (Ra) and standard deviation (σ) for each sample are summarized as follows: Control (Ra = 30.1 mV, σ = 39.5 mV), TFEA (Ra = 27.1 mV, σ = 33.7 mV), TFBA (Ra = 20.1 mV, σ = 25.4 mV), TFPA (Ra = 14.6 mV, σ = 18.4 mV), and TFPmA (Ra = 11.5 mV, σ = 14.8 mV). The reduced σ values indicate suppressed spatial potential fluctuations upon surface modification." (The revised caption of **Supplementary Fig. 19**)

6. Just to make sure I'm understanding correctly: In Fig. 3d, by changing the color of spectrum labeled as "mid" to green color, would it make easier for readers to directly correlate with the corresponding mapping color shown in Supplementary Fig. 21?

***Response*:** We thank the reviewer for this helpful suggestion. We have revised **Figure 3d** by changing the color of the spectrum labeled "mid" to "green", to improve consistency with the corresponding mapping in Supplementary Fig. 21. The updated figure is provided in the revised manuscript.

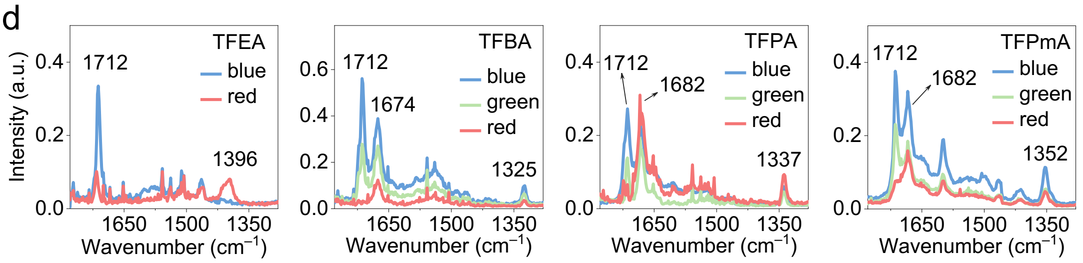

Figure R3 Nano-infrared (nano-IR) line-plot spectra acquired from the perovskite surface mappings (Supplementary Fig. 21, the blue, green, and red lines corresponding to the blue, intermediate, and red mapping, respectively). (Page 11, updated Figure 3d, in the revised manuscript)

7. The field testing under actual outdoor operating conditions are another novelty point in this work. This is not to criticize because of the known challenges involved in the measurements (e.g., weather variations) and analysis. However, I was curious in understanding the variations in the PCE shown in Fig. 5g. From the starting (t = 0) until ~18 days, the PCE seems to keep decaying (from ~24% to 18%), but afterwards, the PCE start to increase and show a rather stable PCE profile. When I compare with the PCE profile of the control sample shown in Supplementary Fig. 53, similar decay (PCE from ~21% to ~16%) is also observed for the first 27 days. I was wondering whether the control sample is also showing similar behavior of PCE recovery after the first 27 days, and it is related to the initial burn-in process. If authors could provide insights for the above point, and further additional insights/discussion from the authors' experiences/challenges in the outdoor testing would be valuable for the readers to attract further attention.

Response**:** We thank the reviewer for this careful and insightful analysis. Some companies and studies have also observed an initial burn-in process in perovskite solar cells during actual outdoor operation (as shown in **Figure R4-R6**), which is consistent with our findings. After the initial burn-in process, the cells enter a relatively stable operating state and exhibit only a gradual decline over an extended period.

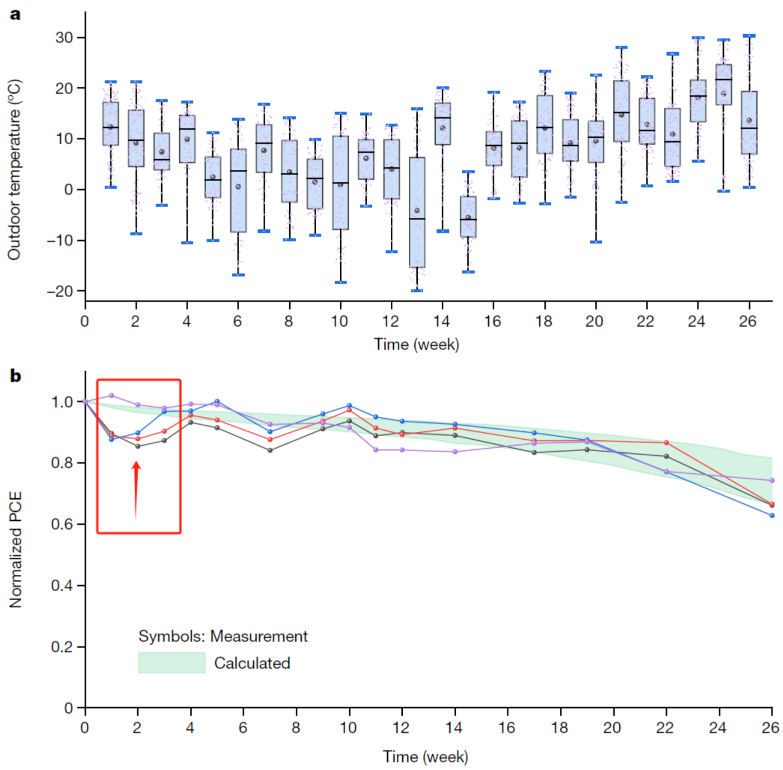

Figure R4 a, Outdoor ambient temperature trend. b, Device efficiency evolution for glass-PIB-packaged PSCs biased near maximum power point under outdoor ageing conditions. (Copyright from ***Nature*** 2023, 623, 313–318)

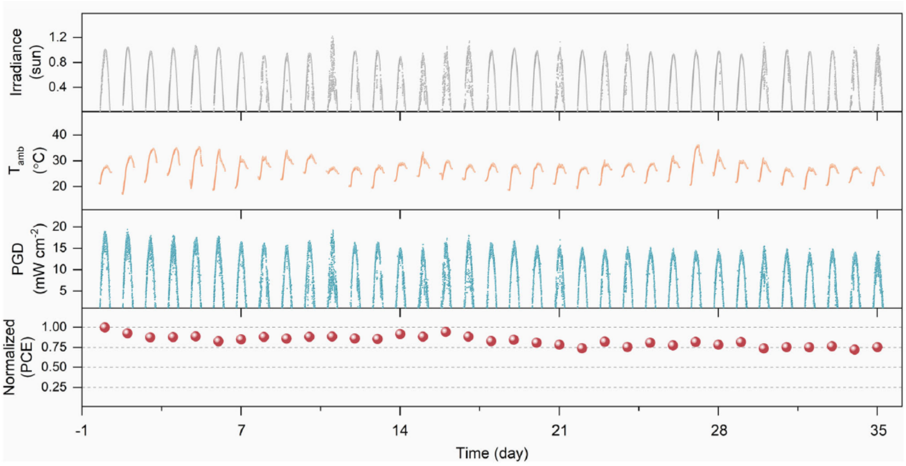

Figure R5 Outdoor stability of a 2D-MAP-ordered device. The outdoor stability measurements were conducted on the KAUST campus in Thuwal, Kingdom of Saudi Arabia, from December 2023 to January 2024 for 35 days. (Copyright from ***Nat. Commun.*** 2025, 16, 1042)

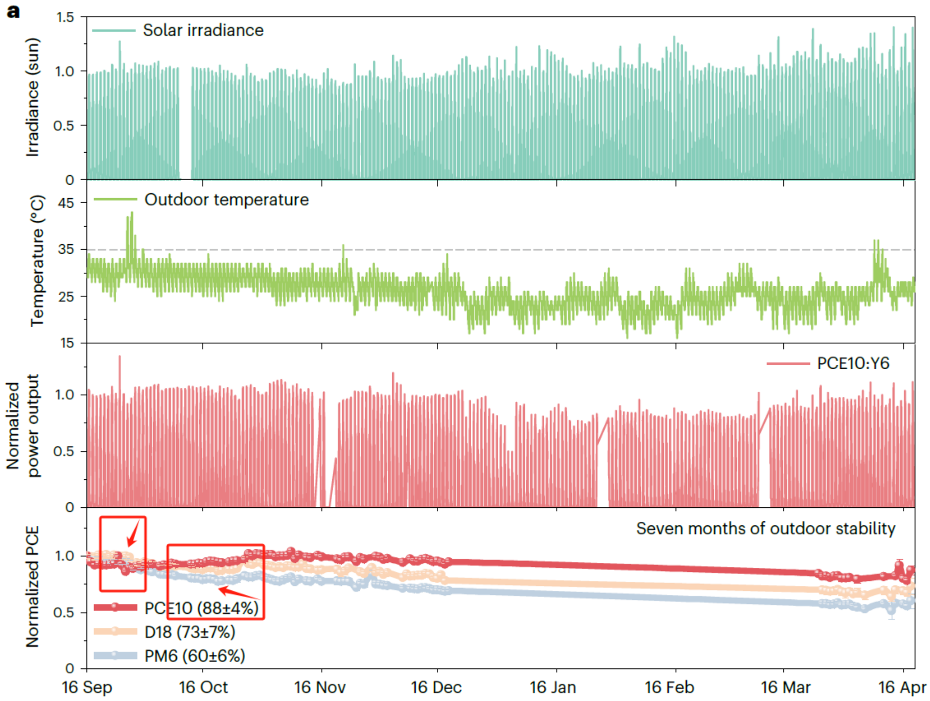

Figure R6 Solar irradiance, normalized power output (the most stable cell), and PCEs of the PCE10:Y6, D18:Y6 and PM6:Y6 cells. (Copyright from ***Nat. Photon.*** 2025, 19, 415–425)

It should also be noted that the outdoor field testing for the control cells occurred from October to November 2025, a period characterized by favorable weather conditions and PGD. During this time, the cells' daily power output was not affected by the weather, resulting in a linear initial burn-in process. In contrast, the outdoor field testing for the target cells began in February 2025, so we observed not only the initial burn-in process but also the impact of weather on daily power output. As shown in **Fig. 5g**, the pronounced PCE drop around day ~18 coincides with a sudden decrease in outdoor temperature, which reached sub-zero conditions. Such abrupt environmental changes can temporarily affect device performance, for example through variations in charge transport, interfacial energetics, and series resistance. When the temperature stabilizes, the device performance correspondingly recovers. Similar fluctuations observed around ~80 and ~130 days can also be correlated with temperature variations during the outdoor testing period.

More generally, outdoor PCE values are influenced not only by intrinsic device stability but also by dynamically changing environmental factors, including temperature, irradiance intensity, and spectral distribution. As a result, fluctuations in PCE under real-world conditions are expected and reflect the complexity of field testing. These results highlight the importance of evaluating device performance under realistic operating conditions and further demonstrate that interfacial engineering plays a critical role in improving the operational robustness of perovskite solar cells.

8. Page 3, Line 7:“are/fundamentally”

Response**:** We thank the reviewer for noting this typographical error. We have corrected “arefundamentally” to “are fundamentally” in the revised manuscript. (Page 3, in the revised manuscript)

9. In the Solar Cells Reporting Summary, the check list regarding to “Current density-voltage (j-V) plots in both forward and backward direction” needs to be updated. Instead of Fig. 5b, is it Supplementary Fig. 38?

Response**:** We thank the reviewer for this helpful suggestion. We have revised the Solar Cells Reporting Summary accordingly and updated the corresponding checklist entry. The reference has been corrected from Fig. 5b to Supplementary Fig. 37.

“The J-V plots measured along both scan direction, as shown in Figure 5c and Supplementary Fig. 37.” (In the section of “Current density-voltage (j-V) plots in both forward and backward direction”)

Referee #3 (Remarks to the Author):

The authors have responded in a very careful manner to the reviewer´s question, providing additional data, explainations, and references. Answers are satisfactory and the quality of the manuscript has further increased. One point which stays unclear/critical to me is the different, not expected shape of the third party EQE measurement (compared to reasonable shape inhouse data) that can influence setting of lamps/ mismatch factor calculation and thus certified values under 1.5AMG standard testing conditions. It would be recommended to resolve this issue, other then that the paper is meeting the level for publication.

Response**:** Thanks reviewer for this important observation. We would like to clarify that the curve provided in the **Supplementary Fig. 42** (now updated as **Supplementary Fig. 41**) corresponds to the spectral responsivity (SR) measured by the accredited certification laboratory, not the external quantum efficiency (EQE) spectrum. While SR and EQE are closely related (**$SR(\lambda) = EQE(\lambda) \times q/h\nu$**), they are not identical representations.

In certification measurements, the SR spectrum is derived from calibrated spectral response measurements that are closely related to EQE measurements, but include instrument-specific calibration and standardized processing protocols. As a result, slight differences in spectral shape compared to in-house EQE data can arise.

Importantly, the certified device performance is determined using standardized procedures that include spectral mismatch correction. Based on calibrated reference cells under AM 1.5G conditions and the measured SR curve, the certification laboratory determined a mismatch factor of 1.0043, which is very close to unity. This indicates excellent spectral matching and confirms that the certified PCE values are not affected by the observed spectral differences.

To further clarify this point, we have added the mismatch factor to the corresponding figure caption in the **Supplementary Fig. 41** and explicitly noted that the provided curve represents SR rather than EQE.

"**Supplementary Fig. 41|** Relative spectral responsivity (SR) curves measured at National Institute of Metrology, China (NIM, China), along with the corresponding data table. The calculated spectral mismatch factor is 1.0043, indicating negligible mismatch under AM 1.5G conditions." (In the revised **Supplementary Fig. 41** caption)
